# Tandem Ring Opening/Intramolecular [2 + 2] Cycloaddition Reaction for the Synthesis of Cyclobutane Fused Thiazolino-2-Pyridones

**DOI:** 10.1021/acs.joc.1c01875

**Published:** 2021-11-12

**Authors:** Mohit Tyagi, Dan E. Adolfsson, Pardeep Singh, Jörgen Ådén, Sanduni Wasana Jayaweera, Anna Gharibyan, Jaideep B. Bharate, Anita Kiss, Souvik Sarkar, Anders Olofsson, Fredrik Almqvist

**Affiliations:** † Department of Chemistry, Umeå University, 90187 Umeå, Sweden; ‡ Department of Medical Biochemistry and Biophysics, Umeå University, 90187 Umeå, Sweden

## Abstract

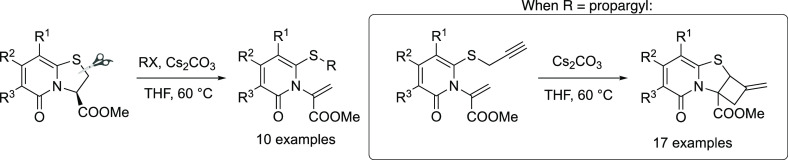

Reaction of thiazoline fused 2-pyridones with alkyl halides in the presence of cesium carbonate opens the thiazoline ring via *S*-alkylation and generates *N*-alkenyl functionalized 2-pyridones. In the reaction with propargyl bromide, the thiazoline ring opens and subsequently closes via a [2 + 2] cycloaddition between an *in situ* generated allene and the α,β-unsaturated methyl ester. This method enabled the synthesis of a variety of cyclobutane fused thiazolino-2-pyridones, of which a few analogues inhibit amyloid β_1–40_ fibril formation. Furthermore, other analogues were able to bind mature α-synuclein and amyloid β_1−40_ fibrils. Several thiazoline fused 2-pyridones with biological activity tolerate this transformation, which in addition provides an exocyclic alkene as a potential handle for tuning bioactivity.

## Introduction

**Figure 1:**
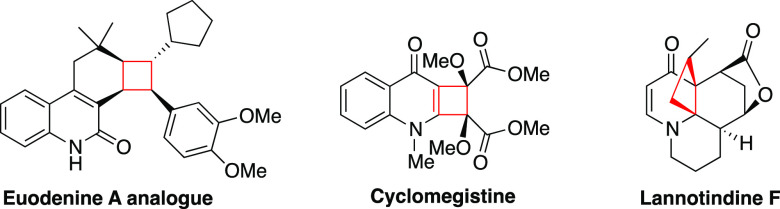
Selected bioactive compounds containing a fused cyclobutane motif.

The direct modification of an existing bioactive scaffold rather than the positioning of substituents is an important strategy to develop compounds with diverse shapes and properties.^1^ Cyclobutanes are an important class of rigid motifs present in a variety of natural products and other biologically important molecules.^2^ A plethora of reactions like [2 + 2] cycloadditions^2c−2e,3^ and rearrangements^4^ have been developed to construct structurally diverse cyclobutane containing scaffolds. Due to their rigid architecture, annulation of a cyclobutane ring with biologically relevant scaffolds like 2-pyridones,^5^ quinolones,^6^ and indoles^2c^ has recently become popular (Figure 1).

**Scheme 1:**
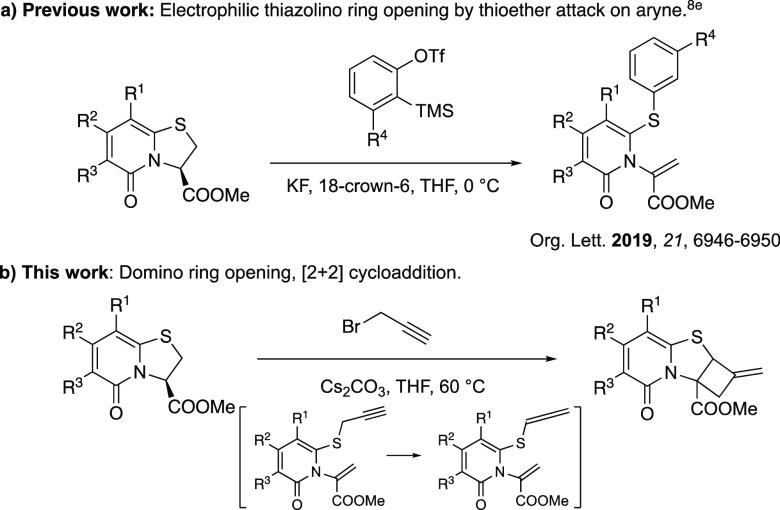
Electrophilic Thiazoline Ring Opening and Its Application in Synthesizing a Variety of Substituted 2-Pyridones (a) Previous work. Aryne induced ring opening. (b) This work. Propargyl bromide triggered ring opening followed by thermal [2 + 2] ring closing cycloaddition.

Thiazoline fused 2-pyridones have found various applications in developing biologically active compounds against *Escherichia coli*, *Chlamydia trachomatis*, *Listeria monocytogenes*, and *Mycobacterium tuberculosis* infections.^7^ We have also demonstrated that rigidification, either by functionalizing the compounds with sterically demanding aryl groups or annulation with heterocycles, has resulted in ring fused 2-pyridones capable of modulating or binding amyloid fibrils.^8^ In a recent report, we demonstrated that the thiazoline ring can be opened by reaction with an aryne to generate *N*-alkenyl-2-pyridones (Scheme 1).^8e^ Knowing that ring opening results in the formation of a Michael acceptor, we envisaged that reaction of thiazolino-2-pyridones with alkyl halides would generate *N*-alkenyl-*S*-alkyl-2-pyridones, which could be used as synthons to build structurally diverse scaffolds.

We further envisioned that annulation of the thiazolino-2-pyridone scaffold with a cyclobutane ring would help in fine-tuning biological activity and may result in improved amyloid binding/modulating properties of the resulting compounds. Intramolecular [2 + 2] cycloadditions of allenes with alkenes constitute a versatile method to synthesize cyclobutane containing rigid bicyclic frameworks.^9^ Since allenes can be prepared from *S*-propargyls,^10^ we planned to open the thiazoline ring with propargyl halides. The resulting *N*-alkenyl-*S*-propargyl-2-pyridone could then be used as a building block to construct a cyclobutane fused thiazolino-2-pyridone via formation of an allene and a subsequent intramolecular [2 + 2] cycloaddition.

## Results and Discussion

**Scheme 2:**
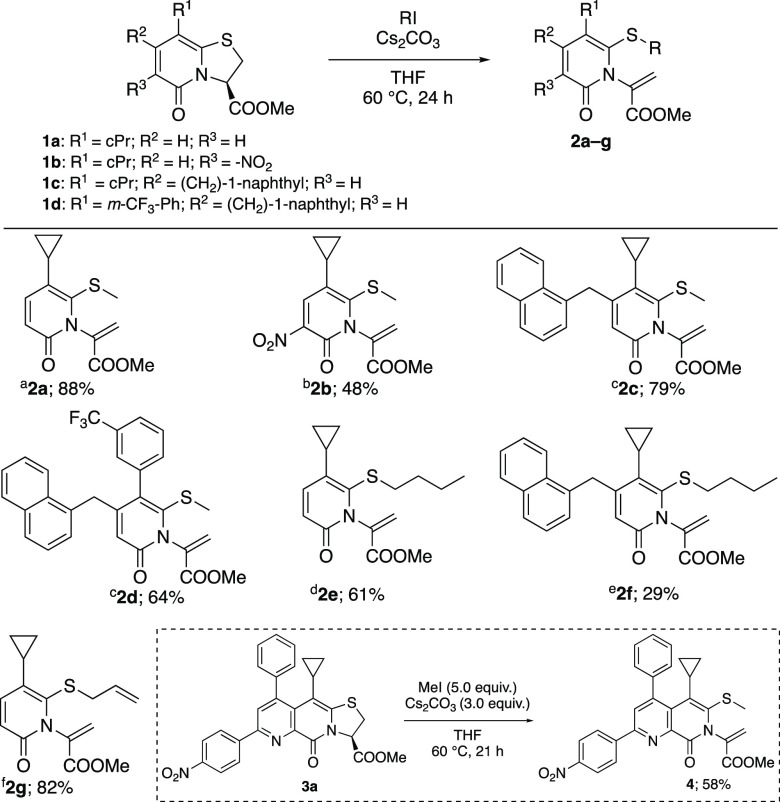
Ring Opening of Thiazolino-2-pyridones with Alkyl Halides 3.0 equiv of methyl iodide. 4.2 equiv of methyl iodide. 9.0 equiv of methyl iodide. 47 h. 9.0 equiv of butyl iodide, 7 days. 4.1 equiv of allyl iodide. All reactions were performed on a 0.5 mmol scale.

To develop the thiazoline ring opening reaction, we commenced our studies by investigating the reaction of **1a** with simple alkyl halides such as methyl iodide. A few bases and solvents were screened to open the ring with methyl iodide (Scheme S1, Supporting Information). Under established conditions, ring opened product **2a** could be obtained in 88% yield by using Cs_2_CO_3_ in THF at 60 °C for 24 h (Scheme 2). To further extend the scope, different alkyl halides and substituted 2-pyridones **1a**–**d** and **3a** were tested under these standardized conditions to give ring opened 2-pyridones **2a**–**g** and **4**.

**Scheme 3:**
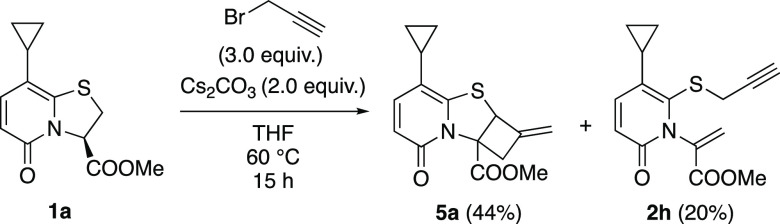
Ring Opening of **1a** with Propargyl Bromide

**Scheme 4:**
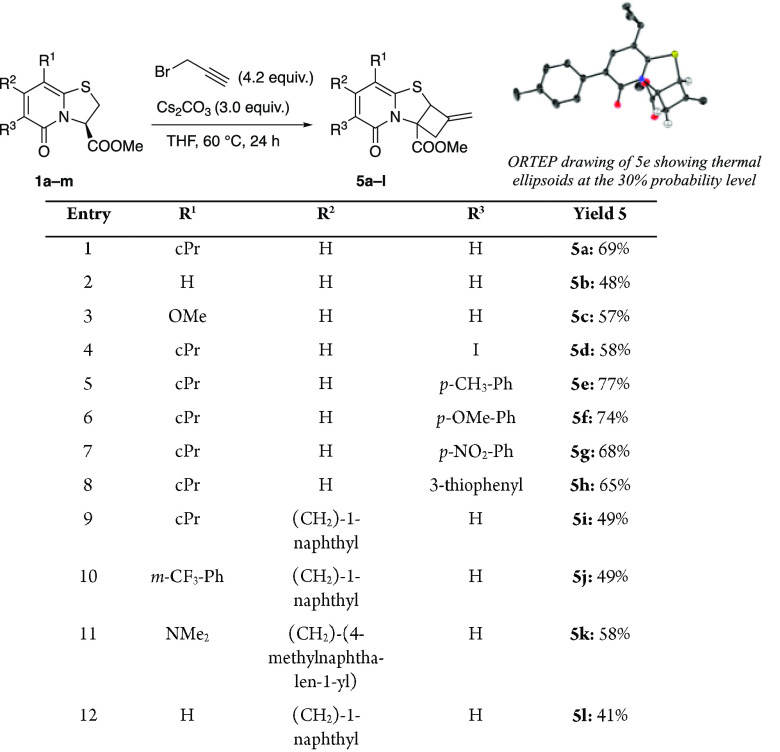
All reactions were performed with 0.5 mmol of **1** at 0.3 M in dry THF. Initially, for 23 h, 2.0 equiv of Cs_2_CO_3_ was added, followed by addition of another 1.0 equiv required for reaction completion. **5e** was crystallized from absolute ethanol and obtained as a racemate.

Next, we attempted ring opening of **1a** with propargyl bromide (Scheme 3). Pleasingly, cyclobutane fused thiazolino-2-pyridone **5a** was formed in 44% yield (as a mixture of enantiomers) together with ring opened product **2h** in 20% yield. To our delight, prolonged heating and use of 3 equiv of Cs_2_CO_3_ gave **5a** exclusively, in 69% yield (Scheme 4). Only starting material was recovered when the reaction was performed in the presence of Na_2_CO_3_ or DIPEA (Scheme S3, Supporting Information). Purified **2h**, when treated with Cs_2_CO_3_ in THF, provided **5a**, which confirms the intermediacy of **2h**.

To evaluate the effect of substituents on the outcome of the reaction, a series of substituted bicyclic thiazolino-2-pyridones was prepared and investigated for their reaction with propargyl bromide (Scheme 4). Compound **5a**–**d** was provided in moderate to good yield. Substrates equipped with an aryl/heteroaryl group as R^3^ substituents reacted smoothly with propargyl bromide to afford 2-pyridones **5e**–**h** in good yields. In line with our previous study,^8e^ low to moderate yields were obtained of **5i**–**l**, with CH_2_-naphthyl groups as R^2^ substituents. A single crystal X-ray diffraction analysis of analogue **5e** verified the structure elucidated by NMR spectroscopy (Scheme 4). When propargyl bromide was replaced with 3-bromo-1-butyne or 4-bromo-1-butyne, no ring opening was triggered. With 1-bromo-2-butyne, only the ring opened product **2i** was provided; no further ring closing was observed (Scheme S4, Supporting Information).

**Scheme 5:**
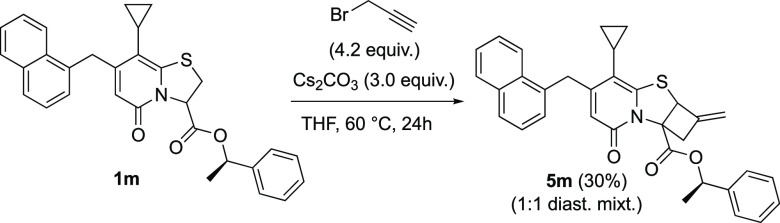
Tandem Ring Opening/Intramolecular [2 + 2] Cycloaddition Using Chiral Ester

The developed intramolecular [2 + 2] cycloaddition between an *in situ* generated allene and the α,β-unsaturated methyl ester gave products as racemic mixtures. To improve diastereoselectivity, sterically demanding chiral esters were prepared using *S*-phenylethanol and menthol. Unfortunately, chiral ester **1m** derived from *S*-(−)-phenylethanol did not influence the diastereoselectivity and cyclobutane fused thiazoloino-2-pyridone **5m** was isolated as a 1:1 diastereomeric mixture (Scheme 5). When l-menthol ester was used as a chiral auxiliary, no ring opening/closing was observed under our standardized conditions (Scheme S5, Supporting Information).

**Scheme 6:**
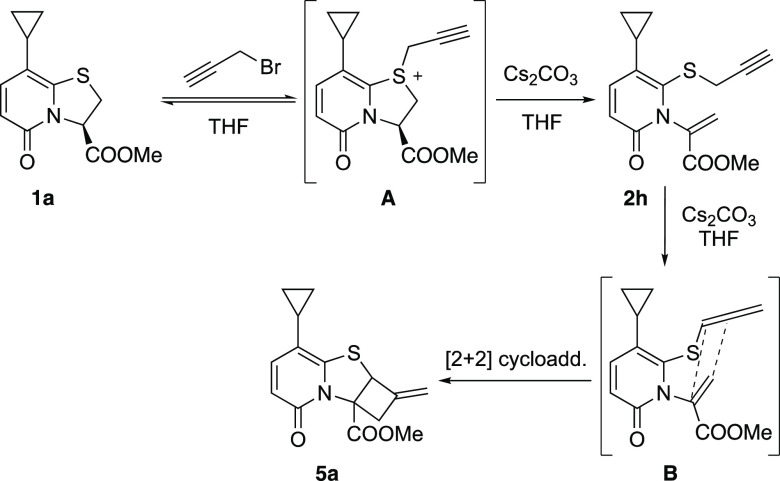
Tentative Mechanism for the Propargyl Bromide Triggered Ring Opening and Subsequent Intramolecular [2 + 2] Cycloaddition

Mechanistically, we propose that nucleophilic attack by the sulfur on propargyl bromide results in the formation of intermediate **A** (Scheme 6) which upon deprotonation by base gives ring opened product **2h**. The intermediate **2h** was isolated and characterized by NMR spectroscopy. Since *S*-propargyls are known to form allene under basic conditions,^10^ it is likely that base promoted abstraction of methylene protons generates allene **B**, which undergoes intramolecular [2 + 2] cycloaddition with the alkene to furnish cyclobutane fused thiazoloino-2-pyridone **5a**.

**Scheme 7:**
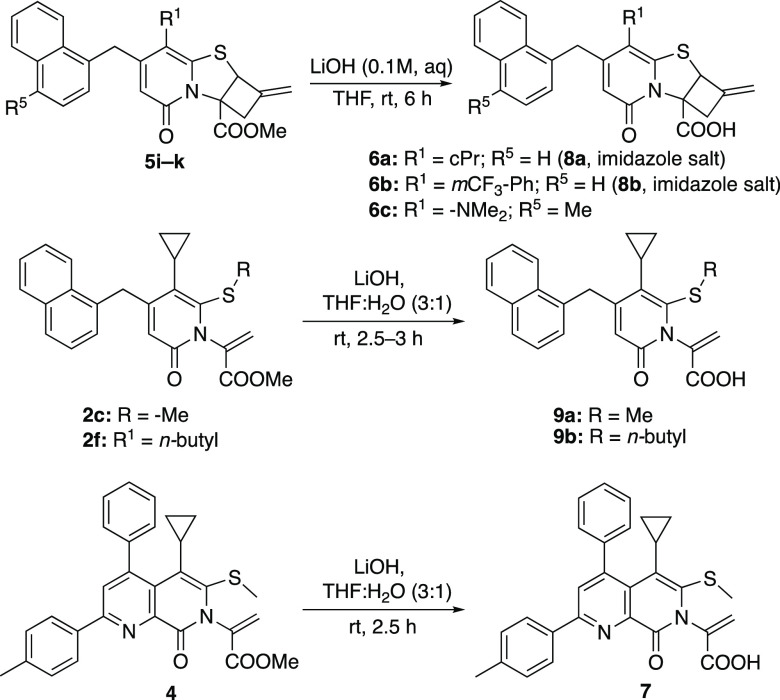
Hydrolysis of Methyl Esters

Knowing that bicyclic and tricyclic thiazolino-2-pyridones have the potential to modulate and bind amyloid fibrils, respectively, cyclobutane fused compounds **5i**–**k** and ring opened 2-pyridones **2c**, **2f**, and **4** were hydrolyzed to their corresponding acids **6a**–**c**, **9a**–**b**, and **7**, respectively (Scheme 7).

**Scheme 8:**
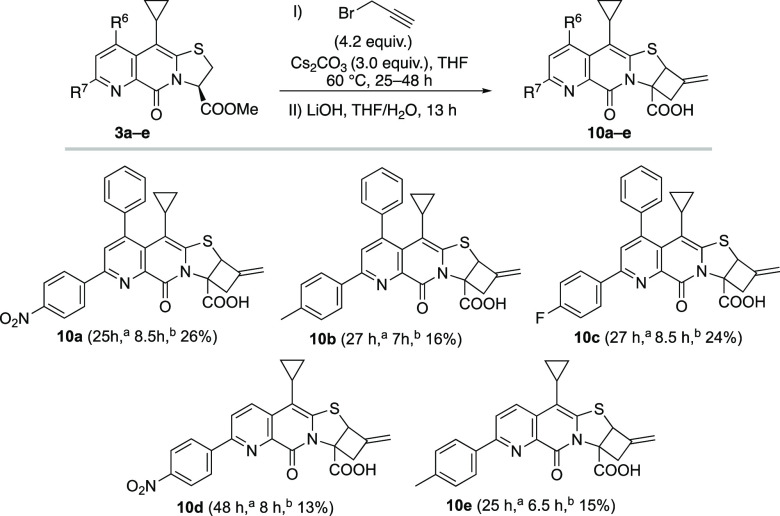
Reaction time for ring opening–closing. Reaction time for ester hydrolysis. All reactions were performed on a 0.25 mmol scale at 0.3 M in dry THF. The mixed esters were, upon purification, directly hydrolyzed to carboxylic acids **10a**–**10e**.

Tricyclic thiazolino-2-pyridones are of therapeutic and diagnostic interest because they have been shown to bind mature α-synuclein and Aβ fibrils.^8d^ Reaction of tricyclic compounds with propargyl bromide (Scheme 8), however, resulted in complex mixtures and the desired cyclobutane fused products were isolated as mixtures of propargyl and methyl esters (perhaps by methyl ester hydrolysis followed by re-esterification with propargyl alcohol). Thus, the mixed esters were saponified directly, using lithium hydroxide, to give **10a**–**e**, in 13–26% yield over two steps.

**Figure 2:**
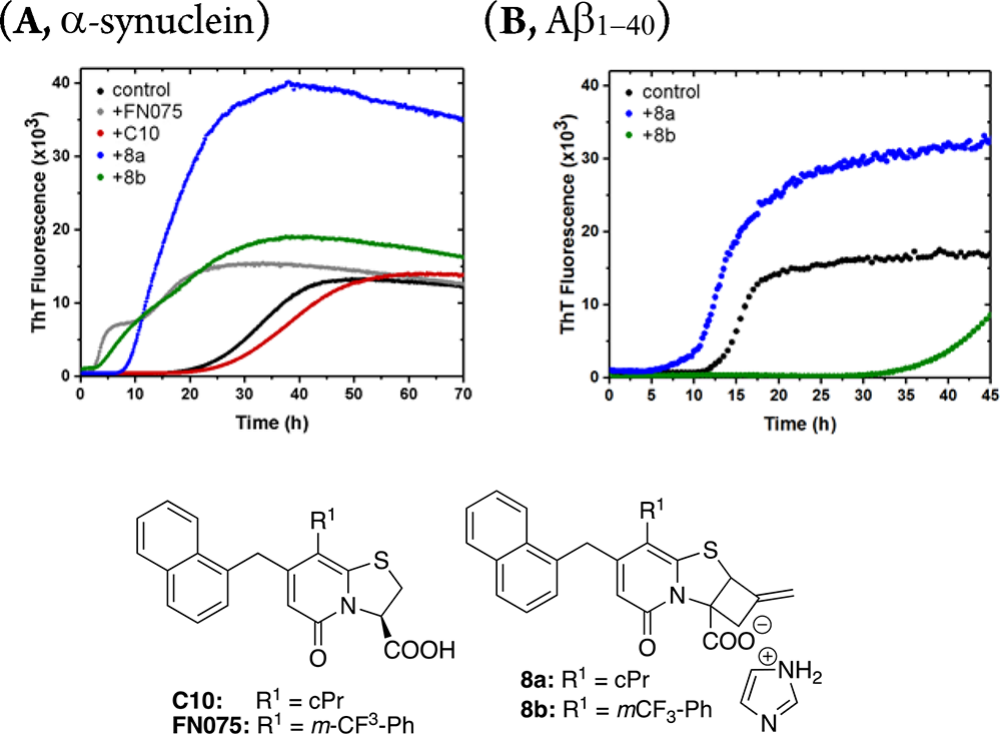
Evaluation of compounds **8a** and **8b** for their effect against (A) α-synuclein and (B) amyloid β_1–40_ fibril formation *in vitro*. In the α-synuclein assay, compound **8a** displays a ThT fluorescence amplitude higher than the control. Both compounds were investigated for whether they modulate the fibers directly, causing the ThT signal to shift. No effect of fiber modulation was found. The higher amplitude seems instead to be a result of altered binding of ThT to the fiber (Figure S16). For control, α-synuclein/amyloid β_1–40_ was incubated in the absence of 2-pyridone.

**Figure 3:**
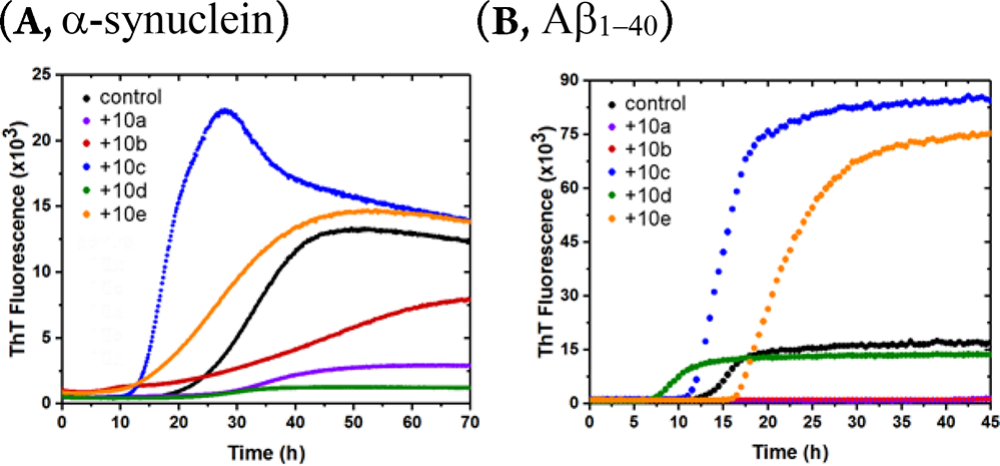
Evaluation of compounds **10a**–**10e** for modulation of (A) α-synuclein and (B) amyloid β_1–40_ fibril formation *in vitro*. For control, α-synuclein/amyloid β_1–40_ was incubated in the absence of 2-pyridone.

Compounds **8a**–**b** (Figure 2), **7**, **9a**–**b** (Figures S9–S12, Supporting Information), and **10a**–**e** (Figure 3) were evaluated for their ability to modulate/bind to α-synuclein and amyloid β_1–40_ fibrils *in vitro*.^8d^ In this assay, the effects on fibril formation are observed as changes of the lag phase duration. Further, the ability to bind α-Syn fibrils and displace fibril bound ThT is indicated by a reduced ThT fluorescence amplitude in comparison to the control experiments, where no peptidomimetic compound is included. Interestingly, both **8a** and **8b** were found to accelerate α-synuclein fibril formation, as indicated by reduction of the lag time (Figure 2A). Compound **8b**, like its parent compound **FN075**, showed strong acceleration of α-synuclein amyloid formation. The cyclopropyl substituted analogue **8a** displayed a milder accelerating effect, while its parent compound **C10** is inactive.^8c^ When tested against amyloid β fibril formation, **8b** inhibited the formation of fibrils like its parent analogue.^8a^

Compounds **10a**–**e** were tested for their effect against α-synuclein and amyloid β_1–40_ fibril formation (Figure 3). 4-Nitrophenyl substituted pyridine fused compounds **10a** and **10d**, like their parent analogues,^8d^ were found to bind mature α-synuclein amyloid fibrils. However, compounds **10b**, **10c**, and **10e** were also found to be very mild accelerators of α-synuclein fibril formation (Figure 3A).

Interestingly, when these compounds were tested for their effect against amyloid β_1–40_ fibril formation (Figure 3B), compounds **10a**–**b** turned out, contrary to their parent compounds which are inactive, to be inhibitors.^8d^

All of the cyclobutane fused thiazolino-2-pyridones were tested as racemates. To investigate the effect of each enantiomer on fibril formation, racemic **6b** was separated to its pure enantiomers using chiral HPLC. When evaluated for their effect on fibril formation *in vitro*, the pure enantiomers were found to modulate α-synuclein and Aβ fibrils equally, to a similar extent as the racemic mixture (Figures S13 and S14, Supporting Information).

## Conclusion

In conclusion, we have prepared *N*-alkenyl 2-pyridones via a thiazoline ring opening reaction with alkyl halides. Reaction of thiazolino-2-pyridones with propargyl bromide gave cyclobutane fused thiazolino-2-pyridones via sequential ring opening, *in situ* allene formation, and intramolecular [2 + 2] cycloaddition. The methodology was also successfully applied to functionalize bioactive tricyclic pyridine fused thiazolino-2-pyridones. The developed methodology transformed inactive compounds to inhibitors of amyloid β_1–40_ fibril formation. Selective modulation of amyloid fibrils by small molecules provides a possible approach in the diagnosis and/or treatment of neurodegenerative diseases,^11^ justifying the importance of such late-stage transformations on thiazolino-2-pyridone peptidomimetic scaffolds for tuning their biological activity. Further advanced structural modifications on these compounds will become a subject for future investigations in order to find new diagnostic/therapeutic agents for neurodegenerative diseases.

## Experimental Section

### General Information

All reagents were purchased from commercial suppliers and used as received, unless otherwise stated. Molecular sieves were dried at 300 °C under a high vacuum for 4 h prior to use. DMF and THF were dried using an SG Water solvent drying tower, according to the manufacturer’s instructions, and stored over activated 3 Å (DMF) or 4 Å (THF) molecular sieves (5% w/v) for 48 h or more before use. Cs_2_CO_3_ was used as purchased from Sigma-Aldrich (i.e., without further drying). Microwave reactions were performed in sealed vessels using a Biotage Initiator microwave synthesizer, temperatures were monitored by an internal IR probe, and stirring was mediated magnetically. TLC was performed on purchased aluminum backed silica gel plates (median pore size 60 Å, fluorescent indicator 254 nm) and detected with UV light at 254 and 366 nm. Flash column chromatography was performed using silica gel (0.063–0.200 mesh). Automated flash column chromatography was performed using a Biotage Isolera One system and purchased prepacked silica gel cartridges (Biotage SNAP cartridge, KP-Sil or Biotage Sfär Silica D, Duo 60 μm, cartridge). Preparative HPLC was performed on a Gilson instrument with a Phenomenex column (250 × 21.2 mm^2^; Gemini 5 μm NX-C18, 110 Å). MeCN/water, with 0.1% HCOOH, was used as mobile phase. A gradient from 30–100% MeCN in water was run over 30 min with a flow rate of 20 mL/min. The elution was monitored with UV-abs. at 254 nm. Freeze-drying was accomplished by freezing the diluted MeCN/water solutions in liquid nitrogen and then emloying a Scanvac CoolSafe freeze-dryer connected to an Edwards 28 rotary vane oil pump. IR spectra were recorded on a Bruker Alpha-t spectrometer. The samples were prepared as KBr pellets or between NaCl plates; absorbances are given in reciprocal cm. ^1^H and ^13^C NMR spectra were recorded on a Bruker Avance III 400 MHz spectrometer with a BBO-F/H Smartprobe or a Bruker Avance III HD 600 MHz spectrometer with a CP BBO-H/F, 5 mm cryoprobe, at 298 K, unless another temperature is given. All spectrometers were operated by Topspin 3.5.7. Spectra were then processed by MestReNnova v. 10. Resonances are given in ppm relative to TMS and calibrated to solvent residual signals [CDCl_3_: δ_H_ = 7.26 ppm, δ_C_ = 77.16 ppm; (CD_3_)_2_SO: δ_H_ = 2.50 ppm, δ_C_ = 39.51 ppm]. The following abbreviations are used to indicate splitting patterns: s = singlet; d = doublet; dd = double doublet; t = triplet; m = multiplet; bs = broad singlet. LC-MS was conducted on a Micromass ZQ mass spectrometer with ES^+^ and ES^–^ ionization. HRMS was performed on a mass spectrometer with ESI-TOF (ES^+^/ES^–^). Bicyclic 2-pyridones **1a**–**d** and tricyclic 2-pyridones **3a**–**e** were prepared according to the reported procedures.^7,8d,8e^ An Oxford Diffraction Excalibur 3 system was used for X-ray data collection and Crysalis RED data extraction. Crystal Maker 9.2 was used for molecular graphics.

#### General Procedure for Synthesis of **2a**–**g** and **4**

Thiazolino fused 2-pyridone **1** (0.5 mmol, 1.0 equiv) and cesium carbonate (326 mg, 1.0 mmol, 2.0 equiv) were weighed in an oven-dried Biotage Initiator microwave reaction tube (2–5 mL) equipped with a magnetic follower. The tube was sealed with a septum and put under a high vacuum for 30 min at room temperature and then backfilled with nitrogen. Dried THF (1.5 mL) was added with a syringe. The septum was removed briefly to add alkyl halide (3.0–9.0 equiv) with an automatic pipet, and the tube was quickly sealed with a crimp cap. The resulting suspension was stirred in an oil bath at 60 °C until reaction completion was indicated. The reactions were monitored with TLC on samples extracted with syringes. Upon complete consumption of starting material **1**, the reaction mixture was transferred to a separation funnel and partitioned between brine (25 mL) and EtOAc (2 × 25 mL). The organic phases were combined, dried over anhydrous sodium sulfate, filtered, and evaporated. The residue was redissolved in a small amount of DCM and purified with automated flash column chromatography.

#### (*R*)-1-Phenylethyl (*R*)-8-Cyclopropyl-7-(naphthalen-1-ylmethyl)-5-oxo-2,3-dihydro-5*H*-thiazolo[3,2-*a*]pyridine-3-carboxylate (**1m**)

C-10 (200 mg, 0.529 mmol), DMAP (6.47 mg, 0.053 mmol), and DCC (163 mg, 0.794 mmol) were dissolved in DCM (5 mL) at 25 °C, and (*S*)-(−)-1-phenylethanol (100 μL, 0.834 mmol) was added dropwise to the mixture. The reaction mixture was then left stirring at 40 °C overnight. After 24 h, the reaction mixture was diluted with DCM (100 mL), washed with aqueous NH_4_Cl (saturated) followed by washing with brine (150 mL), and dried over anhydrous Na_2_SO_4_, filtered, and concentrated. The crude product was purified by automated flash column chromatography (25 g SNAP cartridge) eluting with 0–40% ethyl acetate in heptane, to provide 170 mg (67%) of **1m** as a white powder. IR (KBr): ν 3450, 1740, 1654, 1579, 1487, 1424, 1285, 1209, 1160, 1090, 1061, 1028, 993, 780, 760 cm^–1^. ^1^H NMR (400 MHz, CDCl_3_) δ 7.79 (dd, *J* = 7.7, 1.7 Hz, 1H), 7.76–7.62 (m, 2H), 7.44–7.29 (m, 3H), 7.24–7.16 (m, 5H), 5.85 (d, *J* = 6.6 Hz, 1H), 5.69 (d, *J* = 7.8 Hz, 1H), 5.50 (dd, *J* = 8.6, 2.3 Hz, 1H), 4.34 (dd, *J* = 40.0, 17.2 Hz, 2H), 3.55 (dd, *J* = 11.7, 8.6 Hz, 1H), 3.33 (dd, *J* = 11.7, 2.3 Hz, 1H), 1.81 (dd, *J* = 12.6, 3.4 Hz, 1H), 1.63–1.52 (m, 2H), 1.50 (t, *J* = 5.9 Hz, 3H), 1.43 (d, *J* = 6.6 Hz, 1H), 1.26–1.20 (m, 1H), 1.06–1.00 (m, 1H), 0.88–0.75 (m, 2H), 0.63 (td, *J* = 6.0, 3.0 Hz, 2H); ^13^C{^1^H} NMR [100 MHz, CDCl_3_] δ 167.6, 161.5, 156.9, 147.2, 140.9, 134.1, 132.1, 129.0, 128.7, 128.6, 128.2, 127.8, 127.6, 126.4, 126.1, 125.8, 125.7, 123.9, 115.5, 113.6, 74.8, 63.1, 36.4, 32.1, 25.7, 22.2, 11.4, 8.0; HRMS (ESI-TOF) *m*/*z* [M + H]^+^ calcd for C_30_H_28_NO_3_S^+^ 482.1790; found 482.1795.

#### Methyl 2-(5-Cyclopropyl-6-(methylthio)-2-oxopyridin-1(2*H*)-yl)acrylate (**2a**)

The compound was prepared from **1a** (118 mg, 0.47 mmol) following the general procedure, using 3.0 equiv of methyl iodide (88 μL, 1.4 mmol). The reaction was complete to TLC analysis after 23 h. The crude product was purified with automated flash column chromatography (10 g Sfär cartridge, 20–80% EtOAc in heptane) to give pure **2a** as an orange solid (109 mg, 0.412 mmol, 88%). IR (KBr): ν 3083, 3001, 2952, 2924, 1734, 1669, 1591, 1497, 1437, 1363, 1328, 1302, 1250, 1199, 1170, 1087, 1038 cm^–1^. ^1^H NMR (400 MHz, CDCl_3_) δ 6.89 (d, *J* = 9.6 Hz, 1H), 6.73 (s, 1H), 6.62 (d, *J* = 9.6 Hz, 1H), 5.83 (s, 1H), 3.82 (s, 3H), 2.37–2.30 (m, 1H), 2.29 (s, 3H), 1.04–0.93 (m, 2H), 0.69–0.60 (m, 2H). ^13^C{^1^H} NMR (100 MHz, CDCl_3_) δ 163.3, 162.1, 140.9, 137.3, 136.1, 126.9, 122.8, 122.0, 52.8, 19.6, 12.4, 7.6, 7.4. HRMS (ESI-TOF) *m*/*z*: [M + H]^+^ calcd for C_13_H_16_NO_3_S^+^ 266.0845; observed 266.0846.

#### Methyl 2-(5-Cyclopropyl-6-(methylthio)-3-nitro-2-oxopyridin-1(2*H*)-yl)acrylate (**2b**)

The compound was prepared from **1b** (148 mg, 0.5 mmol) following the general procedure, using 4.2 equiv of methyl iodide (131 μL, 2.1 mmol). The reaction was complete to TLC analysis after 21 h. The crude product was purified with automated flash column chromatography (10 g Sfär cartridge, 10–50% EtOAc in heptane) to give pure **2b** as a bright yellow solid (75 mg, 0.240 mmol, 48%). IR (KBr): ν 3010, 2955, 1733, 1690, 1639, 1516, 1483, 1438, 1392, 1308, 1235, 1201, 1170, 1093 cm^–1^. ^1^H NMR (400 MHz, CDCl_3_) δ 7.97 (s, 1H), 6.79 (d, *J* = 1.2 Hz, 1H), 5.89 (d, *J* = 1.2 Hz, 1H), 3.84 (s, 3H), 2.44 (s, 3H), 2.30–2.20 (m, 1H), 1.17–1.05 (m, 2H), 0.78–0.70 (m, 2H). ^13^C{^1^H} NMR (100 MHz, CDCl_3_) δ 162.5, 154.0, 152.4, 138.3, 136.9, 135.9, 128.3, 124.0, 53.3, 19.8, 13.0, 8.8, 8.1. HRMS (ESI-TOF) *m*/*z*: [M + H]^+^ calcd for C_13_H_15_N_2_O_5_S^+^ 311.0696; observed 311.0691.

#### Methyl 2-(5-Cyclopropyl-6-(methylthio)-4-(naphthalen-1-ylmethyl)-2-oxopyridin-1(2*H*)-yl)acrylate (**2c**)

The compound was prepared from **1c** (176 mg, 0.45 mmol) following the general procedure, using 9.0 equiv of methyl iodide (252 μL, 4.05 mmol). The reaction was complete to TLC analysis after 21 h. The crude product was purified with automated flash column chromatography (10 g Sfär cartridge, 10–60% EtOAc in heptane) to give pure **2c** as an off white powder (144 mg, 0.355 mmol, 79%). IR (KBr): ν 3044, 2999, 2951, 1734, 1663, 1583, 1510, 1482, 1437, 1404, 1358, 1331, 1281, 1204, 1178, 1134 cm^–1^. ^1^H NMR [400 MHz, (CD_3_)_2_SO] δ 8.04–7.93 (m, 1H), 7.89 (dd, *J* = 9.6, 6.9 Hz, 2H), 7.59–7.46 (m, 3H), 7.42 (d, *J* = 6.9 Hz, 1H), 6.55 (s, 1H), 5.92 (s, 1H), 5.49 (s, 1H), 4.54 (d, *J* = 8.3 Hz, 2H), 3.71 (s, 3H), 2.40 (s, 3H), 1.88–1.77 (m, 1H), 1.23–0.91 (m, 3H), 0.80–0.70 (m, 1H). ^13^C{^1^H} NMR [100 MHz, (CD_3_)_2_SO] δ 162.9, 160.7, 156.4, 144.6, 135.6, 134.2, 133.5, 131.6, 128.7, 128.0, 127.8, 127.5, 126.5, 125.9, 125.8, 124.0, 123.5, 118.1, 79.2, 52.6, 35.6, 19.8, 11.3, 10.8, 8.9. HRMS (ESI-TOF) *m*/*z*: [M + H]^+^ calcd for C_24_H_24_NO_3_S^+^ 406.1471; observed 406.1486.

#### Methyl 2-(6-(Methylthio)-4-(naphthalen-1-ylmethyl)-2-oxo-5-(3-(trifluoromethyl)phenyl)pyridin-1(2*H*)-yl)acrylate (**2d**)

The compound was prepared from **1d** (248 mg, 0.5 mmol) following the general procedure, using 9.0 equiv of methyl iodide (280 μL, 4.5 mmol). The reaction was complete to TLC analysis after 21 h. The crude product was purified with automated flash column chromatography (10 g Sfär cartridge, 10–50% EtOAc in heptane) to give pure **2d** as an off white solid (162 mg, 0.317 mmol, 64%). IR (KBr): ν 3046, 3002, 2953, 1734, 1669, 1587, 1500, 1482, 1438, 1401, 1329, 1288, 1203, 1166, 1126, 1094, 1075, 1059 cm^–1^. ^1^H NMR [400 MHz, (CD_3_)_2_SO, 343 K] δ 7.93–7.86 (m, 1H), 7.81 (d, *J* = 8.2 Hz, 1H), 7.73–7.54 (m, 5H), 7.52–7.40 (m, 3H), 7.24 (dd, *J* = 7.1, 1.2 Hz, 1H), 6.65 (d, *J* = 1.0 Hz, 1H), 6.10 (d, *J* = 1.0 Hz, 1H), 5.99 (s, 1H), 4.00–3.88 (m, 2H) 3.76 (s, 3H), 2.02 (s, 3H). ^13^C{^1^H} NMR [100 MHz, (CD_3_)_2_SO, 343 K] δ 162.7, 160.7, 152.6, 141.8, 137.2, 135.0, 133.1, 133.1, 130.9, 128.9, 128.6, 128.2, 127.5, 127.2, 127.1, 126.3, 125.9, 125.4, 125.1, 124.0, 123.9, 123.1, 119.8, 78.8, 52.3, 36.2, 19.3. ^19^F NMR [376 MHz, (CD_3_)_2_SO, 343 K] δ −61.2. HRMS (ESI-TOF) *m*/*z*: [M + H]^+^ calcd for C_28_H_23_F_3_NO_3_S^+^ 510.1345; observed 510.1359.

#### Methyl 2-(6-(Butylthio)-5-cyclopropyl-2-oxopyridin-1(2*H*)-yl)acrylate (**2e**)

The compound was prepared from **1a** (126 mg, 0.5 mmol) following the general procedure, using 3.0 equiv of butyl iodide (171 μL, 1.5 mmol). The reaction was complete to TLC analysis after 47 h. The crude product was purified with automated flash column chromatography (10 g Sfär cartridge, 10–70% EtOAc in heptane) to give pure **2e** as a yellow solid (93 mg, 0.303 mmol 61%). IR (KBr): ν 2957, 1736, 1671, 1593, 1498, 1438, 1363, 1327, 1303, 1249, 1200, 1171, 1088 cm^–1^. ^1^H NMR (600 MHz, CDCl_3_) δ 6.86 (d, *J* = 9.6 Hz, 1H), 6.73 (s, 1H), 6.62 (d, *J* = 9.6 Hz, 1H), 5.82 (d, *J* = 0.7 Hz, 1H), 3.80 (s, 3H), 2.71 (q, *J* = 7.3 Hz, 2H), 2.35 (tt, *J* = 8.5, 5.2 Hz, 1H), 1.58–1.48 (m, 2H), 1.42–1.34 (m, 2H), 1.01–0.94 (m, 2H), 0.89 (t, *J* = 7.3 Hz, 3H), 0.68–0.61 (m, 2H). ^13^C{^1^H} NMR (151 MHz, CDCl_3_) δ 163.4, 162.4, 140.0, 137.1, 136.3, 127.6, 127.2, 122.0, 52.9, 36.9, 31.3, 22.1, 13.7, 12.8, 7.9, 7.8. HRMS (ESI-TOF) *m*/*z*: [M + H]^+^ calcd for C_16_H_21_NO_3_S 308.1315; observed 308.1328.

#### Methyl 2-(6-(Butylthio)-5-cyclopropyl-4-(naphthalen-1-ylmethyl)-2-oxopyridin-1(2*H*)-yl)acrylate (**2f**)

The compound was prepared from **1c** (196 mg, 0.5 mmol) following the general procedure, using 9.0 equiv of butyl iodide (280 μL, 4.5 mmol). The reaction was complete to TLC analysis after 7 d. The crude product was purified with automated flash column chromatography (25 g Sfär cartridge, 10–40% EtOAc in heptane) to give pure **2f** as a light yellow solid (66 mg, 0.147 mmol, 29%). IR (KBr): ν 3001, 2955, 2931, 2870, 1736, 1665, 1584, 1481, 1435, 1403, 1358, 1331, 1280, 1203, 1178, 1134, 792, 781 cm^–1^. ^1^H NMR (400 MHz, CDCl_3_) δ 7.90–7.84 (m, 1H), 7.84–7.75 (m, 2H), 7.51–7.44 (m, 2H), 7.44–7.38 (m, 1H), 7.26 (d, *J* = 9.0 Hz, 1H), 6.67 (s, 1H), 6.03 (s, 1H), 5.76 (s, 1H), 4.58–4.40 (m, 2H), 3.79 (s, 3H), 2.83 (q, *J* = 7.5 Hz, 2H), 1.62–1.46 (m, 3H), 1.44–1.30 (m, 2H), 1.22–1.12 (m, 1H), 1.11–0.95 (m, 2H), 0.91 (t, *J* = 7.3 Hz, 3H), 0.85–0.74 (m, 1H). ^13^C{^1^H} NMR (100 MHz, CDCl_3_) δ 163.3, 162.2, 155.9, 143.6, 136.3, 134.1, 134.1, 132.1, 129.0, 127.9, 127.6, 127.4, 126.4, 125.9, 125.7, 124.6, 123.8, 120.2, 52.8, 36.9, 36.6, 31.3, 22.0, 13.8, 11.9, 11.5, 9.6. HRMS (ESI-TOF) *m*/*z*: [M + H]^+^ calcd for C_27_H_30_NO_3_S^+^ 448.1941; observed 448.1954.

#### Methyl 2-(6-(Allylthio)-5-cyclopropyl-2-oxopyridin-1(2*H*)-yl)acrylate (**2g**)

The compound was prepared from **1a** (126 mg, 0.5 mmol) following the general procedure using allyl iodide (192 μL, 2.1 mmol). TLC showed completion of reaction in 12 h. The crude product was purified with automated flash column chromatography (50 g Sfär cartridge, 10–80% EtOAc in heptane) to give pure **2g** as a light brown solid (120 mg, 0.4 mmol, 82%). IR (KBr): ν 3435, 3081, 3003, 2953, 1734, 1669, 1592, 1498, 1437, 1326, 1303, 1250, 1200, 1171 cm^–1^. ^1^H NMR (400 MHz, CDCl_3_) δ 6.85 (d, *J* = 9.6 Hz, 1H), 6.75 (d, *J* = 0.7 Hz, 1H), 6.59 (dd, *J* = 9.6, 0.6 Hz, 1H), 5.87–5.70 (m, 2H), 5.11–4.99 (m, 2H), 3.81 (s, 3H), 3.40 (dd, *J* = 12.7, 7.1 Hz, 1H), 3.29 (dd, *J* = 12.7, 7.6 Hz, 1H), 2.40–2.30 (m, 1H), 1.04–0.90 (m, 2H), 0.72–0.52 (m, 2H). ^13^C{^1^H} NMR (100 MHz, CDCl_3_) δ 163.4, 162.1, 138.3, 136.6, 136.1, 132.2, 127.8, 127.3, 122.4, 118.8, 77.3, 77.0, 76.7, 52.8, 39.8, 12.7, 7.8, 7.8. HRMS (ESI-TOF) *m*/*z*: [M + H]^+^ calcd for C_15_H_18_NO_3_S^+^ 292.1002; observed 292.1011.

#### Methyl 2-(5-Cyclopropyl-6-(methylthio)-2-(4-nitrophenyl)-8-oxo-4-phenyl-1,7-naphthyridin-7(8*H*)-yl)acrylate (**4**)

The compound was prepared from **3a** (125 mg, 0.25 mmol) following the general procedure but at 0.25 mmol scale, using 5.0 equiv of methyl iodide (78 μL, 1.25 mmol) and 3.0 equiv of cesium carbonate (244 mg, 0.75 mmol). The reaction was complete to TLC analysis after 21 h. The crude product was purified with automated flash column chromatography (10 g Sfär cartridge, 10–50% EtOAc in heptane) to give pure **4** as a bright yellow powder (74 mg, 0.144 mmol, 58%). IR (KBr): ν 3080, 3003, 2951, 2926, 1734, 1677, 1585, 1520, 1453, 1438, 1410, 1344, 1256, 1210, 1155, 1141, 1108, 911, 853, 735 cm^–1^. ^1^H NMR (400 MHz, CDCl_3_) δ 8.42 (d, *J* = 9.0 Hz, 2H), 8.33 (d, *J* = 9.0 Hz, 2H), 8.00 (s, 1H), 7.65–7.36 (m, 5H), 6.82 (d, *J* = 0.7 Hz, 1H), 6.00 (d, *J* = 0.8 Hz, 1H), 3.84 (s, 3H), 2.42 (s, 3H), 1.27–1.12 (m, 1H), 0.68–0.13 (m, 4H). ^13^C{^1^H} NMR (100 MHz, CDCl_3_) δ 163.6, 160.5, 153.1, 149.4, 148.6, 143.9, 143.8, 143.3, 141.3, 135.7, 133.6, 128.7, 128.3, 128.3, 127.8, 126.6, 124.1, 120.5, 53.0, 20.2, 16.7. HRMS (ESI-TOF) *m*/*z*: [M + H]^+^ calcd for C_28_H_24_N_3_O_5_S^+^ 514.1431; observed 514.1444.

#### General Procedure for Synthesis of **2h** and **5a**–**l**

Thiazolino fused 2-pyridone **1** (0.5 mmol, 1.0 equiv) and cesium carbonate (326 mg, 1.0 mmol, 2.0 equiv) were weighed in an oven-dried 2–5 mL microwave vial and flushed with nitrogen. Dried THF (1.5 mL) and propargyl bromide (225 μL, 2.10 mmol, 4.2 equiv) were added to it, and the resulting mixture was stirred at 60 °C. After 23 h, additional cesium carbonate (163 mg, 0.5 mmol, 1.0 equiv) was added and the mixture was stirred for another 1 h to consume any traces of ring opened intermediate **2**. The mixture was then concentrated on a rotary evaporator and transferred to a separation funnel. DCM (50 mL) was added, and the solution was washed with brine (30 mL). The organic phase was concentrated and purified with automated flash column chromatography using a 100 g SNAP cartridge unless otherwise specified.

#### Bulk Scale Preparation of **5j**

Thiazolino fused 2-pyridone **1j** (1.27 g, 2.57 mmol, 1.0 equiv) and cesium carbonate (1.67 g, 5.14 mmol, 2.0 equiv) were weighed in an oven-dried 10–20 mL microwave vial and flushed with nitrogen. Dried THF (7 mL) and propargyl bromide (1.16 mL, 10.8 mmol, 4.2 equiv) were added to it, and the resulting mixture was stirred at 60 °C. The mixture was then concentrated on a rotary evaporator and transferred to a separation funnel. DCM (50 mL) was added, and the solution was washed with brine (30 mL). The organic phase was concentrated and purified with automated flash column chromatography using a 100 g SNAP cartridge to give 700 mg of **5j** in 51% yield.

#### Methyl 2-(5-Cyclopropyl-2-oxo-6-(prop-2-yn-1-ylthio)pyridin-1(2*H*)-yl)acrylate (**2h**)

The compound was prepared following the general procedure, using propargyl bromide (225 μL, 2.10 mmol, 4.2 equiv). The crude product was purified with automated flash column chromatography (50 g Sfär cartridge, 10–80% EtOAc in heptane) to give pure **2h** as a light brown syrup (30 mg, 0.10 mmol, 20%) and **5a** as a light brown powder (65 mg, 0.22 mmol, 44%). IR (KBr): ν 3437, 3288, 3082, 3002, 2953, 1733, 1666, 1590, 1498, 1438, 1364, 1327, 1303, 1252, 1200, 1171, 1087 cm^–1^. ^1^H NMR (400 MHz, CDCl_3_) δ 6.88 (d, *J* = 9.7 Hz, 1H), 6.76 (s, 1H), 6.67–6.57 (m, 1H), 5.88 (s, 1H), 3.81 (s, 3H), 3.49 (dd, *J* = 16.0, 2.6 Hz, 1H), 3.39 (dd, *J* = 16.0, 2.6 Hz, 1H), 2.45–2.38 (m, 1H), 2.45–2.35 (m, 1H), 1.10–0.90 (m, 2H), 0.68–0.65 (m, 2H). ^13^C{^1^H} NMR (100 MHz, CDCl_3_) δ 163.4, 162.1, 137.2, 136.9, 136.1, 128.9, 127.7, 123.2, 77.8, 77.4, 77.1, 76.8, 73.3, 53.0, 24.8, 12.9, 8.26, 8.20. HRMS (ESI-TOF) *m*/*z*: [M + H]^+^ calcd for C_15_H_16_NO_3_S^+^ 290.0845; observed 290.0848.

#### Methyl 4-Cyclopropyl-2-methylene-7-oxo-2,2a-dihydro-7*H*-cyclobuta[4,5]thiazolo[3,2-*a*]pyridine-8*a*(1*H*)-carboxylate (**5a**)

**5a** was prepared following the procedure described above. Brown powder, 100 mg, 69%. IR (KBr): ν 3437, 3083, 2349, 1744, 1657, 1585, 1498, 1436, 1303 cm^–1^. ^1^H NMR (400 MHz, CDCl_3_) δ 7.10 (d, *J* = 9.2 Hz, 1H), 6.22 (d, *J* = 9.2 Hz, 1H), 5.31–5.24 (m, 1H), 5.20–5.13 (m, 1H), 4.80 (q, *J* = 2.5 Hz, 1H), 4.01 (dt, *J* = 17.3, 2.9 Hz, 1H), 3.11 (dq, *J* = 17.3, 2.5 Hz, 1H), 1.60–1.51 (m, 1H), 0.89–0.80 (m, 2H), 0.62–0.53 (m, 2H). ^13^C NMR (100 MHz, CDCl_3_) δ 168.12, 161.11, 149.29, 145.15, 141.72, 115.25, 114.69, 113.13, 74.33, 53.41, 51.60, 41.17, 12.49, 6.54, 6.17. HRMS (ESI-TOF) *m*/*z*: [M + H]^+^ calcd for C_15_H_16_NO_3_S^+^ 290.0845; observed 290.0846.

#### Methyl 2-(6-(But-2-yn-1-ylthio)-5-cyclopropyl-2-oxopyridin-1(2*H*)-yl)acrylate (**2i**)

The compound was prepared from **1a** (126 mg, 0.5 mmol) following the general procedure using 1-bromo-2-butyne (252 μL, 2.1 mmol). After 23 h, the ring opening reaction was complete. To be consistent with the preparation of **3a** and to check if cycloaddition occurred, additional cesium carbonate (163 mg, 0.5 mmol) was added. TLC showed no further reaction after 1 h. The mixture was then concentrated on a rotary evaporator and transferred to a separation funnel. DCM (50 mL) was added, and the solution was washed with brine (30 mL). The organic layer was concentrated on a rotary evaporator and purified by automated flash column chromatography (50 g Sfär cartridge, 10–80% EtOAc in heptane) to give pure **2i** as a light brown syrup (73 mg, 0.24 mmol, 48%). IR (KBr): ν 3443, 3081, 3001, 2952, 2851, 2234, 1734, 1669, 1592, 1498, 1437, 1363, 1326, 1303, 1250, 1200, 1171, 1086, 1037 cm^–1^. ^1^H NMR [600 MHz, (CD_3_)_2_SO] δ 7.04 (d, *J* = 9.6 Hz, 1H), 6.65 (s, 1H), 6.50 (d, *J* = 9.6 Hz, 1H), 6.04 (s, 1H), 3.73 (s, 3H), 3.59 (dq, *J* = 15.8, 2.2 Hz, 1H), 3.43 (dq, *J* = 15.8, 2.3 Hz, 1H), 2.42–2.37 (m, 1H), 1.76 (t, *J* = 2.6 Hz, 3H), 0.95–0.91 (m, 2H), 0.73–0.61 (m, 2H). ^13^C{^1^H} NMR [151 MHz, (CD_3_)_2_SO] δ 163.1, 160.9, 136.9, 136.8, 135.5, 128.0, 127.7, 122.3, 81.2, 73.6, 52.7, 24.9, 12.5, 7.44, 7.42, 3.2. HRMS (ESI-TOF) *m*/*z*: [M + H]^+^ calcd for C_16_H_18_NO_3_S^+^ 304.1002; observed 304.1002.

#### Methyl 2-Methylene-7-oxo-2,2a-dihydro-7*H*-cyclobuta[4,5]thiazolo[3,2-*a*]pyridine-8*a*(1*H*)-carboxylate (**5b**)

**5b** was prepared following the general procedure described above. Brown syrup, 60 mg, 48%. IR (KBr): ν 1742, 1639, 1570, 1511, 1302, 1221 cm^–1^. ^1^H NMR [600 MHz, (CD_3_)_2_SO] δ 7.43 (dd, *J* = 9.0, 7.2 Hz, 1H), 6.31 (dd, *J* = 7.2, 1.0 Hz, 1H), 6.13 (dd, *J* = 9.0, 1.0 Hz, 1H), 5.34–5.25 (m, 2H), 5.19 (t, *J* = 2.0 Hz, 1H), 3.81–3.77 (m, 1H), 3.69 (s, 3H), 2.94 (dd, *J* = 17.1, 2.5 Hz, 1H). ^13^C{^1^H} NMR [151 MHz, (CD_3_)_2_SO] δ 167.3, 160.4, 150.0, 145.2, 141.8, 114.2, 112.8, 100.4, 72.8, 53.0, 51.1, 39.8, 39.6, 39.5, 39.3, 39.2. HRMS (ESI-TOF) *m*/*z*: [M + H]^+^ calcd for C_12_H_12_NO_3_S^+^ 250.0532; observed 250.0534.

#### Methyl 4-Methoxy-2-methylene-7-oxo-2,2a-dihydro-7*H*-cyclobuta[4,5]thiazolo[3,2-*a*]pyridine-8*a*(1*H*)-carboxylate (**5c**)

**5c** was prepared following the general procedure described above. Brown syrup, 80 mg, 57%. IR (KBr): ν 3436, 2952, 2837, 1744, 1663, 1579, 1503, 1453, 1435, 1411, 1353, 1303, 1268, 1218, 1178, 1150, 1088, 1051 cm^–1^. ^1^H NMR [600 MHz, (CD_3_)_2_SO] δ 7.60 (d, *J* = 9.7 Hz, 1H), 6.13 (d, *J* = 9.7 Hz, 1H), 5.39–5.26 (m, 2H), 5.24–5.15 (m, 1H), 3.77 (dt, *J* = 17.2, 2.8 Hz, 1H), 3.71 (s, 3H), 3.69 (s, 3H), 2.97–2.92 (m, 1H). ^13^C{^1^H} NMR [151 MHz, (CD_3_)_2_SO] δ 167.2, 158.4, 145.2, 137.4, 136.4, 133.6, 114.4, 112.9, 73.5, 58.5, 53.0, 51.8. HRMS (ESI-TOF) *m*/*z*: [M + H]^+^ calcd for C_13_H_14_N_4_OS^+^ 280.0638; observed 280.0640.

#### Methyl 4-Cyclopropyl-6-iodo-2-methylene-7-oxo-2,2a-dihydro-7*H*-cyclobuta[4,5]thiazolo[3,2-*a*]pyridine-8*a*(1*H*)-carboxylate (**5d**)

**5d** was prepared following the general procedure described above. Brown syrup, 120 mg, 58%. IR (KBr): ν 1745, 1649, 1578, 1481, 1433, 1329, 1301, 1244, 1218, 1175, 1147, 1089, 1025 cm^–1^. ^1^H NMR [600 MHz, (CD_3_)_2_SO] δ 7.72 (s, 1H), 5.32 (t, *J* = 2.5 Hz, 2H), 5.24–5.14 (m, 1H), 3.79 (dt, *J* = 17.3, 2.6 Hz, 1H), 3.70 (s, 3H), 1.52–1.50 (m, 1H), 0.89–0.73 (m, 2H), 0.63–0.59 (m, 2H). ^13^C{^1^H} NMR [151 MHz, (CD_3_)_2_SO] δ 167.0, 157.2, 150.4, 148.9, 144.9, 115.3, 113.0, 84.6, 74.2, 53.1, 51.4, 11.9, 6.4, 6.1. HRMS (ESI-TOF) *m*/*z*: [M + H]^+^ calcd for C_15_H_15_INO_3_S^+^ 415.9812; observed 415.9814.

#### Methyl 4-Cyclopropyl-2-methylene-7-oxo-6-(*p*-tolyl)-2,2*a*-dihydro-7*H*-cyclobuta[4,5]thiazolo[3,2-*a*]pyridine-8*a*(1*H*)-carboxylate (**5e**)

Colorless syrup, 146 mg, 77%. IR (KBr): ν 3734, 3087, 3018, 2954, 2917, 1739, 1682, 1639, 1593, 1527, 1507, 1427, 1375, 1341, 1298, 1269, 1242, 1217, 1196, 1183, 1171, 1111, 1089, 1046, 1036, 1023 cm^–1^. ^1^H NMR [600 MHz, (CD_3_)_2_SO] δ 7.57 (d, *J* = 8.0 Hz, 2H), 7.29 (s, 1H), 7.16 (d, *J* = 7.9 Hz, 2H), 5.35–5.34 (m, 2H), 5.20 (s, 1H), 3.83 (dt, *J* = 17.2, 2.6 Hz, 1H), 3.70 (s, 3H), 3.02 (dd, *J* = 17.2, 2.6 Hz, 1H), 2.31 (s, 3H), 1.59–1.56 (m, 1H), 0.88–0.80 (m, 2H), 0.76–0.63 (m, 2H). ^13^C{^1^H} NMR [151 MHz, (CD_3_)_2_SO] δ 167.5, 158.6, 147.3, 145.4, 138.1, 136.4, 132.9, 128.4, 128.0, 124.7, 113.6, 112.8, 73.7, 52.9, 51.2, 40.6, 39.9, 39.8, 39.6, 39.5, 39.3, 39.2, 39.1, 20.7, 12.3, 6.3, 6.1. HRMS (ESI-TOF) *m*/*z*: [M + H]^+^ calcd for C_22_H_22_NO_3_S^+^ 380.1315; observed 380.1319.

#### Methyl 4-Cyclopropyl-6-(4-methoxyphenyl)-2-methylene-7-oxo-2,2a-dihydro-7*H*-cyclobuta[4,5]thiazolo[3,2-*a*]pyridine-8*a*(1*H*)-carboxylate (**5f**)

Colorless syrup, 146 mg, 74%. IR (KBr): ν 2241, 1747, 1691, 1632, 1604, 1585, 1519, 1453, 1385, 1342, 1305, 1290, 1249, 1216, 1179, 1141, 1114, 1089, 1058, 1029 cm^–1^. ^1^H NMR [600 MHz, (CD_3_)_2_SO] δ 7.66–7.59 (m, 2H), 7.27 (s, 1H), 6.97–6.87 (m, 2H), 5.34 (t, *J* = 2.5 Hz, 2H), 5.21 (t, *J* = 2.0 Hz, 1H), 3.83 (dt, *J* = 17.6, 2.7 Hz, 1H), 3.77 (s, 3H), 3.70 (s, 3H), 3.08–2.97 (m, 1H), 1.60–1.55 (m, 1H), 0.88–0.79 (m, 2H), 0.74–0.62 (m, 2H). ^13^C{^1^H} NMR [151 MHz, (CD_3_)_2_SO] δ 167.5, 158.7, 158.5, 146.7, 145.5, 137.5, 129.3, 128.1, 124.5, 113.6, 113.3, 112.8, 73.7, 55.1, 52.9, 51.2, 12.3, 6.3, 6.1. HRMS (ESI-TOF) *m*/*z*: [M + H]^+^ calcd for C_22_H_22_NO_4_S^+^ 396.1263; observed 396.1263.

#### Methyl 4-Cyclopropyl-2-methylene-6-(4-nitrophenyl)-7-oxo-2,2*a*-dihydro-7*H*-cyclobuta[4,5]thiazolo[3,2-*a*]pyridine-8*a*(1*H*)-carboxylate (**5g**)

Colorless syrup, 140 mg, 68%. IR (KBr): ν 3730, 1746, 1641, 1590, 1509, 1434, 1338, 1308, 1265, 1242, 1218, 1172, 1136, 1107, 1089, 1055, 1031 cm^–1^. ^1^H NMR [600 MHz, (CD_3_)_2_SO] δ 8.26–8.16 (m, 2H), 8.10–8.00 (m, 2H), 7.56 (s, 1H), 5.41 (q, *J* = 2.5 Hz, 1H), 5.37 (q, *J* = 2.8 Hz, 1H), 5.22 (s, 1H), 3.85 (dt, *J* = 17.4, 2.8 Hz, 1H), 3.72 (s, 3H), 3.10 (dq, *J* = 17.4, 2.6 Hz, 1H), 1.62–1.59 (m, 1H), 0.88–0.86 (m, 2H), 0.78–0.65 (m, 2H). ^13^C{^1^H} NMR [151 MHz, (CD_3_)_2_SO] δ 167.2, 158.2, 150.9, 145.9, 145.1, 142.7, 140.2, 128.9, 123.0, 121.7, 114.1, 113.1, 73.9, 53.1, 51.3, 12.3, 6.4, 6.2. HRMS (ESI-TOF) *m*/*z*: [M + H]^+^ calcd for C_21_H_19_N_2_O_5_S^+^ 411.1009; observed 411.1013.

#### Methyl 4-Cyclopropyl-2-methylene-7-oxo-6-(thiophen-3-yl)-2,2*a*-dihydro-7*H*-cyclobuta[4,5]thiazolo[3,2-*a*]pyridine-8*a*(1*H*)-carboxylate (**5h**)

Colorless syrup, 120 mg, 65%. IR (KBr): ν 3556, 2933, 1739, 1676, 1636, 1585, 1526, 1502, 1449, 1425, 1400, 1374, 1351, 1305, 1271, 1248, 1231, 1216, 1194, 1165, 1126, 1107, 1084, 1056, 1031 cm^–1^. ^1^H NMR [600 MHz, (CD_3_)_2_SO] δ 8.17 (dd, *J* = 3.1, 1.3 Hz, 1H), 7.66 (dd, *J* = 5.2, 1.3 Hz, 1H), 7.56 (s, 1H), 7.53 (dd, *J* = 5.1, 3.1 Hz, 1H), 5.36–5.34 (m, 2H), 5.22–5.18 (m, 1H), 3.85 (dt, *J* = 17.3, 2.8 Hz, 1H), 3.71 (s, 3H), 3.03 (dq, *J* = 17.2, 2.6 Hz, 1H), 1.61–1.56 (m, 1H), 0.89–0.84 (m, 2H), 0.78–0.63 (m, 2H). ^13^C{^1^H} NMR [151 MHz, (CD_3_)_2_SO] δ 167.4, 158.2, 146.8, 145.3, 136.6, 135.8, 126.9, 125.1, 123.1, 119.9, 113.5, 112.8, 73.7, 53.0, 51.1, 40.6, 40.0, 39.9, 39.8, 39.6, 39.5, 39.3, 39.2, 39.1, 12.4, 6.4, 6.2. HRMS (ESI-TOF) *m*/*z*: [M + H]^+^ calcd for C_19_H_18_FNO_3_S_2_^+^ 372.0723; observed 372.0724.

#### Methyl 4-Cyclopropyl-2-methylene-5-(naphthalen-1-ylmethyl)-7-oxo-2,2*a*-dihydro-7*H*-cyclobuta[4,5]thiazolo[3,2-*a*]pyridine-8*a*(1*H*)-carboxylate (**5i**)

The compound was prepared following the general procedure, but after addition of Cs_2_CO_3_ (325 mg, 1 mmol), the reaction mixture was stirred for 2 h. White powder, 105 mg, 49%. IR (KBr): ν 3087, 3004, 2952, 1745, 1652, 1577, 1488, 1428, 1398, 1363, 1331, 1302, 1290, 1264, 1216, 1160, 1092, 1067, 1030 cm^–1^. ^1^H NMR [600 MHz, (CD_3_)_2_SO] δ 8.00–7.94 (m, 1H), 7.94–7.85 (m, 2H), 7.56–7.47 (m, 3H), 7.41 (dd, *J* = 7.0, 1.2 Hz, 1H), 5.32–5.31 (m, 1H), 5.24–5.22 (m, 1H), 5.20–5.17 (m, 1H) 5.17–5.16 (m, 1H), 4.51–4.42 (m, 2H), 3.73 (dt, *J* = 17.1, 2.9 Hz, 1H), 3.62 (d, *J* = 18.4 Hz, 3H), 2.85 (dq, *J* = 17.1, 2.6 Hz, 1H), 1.80–1.74 (m, 1H), 0.99–0.90 (m, 2H), 0.82–0.69 (m, 2H). ^13^C{^1^H} NMR [151 MHz, (CD_3_)_2_SO] δ 167.5, 159.2, 157.6, 149.6, 145.4, 134.2, 133.4, 131.6, 128.6, 127.8, 127.4, 126.4, 125.8, 125.7, 124.1, 113.4, 112.7, 112.4, 72.4, 52.9, 51.0, 40.4, 40.0, 39.9, 39.8, 39.6, 39.5, 39.3, 39.2, 39.1, 35.2, 10.7, 7.7, 7.4. HRMS (ESI-TOF) *m*/*z*: [M + H]^+^ calcd for C_26_H_24_NO_3_S^+^ 430.1471; observed 430.1467.

#### Methyl 2-Methylene-5-(naphthalen-1-ylmethyl)-7-oxo-4-(3-(trifluoromethyl)phenyl)-2,2*a*-dihydro-7*H*-cyclobuta[4,5]thiazolo[3,2-*a*]pyridine-8*a*(1*H*)-carboxylate (**5j**)

Yellow powder, 105 mg, 49%. IR (KBr): ν 3061, 2953, 1746, 1656, 1579, 1482, 1435, 1330, 1292, 1267, 1218, 1165, 1125, 1094, 1072, 1046, 1018 cm^–1^. ^1^H NMR [600 MHz, (CD_3_)_2_SO] δ 7.93–7.89 (m, 1H), 7.82 (d, *J* = 8.3 Hz, 1H), 7.78–7.76 (m, 2H), 7.72 (dd, *J* = 15.6, 7.3 Hz, 2H), 7.66 (dd, *J* = 14.2, 6.5 Hz, 1H), 7.48 (p, *J* = 6.9 Hz, 2H), 7.43 (dd, *J* = 8.2, 7.0 Hz, 1H), 7.28–7.24 (m, 1H), 5.57 (d, *J* = 13.6 Hz, 1H), 5.32–5.14 (m, 3H), 4.02 (qd, *J* = 17.0, 10.0 Hz, 2H), 3.78 (dt, *J* = 17.2, 2.8 Hz, 1H), 3.70 (s, 3H), 3.05–2.89 (m, 1H). ^13^C{^1^H} NMR [151 MHz, (CD_3_)_2_SO] δ 167.2, 159.3, 154.5, 154.5, 149.8, 145.0, 136.8, 136.7, 134.4, 134.3, 133.6, 133.3, 131.2, 131.2, 130.1, 130.0, 128.5, 127.7, 127.6, 127.4, 126.7, 126.2, 125.7, 125.5, 125.1, 123.7, 123.7, 114.1, 114.0, 113.5, 113.1, 79.1, 78.9, 78.7, 73.5, 73.4, 53.1, 51.2, 40.5, 40.5, 40.0, 39.9, 39.8, 39.6, 39.5, 39.3, 39.2, 39.1, 35.7, 31.2. ^19^F NMR [400 MHz, (CD_3_)_2_SO] δ −61.25. HRMS (ESI-TOF) *m*/*z*: [M + H]^+^ calcd for C_30_H_23_F_3_NO_3_S^+^ 534.1345; observed 534.1348.

#### Methyl 4-(Dimethylamino)-2-methylene-5-((4-methylnaphthalen-1-yl)methyl)-7-oxo-2,2*a*-dihydro-7*H*-cyclobuta[4,5]thiazolo[3,2-*a*]pyridine-8*a*(1*H*)-carboxylate (**5k**)

Colorless powder, 130 mg, 58%. IR (KBr): ν 3442, 2930, 2860, 2828, 2785, 1745, 1653, 1573, 1481, 1435, 1392, 1338, 1298, 1248, 1216, 1171, 1149, 1088, 1063 cm^–1^. ^1^H NMR [400 MHz, (CD_3_)_2_SO] δ 8.10–8.03 (m, 1H), 7.94–7.86 (m, 1H), 7.62–7.52 (m, 2H), 7.39–7.27 (m, 2H), 5.33–5.31 (m, 1H), 5.28–5.21 (m, 2H), 5.19–5.17 (m, 1H), 4.29 (s, 2H), 3.75–3.74 (m, 1H), 3.65 (s, 3H), 2.87–2.85 (m, 1H), 2.75 (s, 5H), 2.68–2.63 (m, 3H). ^13^C{^1^H} NMR [100 MHz, (CD_3_)_2_SO] δ 167.4, 158.8, 157.4, 148.5, 145.4, 133.2, 132.5, 132.4, 131.5, 127.5, 126.2, 126.0, 125.7, 125.4, 124.8, 124.5, 113.3, 112.8, 72.7, 52.9, 51.3, 33.7, 19.0. HRMS (ESI-TOF) *m*/*z*: [M + H]^+^ calcd for C_26_H_27_N_2_O_3_S^+^ 447.1737; observed 447.1747.

#### Methyl 2-Methylene-5-(naphthalen-1-ylmethyl)-7-oxo-2,2*a*-dihydro-7*H*-cyclobuta[4,5]thiazolo[3,2-*a*]pyridine-8*a*(1*H*)-carboxylate (**5l**)

White powder, 81 mg, 41%. IR (KBr): ν 2952, 1743, 1656, 1573, 1506, 1435, 1396, 1306, 1222, 1156, 1090, 1068, 1018 cm^–1^. ^1^H NMR [400 MHz, (CD_3_)_2_SO] δ 8.08–8.03 (m, 1H), 7.95 (dd, *J* = 7.3, 2.1 Hz, 1H), 7.90–7.83 (m, 1H), 7.59–7.46 (m, 4H), 6.23 (d, *J* = 1.4 Hz, 1H), 5.88 (d, *J* = 1.4 Hz, 1H), 5.26 (q, *J* = 2.6 Hz, 2H), 5.14 (q, *J* = 2.0 Hz, 1H), 4.25 (s, 2H), 3.73 (dt, *J* = 17.1, 2.7 Hz, 1H), 3.65 (s, 3H), 2.88 (dq, *J* = 17.2, 2.6 Hz, 1H). ^13^C{^1^H} NMR [100 MHz, (CD_3_)_2_SO] δ 167.3, 160.1, 156.0, 149.3, 145.1, 134.2, 133.5, 131.4, 128.6, 127.8, 127.4, 126.3, 125.8, 125.7, 124.0, 112.8, 112.7, 101.5, 72.4, 52.9, 51.3, 37.4. HRMS (ESI-TOF) *m*/*z*: [M + H]^+^ calcd for C_23_H_20_NO_3_S^+^ 390.1158; observed 390.1157.

#### (*R*)-1-Phenylethyl (8*a*)-4-Cyclopropyl-2-methylene-5-(naphthalen-1-ylmethyl)-7-oxo-2,2a-dihydro-7*H*-cyclobuta[4,5]thiazolo[3,2-*a*]pyridine-8*a*(1*H*)-carboxylate (a Diastereomeric Mixture of **5m**)

Starting from **1m** (170 mg, 0.353 mmol, 1.0 equiv), the product was synthesized following the general procedure and isolated in 33% yield (170 mg). **5m** as white powder. IR (KBr): ν 1740, 1654, 1487, 1285, 1160, 780 cm^–1^; ^1^H NMR (400 MHz, CDCl_3_) δ 7.81–7.76 (m, 2H), 7.74–7.67 (m, 4H), 7.42–7.34 (m, 4H), 7.34–7.30 (m, 2H), 7.29–7.20 (m, 3H), 7.20–7.13 (m, 9H), 5.84 (dq, *J* = 13.0, 6.5 Hz, 2H), 5.66 (s, 1H), 5.62 (s, 1H), 5.23–5.15 (m, 2H), 5.12–5.01 (m, 2H), 4.62–4.51 (m, 2H), 4.42–4.26 (m, 4H), 3.88 (ddt, *J* = 17.2, 8.9, 2.8 Hz, 2H), 3.03 (tdd, *J* = 14.9, 4.9, 2.5 Hz, 2H), 1.62–1.52 (m, 2H), 1.51 (d, *J* = 6.6 Hz, 3H), 1.36 (d, *J* = 6.6 Hz, 3H), 0.93–0.81 (m, 4H), 0.69 (m, *J* = 7.2, 5.5 Hz, 4H); ^13^C{^1^H} NMR [100 MHz, CDCl_3_] δ 167.0, 161.0, 157.3, 149.8, 145.6, 141.4, 140.9, 134.2, 132.1, 129.0, 128.6, 128.0, 127.8, 127.6, 126.3, 126.0, 125.8, 125.7, 123.9, 115.7, 113.3, 112.8, 74.5, 51.4, 40.8, 36.4, 22.2, 11.2, 8.3, 7.9; HRMS (ESI-TOF) *m*/*z* [M + H]^+^ calcd for C_33_H_30_NO_3_S^+^ 520.1946, found 520.1965.

#### General Procedure for Synthesis of **6a**–**c**

Methyl ester **5j**–**l** was dissolved in THF, and LiOH (0.10 M, 4.5 equiv) was added. Upon completion, HCl (1.00 M, 5.0 equiv) was added. The mixture was stirred for 1 min and concentrated on a rotary evaporator. The residue was dissolved in EtOAc (50 mL) and washed with brine (30 mL). The organic phase was concentrated on a rotary evaporator, dissolved in DMSO, filtered, and purified with preparative HPLC.

#### 4-Cyclopropyl-2-methylene-5-(naphthalen-1-ylmethyl)-7-oxo-2,2a-dihydro-7*H*-cyclobuta[4,5]thiazolo[3,2-*a*]pyridine-8*a*(1*H*)-carboxylic Acid (**6a**)

The reaction was performed on 39 mg (0.09 mmol) of **5i** in THF (4 mL) and showed completion in 6 h. White powder, 20 mg, 53%. IR (KBr): ν 3425, 3086, 1944, 1724, 1621, 1539, 1509, 1485, 1416, 1393, 1359, 1294, 1272, 1214, 1180, 1161, 1025 cm^–1^. ^1^H NMR [600 MHz, (CD_3_)_2_SO] δ 7.98–7.96 (m, 1H), 7.93–7.86 (m, 2H), 7.56–7.48 (m, 3H), 7.42–7.36 (m, 1H), 5.29–5.28 (m, 1H), 5.18 (s, 1H), 5.15–5.14 (m, 2H), 4.46 (s, 2H), 3.68 (dt, *J* = 16.9, 2.7 Hz, 1H), 2.82 (dq, *J* = 17.1, 2.5 Hz, 1H), 1.79–1.74 (m, 1H), 1.00–0.87 (m, 2H), 0.78–0.73 (m, 2H). ^13^C{^1^H} NMR [151 MHz, (CD_3_)_2_SO] δ 168.5, 159.3, 157.3, 149.8, 145.9, 134.3, 133.4, 131.6, 128.6, 127.8, 127.3, 126.4, 125.8, 125.7, 124.1, 113.5, 112.3, 112.1, 72.9, 51.0, 35.2, 10.7, 7.6, 7.5. HRMS (ESI-TOF) *m*/*z*: [M + H]^+^ calcd for C_25_H_22_NO_3_S^+^ 416.1315; observed 416.1319.

#### 2-Methylene-5-(naphthalen-1-ylmethyl)-7-oxo-4-(3-(trifluoromethyl)phenyl)-2,2*a*-dihydro-7*H*-cyclobuta[4,5]thiazolo[3,2-*a*]pyridine-8*a*(1*H*)-carboxylic Acid (**6b**)

The reaction was performed on 48 mg (0.09 mmol) of **5j** in THF (4 mL) and showed completion in 6 h. White powder, 10 mg, 21%. IR (KBr): ν 3446, 1733, 1638, 1576, 1483, 1437, 1397, 1377, 1335, 1299, 1269, 1220, 1166, 1125, 1094, 1073, 1046 cm^–1^. ^1^H NMR [600 MHz, (CD_3_)_2_SO] δ 7.91 (dd, *J* = 7.4, 1.9 Hz, 1H), 7.82 (d, *J* = 8.2 Hz, 1H), 7.80–7.74 (m, 2H), 7.74–7.61 (m, 3H), 7.50–7.48 (m, 2H), 7.45–7.42 (m, 1H), 7.26 (d, *J* = 7.0 Hz, 1H), 5.54 (d, *J* = 6.0 Hz, 1H), 5.23 (s, 1H), 5.16 (s, 1H), 5.13 (s, 1H), 4.06–3.95 (m, 2H), 3.73 (dt, *J* = 17.1, 2.9 Hz, 1H), 2.95 (dd, *J* = 16.9, 3.3 Hz, 1H). ^13^C{^1^H} NMR [151 MHz, (CD_3_)_2_SO] δ 168.3, 159.4, 154.1, 150.0, 145.6, 133.7, 133.3, 131.2, 130.0, 128.5, 127.6, 127.6, 127.3, 126.2, 125.7, 125.5, 123.7, 114.1, 113.3, 112.7, 74.0, 51.3, 35.7. ^19^F NMR [400 MHz, (CD_3_)_2_SO] δ −61.24. HRMS (ESI-TOF) *m*/*z*: [M + H]^+^ calcd for C_29_H_21_F_3_NO_3_S^+^ 520.1180; observed 520.1190.

#### 4-(Dimethylamino)-2-methylene-5-((4-methylnaphthalen-1-yl)methyl)-7-oxo-2,2a-dihydro-7*H*-cyclobuta[4,5]thiazolo[3,2-*a*]pyridine-8*a*(1*H*)-carboxylic Acid (**6c**)

The reaction was performed on 20 mg (0.044 mmol) of **5k** in THF (4 mL) and showed completion in 5 h. Colorless powder, 8 mg, 41%. IR (KBr): ν 3437, 2925, 2854, 2828, 2786, 1726, 1622, 1531, 1480, 1412, 1293, 1216 cm^–1^. ^1^H NMR [400 MHz, (CD_3_)_2_SO] δ 8.10–8.01 (m, 1H), 7.93–7.85 (m, 1H), 7.61–7.51 (m, 2H), 7.39–7.24 (m, 2H), 5.28 (t, *J* = 2.7 Hz, 1H), 5.19 (d, *J* = 1.1 Hz, 1H), 5.13 (dd, *J* = 11.6, 2.7 Hz, 2H), 4.28 (s, 2H), 3.72–3.60 (m, 1H), 2.86 (d, *J* = 2.6 Hz, 1H), 2.75 (s, 6H), 2.66 (s, 3H). ^13^C{^1^H} NMR [151 MHz, (CD_3_)_2_SO] δ 168.9, 159.3, 157.4, 149.4, 133.6, 132.9, 132.0, 128.0, 126.7, 126.5, 126.1, 125.5, 125.3, 125.0, 113.8, 112.7, 51.8, 43.2, 42.3, 34.2, 19.5. HRMS (ESI-TOF) *m*/*z*: [M + H]^+^ calcd for C_25_H_25_N_2_O_3_S ^+^ 433.1580; observed 433.1588.

#### Preparation of Imidazolium Carboxylate Salts **8a**–**b**

Caboxylicacid **6a** (8.00 mg, 0.019 mmol, 1.0 equiv) or **6b** (10.0 mg, 0.019 mmol, 1.0 equiv) was dissolved in methanol (5 mL). Imidazole solution (20 mg/mL in methanol, 66 μL, 0.019 mmol, 1.0 equiv) was added. After 24 h of stirring at room temperature, the reaction mixture was concentrated on a rotary evaporator. The residue was dissolved in acetonitrile/water 1:3 (10 mL) and lyophilized.

#### General Procedure for Synthesis of **9a**–**b** and **7**

The methyl ester (≈0.1 mmol, 1.0 equiv) and LiOH (0.6 mmol, 6.0 equiv) were dissolved in THF/H_2_O 3:1 (5 mL) and stirred at room temperature until complete or almost complete hydrolysis of the methyl ester was indicated by TLC analysis. Then, 1 M HCl (0.7 mmol, 7.0 equiv) was added, and the resulting mixture was stirred for 1 min or until no further color change was seen. The mixture was evaporated partially (THF) and partitioned between brine (5 mL) and DCM/MeOH 9:1 (2 × 10 mL). The organic phase was dried, filtered, and evaporated. The residue was dissolved in DMSO (1–2 mL), filtered through a 0.45 μm syringe filter, and purified with preparative reverse phase chromatoghraphy. The fractions containing the pure desired product were combined and concentrated partially and then redissolved by addition of a small amount of MeCN. The solution was diluted by quick addition of water, frozen in liquid nitrogen, and freeze-dried. *Note: The hydrolysis of the ring opened products****2****was slower and lower yielding, and the conversion was much less clean compared to general thiazolino fused 2-pyridones and the ring closed compounds****5****.*

#### 2-(5-Cyclopropyl-6-(methylthio)-4-(naphthalen-1-ylmethyl)-2-oxopyridin-1(2*H*)-yl)acrylic Acid (**9a**)

The compound was prepared from **2c** (52 mg, 0.128 mmol) following the general procedure. The reaction was finished after 2.5 h, and the product was subsequently isolated as a white powder (11 mg, 0.028 mmol, 22%). IR (KBr): ν 3431, 3063, 3005, 2925, 1718, 1647, 1598, 1556, 1481, 1409, 1277, 1194, 1140, 781 cm^–1^. ^1^H NMR [600 MHz, (CD_3_)_2_SO] δ 13.14 (bs, 1H), 8.03–7.94 (m, 1H), 7.89 (t, *J* = 8.4 Hz, 2H), 7.60–7.47 (m, 3H), 7.41 (d, *J* = 6.9 Hz, 1H), 6.46 (s, 1H), 5.79 (s, 1H), 5.47 (s, 1H), 4.61–4.44 (m, 2H), 2.39 (s, 3H), 1.83 (ddd, *J* = 13.9, 8.1, 5.8 Hz, 1H), 1.23–1.13 (m, 1H), 1.13–1.06 (m, 1H), 0.97 (dd, *J* = 9.6, 4.5 Hz, 1H), 0.80–0.70 (m, 1H). ^13^C{^1^H} NMR [151 MHz, (CD_3_)_2_SO] δ 163.8, 160.7, 156.0, 144.8, 134.3, 133.5, 131.6, 128.7, 127.8, 127.4, 126.4, 125.9, 125.7, 124.0, 123.1, 118.1, 35.6, 19.9, 11.3, 10.8, 9.0. HRMS (ESI-TOF) *m*/*z*: [M + H]^+^ calcd for C_23_H_22_NO_3_S^+^ 392.1315; observed 392.1324.

#### 2-(5-Cyclopropyl-6-(methylthio)-2-(4-nitrophenyl)-8-oxo-4-phenyl-1,7-naphthyridin-7(8*H*)-yl)acrylic Acid (**7**)

The compound was prepared from **4** (50 mg, 0.097 mmol) following the general procedure. The reaction was finished after 2.5 h, and the product was subsequently isolated as a yellow powder (14 mg, 0.028 mmol, 29%). IR (KBr): ν 3433, 3081, 3005, 1731, 1649, 1584, 1520, 1455, 1440, 1410, 1345, 1312, 1258, 1165, 1144, 854, 736 cm^–1^. ^1^H NMR [600 MHz, (CD_3_)_2_SO] δ 13.30 (bs, 1H), 8.59 (d, *J* = 8.9 Hz, 2H), 8.46–8.29 (m, 3H), 7.82–7.39 (m, 5H), 6.66 (s, 1H), 6.07 (s, 1H), 2.45 (s, 3H), 1.18 (ddd, *J* = 13.9, 7.6, 6.0 Hz, 1H), 0.52–0.01 (m, 4H). ^13^C{^1^H} NMR [151 MHz, (CD_3_)_2_SO] δ 164.0, 159.3, 151.7, 148.6, 148.0, 144.5, 143.5, 142.4, 140.7, 137.0, 133.3, 129.2, 128.3, 128.2, 128.0, 127.2, 126.4, 124.0, 118.8, 19.7, 16.5, 13.0, 12.1. HRMS (ESI-TOF) *m*/*z*: [M + H]^+^ calcd for C_27_H_22_N_3_O_5_S^+^ 500.1275; observed 500.1286.

#### 2-(6-(Butylthio)-5-cyclopropyl-4-(naphthalen-1-ylmethyl)-2-oxopyridin-1(2*H*)-yl)acrylic Acid (**9b**)

The compound was prepared from **2f** (44 mg, 0.098 mmol) following the general procedure. The reaction was finished after 3 h, and the product was subsequently isolated as a white powder (12 mg, 0.028 mmol, 28%). IR (KBr): ν 3045, 3004, 2958, 2931, 2871, 1720, 1646, 1598, 1480, 1409, 1274, 1194, 1141, 793, 781 cm^–1^. ^1^H NMR [600 MHz, (CD_3_)_2_SO] δ 13.11 (bs, 1H), 8.04–7.93 (m, 1H), 7.89 (dd, *J* = 8.7, 4.2 Hz, 2H), 7.61–7.45 (m, 3H), 7.39 (d, *J* = 7.0 Hz, 1H), 6.45 (s, 1H), 5.75 (s, 1H), 5.58 (s, 1H), 4.53 (s, 2H), 2.87 (q, *J* = 7.3 Hz, 2H), 1.72 (ddd, *J* = 13.8, 8.1, 5.6 Hz, 1H), 1.46 (p, *J* = 7.3 Hz, 2H), 1.33 (ddt, *J* = 13.9, 11.3, 6.7 Hz, 2H), 1.14 (tt, *J* = 8.6, 4.3 Hz, 1H), 1.05 (tt, *J* = 8.4, 4.7 Hz, 1H), 0.92 (dq, *J* = 10.5, 5.4 Hz, 1H), 0.86 (t, *J* = 7.3 Hz, 3H), 0.74 (dq, *J* = 10.1, 5.1 Hz, 1H). ^13^C{^1^H} NMR [151 MHz, (CD_3_)_2_SO] δ 163.7, 160.8, 155.7, 143.5, 134.6, 133.5, 131.5, 128.7, 127.5, 127.4, 126.4, 125.9, 125.7, 123.9, 123.5, 118.2, 99.5, 79.2, 35.8, 35.6, 30.7, 21.2, 13.5, 11.4, 11.0, 9.2. HRMS (ESI-TOF) *m*/*z*: [M + H]^+^ calcd for C_26_H_28_NO_3_S^+^ 434.1784; observed 434.1793.

#### General Procedure for Synthesis of **10a**–**e**

Thiazolino fused 2-pyridone **3a**–**e** (0.25 mmol, 1.0 equiv) and cesium carbonate (0.5 mmol, 2.0 equiv) were weighed together in a 2–5 mL microwave reaction tube and flushed with nitrogen. Dry THF (1.5 mL) and propargyl bromide (1.05 mmol, 4.2 equiv) were added. After 24 h, additional cesium carbonate and propargyl bromide was added, as specified below, and the reaction mixture was left stirring for 1–3 h more until reaction completion. THF was removed on a rotary evaporator, and the remaining mixture was partitioned between DCM (50 mL) and brine (30 mL). The organic phase was filtered, concentrated, and purified with automated flash column chromatography using a 50 g Sfär cartridge. Because of partial transesterification from methyl ester to propargyl ester and difficulty in their separation by column chromatography, the mixture of both esters was proceeded for ester hydrolysis using LiOH.

#### General Procedure for Ester Hydrolysis

The obtained mixture of esters was dissolved in THF (3 mL), and LiOH (0.10 M, 10.0 equiv) was added. Upon completion, HCl (1.00 M, 11.0 equiv) was added. The mixture was stirred for 1 min and concentrated on a rotary evaporator. The residue was dissolved in EtOAc (50 mL) and washed with brine (30 mL). The organic phase was concentrated on a rotary evaporator, dissolved in DMSO, filtered, and purified with preparative HPLC. **10a** and **10c** were instead purified with normal phase chromatography using 5–30% MeOH in DCM. The yields are reported as overall yields for the two steps.

#### 5-Cyclopropyl-7-methylene-2-(4-nitrophenyl)-10-oxo-4-phenyl-7,8-dihydro-10*H*-cyclobuta[4,5]thiazolo[2,3-*g*][1,7]naphthyridine-8*a*(6*aH*)-carboxylic Acid (**10a**)

After 24 h, Cs_2_CO_3_ (81 mg, 0.25 mmol, 1.0 equiv) and propargyl bromide (56 μL, 0.50 mmol, 2.0 equiv) were added and the mixture was stirred for 1 h more; the reaction completed in 25 h in total. Reaction residue was purified by automated flash column chromatography (50 g Sfär cartridge, 10–80% EtOAc in heptane) to give the mixture of esters as a yellow syrup which was subjected to ester hydrolysis by LiOH in 4 h, as described above. Yellow powder, 20 mg, 26%. IR (KBr): ν 3725, 2997, 1918, 1584, 1549, 1520, 1458, 1376, 1344, 1281, 1197, 1109, 1034 cm^–1^. ^1^H NMR [600 MHz, (CD_3_)_2_SO] δ 8.58–8.52 (m, 2H), 8.39–8.33 (m, 2H), 8.26 (s, 1H), 7.62–7.56 (m, 2H), 7.51–7.42 (m, 3H), 5.30 (s, 1H), 5.18 (s, 2H), 3.84 (dt, *J* = 17.1, 2.6 Hz, 1H), 3.12 (dq, *J* = 17.3, 2.6 Hz, 1H), 1.16–1.12 (m, 1H), 0.16–0.14 (m, 4H). ^13^C{^1^H} NMR [151 MHz, (CD_3_)_2_SO] δ 168.6, 157.6, 150.0, 147.8, 147.3, 143.5, 140.8, 140.5, 133.3, 129.3, 127.9, 127.9, 127.7, 126.6, 123.9, 112.1, 107.0, 51.0, 15.7, 10.9, 10.8. HRMS (ESI-TOF) *m*/*z*: [M + H]^+^ calcd for C_29_H_22_N_3_O_5_S^+^ 524.1275; observed 524.1274.

#### 5-Cyclopropyl-7-methylene-10-oxo-4-phenyl-2-(*p*-tolyl)-7,8-dihydro-10*H*-cyclobuta[4,5]thiazolo[2,3-*g*][1,7]naphthyridine-8*a*(6*aH*)-carboxylic Acid (**10b**)

After 24 h, Cs_2_CO_3_ (162 mg, 0.5 mmol, 2.0 equiv) and propargyl bromide (113 μL, 1.0 mmol, 4.0 equiv) were added and the mixture was stirred for 3 h more; the reaction completed in 27 h in total. The crude product was purified by automated flash column chromatography (50 g Sfär cartridge, 10–80% EtOAc in heptane) to give a mixture of esters as a yellow syrup which was subjected to ester hydrolysis by LiOH in 7 h, as described above. Light yellow powder, 20 mg, 16%. IR (KBr): ν 3358, 1728, 1644, 1588, 1554, 1505, 1488, 1459, 1441, 1376, 1278, 1225, 1185, 1139, 1032 cm^–1^. ^1^H NMR [600 MHz, (CD_3_)_2_SO] δ 8.16 (s, 1H), 8.15 (s, 1H), 8.03 (s, 1H), 7.55 (s, 1H), 7.54 (s, 1H), 7.47–7.43 (m, 4H), 7.33 (s, 1H), 7.32 (s, 1H), 5.30 (s, 1H), 5.22–5.13 (m, 2H), 3.83 (dt, *J* = 17.0, 2.7 Hz, 1H), 3.10 (dt, *J* = 17.0, 2.7 Hz, 1H), 2.37 (s, 4H), 1.14–1.10 (m, 1H), 0.16–0.11 (m, 4H). ^13^C{^1^H} NMR [151 MHz, (CD_3_)_2_SO] δ 168.8, 157.8, 152.7, 147.0, 146.8, 145.7, 141.1, 140.3, 139.1, 134.8, 132.3, 129.4, 127.8, 127.7, 126.7, 125.5, 112.2, 107.2, 73.3, 51.0, 20.8, 15.8, 10.8, 10.7. HRMS (ESI-TOF) *m*/*z*: [M + H]^+^ calcd for C_30_H_25_N_2_O_3_S^+^ 493.1580; observed 493.1582.

#### 2-(4-Fluorophenyl)-7-methylene-10-oxo-5-phenyl-7,8-dihydro-10*H*-cyclobuta[4,5]thiazolo[2,3-*g*][1,7]naphthyridine-8*a*(6*aH*)-carboxylic Acid (**10c**)

After 24 h, Cs_2_CO_3_ (162 mg, 0.5 mmol, 2.0 equiv) and propargyl bromide (113 μL, 1.0 mmol, 4.0 equiv) were added and the reaction mixture was stirred for 3 h more; the reaction completed in 27 h in total. The crude product was purified by automated flash column chromatography (50 g Sfär cartridge, 10–80% EtOAc in heptane) to give a mixture of esters as a yellow syrup which was subjected to ester hydrolysis by LiOH in 8.5 h, as described above. Light yellow powder, 18 mg, 24%. IR (KBr): ν 3741, 3508, 1740, 1614, 1585, 1563, 1526, 1478, 1382, 1351, 1262, 1226, 1197, 1186, 1171 cm^–1^. ^1^H NMR [400 MHz, (CD_3_)_2_SO] δ 8.32 (dd, *J* = 8.7, 5.6 Hz, 2H), 8.08 (s, 1H), 7.57–7.55 (m, 2H), 7.48–7.41 (m, 3H), 7.35 (t, *J* = 8.7 Hz, 2H), 5.31 (s, 1H), 5.19 (q, *J* = 2.8 Hz, 2H), 3.83 (dt, *J* = 17.1, 2.8 Hz, 1H), 3.10 (dt, *J* = 17.2, 2.6 Hz, 1H), 1.18–1.09 (m, 1H), 0.16–0.09 (m, 4H). ^13^C{^1^H} NMR [100 MHz, (CD_3_)_2_SO] δ 168.8, 164.3, 161.8, 157.8, 151.7, 147.2, 147.1, 145.6, 141.0, 140.3, 134.1, 134.0, 132.4, 129.3, 129.1, 129.0, 127.8, 127.7, 125.7, 115.7, 115.5, 112.2, 107.1, 99.5, 73.3, 51.0, 15.8, 10.8, 10.7. ^19^F NMR [400 MHz, (CD_3_)_2_SO] δ −112.37. HRMS (ESI-TOF) *m*/*z*: [M + H]^+^ calcd for C_29_H_22_FN_2_O_3_S^+^ 497.1330; observed 497.1326.

#### 5-Cyclopropyl-7-methylene-2-(4-nitrophenyl)-10-oxo-7,8-dihydro-10*H*-cyclobuta[4,5]thiazolo[2,3-*g*][1,7]naphthyridine-8*a*(6*aH*)-carboxylic Acid (**10d**)

After 24 h, Cs_2_CO_3_ (162 mg, 0.5 mmol, 2.0 equiv) and propargyl bromide (113 μL, 1.0 mmol, 4.0 equiv) were added and the mixture was stirred for 24 h more; the reaction completed in 48 h in total. After purification, the mixture of esters was subjected to ester hydrolysis for 8 h as described above. Yellow powder, 15 mg, 13%. IR (KBr): ν 3749, 3522, 2897, 1737, 1631, 1589, 1572, 1524, 1474, 1412, 1377, 1338, 1280, 1223, 1186, 1157, 1107, 1061, 1035 cm^–1^. ^1^H NMR [600 MHz, (CD_3_)_2_SO] δ 8.54–8.46 (m, 5H), 8.41–8.37 (m, 3H), 5.31 (q, *J* = 2.8 Hz, 1H), 5.24 (q, *J* = 2.5 Hz, 1H), 5.17 (q, *J* = 2.6 Hz, 1H), 3.82 (dt, *J* = 17.1, 2.8 Hz, 1H), 3.07 (tt, *J* = 17.0, 2.6 Hz, 1H), 1.87–1.82 (m, 1H), 1.14–1.07 (m, 2H), 0.69–0.57 (m, 2H). ^13^C{^1^H} NMR [151 MHz, (CD_3_)_2_SO] δ 168.7, 157.9, 151.0, 147.8, 143.7, 139.1, 134.8, 133.0, 127.7, 127.7, 124.3, 124.1, 124.0, 112.3, 106.7, 73.0, 51.2, 9.7, 7.5, 7.3. HRMS (ESI-TOF) *m*/*z*: [M + H]^+^ calcd for C_23_H_18_N_3_O_5_S^+^ 448.0962; observed 448.0968.

#### 5-Cyclopropyl-7-methylene-10-oxo-2-(*p*-tolyl)-7,8-dihydro-10*H*-cyclobuta[4,5]thiazolo[2,3-*g*][1,7]naphthyridine-8*a*(6*aH*)-carboxylic Acid (**10e**)

After 24 h, Cs_2_CO_3_ (162 mg, 0.5 mmol, 2.0 equiv) and propargyl bromide (113 μL, 1.0 mmol, 4.0 equiv) were added and the mixture was stirred for 1 h more; the reaction completed in 25 h in total. The crude mixture was purified by automated flash column chromatography (50 g Sfär cartridge, 10–80% EtOAc in heptane) to give a mixture of esters as a yellow syrup, which was subjected to ester hydrolysis by LiOH in 6.5 h, as described above. Light yellow powder, 16 mg, 15%. IR (KBr): ν 3876, 3520, 2998, 1784, 1726, 1587, 1533, 1514, 1490, 1460, 1441, 1410, 1377, 1277, 1227, 1158, 1075, 1034 cm^–1^. ^1^H NMR [600 MHz, (CD_3_)_2_SO] δ 8.16 (s, 1H), 8.15 (s, 1H), 8.03 (s, 1H), 7.55 (s, 1H), 7.54 (s, 1H), 7.47–7.43 (m, 4H), 7.33 (s, 1H), 7.32 (s, 1H), 5.30 (s, 1H), 5.22–5.13 (m, 2H), 3.83 (dt, *J* = 17.0, 2.7 Hz, 1H), 3.10 (dt, *J* = 17.0, 2.7 Hz, 1H), 2.37 (s, 4H), 1.14–1.10 (m, 1H), 0.16–0.11 (m, 4H). ^13^C{^1^H} NMR [151 MHz, (CD_3_)_2_SO] δ 168.8, 157.8, 152.7, 147.0, 146.8, 145.7, 141.1, 140.3, 139.1, 134.8, 132.3, 129.4, 127.8, 127.7, 126.7, 125.5, 112.2, 107.2, 73.3, 51.0, 20.8, 15.8, 10.8, 10.7. HRMS (ESI-TOF) *m*/*z*: [M + H]^+^ calcd for C_24_H_21_N_2_O_3_S^+^ 417.1267; observed 417.1266.

## Supporting Information Available

Experimental procedures and spectra for characterization of compounds synthesized (PDF)

## Accession Codes

CCDC 2087355 contains the supplementary crystallographic data for this paper. These data can be obtained free of charge via www.ccdc.cam.ac.uk/data_request/cif, or by emailing data_request@ccdc.cam.ac.uk, or by contacting The Cambridge Crystallographic Data Centre, 12 Union Road, Cambridge CB2 1EZ, UK; fax: +44 1223 336033.
