# Supplementary material for: Tandem Ring Opening/Intramolecular [2 + 2] Cycloaddition Reaction for the Synthesis of Cyclobutane Fused Thiazolino-2-Pyridones
Source: J Org Chem. 2021 Nov 12;86(23):16582–92. doi: 10.1021/acs.joc.1c01875 (PMC8650012; doi:10.1021/acs.joc.1c01875)
Supplement: Supplementary file 1 — jo1c01875_si_001.pdf [file jo1c01875_si_001.pdf]

Tandem Ring Opening/Intramolecular [2+2] Cycloaddition Reaction for the Synthesis of Cyclobutane Fused Thiazolino-2-Pyridones

Mohit Tyagi,[a,ϕ] Dan E. Adolfsson,[a,ϕ] Pardeep Singh,[a,ϕ] Jörgen Ådén,[a] Sanduni Wasana Jayaweera,[b] Anna Gharibyan,[b] Jaideep B. Bharate,[a] Anita Kiss,[a] Souvik Sarkar,[a] Anders Olofsson[b] and Fredrik Almqvist[a,*]

[a]Department of Chemistry, Umeå University, 90187 Umeå, Sweden. [b]Department of Medical Biochemistry and Biophysics, Umeå University, 90187 Umeå, Sweden

Index

Optimization of ring opening reaction (**Scheme S1**) S2

Preparation of ring opened compounds **2a–g** and **4** (**Scheme S2**) S2

Reaction of **1a** with propargyl bromide in the presence of Na_2CO_3 and DIPEA (**Scheme S3**) S2

Reaction of thiazolino-fused 2-pyridone **1a** with 1-bromo-2-butyne (**Scheme S4**) S3

Tandem Ring Opening/Intramolecular [2+2] Cycloaddition in presence of chiral auxiliary (**Scheme S5**) S3

Separation of enantiomers (**Scheme S6** and **Scheme S7**) S4

Fibril modulation assays S9

Biological evaluation of enantiomers S10

TEM images of human wild-type α-synuclein fibrils formed in the presence of **10a** or **10d** S13

TEM images of human wild-type α-synuclein fibrils S14

Experimental details for crystallography S15

Copies of ^{1}H, ^{13}C and ^{19}F NMR spectra S17

References S65

Optimization of ring opening reaction:

We commenced our investigation with treatment of thiazolino fused 2-pyridone **1a** with methyl iodide and K_2CO_3. The reaction proceeded slowly and cleanly at rt. and led to isolation of **2a** in 26% yield (along with 69% of unreacted **1a**) in 10 days. Elevation of reaction temp. was considered first for speeding up the transformation, therefore a solution of **1a** in DMF was allowed to react with methyl iodide in presence of various bases at 60 °C in a sealed tube. Increasing the temperature from room temperature to 60 °C did speed up the reaction, but it was still incomplete after 5 days (entry 1). The organic bases DMAP and DBU did not afford more than trace amounts of **2a**. Replacing K_2CO_3 with Cs_2CO_3 however, provided full, clean conversion in just 24 hours. We also observed that we could replace DMF with THF without penalties, allowing for a simpler workup procedure, and the amount of MeI could be reduced to 3.0 equivalents for **1a**.

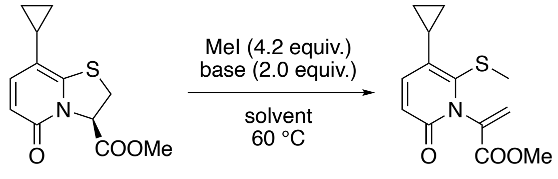

1a **2a**

Entry	Base	Solvent	Time	Results
1	K_2CO_3	DMF	5 d	Incomplete
2	DMAP	DMF	5 d	Traces of product
3	DBU	DMF	5 d	Traces of product
4	Cs_2CO_3	DMF	1 d	Complete
5	Cs_2CO_3	THF	1 d	Complete

Scheme S1. Screening of reaction conditions for ring opening of **1a** at 0.1 mmol scale at a concentration of 0.3 M in dry THF.

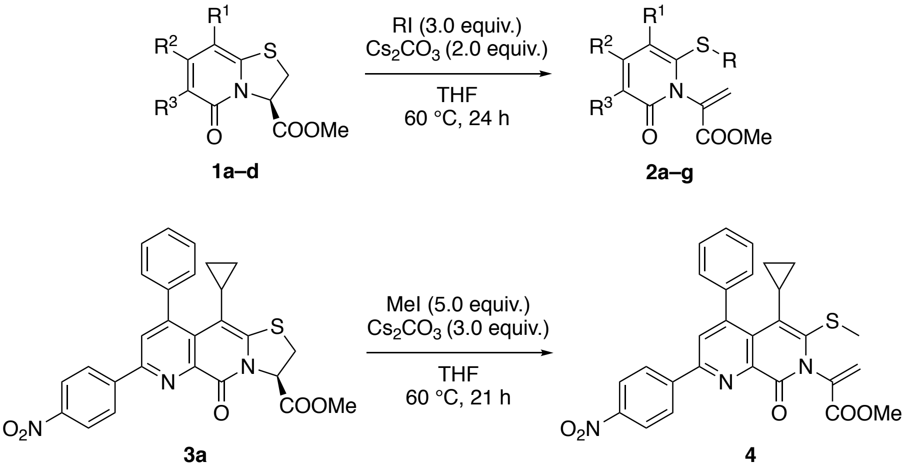

Scheme S2. Preparation of ring opened compounds **2a–g** and **4**.

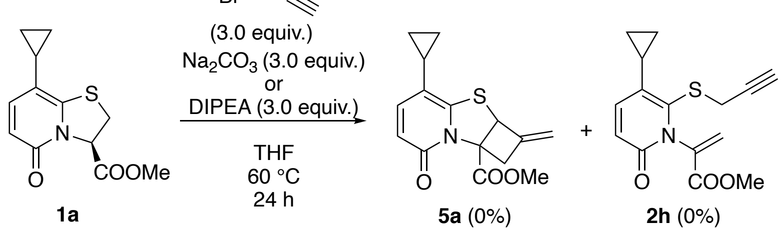

Scheme S3: Reaction of thiazolino-fused 2-pyridone **1a** with propargyl bromide in the presence of Na_2CO_3 and DIPEA.

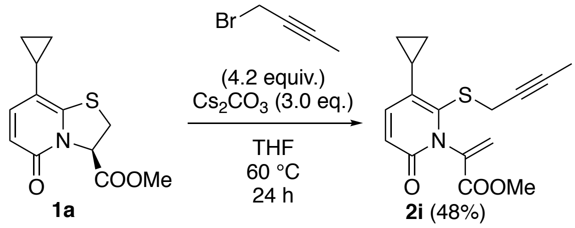

Scheme S4: Reaction of thiazolino-fused 2-pyridone **1a** with 1-bromo-2-butyne

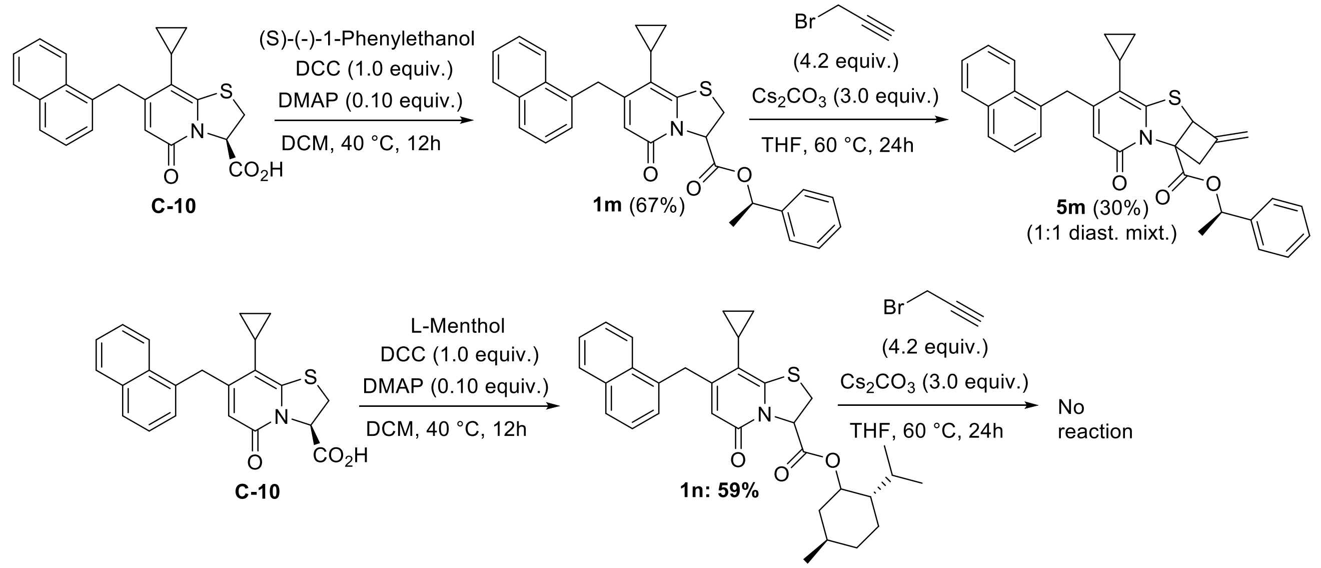

Scheme S5: Tandem Ring Opening/Intramolecular [2+2] Cycloaddition in presence of chiral auxiliary

*(2S,5R)-2-isopropyl-5-methylcyclohexyl (3S)-8-cyclopropyl-7-(naphthalen-1-ylmethyl)-5-oxo-2,3-dihydro-5H-thiazolo[3,2-a]pyridine-3-carboxylate **(1n):*** C-10 (200 mg, 0.529 mmol), DMAP (6.47 mg, 0.053 mmol) and DCC (163 mg, 0.794 mmol) were dissolved in DCM (5 mL) at 25 °C and L-menthol (124 mg, 0.794 mmol) was added to the mixture. The reaction mixture was then left on stirring at 40 °C overnight. After 24 h, reaction mixture was diluted with DCM (100 mL) washed with aqueous NH_4Cl (saturated) followed by washing with brine (150 mL), and dried over anhydrous Na_2SO_4, filtered, and concentrated. The crude product was purified by automated flash column chromatography (25 g SNAP Cartridge) eluting with 0–40% ethyl acetate in heptane, to provide 170 mg (67%) of **1n**, as a white powder. The product was purified by automated flash column chromatography (25 g SNAP Cartridge) eluting with 0–40% ethyl acetate in heptane, and 200 mg of **C-10** was converted to 160 mg (59%) of **1n**, isolated as a white solid; IR (KBr): *ν* 3447, 1742, 1660, 1575, 1497, 1214, 1201, 1170 cm^{-1}; ^{1}H NMR (400 MHz, $CDCl_3$) δ 7.80–7.68 (m, 3H), 7.40–7.30 (m, 3H), 7.18 (dd, J = 6.3, 4.5 Hz, 1H), 5.67 (d, J = 1.0 Hz, 1H), 5.47 (ddd, J = 10.9, 8.3, 2.1 Hz, 1H), 4.67–4.56 (m, 1H), 4.40–4.25 (m, 2H), 3.56–3.35 (m, 2H), 1.94 (d, J = 11.9 Hz, 1H), 1.61–1.53 (m, 3H), 1.42–1.33 (m, 1H), 1.28–1.15 (m, 3H), 0.84–0.74 (m, 10H), 0.65 (t, J = 6.7 Hz, 5H); ^{13}C NMR (100 MHz, $CDCl_3$) δ 168.0, 161.4, 156.6, 156.4, 147.1, 134.2, 134.1, 134.0, 132.1, 129.0, 127.7, 127.5, 126.3, 125.9, 125.6, 123.9, 115.5, 113.4, 63.3, 47.0, 40.5, 36.3, 34.2, 31.8, 26.2, 23.4, 22.0, 20.9, 16.3, 14.2, 11.2, 8.0, 7.8, 7.2; HRMS (ESI-TOF) *m/z* [M+H]$^+$ Calcd for $C_{32}H_{38}NO_3S^+$516.2572, Found 516.2588.

Separation of Enantiomers

Separation of enantiomers of 6b

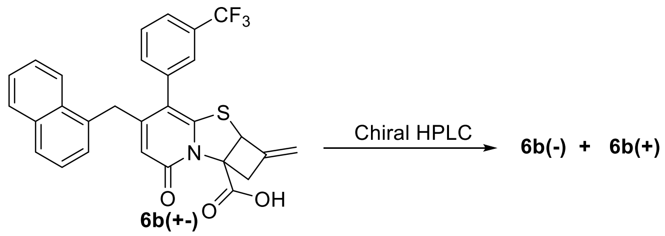

Scheme S6: Separation of enantiomers of **6b** using chiral HPLC

After establishing the method, semipreparative chiral HPLC was used to separate the enantiomers of **6b**. 40 mg of compound **6b** was dissolved in 2.0 ml of DMSO and injected in four iterations. MeCN/H_2O with 0.15% TFA was used as mobile phase. An isocratic gradient of 55% MeCN (0.15% TFA) and 45% H_2O (0.15% TFA with was run for 65 min. at a constant flow rate of 18 ml/min. The first peak eluted after 45 min. and followed by the second peak (**Figure S1).** Fractions were collected manually, concentrated under vacuo and freeze dried. Four separate injections of racemic **6b** were performed in order to get enough material for biological testing. NMR confirmed that the first peak was a pure enantiomer (also confirmed by analytical HPLC, (**Figure S3**) and the second peak was 92% pure enantiomer (**Figure S4**).

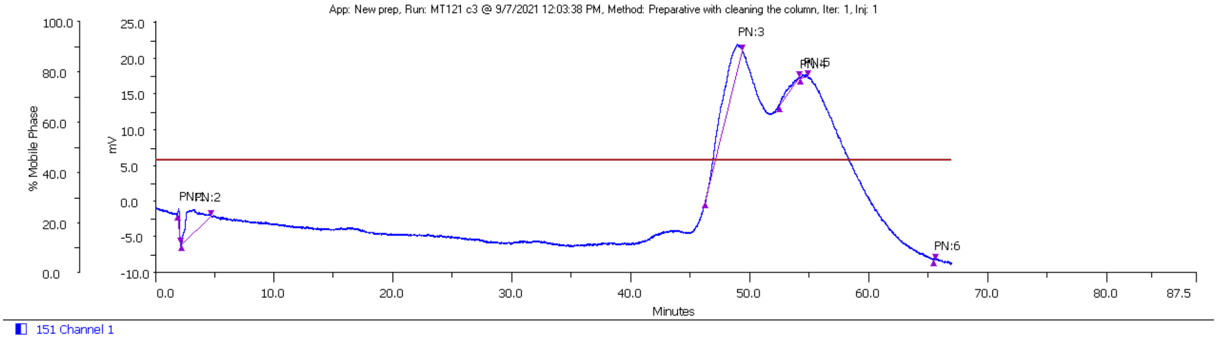

Figure S1. The high-performance liquid chromatography (HPLC) of injected racemic **6b**, with Lux 5 µm i-Amylose-1 (150 x 21.2 mm) chiral column, MeCN/H_2O (0.15% TFA) eluting-solvent system, 18 mL/min flow rate and 254 nm detection wavelength at ambient temperature.

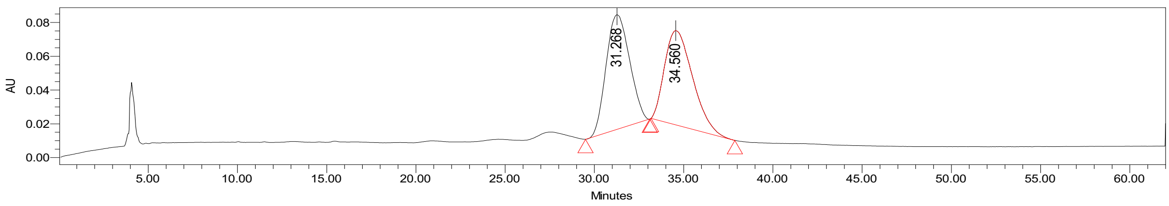

	Name	Retention Time	Area	% Area	Height	Int Type	Amount	Units	Peak Type	Peak Codes
1		31.268	6216276	50.44	67818	bb			Unknown	
2		34.560	6106821	49.56	55804	bb			Unknown	

Figure S2. The high-performance liquid chromatography (HPLC) of injected racemic **6b** (10 µL, 1 mg/mL in DMSO), with Lux 5 µm i-Amylose-1 (250 x 4.6 mm) chiral column, isocratic 55% MeCN+ 45% H_2O (with 0.15% TFA) eluting-solvent system, 0.8 mL/min flow rate and 254 nm detection wavelength at ambient temperature.

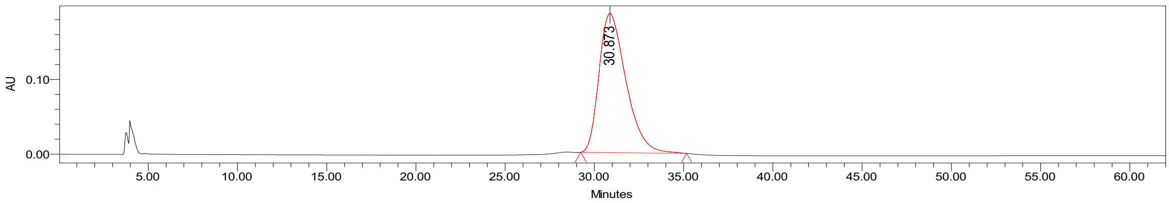

	Name	Retention Time	Area	% Area	Height	Int Type	Amount	Units	Peak Type	Peak Codes
1		30.873	19513782	100.00	186678	bb			Unknown	

Figure S3. The high-performance liquid chromatography (HPLC) of injected pure enantiomer of **6b (-)** (10 µL, 1 mg/mL in DMSO), with Lux 5 µm i-Amylose-1 (250 x 4.6 mm) chiral column, isocratic 55% MeCN+ 45% H_2O (with 0.15% TFA) eluting-solvent system, 0.8 mL/min flow rate and 254 nm detection wavelength at ambient temperature.

6b (-): $[\alpha]_D^{25}$ –248 (*c* 0.092, DMSO); ^{1}H NMR (400 MHz, $(CD_3)_2SO$) δ 13.53 (s, 1H), 7.90 (d, *J* = 6.9 Hz, 1H), 7.89–7.49 (m, 6H), 7.44 (dd, *J* = 17.3, 9.8 Hz, 3H), 7.26 (d, *J* = 6.7 Hz, 1H), 5.54 (s, 1H), 5.19 (d, *J* = 33.9 Hz, 3H), 4.21–3.88 (m, 2H), 3.73 (d, *J* = 17.0 Hz, 1H), 2.95 (d, *J* = 17.0 Hz, 1H); ^{13}C NMR (100 MHz, $(CD_3)_2SO$) δ 168.3, 159.3, 154.1, 149.9, 145.5, 136.8, 134.4, 134.0, 133.7, 133.2, 131.2, 130.0, 128.5, 127.6, 126.7, 126.2, 125.7, 125.4, 125.0, 123.7, 122.5, 114.0, 113.3, 112.7, 73.9, 51.2, 35.7; HRMS (ESITOF) *m/z:* $[M+H]^+$ Calcd for $C_{29}H_{21}F_3NO_3S^+$ 520.1194; observed 520.1217.

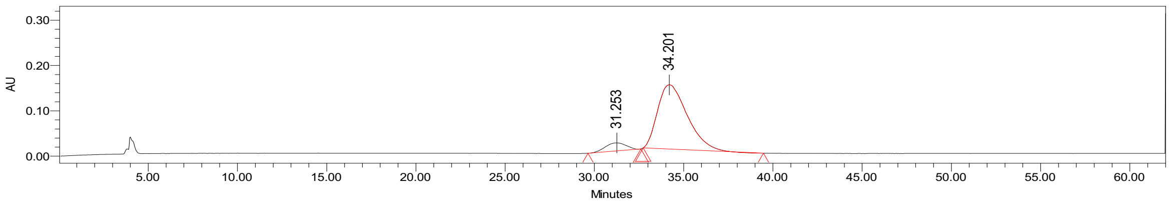

	Name	Retention Time	Area	% Area	Height	Int Type	Amount	Units	Peak Type	Peak Codes
1		31.253	1442392	8.03	17843	bb			Unknown	
2		34.201	16513888	91.97	141734	bb			Unknown	

Figure S4. The high-performance liquid chromatography (HPLC) of injected pure enantiomer of **6b (+)** (10 µL, 2 mg/mL in DMSO), with Lux 5 µm i-Amylose-1 (250 x 4.6 mm) chiral column, isocratic 55% MeCN+ 45% H_2O (with 0.15% TFA) eluting-solvent system, 0.8 mL/min and 254 nm detection wavelength at ambient temperature.

6b (+): $[\alpha]_D^{25}$ +213 (*c* 0.15, DMSO); ^{1}H NMR (400 MHz, $(CD_3)_2SO$) δ 13.55 (s, 1H), 7.98–7.86 (m, 1H), 7.90–7.50 (m, 6H), 7.62–7.29 (m, 3H), 7.26 (d, *J* = 6.9 Hz, 1H), 5.54 (s, 1H), 5.41–4.87 (m, 3H), 4.01 (dd, *J* = 11.8, 4.7 Hz, 2H), 3.74 (d, *J* = 17.1 Hz, 1H), 2.96 (d, *J* = 17.0 Hz, 1H); ^{13}C NMR (100 MHz, $(CD_3)_2SO$) δ 168.4, 159.4, 154.2, 150.0, 145.6, 136.9, 134.5, 134.4, 133.8, 133.3, 131.3, 130.0, 128.6, 127.7, 127.4, 126.3, 125.7, 125.5, 125.3, 123.8, 122.8, 114.1, 113.4, 112.8, 74.0, 51.3, 35.8; HRMS (ESITOF) *m/z:* $[M+H]^+$ Calcd for $C_{29}H_{21}F_3NO_3S^+$ 520.1194; observed 520.1209.

Separation of enantiomers of 10b

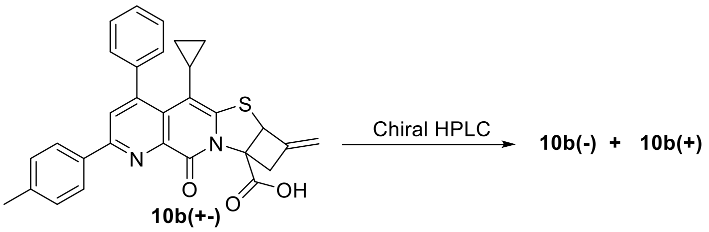

Scheme S7: Separation of enantiomers of **10b** using chiral HPLC

After establishing the method, semipreparative chiral HPLC was used to separate the enantiomers of **10b**. 40 mg of compound **10b** was dissolved in 2.0 ml of DMSO and injected in four iterations. MeCN/H_2O with 0.15% TFA was used as mobile phase. An isocratic gradient of 55% MeCN (0.15% TFA) and 45% H_2O (0.15% TFA with was run for 65 min. at a constant flow rate of 18 ml/min. The first peak eluted after 20 min. and followed by the second peak (**Figure S5**). Fractions were collected manually, concentrated under vacuo and freeze dried. Four separate injections of racemic **10b** were performed in order to get enough material for biological testing. NMR confirmed that the first peak was a pure enantiomer (also confirmed by analytical HPLC, (**Figure S7**). and the second peak was 95% pure enantiomer (**Figure S8**).

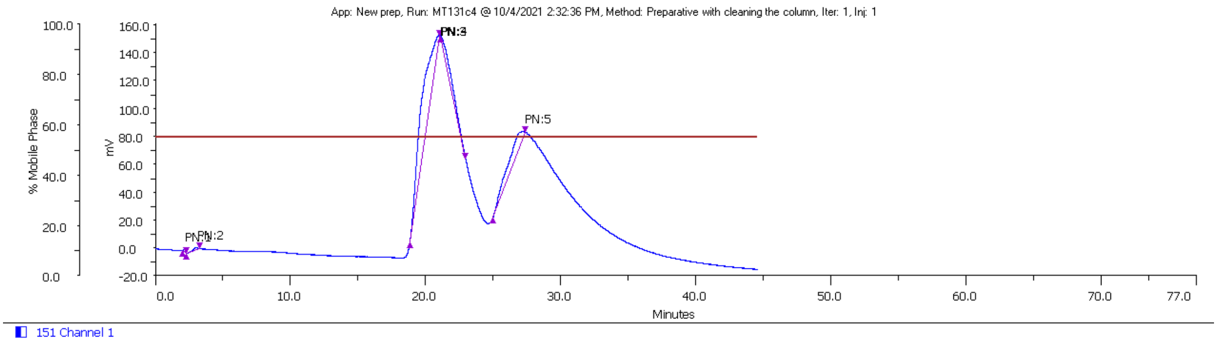

Figure S5. The high-performance liquid chromatography (HPLC) of injected racemic **10b** with Lux 5 µm i-Amylose-1 (150 x 21.2 mm) chiral column, MeCN/H_2O (0.15% TFA) eluting-solvent system, 18 mL/min flow rate and 254 nm detection wavelength at ambient temperature.

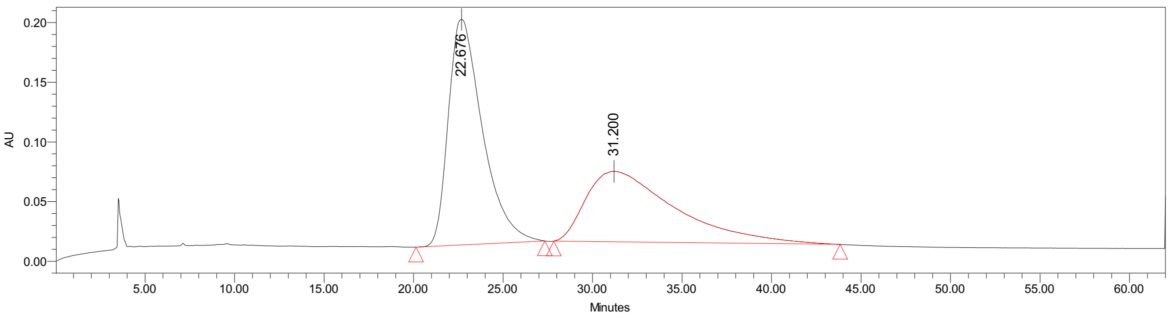

	Name	Retention Time	Area	% Area	Height	Int Type	Amount	Units	Peak Type	Peak Codes
1		22.676	25069955	55.13	188951	bb			Unknown	
2		31.200	20401374	44.87	59028	bb			Unknown	I08

Figure S6. The high-performance liquid chromatography (HPLC) of injected racemic **10b** (10 µL, 1 mg/mL in DMSO), with Lux 5 µm i-Amylose-1 (250 x 4.6 mm) chiral column, isocratic 55% MeCN+ 45% H_2O (with 0.15% TFA) eluting-solvent system, 0.8 mL/min flow rate and 254 nm detection wavelength at ambient temperature.

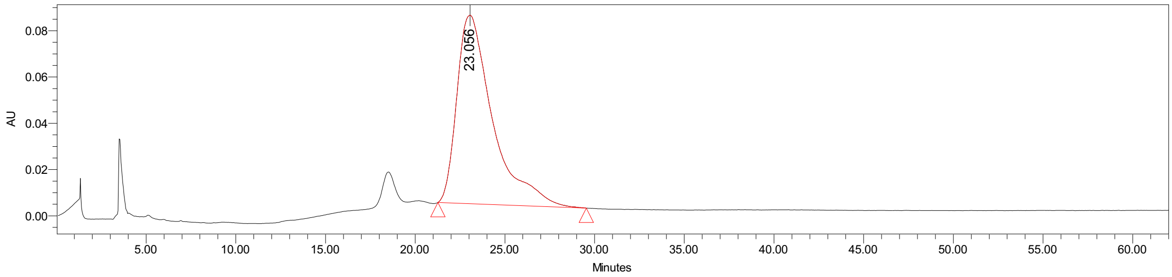

	Name	Retention Time	Area	% Area	Height	Int Type	Amount	Units	Peak Type	Peak Codes
1		23.056	11274881	100.00	81476	bb			Unknown	

Figure S7. The high-performance liquid chromatography (HPLC) of injected racemic **10b (-)** (10 µL, 1 mg/mL in DMSO), with Lux 5 µm i-Amylose-1 (250 x 4.6 mm) chiral column, isocratic 55% MeCN+ 45% H_2O (with 0.15% TFA) eluting-solvent system, 0.8 mL/min flow rate and 254 nm detection wavelength at ambient temperature.

10b (-): $[\alpha]_D^{25}$–230 (c, 0.167, DMSO);^{1}H NMR (400 MHz, $(CD_3)_2SO$) δ 7.71 (d, J = 8.1 Hz, 2H), 7.58 (s, 1H), 7.10 (d, J = 6.2 Hz, 2H), 7.00 (d, J = 7.1 Hz, 3H), 6.88 (d, J = 8.1 Hz, 2H), 4.84 (s, 1H), 4.70 (d, J = 8.8 Hz, 2H), 3.38 (d, J = 17.0 Hz, 1H), 2.63 (d, J = 17.3 Hz, 1H), 1.92 (s, 3H), 0.70–0.61 (m, 1H), -0.30– -0.33 (m, J = 7.2 Hz, 4H); ^{13}C NMR (100 MHz, $(CD_3)_2SO$) δ 169.1, 157.9, 152.7, 147.0, 146.0, 141.2, 140.0, 139.1, 134.9, 132.3, 129.4, 129.3, 127.8, 127.7, 126.8, 125.5, 112.1, 107, 73.6, 51.0, 20.9, 15.8, 10.8; HRMS (ESI-TOF) *m/z* $[M+H]^+$ Calcd for $C_{30}H_{25}N_2O_3S^+$ 493.1586, Found 493.1603

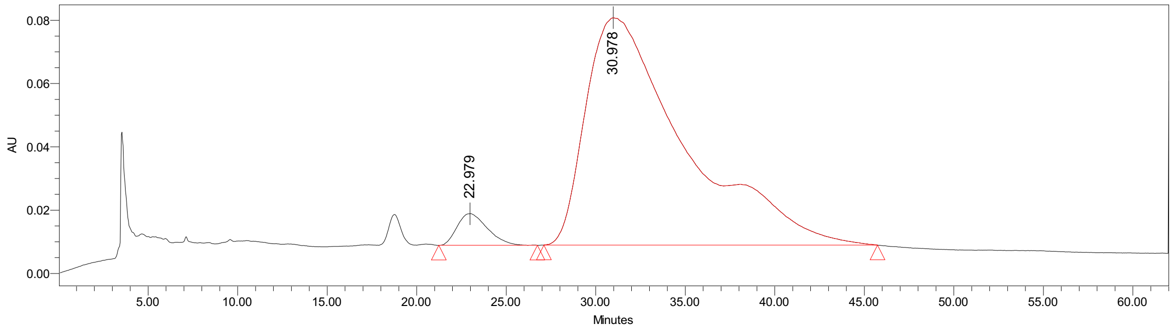

	Name	Retention Time	Area	% Area	Height	Int Type	Amount	Units	Peak Type	Peak Codes
1		22.979	1172595	4.07	9982	bb			Unknown	
2		30.978	27618778	95.93	71841	bb			Unknown	

Figure S8. The high-performance liquid chromatography (HPLC) of injected racemic **10b (+)** (10 µL, 1 mg/mL in DMSO), with Lux 5 µm i-Amylose-1 (250 x 4.6 mm) chiral column, isocratic 55% MeCN+ 45% H_2O (with 0.15% TFA) eluting-solvent system, 0.8 mL/min flow rate and 254 nm detection wavelength at ambient temperature.

10b (+): $[\alpha]_D^{25}$ +232 (c, 0.189, DMSO); ^{1}H NMR (400 MHz, $(CD_3)_2SO$) δ 7.71 (d, J = 8.1 Hz, 2H), 7.59 (s, 1H), 7.10 (d, J = 5.9 Hz, 2H), 7.00 (d, J = 6.8 Hz, 3H), 6.88 (d, J = 8.0 Hz, 2H), 4.86 (s, 1H), 4.74 (d, J = 2.0 Hz, 2H), 3.37 (d, J = 17.0 Hz, 1H), 2.65 (d, J = 17.1 Hz, 1H), 1.93 (s, 3H), 0.70–0.64 (m, 1H), -0.29– -0.33 (m, 4H); ^{13}C NMR (100 MHz, $(CD_3)_2SO$) δ 168.7, 157.7, 152.6, 146.9, 146.7, 145.6, 141.0, 140.2, 139.0, 134.7, 132.2, 129.3, 129.2, 127.6, 126.6, 125.4, 112.1, 107.1, 73.1, 50.8, 20.7, 15.6, 10.7; HRMS (ESI-TOF) *m/z* $[M+H]^+$ Calcd for $C_{30}H_{25}N_2O_3S^+$ 493.1586, Found 493.1602

Experimental procedure for evaluation of fibrillation modulating properties.

Human wild-type α-synuclein was expressed and purified as described previously,[2] and denatured and refolded according to the published procedure,[1a] in order to enhance the reproducibility of the experiments. A 96-well plate (Corning 3650, *Kennebunk, ME, USA*) was loaded with samples containing wild-type α-synuclein (70 µM) and compound (100 µM) solubilized in PBS (10 mM) and DMSO (100 µM), followed by addition of 20 µM ThT (Sigma-Aldrich, Saint Louis, MO, USA) and a 2 mm glass bead. The plate was incubated at 37 °C using a FLUOstar Omega microplate reader (BMG Labtech GmbH, Ortenberg, Germany) set to orbital averaging, 500 cycles, and a cycle time of 600 seconds, during 70 hours. Each experiment was performed in triplicate. The formation of amyloid fibers was followed by ThT fluorescence as a function of time (λ_{ex} = 440 nm, λ_{em} = 480 nm)[3].

Recombinant $A\beta_{1-40}$ was obtained from AlexoTech AB (Umeå, Sweden) and fibril formation was monitored according to a previously published procedure.[4] Lyophilized $A\beta_{1-40}$ *was dissolved in 20 mM NaOH, and the monomeric form was collected in PBS* buffer containing 1 mM EDTA 0.02% NaN_3 (buffer A) *after* size-exclusion chromatography (Superdex 75 10/300 GL; GE Healthcare, Chicago, IL, USA). Thioflavin T *assay for* $A\beta_{1-40}$ *was performed using a* 5 µM peptide concentration in buffer A supplemented with 40 µM Thioflavin T (Sigma-Aldrich, Saint Louis, MO, USA). Respective compounds were dissolved in DMSO at a stock concentration of 20 mM and diluted 1000x accordingly in buffer A. Fluorescence measurements were performed at 37 °C in a 96 microtiter-plate, (black walls and clear bottoms, *Corning, Kennebunk, ME, USA*) using a FLUOstar Omega microplate reader (BMG Labtech GmbH, Ortenberg, Germany) with an excitation wavelength of 430 nm and an emission wavelength of 480 nm. The samples were shaken for 1 s at 100 rpm every 30 mins before measuring. All experiments were performed in at least triplicates, and each experiment has been verified two times or more.

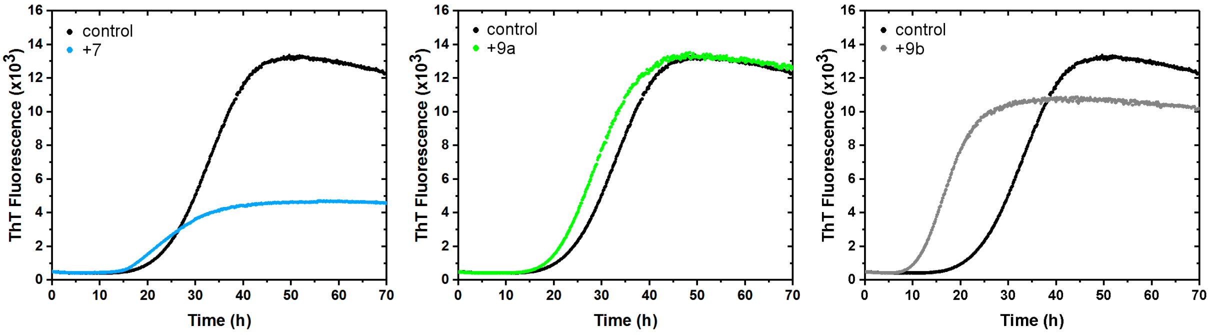

Figure S9. Evaluation of compound **7**, **9a** and **9b** and for their effect against α-synuclein fibrils *in vitro.*

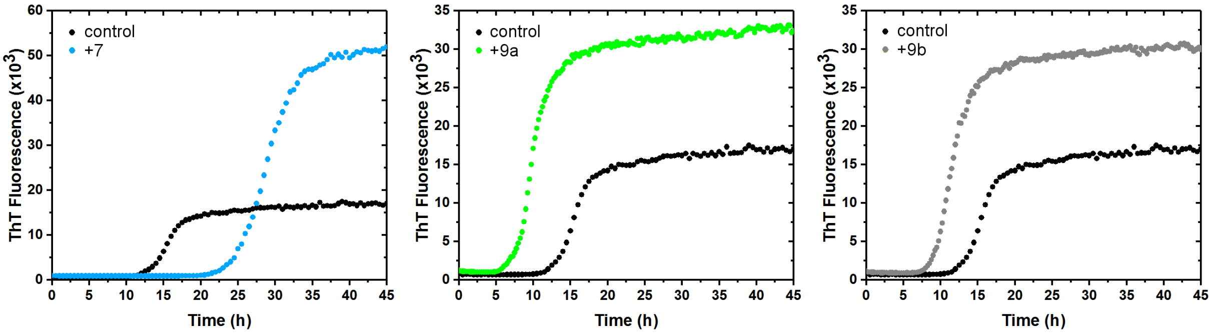

Figure S10. Evaluation of compound **7**, **9a** and **9b** and or their effect against Amyloid β fibrils *in vitro.*

Note: To verify that the low fluorescence intensity did indeed correspond to a competitive binding with ThT, mature fibrils were allowed to form in the absence of compound for 70 h, whereupon the supposed fibril binder was added. The remaining ThT fluorescence was then measured (**Figure S11**).

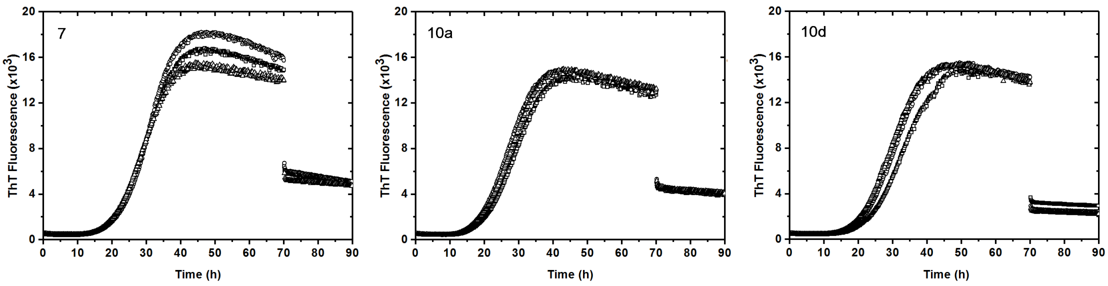

Figure S11: Compounds **7**, **10a** and **10e** showing low ThT amplitudes in the α-synuclein experiments displace bound ThT. Displacement of ThT was monitored after 70 hours when fibers were fully formed by addition of 100 µM of compound and further incubation for 20 hours. The decrease in signal intensity can be compared before (70 hours) and after addition (75 hours) of compound, when the signals had reached a steady plateau.

Note: In order to distinguish between inhibitors and compounds binding to mature fibrils, compounds **8b**, **10a** and **10b** were added to mature Amyloid $\beta_{1\text{–}40}$ fibrils (**Figure S12**).

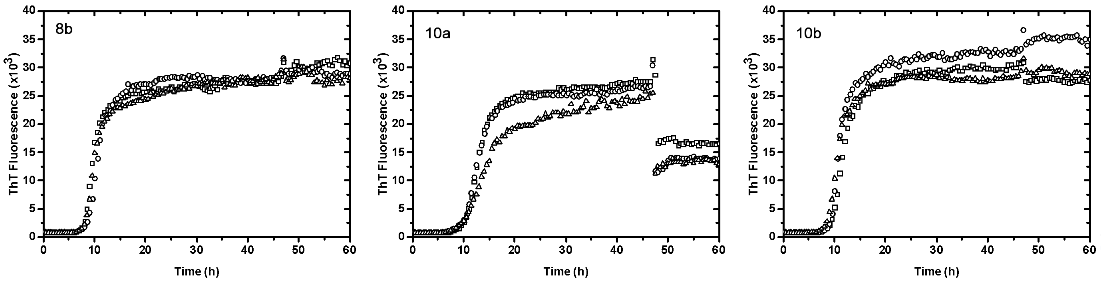

Figure S12: Compounds **8b**, **10a** and **10b** showing an inhibitory effect of $A\beta_{1\text{–}40}$ fiber formation were investigated for displacement of bound ThT. The ThT amplitude was monitored after 47 hours when fibers were fully formed by addition of 20 µM of compound and further incubation for 13 hours. The signal intensity can be compared before (47 hours) and after addition (50 hours) of compound, when the signals had reached a steady plateau. Only compound **10a** show minor displacement of ThT indicated by a reduction of amplitude.

Biological Evaluation of Enantiomers

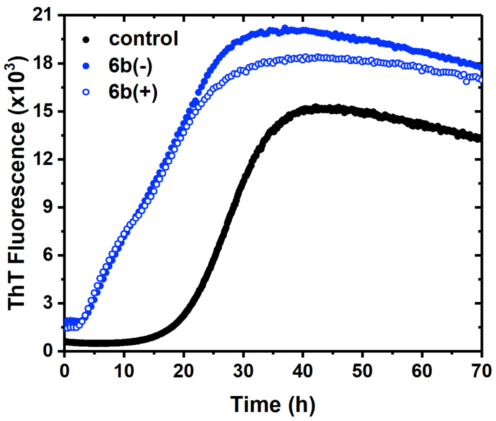

Figure S13: Evaluation of compound **6b (-)** and **6b (+)** for modulation of α-synuclein fibril formation *in vitro*. For control, α-synuclein was incubated in the absence of 2-pyridone.

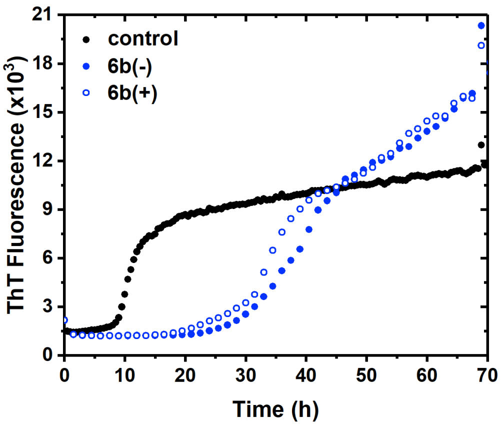

Figure S14: (A) Evaluation of compound **6b (-)** and **6b (+)** for modulation of Amyloid $\beta_{1\text{–}40}$ fibril formation *in vitro*. For control, Amyloid$\beta_{1\text{–}40}$ was incubated in the absence of 2-pyridone.

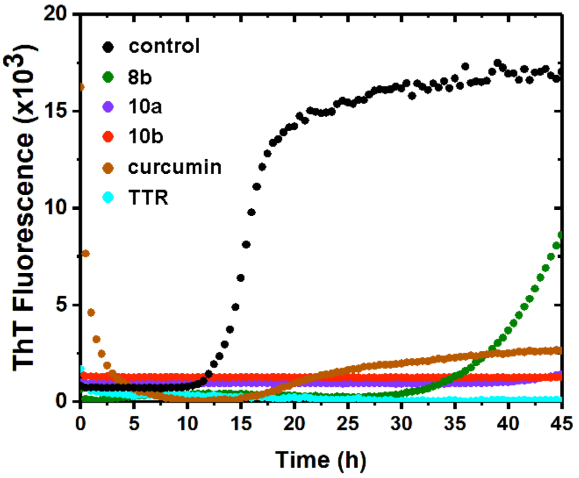

Figure S15: ThT plots for tested inhibitory compounds acquired for Amyloid$\beta_{1\text{–}40}$. Plots of the previously reported inhibitor TTR (Transthyretin)[5] and curcumin[6] were added as controls for Amyloid$\beta_{1\text{–}40}$ inhibition. For the controls, 200 nM of transthyretin was used, whereas for curcumin, 20 uM was used. Due to a higher sensitivity setting for these two controls, the signal amplitude has been adjusted for clarity.

Experimental procedure for investigation of elevated ThT amplitudes.

In contrast to outcompeting ThT, causing the fluorescence signal amplitude to decrease, some traces show a higher fluorescence signal compared to the control experiment. A test was therefore performed to distinguish whether these fibrils are modulated by the compound, or if the compound rather alter the binding mode of ThT to the fibril, causing the fluorescnence to increase. Fibril formation of α-synuclein propagates via a primary-nucleation, fibril-breakage mechanism at neutral pH.[2] The process of polymerization occurs according to a template-dependent effect where free monomers accommodate into the end of the parental fibril. The fibrillar architecture is hence preserved.[3] To selectively probe the template-dependent polymerization of α-synuclein and hence investigate if an alternative fibrillar architecture has been formed as a result of the 2-pyridone compound present, fibril-propagation during high seed conditions was performed. Using this approach, the rate of nucleation can be neglected since fibril-elongation by far dominates the process during the logarithmic-phase.[7] To asses this, 400 µl of mature fibrils were taken after 70 h, centrifuged at 17000 x g for 20 min. using a Micro Star 17 centrifuge (VWR, USA). The supernatant was then discarded. To remove any traces of 2-pyridone compound, 1 ml of Milli-Q water was added and the centrifugation was repeated as described above. This washing procedure was repeated in twice more. To maximize the number of free fibrillar ends, the fibrils were then sonicated using a Qsonica Q500 Sonicator (Qsonica, USA) (20% amplitude, 8 cycles, and 3 sec on, 3 sec off). 5% (v/v) of the washed and sonicated fibrils

were then added to a new ThT experiment together with monomeric α-synuclein in a new fibrilisation experiment, without 2-pyridone compound. The maximum fluorescence intensities of the seeded experiments vs. control experiment were then compared to investigate whether the compound modulate the fibrils formed or not (**Figure S16**). No significant differences in ThT fluorescence between the seeded experiments and the control was observed. Thus, the greatly increased fluorescence measured at the plateau phase of the fibrilisation experiments with **8a**, **10c** or **10e** present are not caused by any modulation of the fibril form, exerted by the 2-pyridone compound.

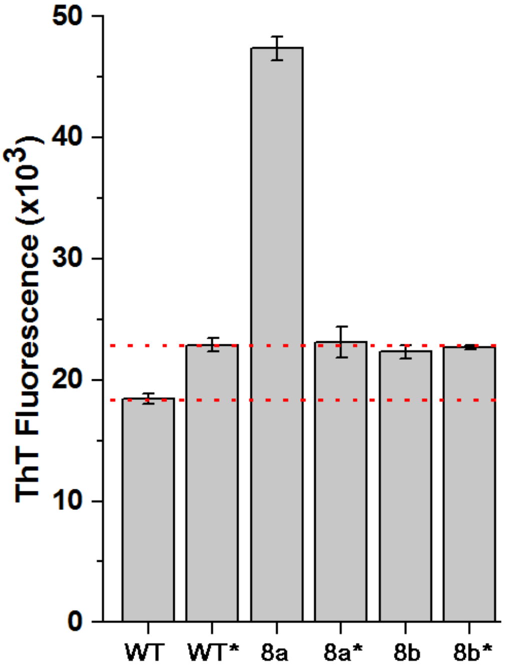

Figure S16: Experiments with α-synuclein in the presence of some 2-pyridone compounds showed greatly increased ThT amplitudes at the plateau phase (after about 30 h incubation). These observations are not due to modulation of the fibrils, exerted by the compounds. Monomeric α-synuclein was incubated together with 5% (v/v) washed fibrils taken from a previous experiment where the 2-pyridone-compounds were present. No significant difference in the ThT signal at the end of the incubation (70 h) was seen. The dotted line represents the difference in ThT signal between unseeded and seeded control experiment. **WT** = control experiment (α-synuclein only), **WT*** = seeded control experiment (seeded with fibrils formed in the absence of 2-pyridonecompound), **8a** = α-synuclein incubated in the presence of 8a, **8a*** = α-synuclein monomers seeded with fibrils generated together with compound **8a**, **8b** = α-synuclein incubated in the presence of 8b, **8b*** = α-synuclein monomers seeded with fibrils generated together with compound 8b.

Preparation of samples for TEM visualization of amyloid fibrils.

At the end point of the fibrilization experiments, samples (3.5µL) were applied to glow discharged formvar and carbon coated Cu-grids. The grids were washed and then negatively stained in 1.5% uranyl acetate for 2 x 15 s. A Talos 120C microscope (FEI, Eindhoven, The Netherlands) was used for sample examination, operating at 120kV. Micrographs were acquired with a Ceta 16M CCD camera (FEI, Eindhoven, The Netherlands) using TEM Image & Analysis software ver. 4.17 (FEI, Eindhoven, The Netherlands). Pictures were taken at 12 000 X and 40 000 X magnification, and are shown below. Fibers formed in the presence of compound **10a, 10d** and in the absence of compound showed no visual differences (control).

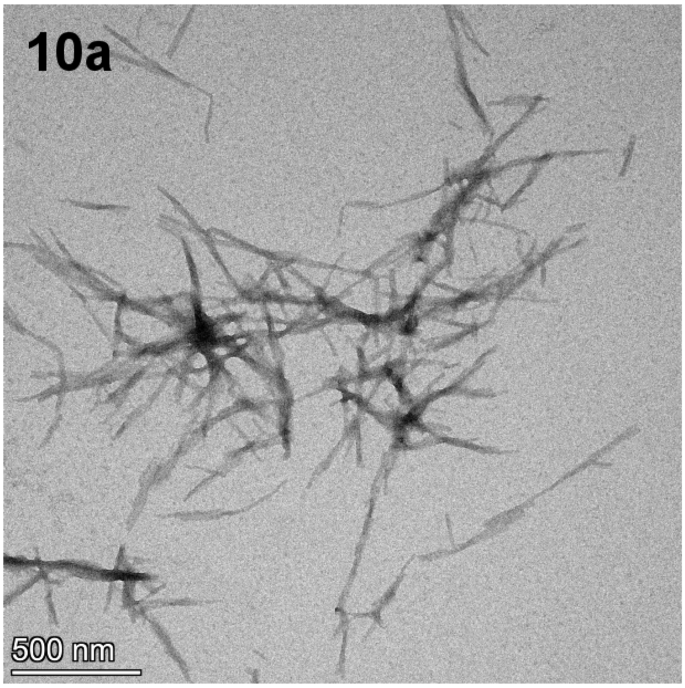

Figure S17: Transmission electron microscope images of fibers formed after the addition of **10a** to human wild-type α-synuclein

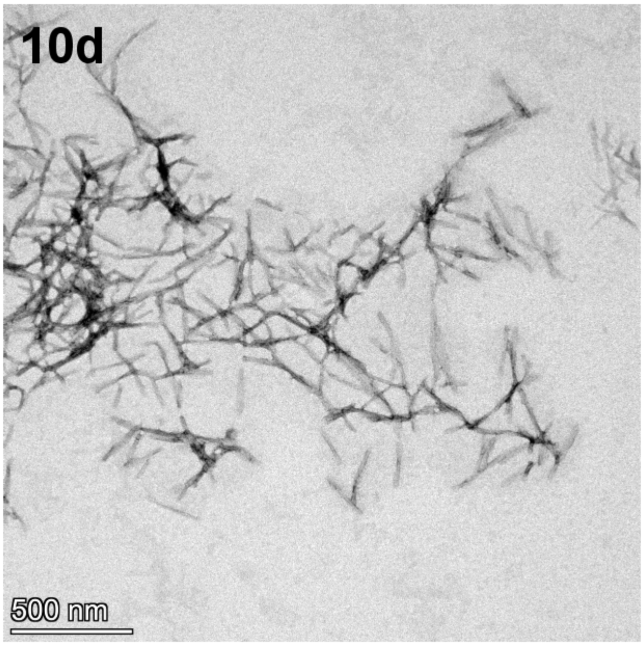

Figure S18: Transmission electron microscope images of fibers formed after the addition of **10d** to human wild-type α-synuclein

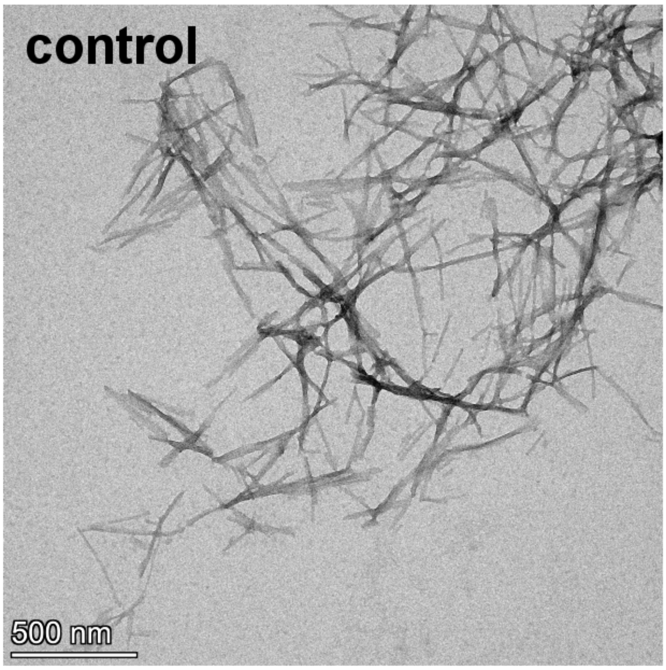

Figure S19: Transmission electron microscope images of human wild-type α-synuclein fibers

Crystallography. X-ray quality crystals of **5e** were obtained as a racemic mixture through recrystallization from absolute ethanol. Intensity data was collected with an Oxford Diffraction Excalibur 3 system, using ω-scans and Mo Kα (λ = 0.71073 Å) radiation.[8] The data was extracted, integrated and empirically absorption corrected using Crysalis RED.[9] The structure was solved by direct methods and refined by full-matrix least-squares calculations on F^2 using SHELXL and WinGX.[10,11] Molecular graphics were generated using Crystal Maker 9.2.[12] CCDC deposition number 2087355

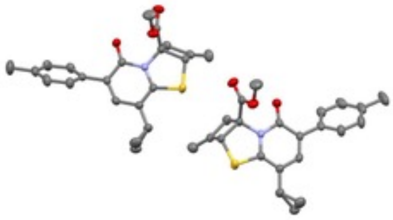

Figure S20. Crystal Structure of both enantiomers of **5e. 30%** ellipsoid contour probability.

Table 1. Crystal data and structure refinement details for the structure

	**5e**
**Empirical formula**	$C_{22}H_{21}NO_3S$
**Formula weight**	379.46
**Temperature/K**	293(2)
**Crystal system**	Monoclinic
**Space group**	$P2_1/a$
**a/Å**	22.774(13)
**b/Å**	19.5701(11)
**c/Å**	18.880(11)
**β/°**	153.27(17)
**Volume/Å^3**	3785(10)
**Z**	8
**ρ_{calc}g/cm^3**	1.332
**μ/mm^{-1}**	0.193
**F(000)**	1600
**Θ range for data collection/°**	3.432 to 29.859
**Reflections collected**	38488

**Independent reflections**	9303 [R_{int} = 0.0448, R_{sigma} = 0.0536]
**Data/restraints/parameters**	9303/0/487
**Goodness-of-fit on F^2**	1.006
**Final R indexes [I>=2σ (I)]**	R1 = 0.0608<br>wR2 = 0.1070
**Final R indexes [all data]**	R1 = 0.1037<br>wR2 = 0.1234
**Largest diff. peak/hole / e Å^{-3}**	0.302 / -0.293
**CCDC**	2087355

Copies of ^{1}H, ^{13}C{^{1}H} and ^{19}F NMR spectra.

Compound **1m**. ^{1}H NMR (400 MHz, $CDCl_3$) ^{13}C{^{1}H} NMR (100 MHz, $CDCl_3$)

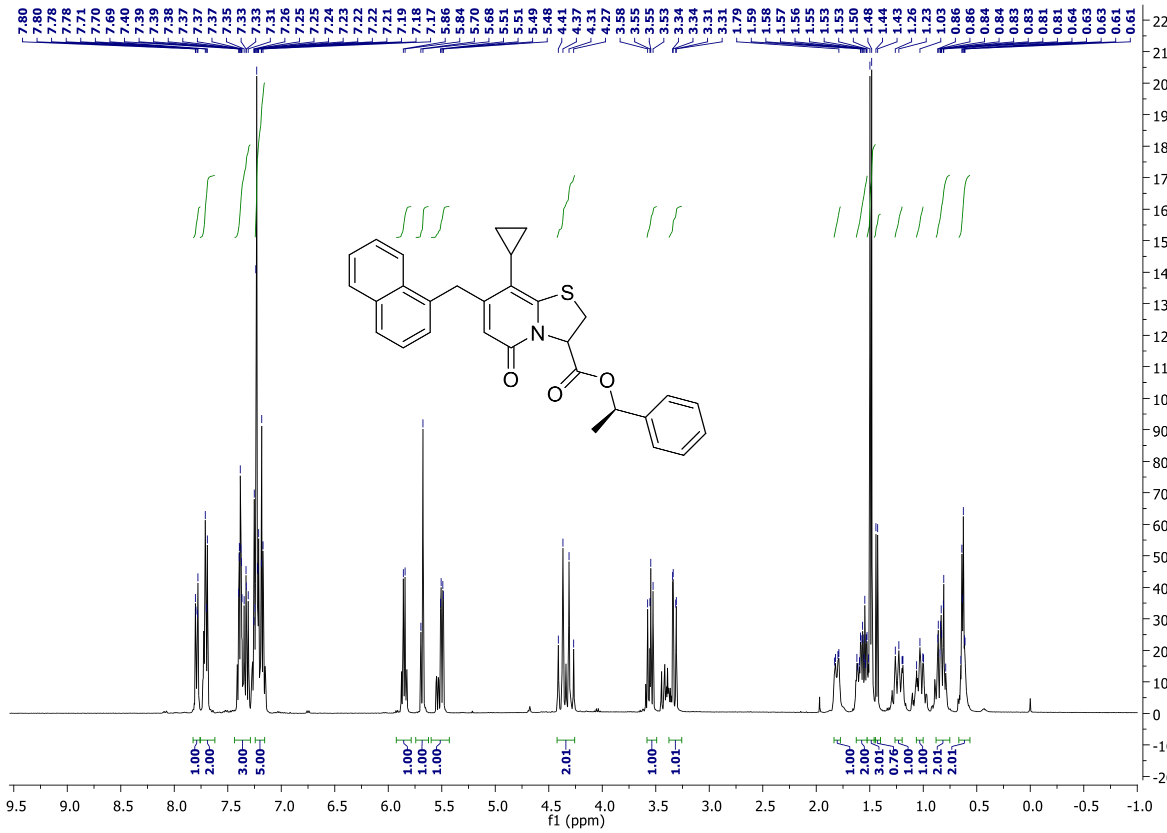

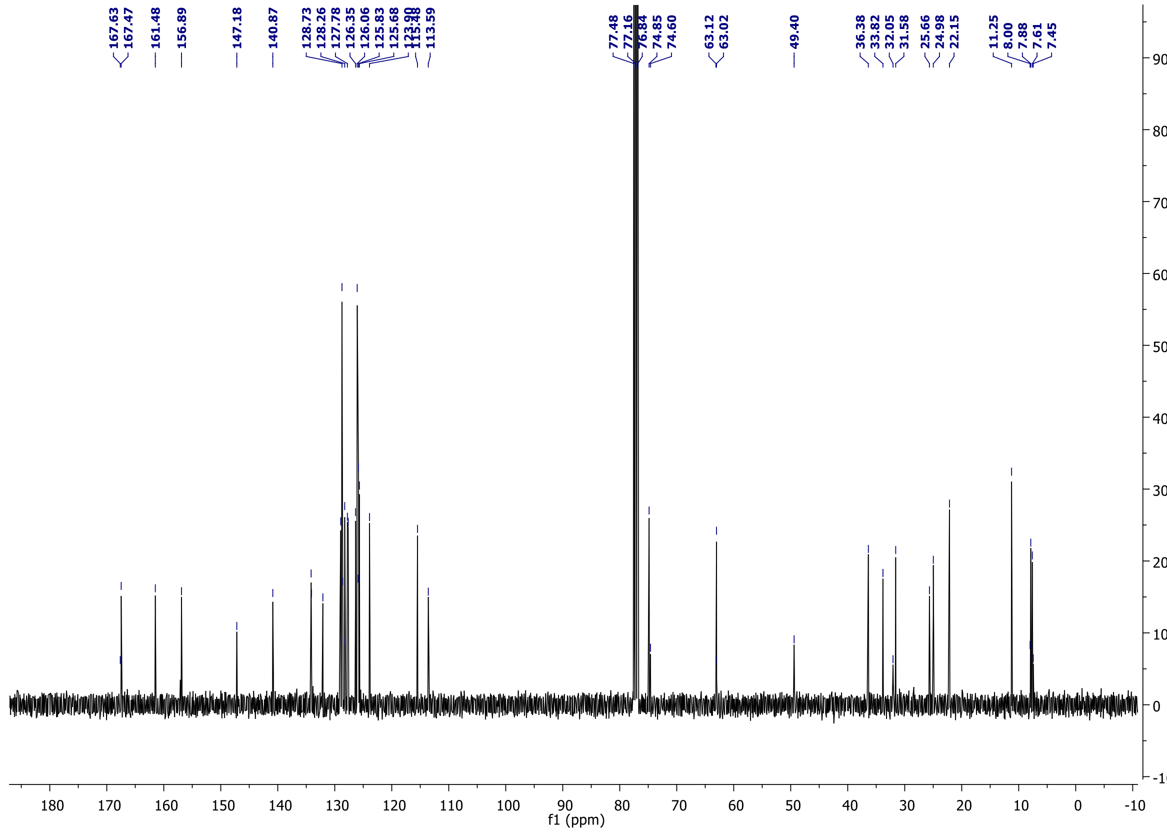

Compound **1n**. ^{1}H NMR (400 MHz, $CDCl_3$) $^{13}C\{^{1}H\}$ NMR (100 MHz, $CDCl_3$)

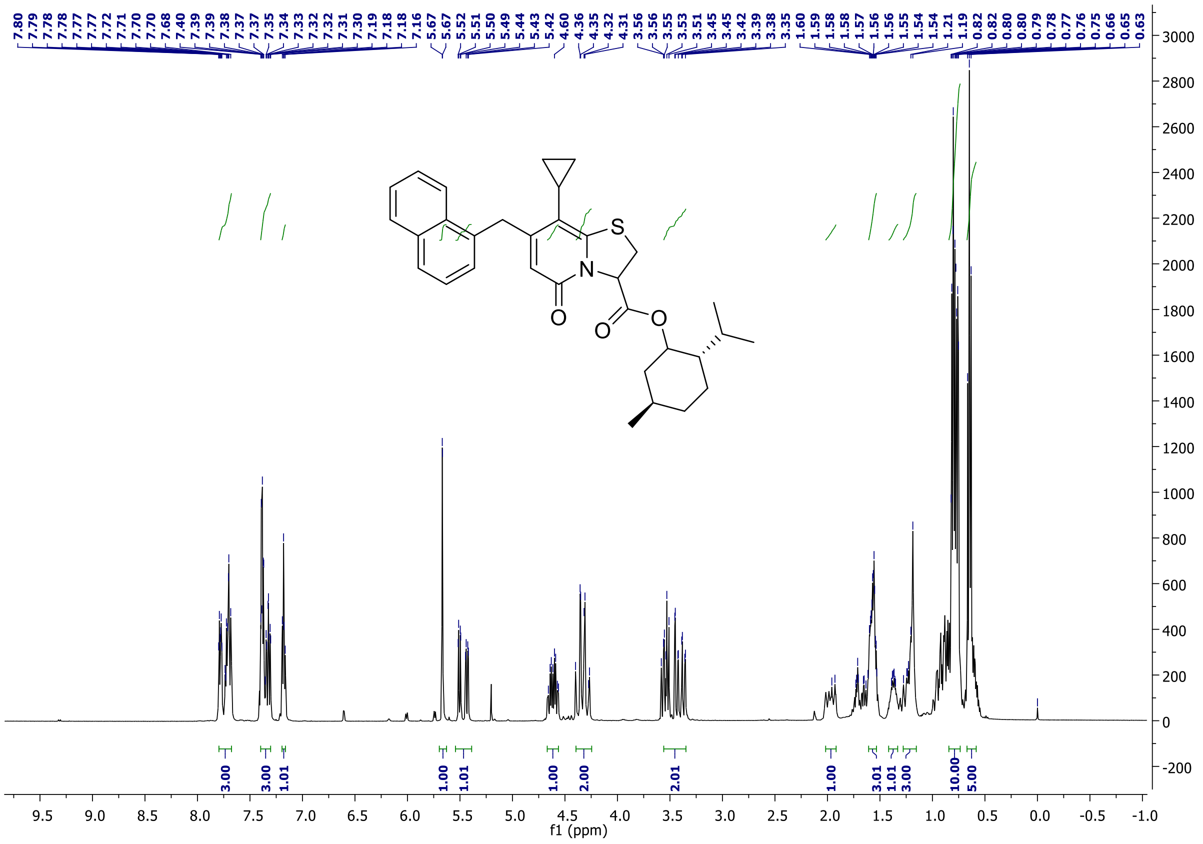

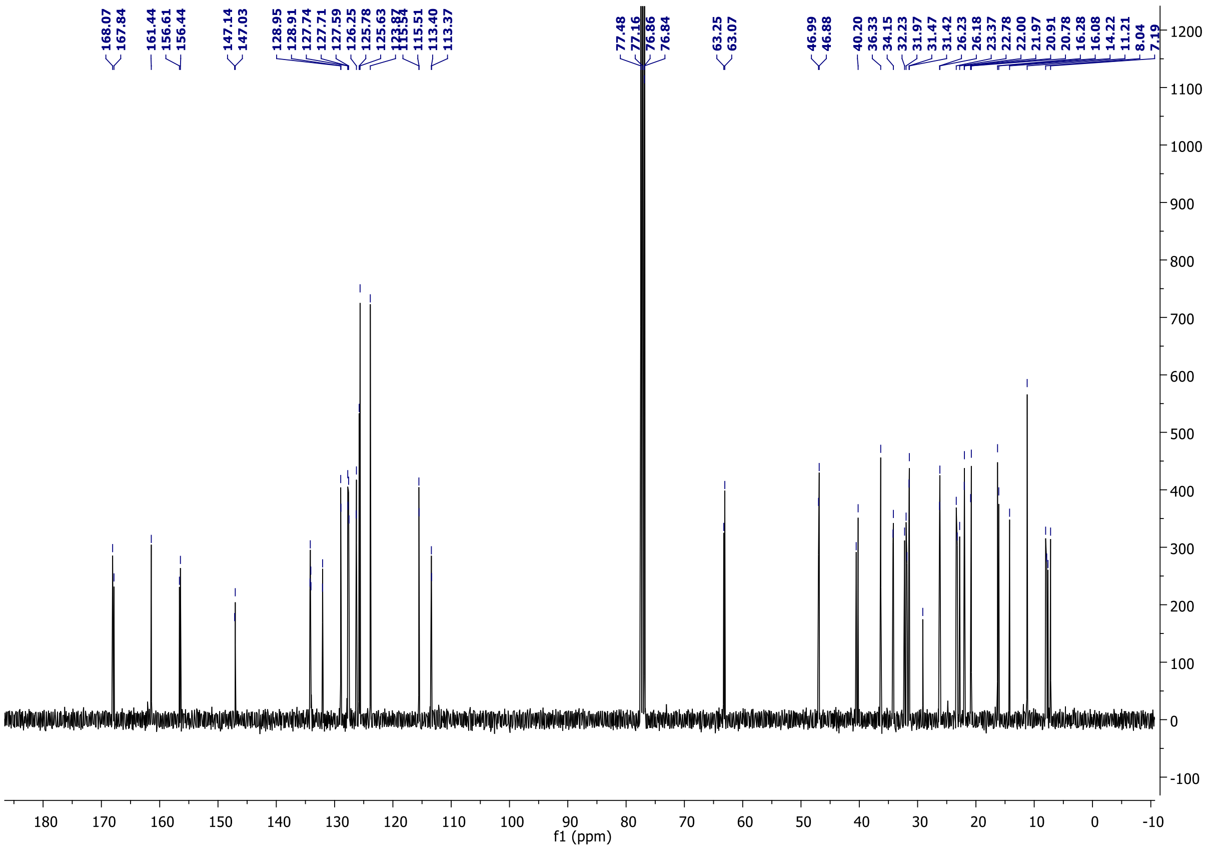

Compound **2a**. ^{1}H NMR (400 MHz, $CDCl_3$) $^{13}C\{^{1}H\}$ NMR (100 MHz, $CDCl_3$)

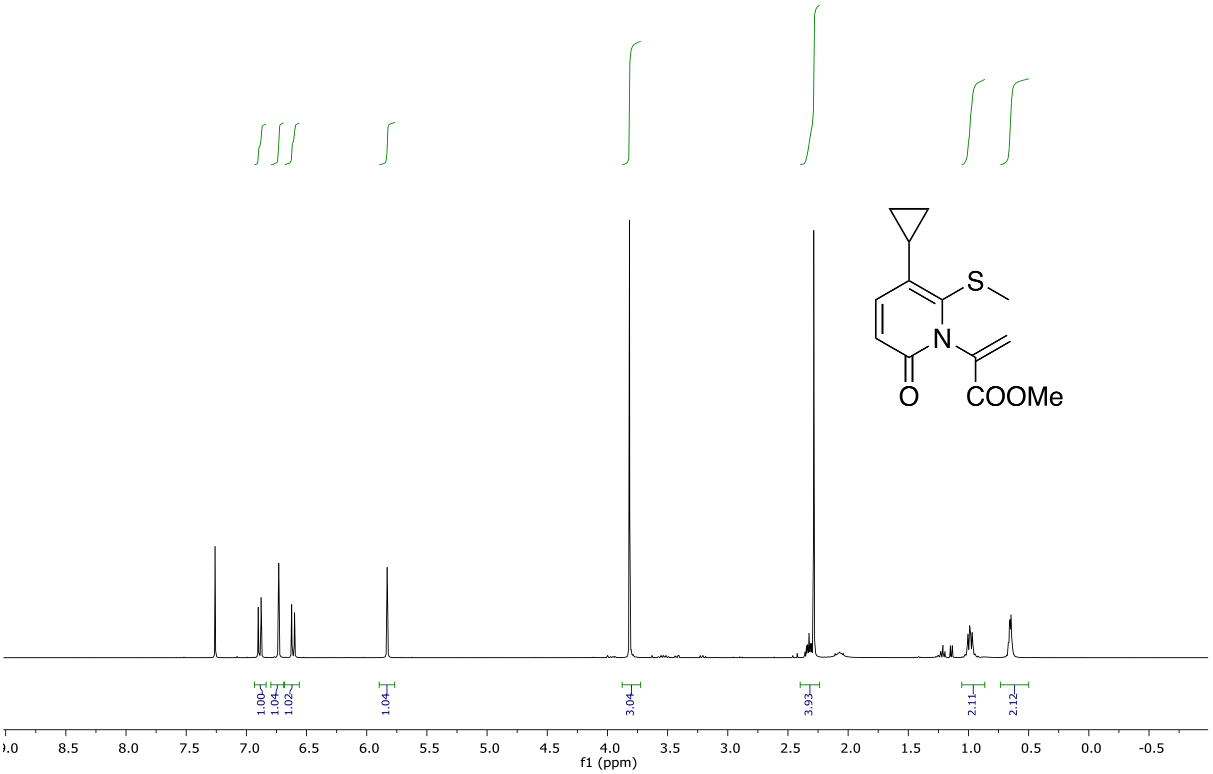

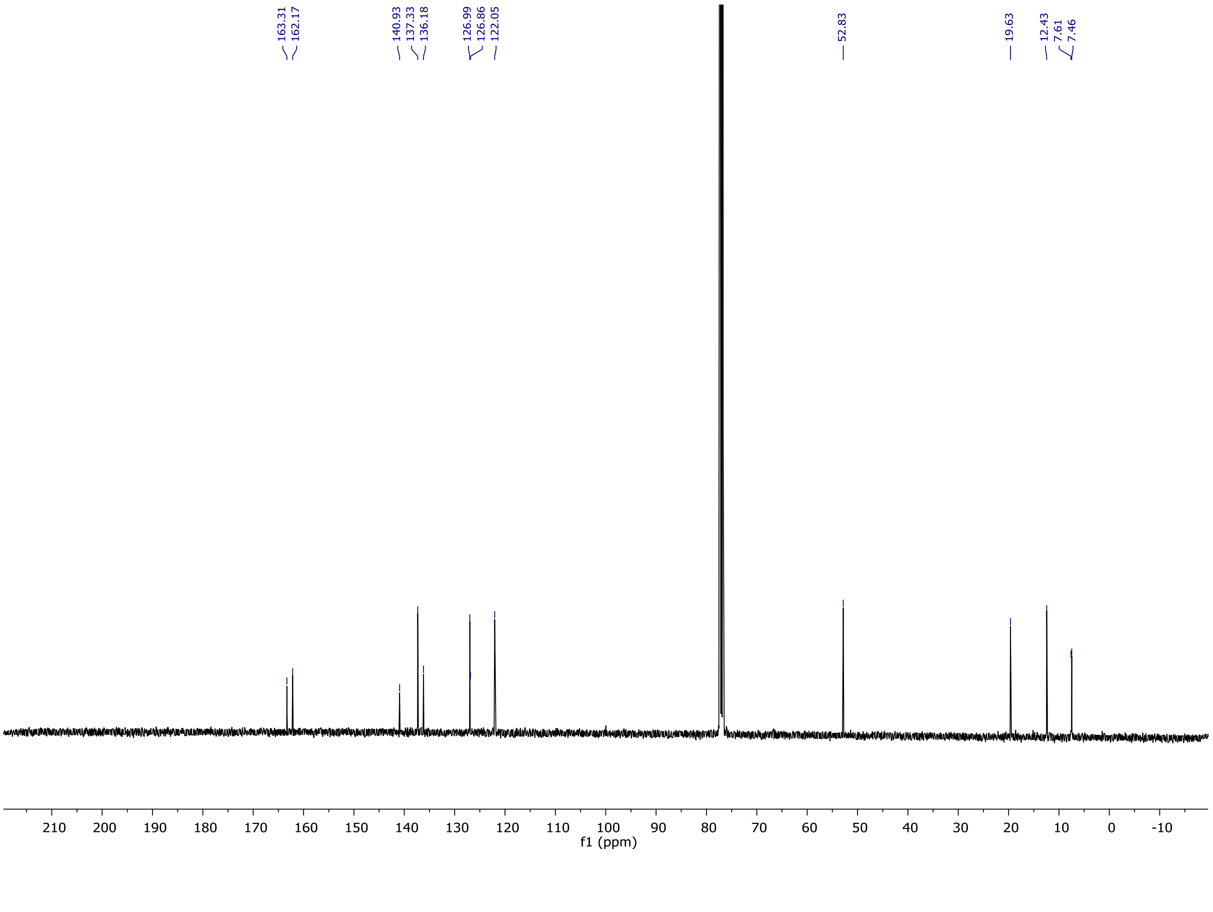

Compound **2b**. ^{1}H NMR (400 MHz, $CDCl_3$) $^{13}C\{^{1}H\}$ NMR (100 MHz, $CDCl_3$)

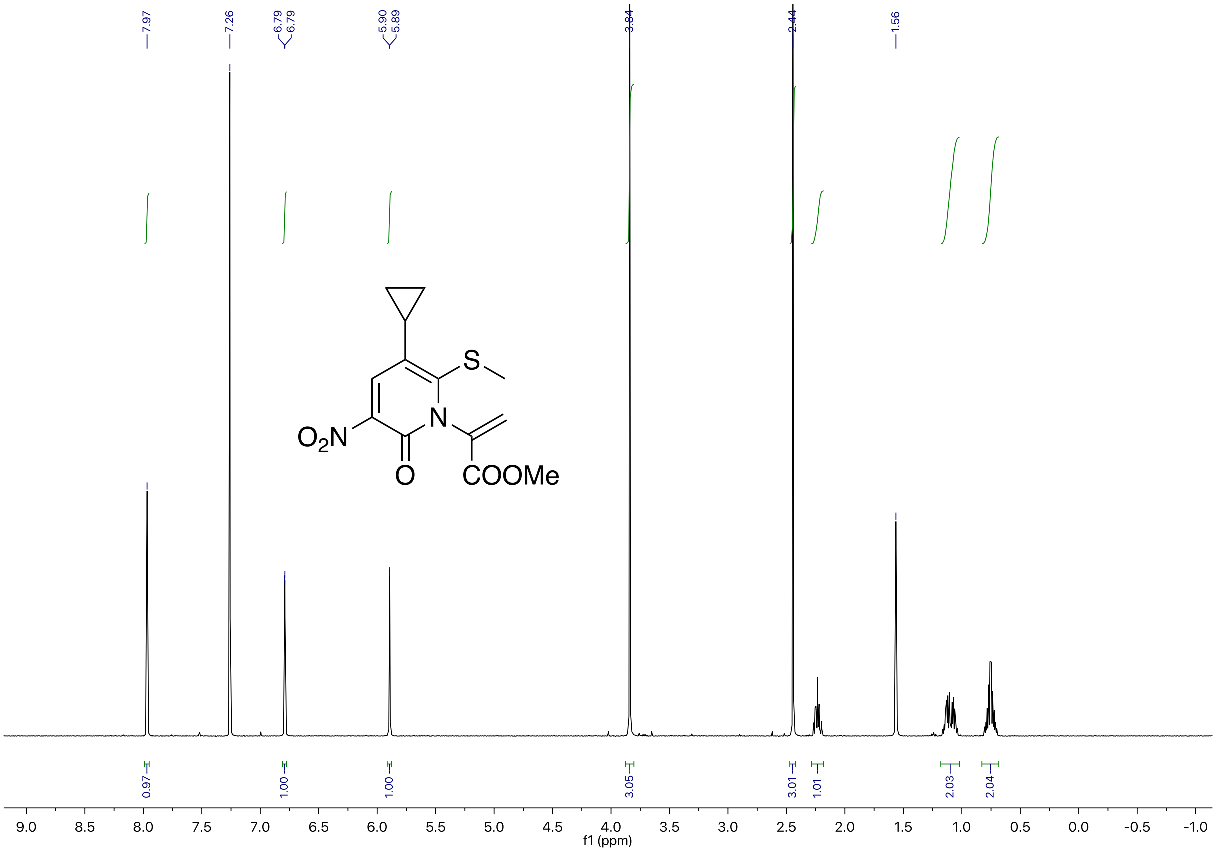

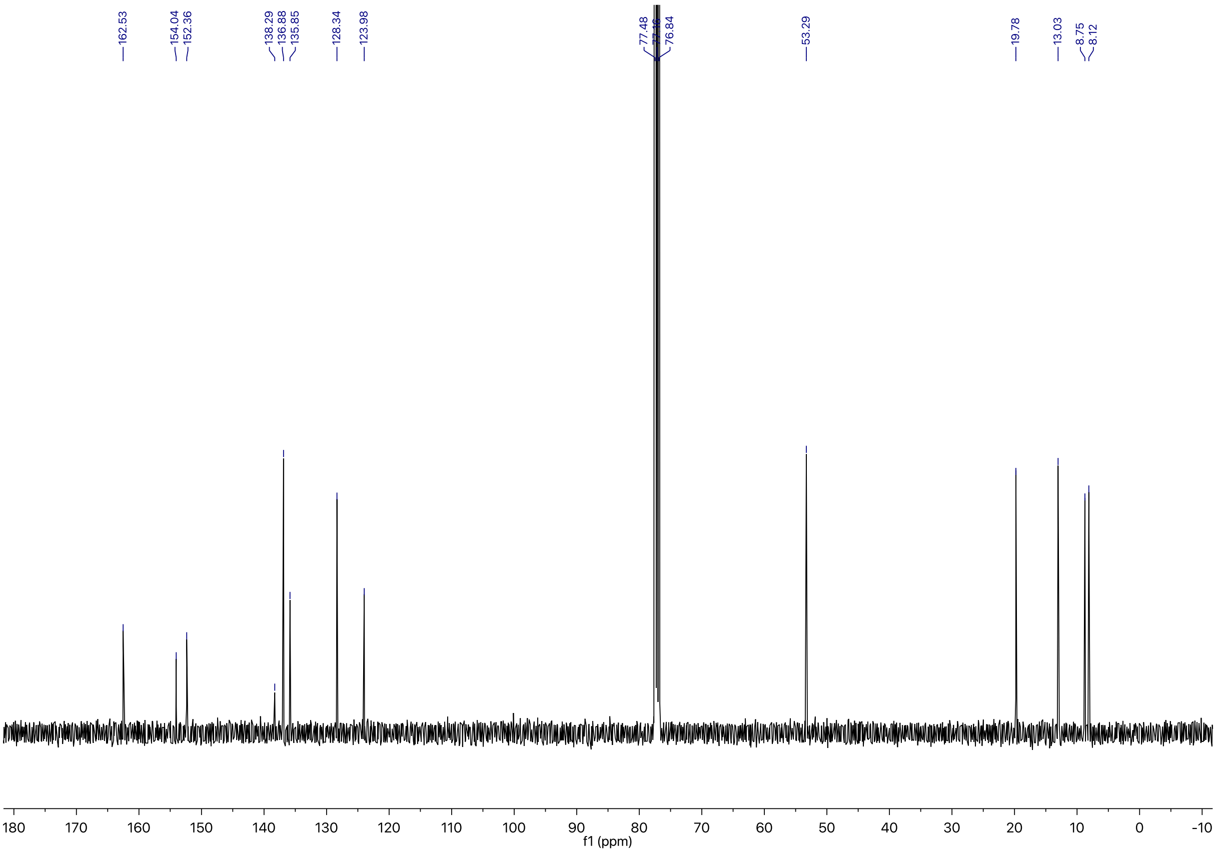

Compound **2c**. ^{1}H NMR [400 MHz, $(CD_3)_2SO$] ^{13}C{^{1}H} NMR [100 MHz, $(CD_3)_2SO$]

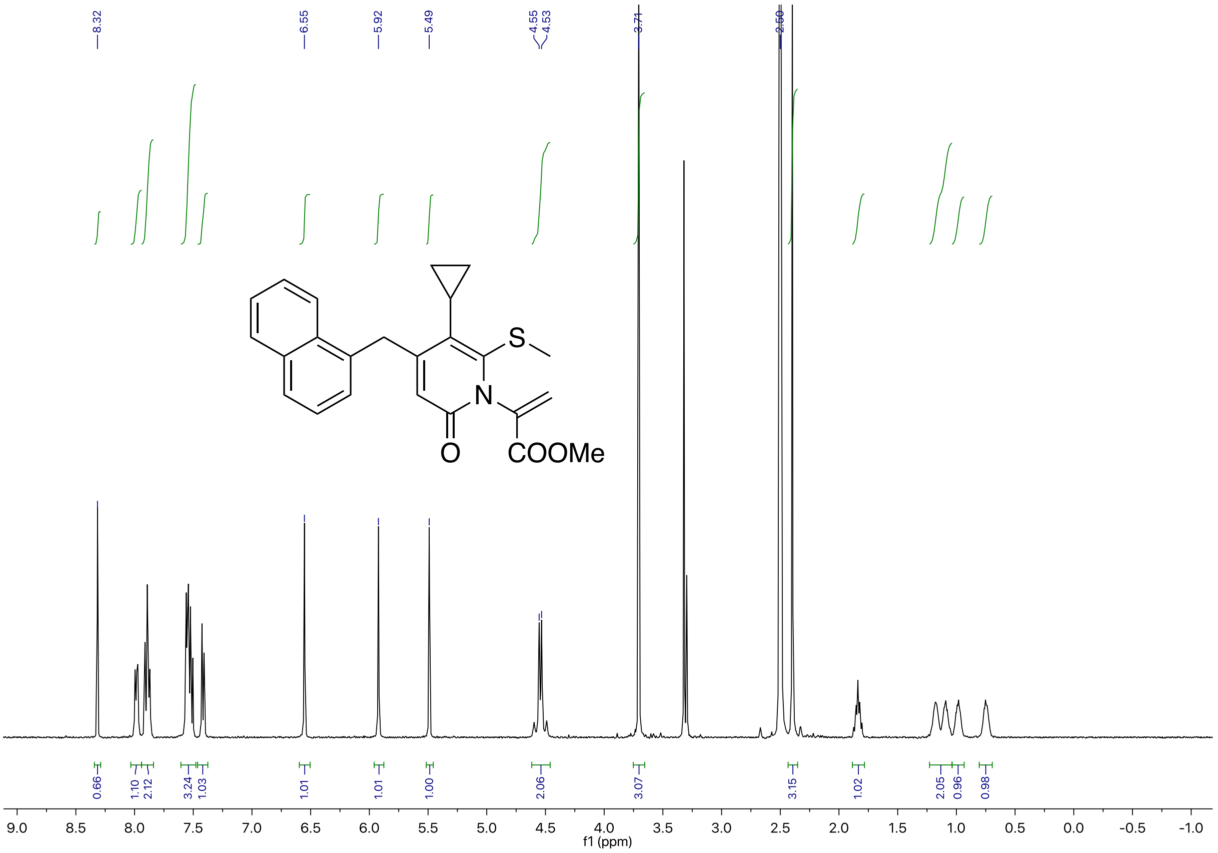

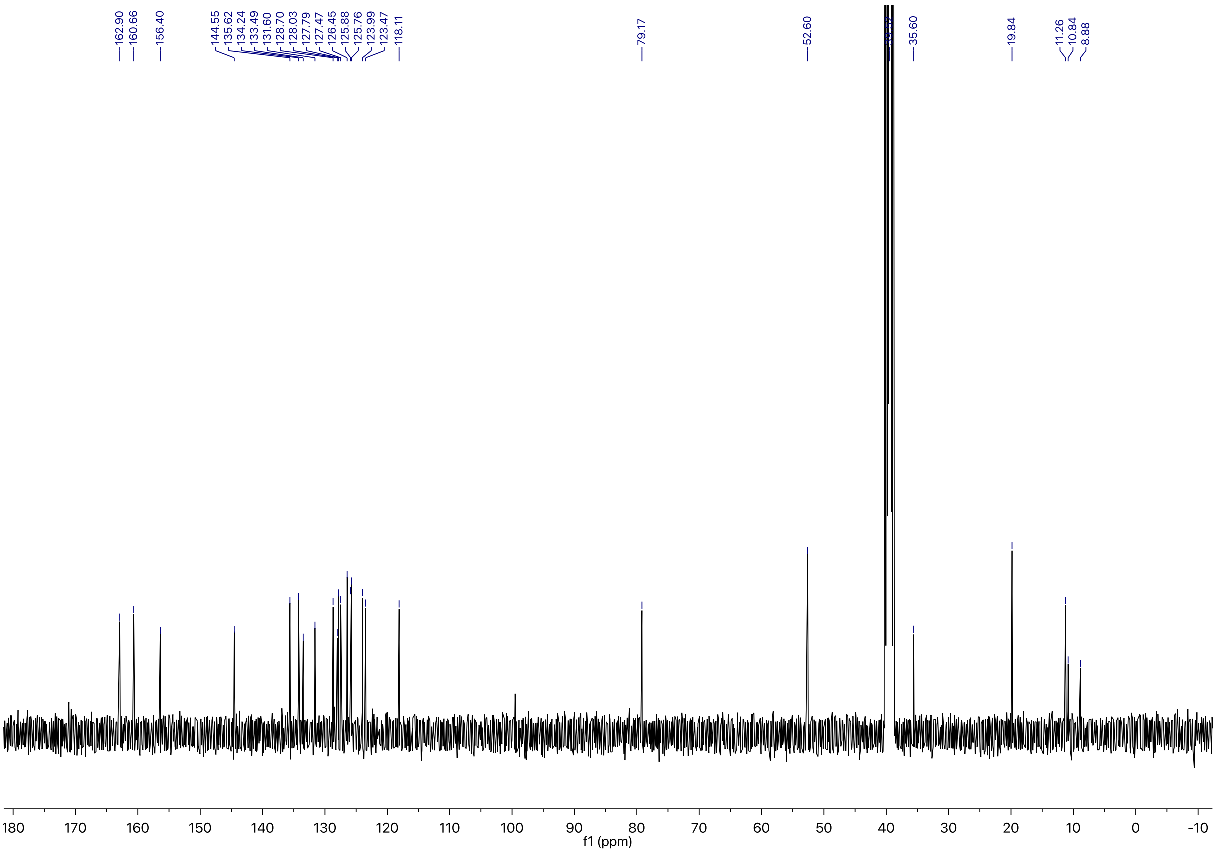

Compound **2d**. ^{1}H NMR [400 MHz, $(CD_3)_2SO$] ^{13}C{^{1}H} NMR [100 MHz, $(CD_3)_2SO$] ^{19}F NMR [376 MHz, $(CD_3)_2SO$] 343 K

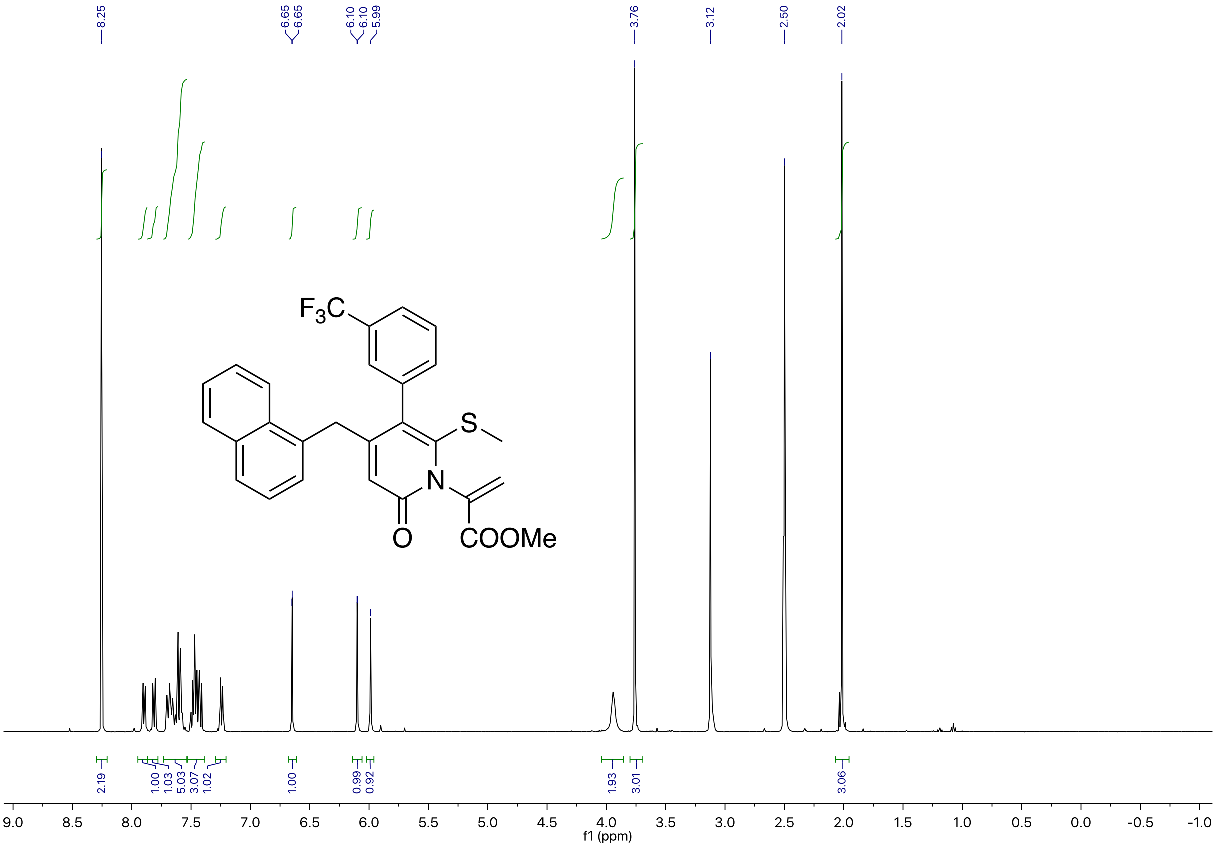

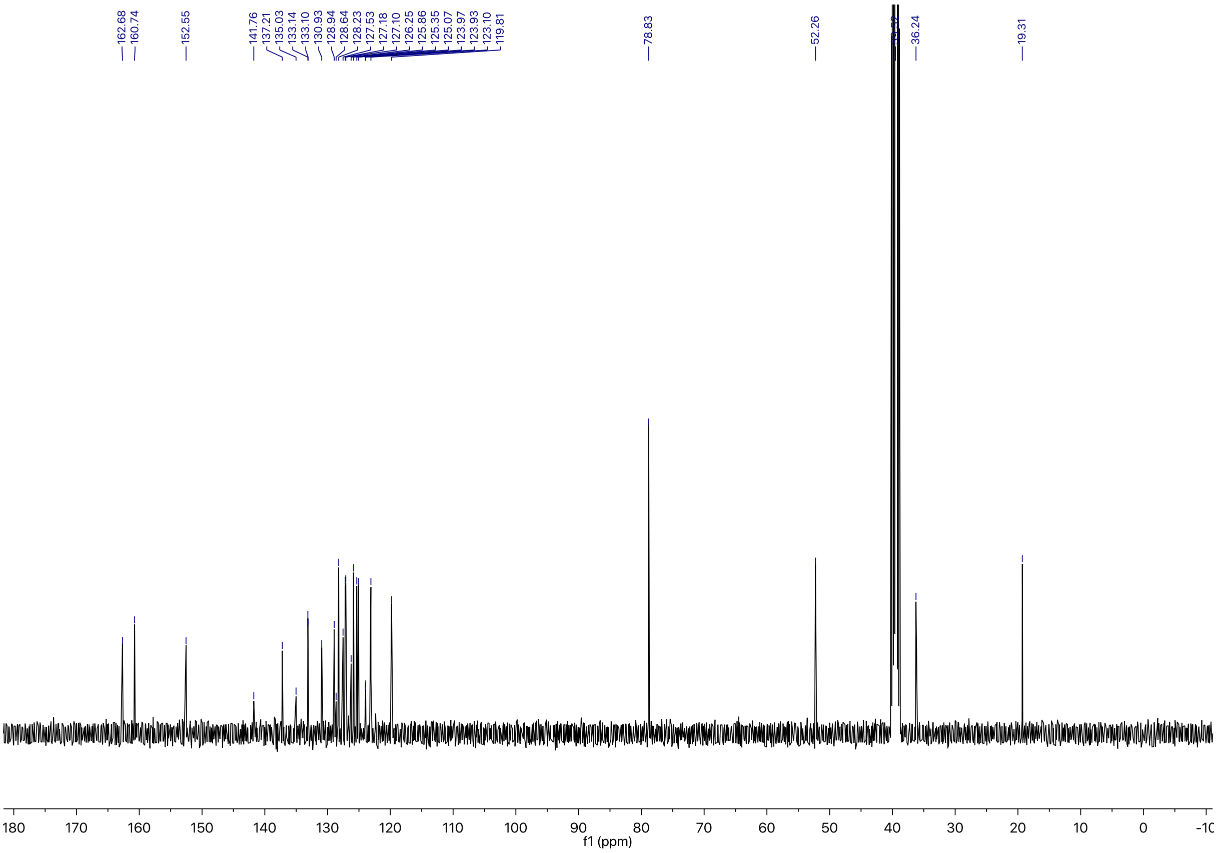

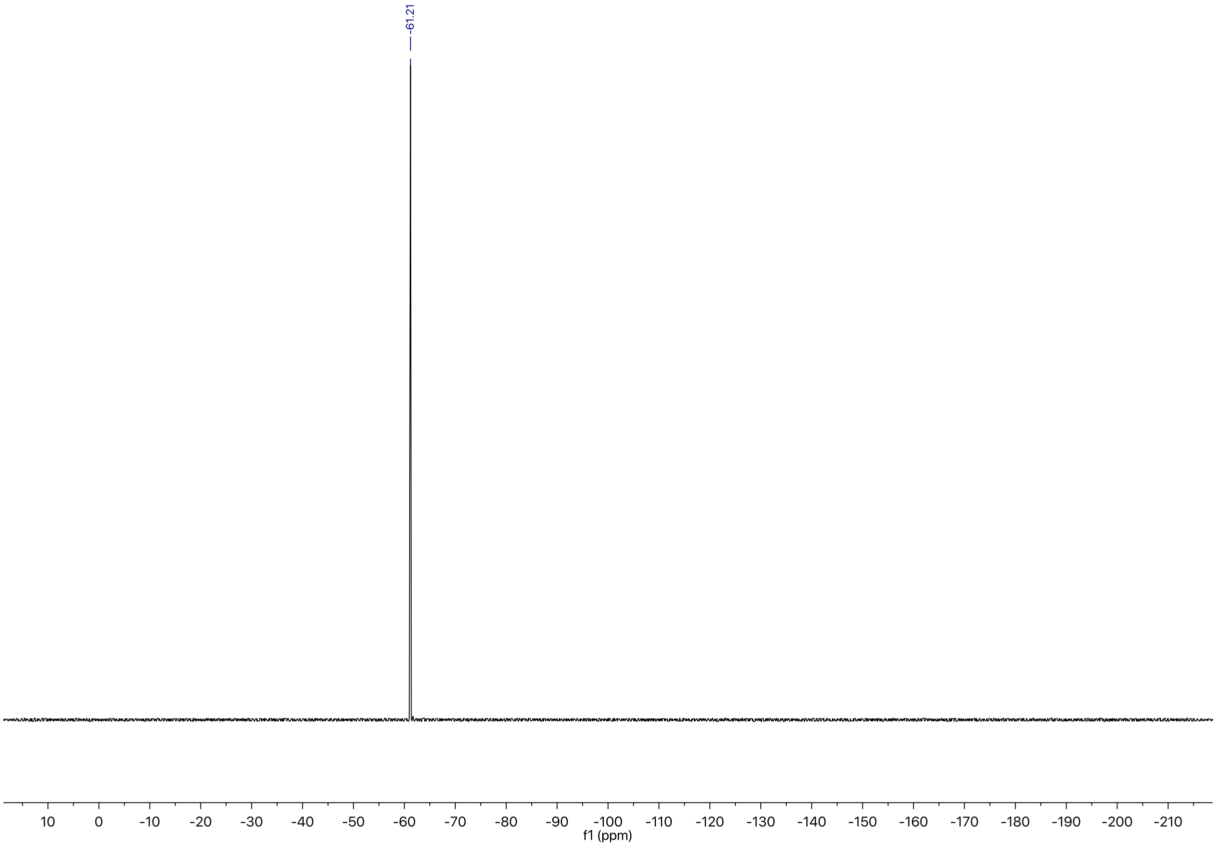
-61.21
10
0
-10
-20
-30
-40
-50
-60
-70
-80
-90
-100
-110
-120
-130
-140
-150
-160
-170
-180
-190
-200
-210
f1 (ppm)

Compound **4**. ^{1}H NMR (400 MHz, $CDCl_3$) $^{13}C\{^{1}H\}$ NMR (100 MHz, $CDCl_3$)

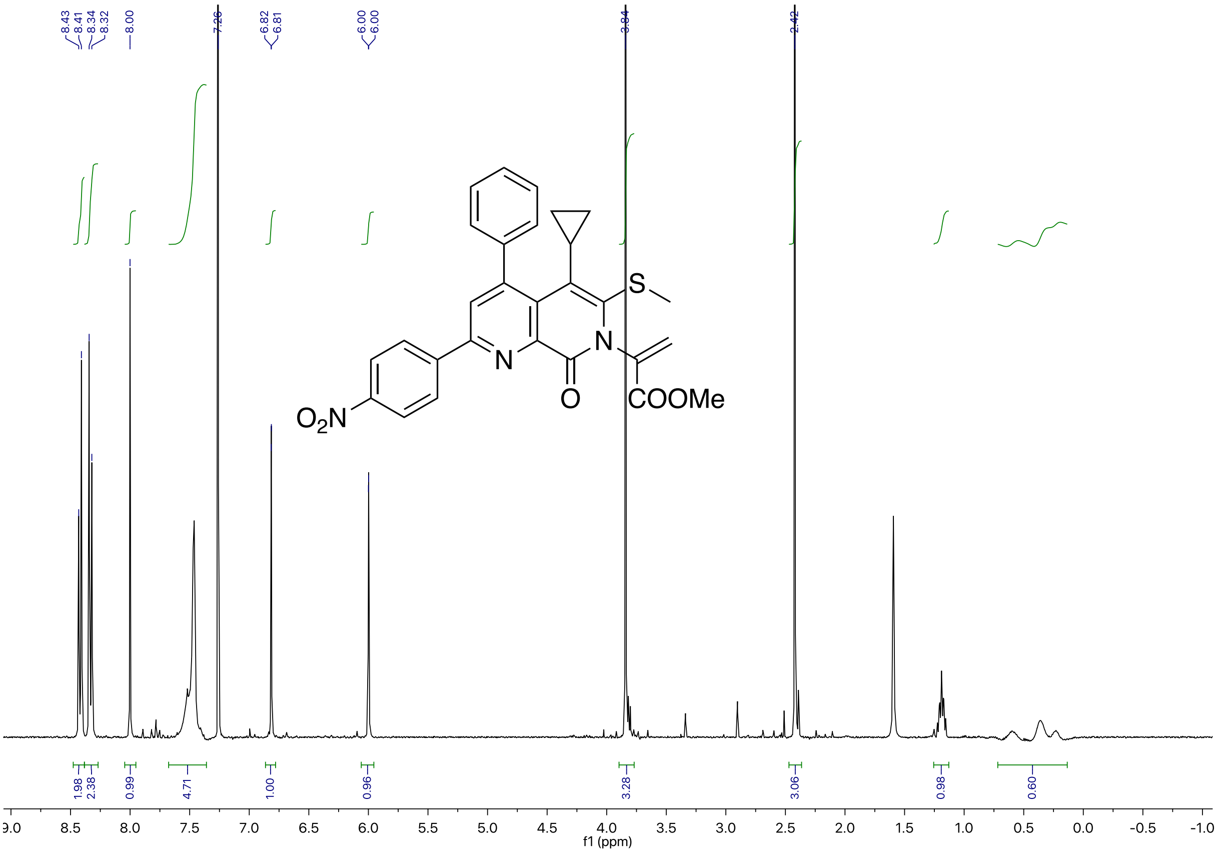

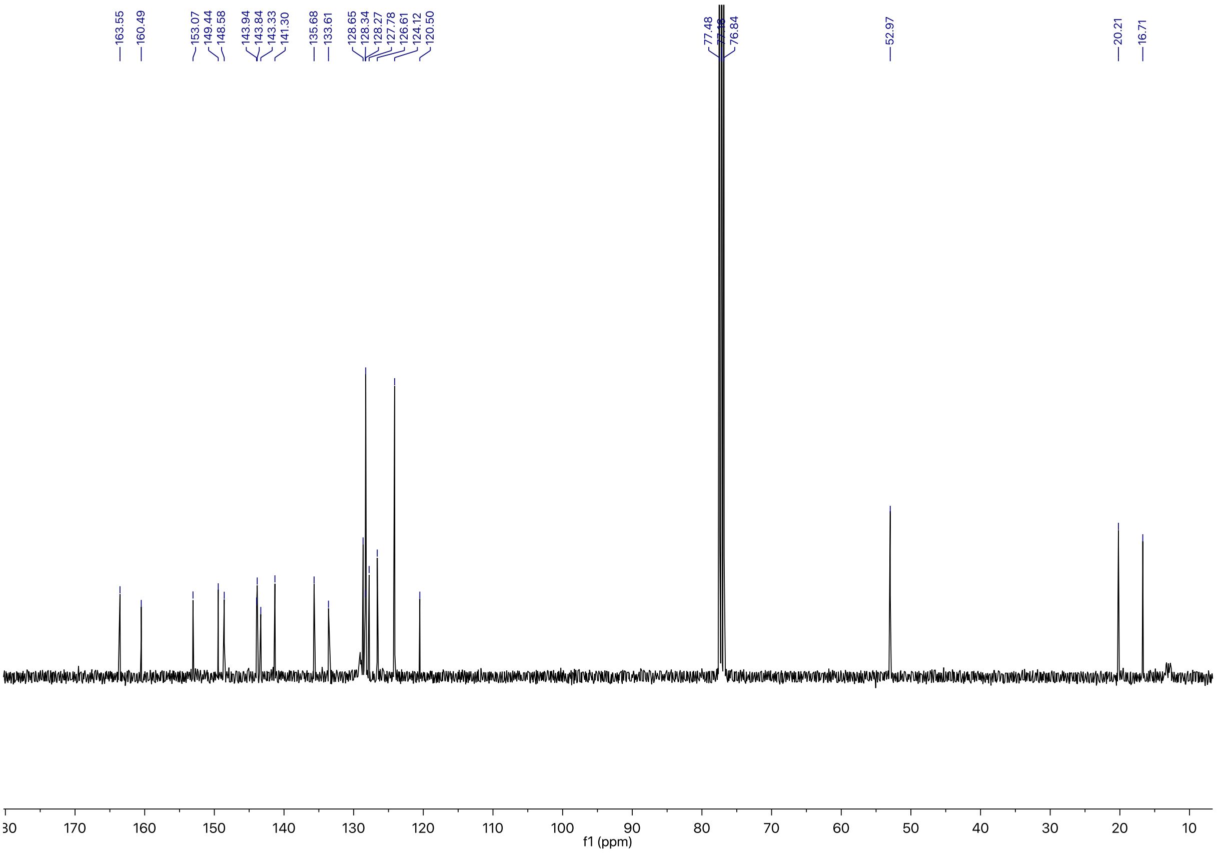

Compound **2e**. ^{1}H NMR (600 MHz, $CDCl_3$) $^{13}C\{^{1}H\}$ NMR (151 MHz, $CDCl_3$)

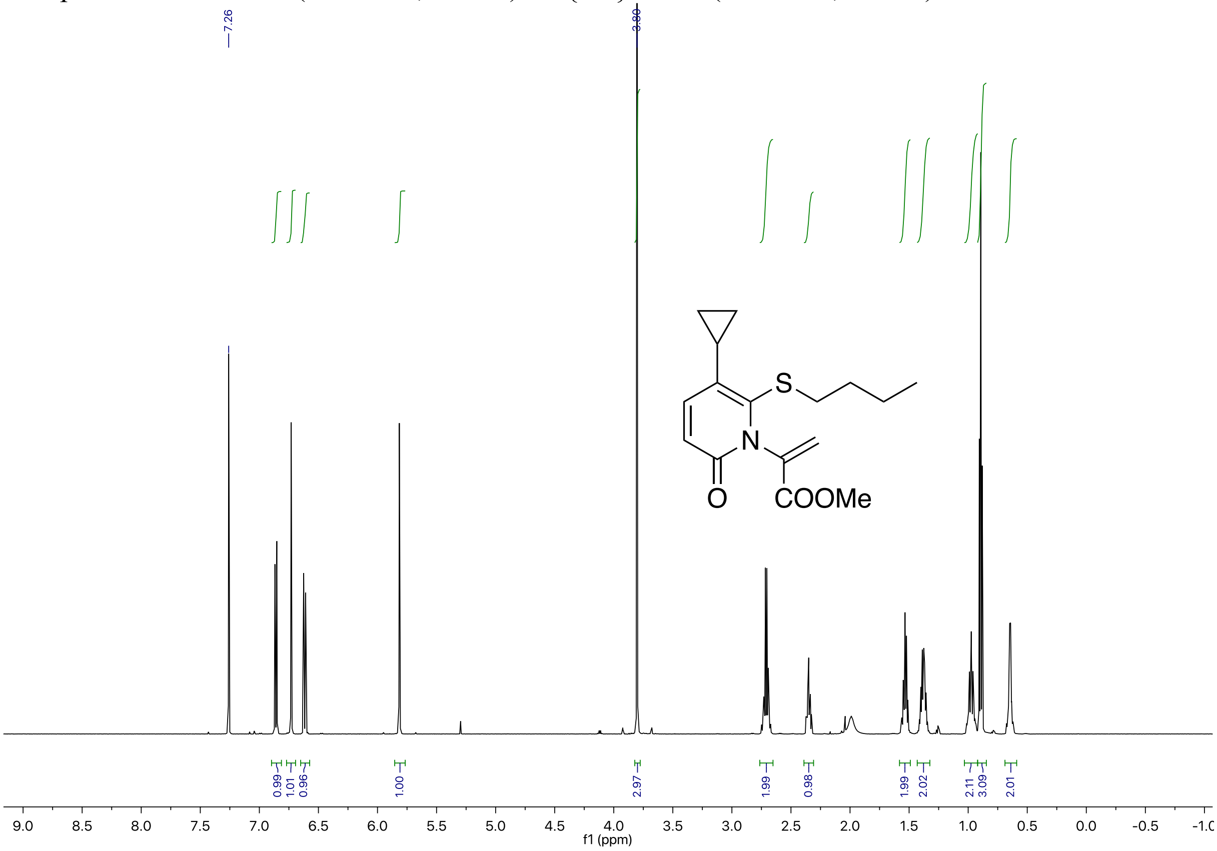

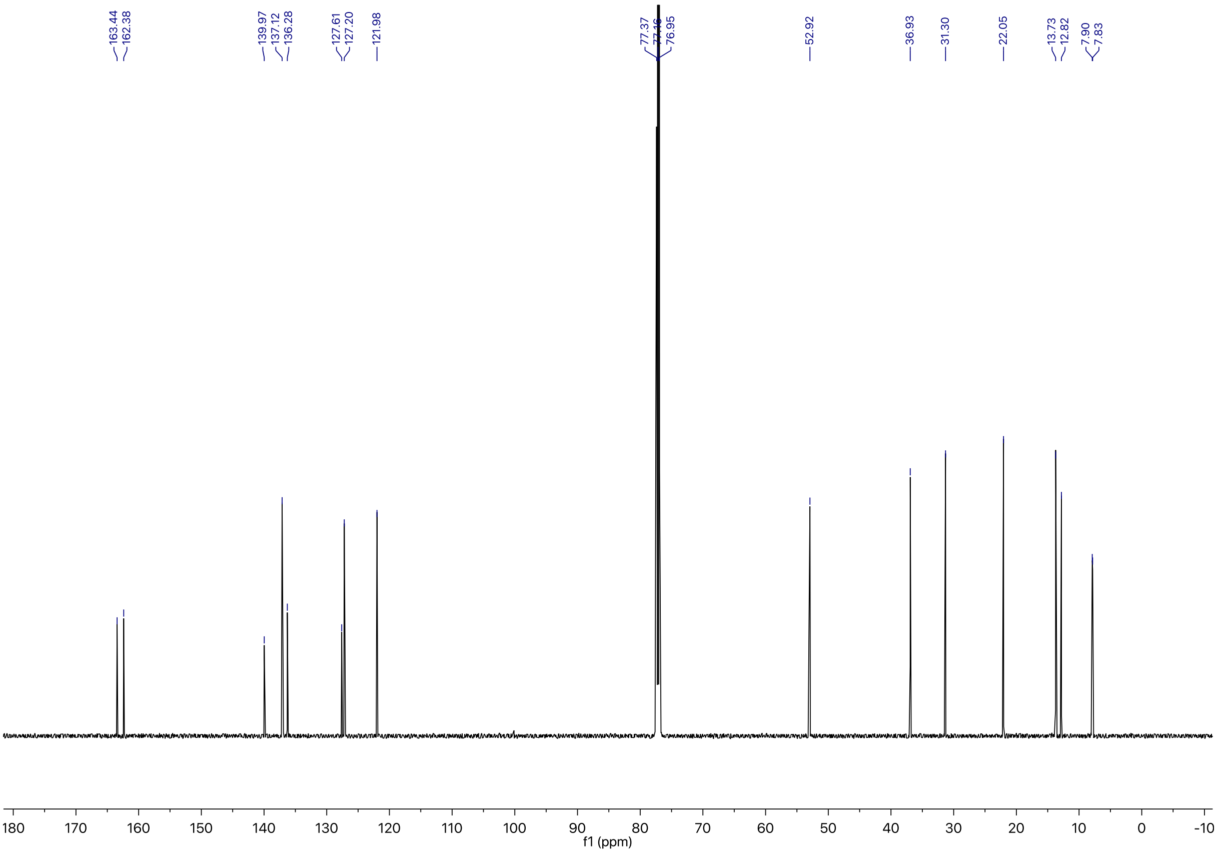

Compound **2f**. ^{1}H NMR (400 MHz, $CDCl_3$) $^{13}C\{^{1}H\}$ NMR (100 MHz, $CDCl_3$)

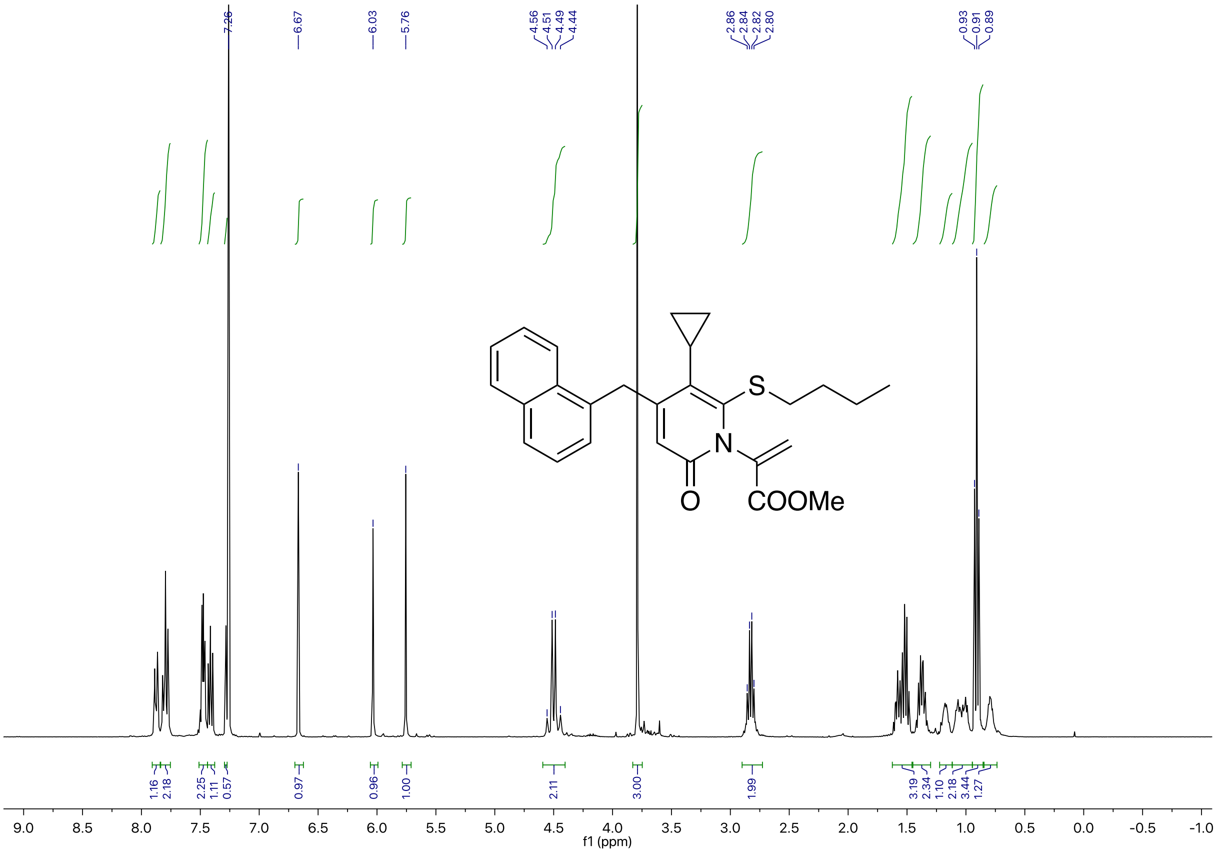

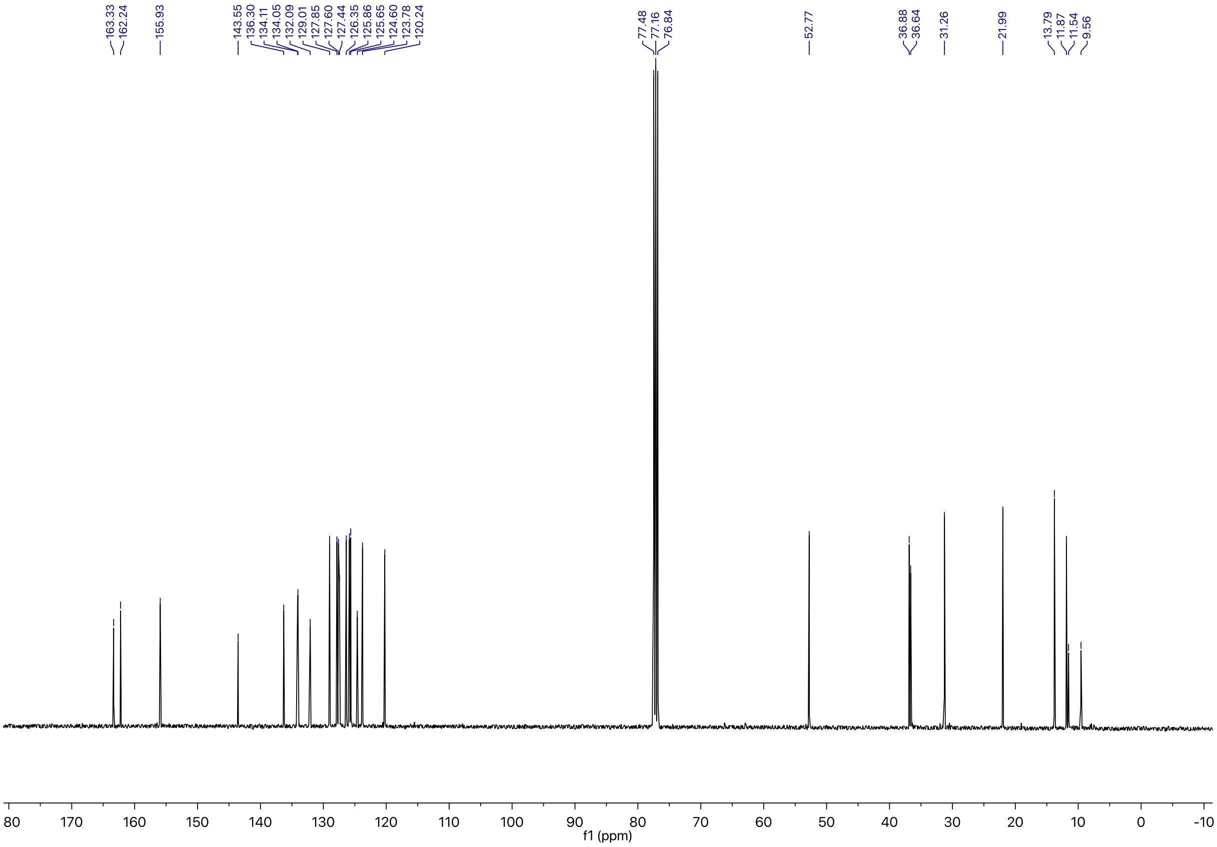

Compound **2g**. ^{1}H NMR (400 MHz, $CDCl_3$) $^{13}C\{^{1}H\}$ NMR (100 MHz, $CDCl_3$)

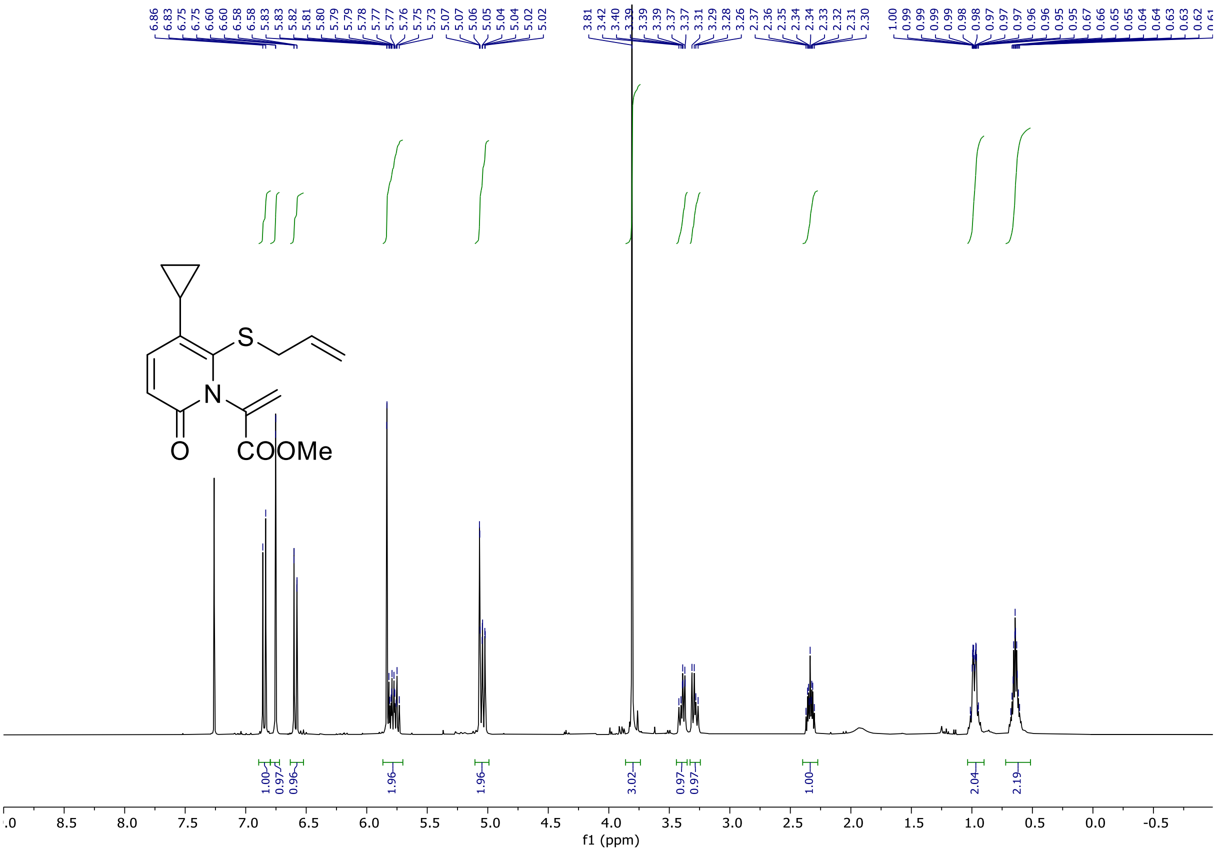

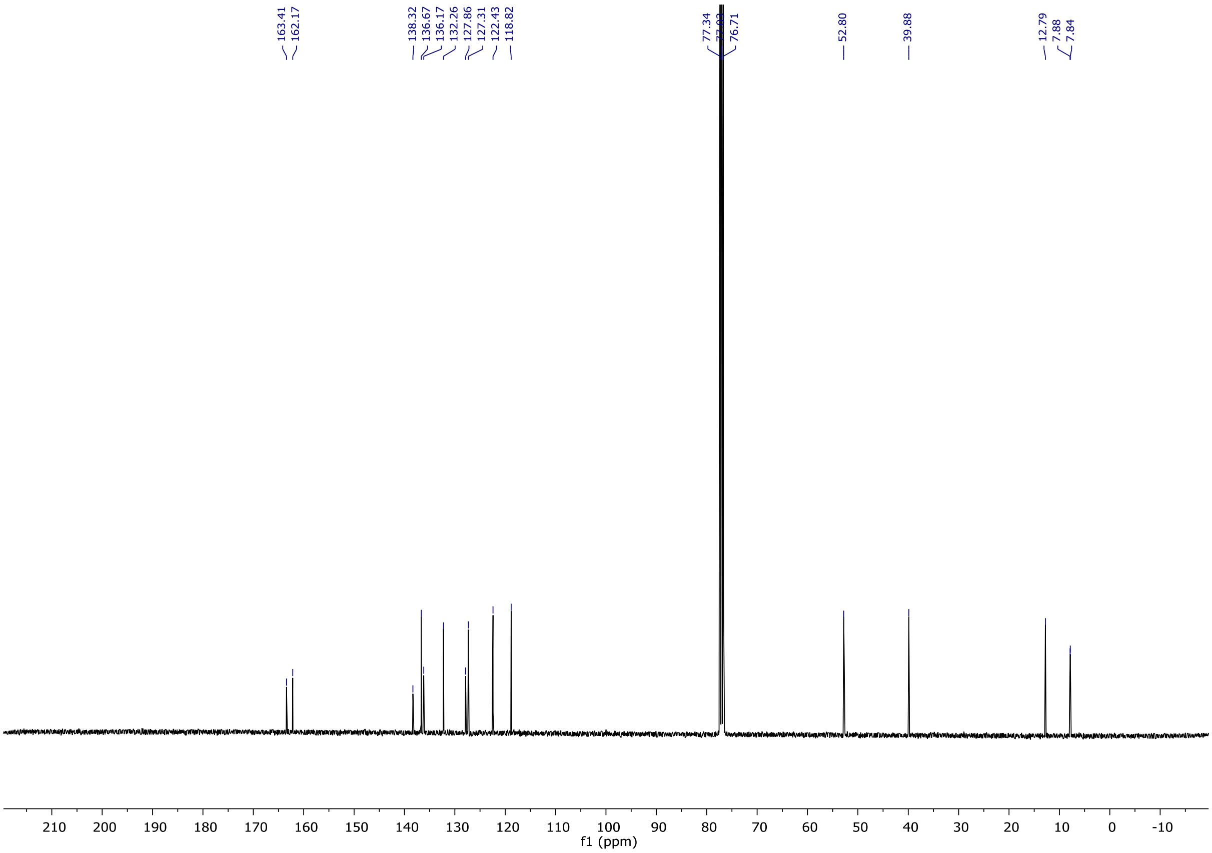

Compound **2h** ^{1}H NMR [400 MHz, $(CD_3)_2SO$] $^{13}C\{^{1}H\}$ NMR [100 MHz, $(CD_3)_2SO$]

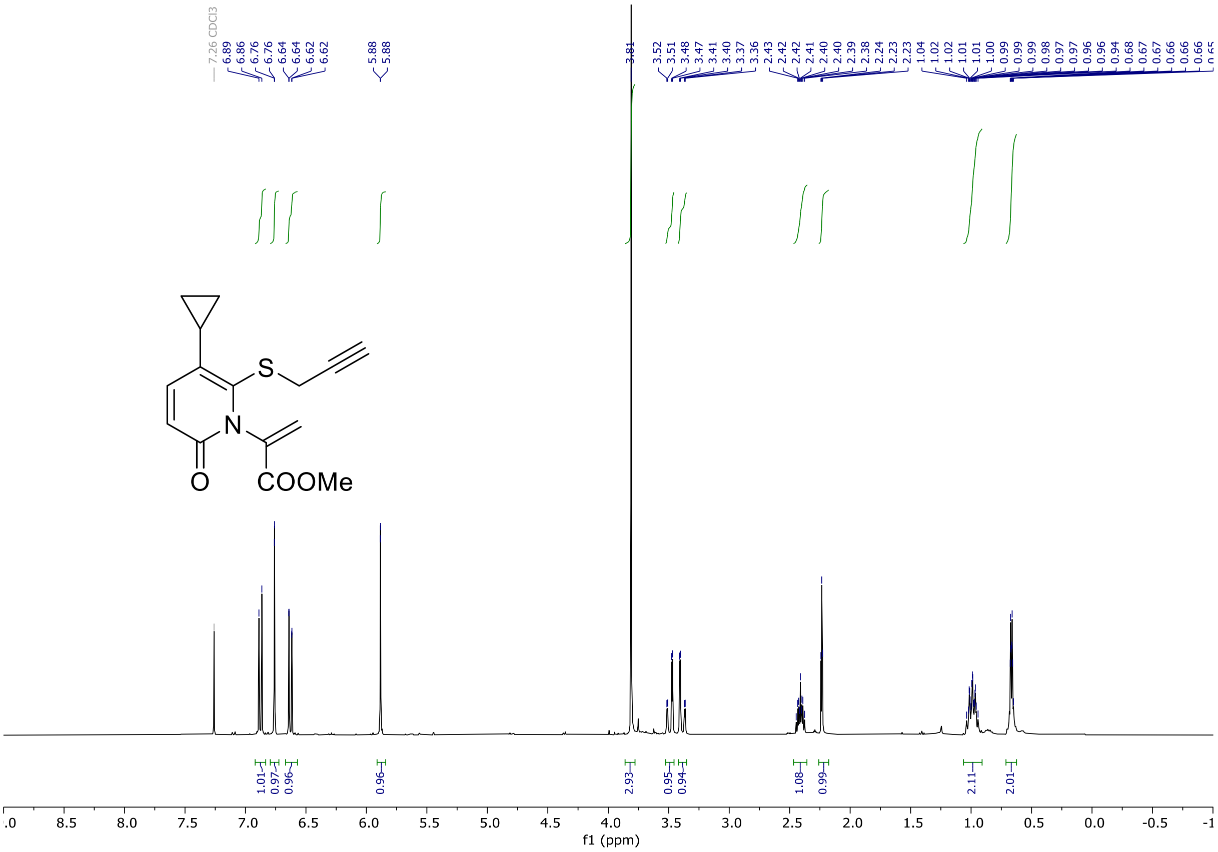

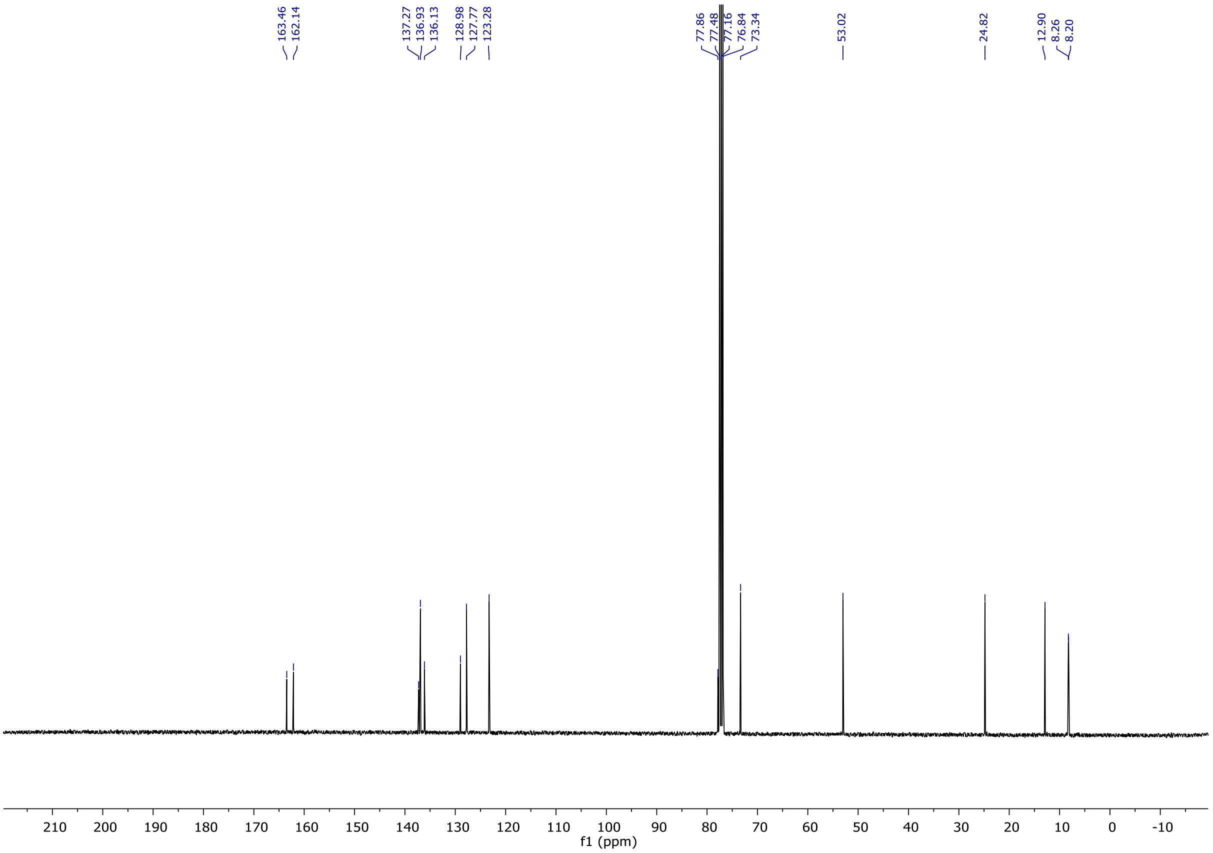

^{1}H-^{1}H COSY [400 MHz, $CDCl_3$]

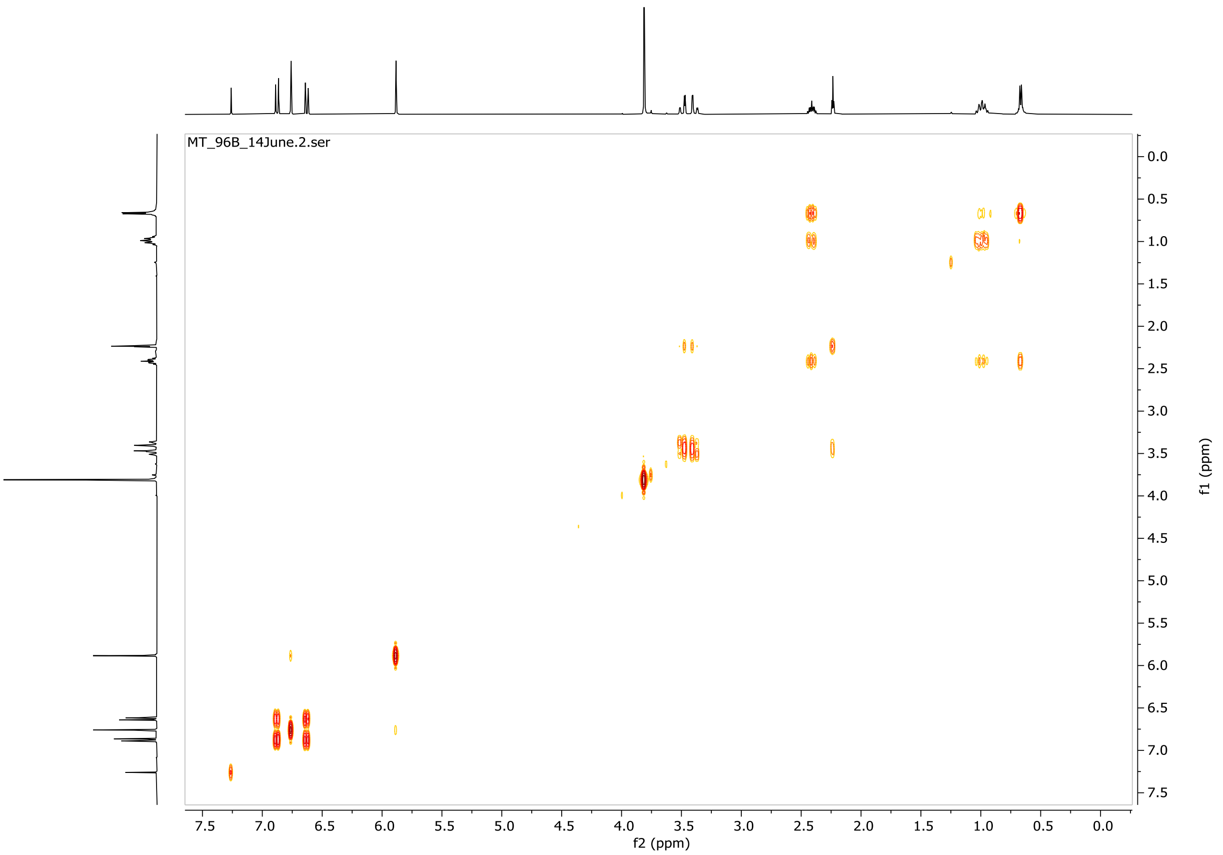

^{1}H-^{13}C HSQC [400 MHz, $CDCl_3$]

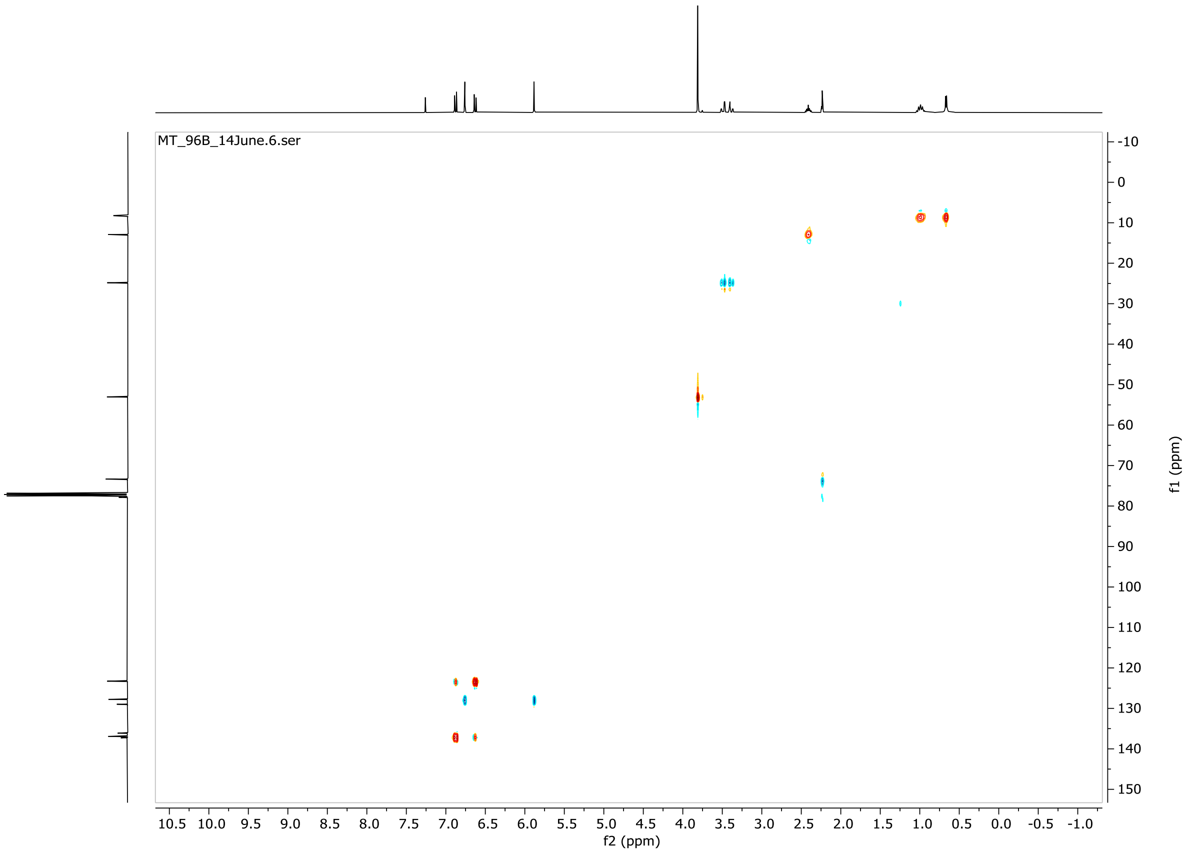

^{1}H-^{13}C HMBC [400 MHz, $CDCl_3$]

^{13}DEPT-135 [400 MHz, $CDCl_3$]

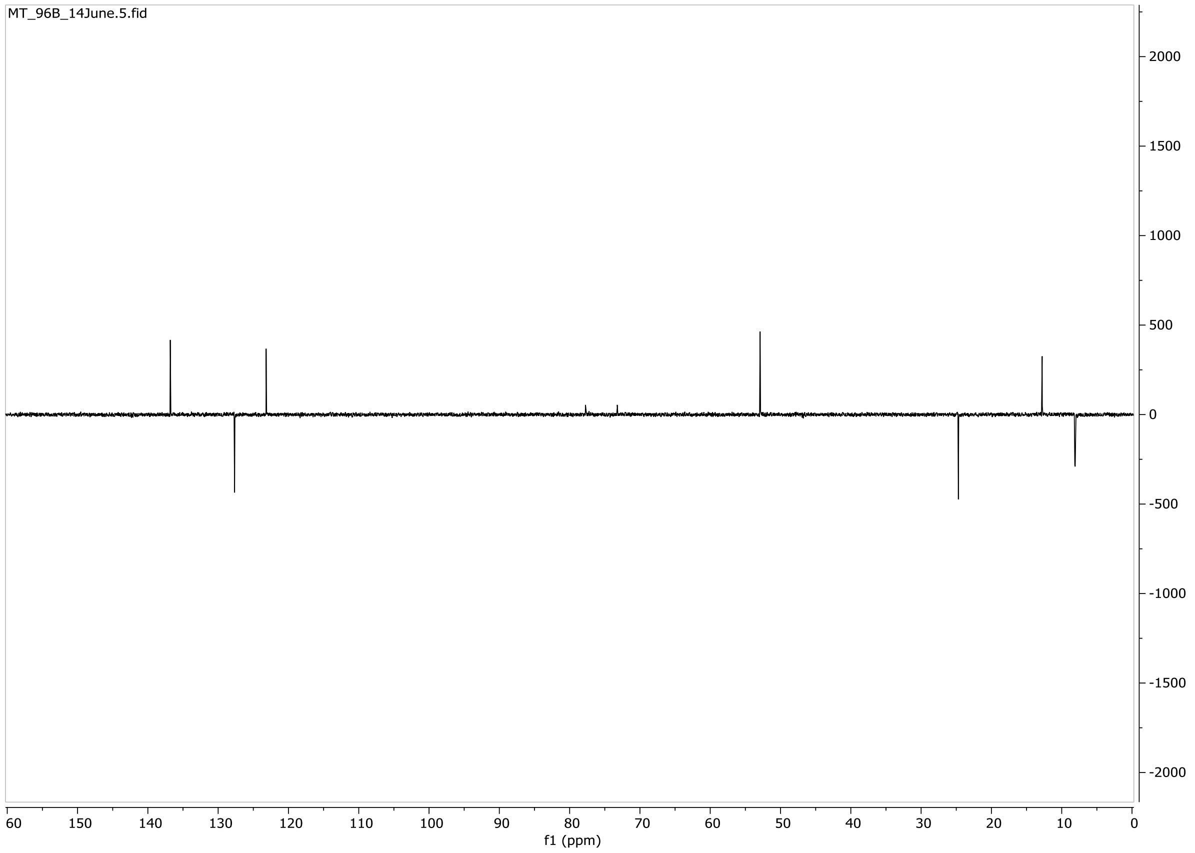

Compound **2i**. ^{1}H NMR [400 MHz, $(CD_3)_2SO$] $^{13}C\{^{1}H\}$ NMR [100 MHz, $(CD_3)_2SO$]

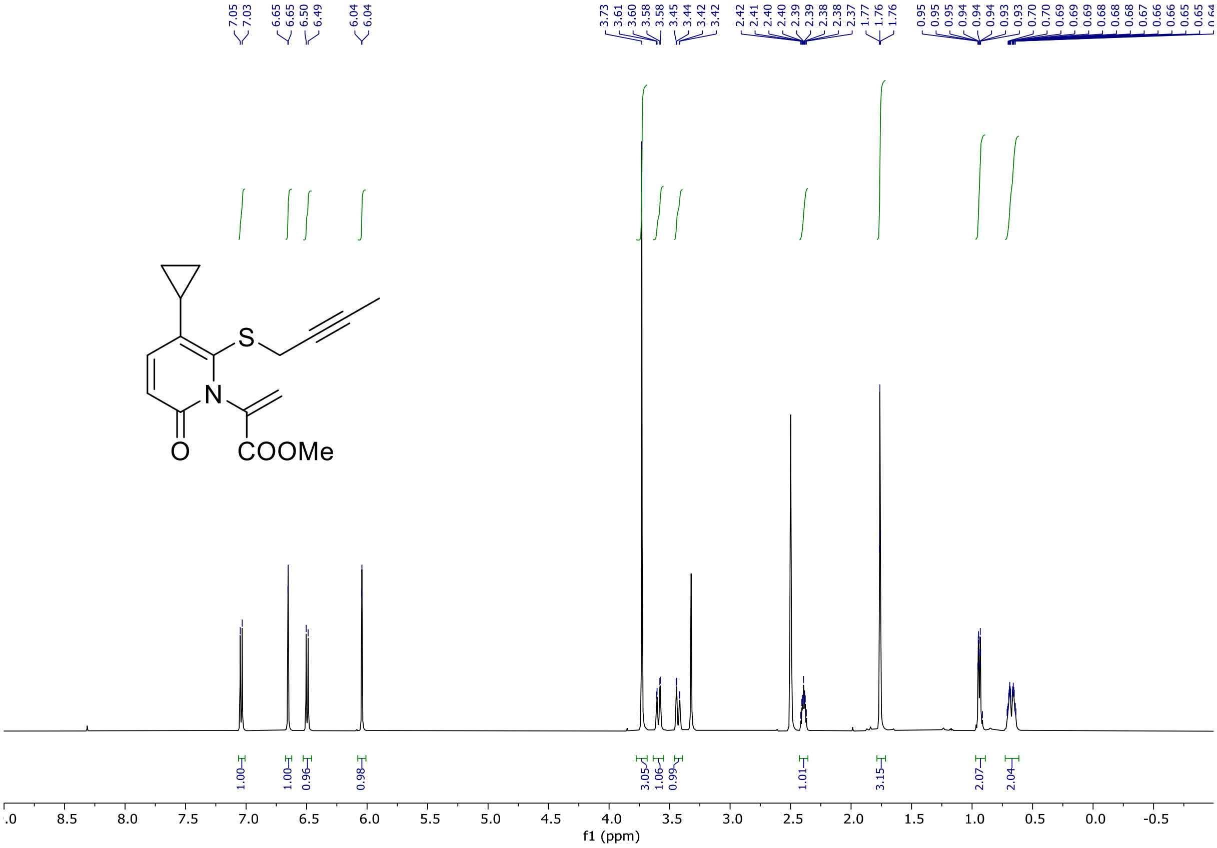

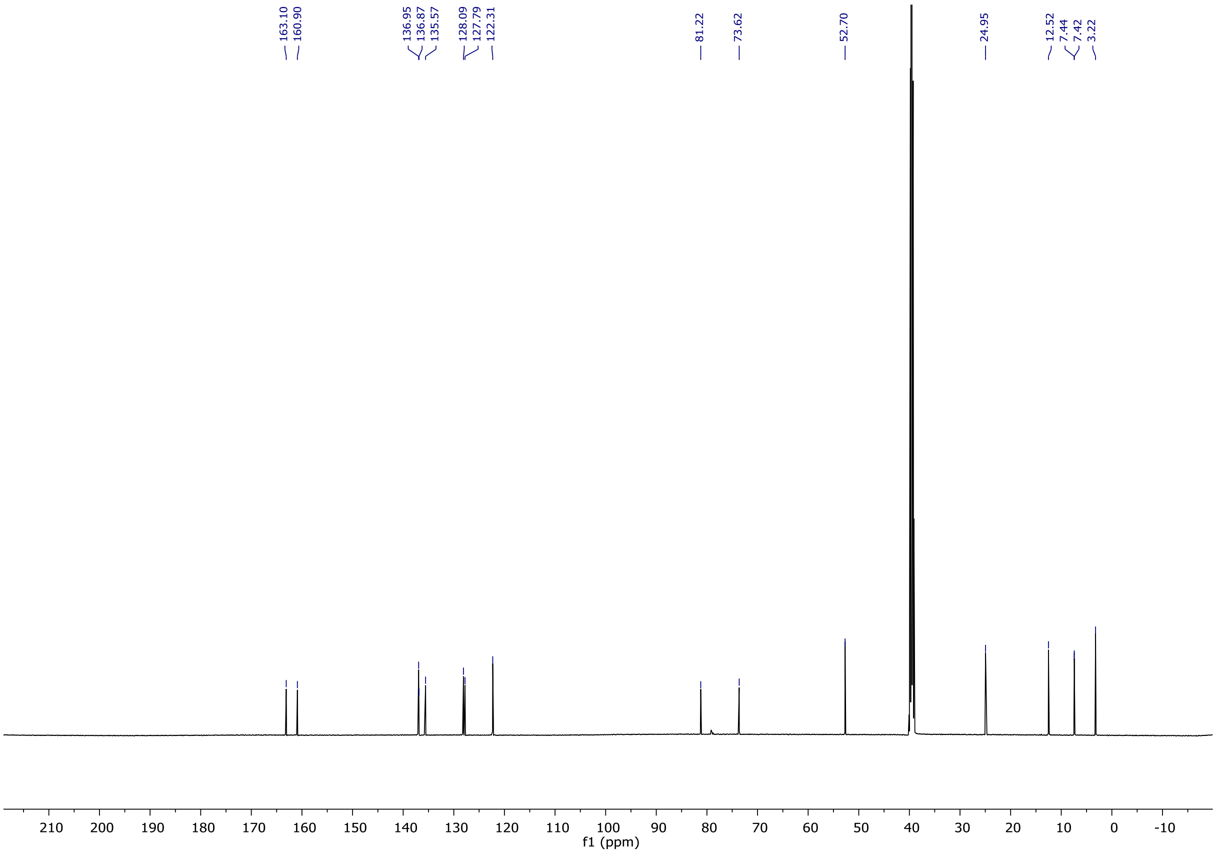

Compound **5a** ^{1}H NMR (400 MHz, $CDCl_3$) $^{13}C\{^{1}H\}$ NMR (100 MHz, $CDCl_3$)

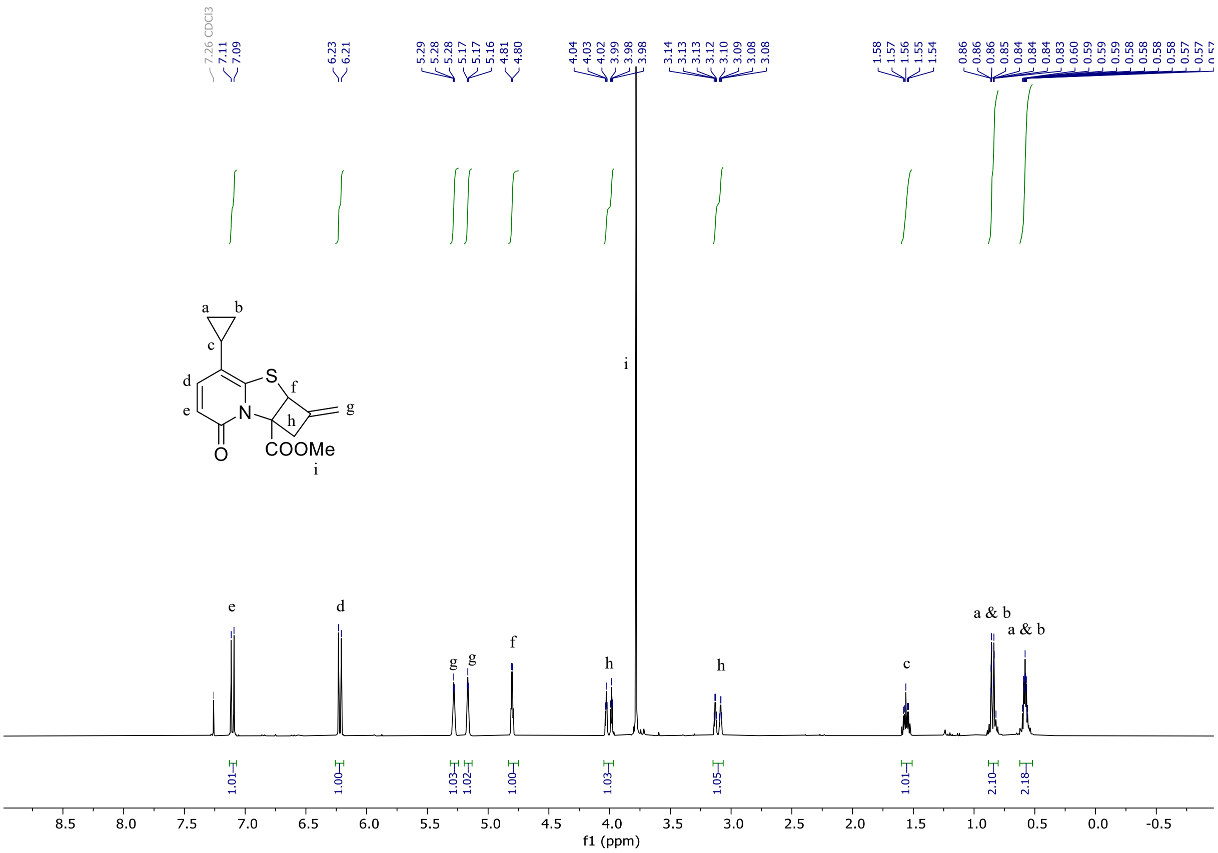

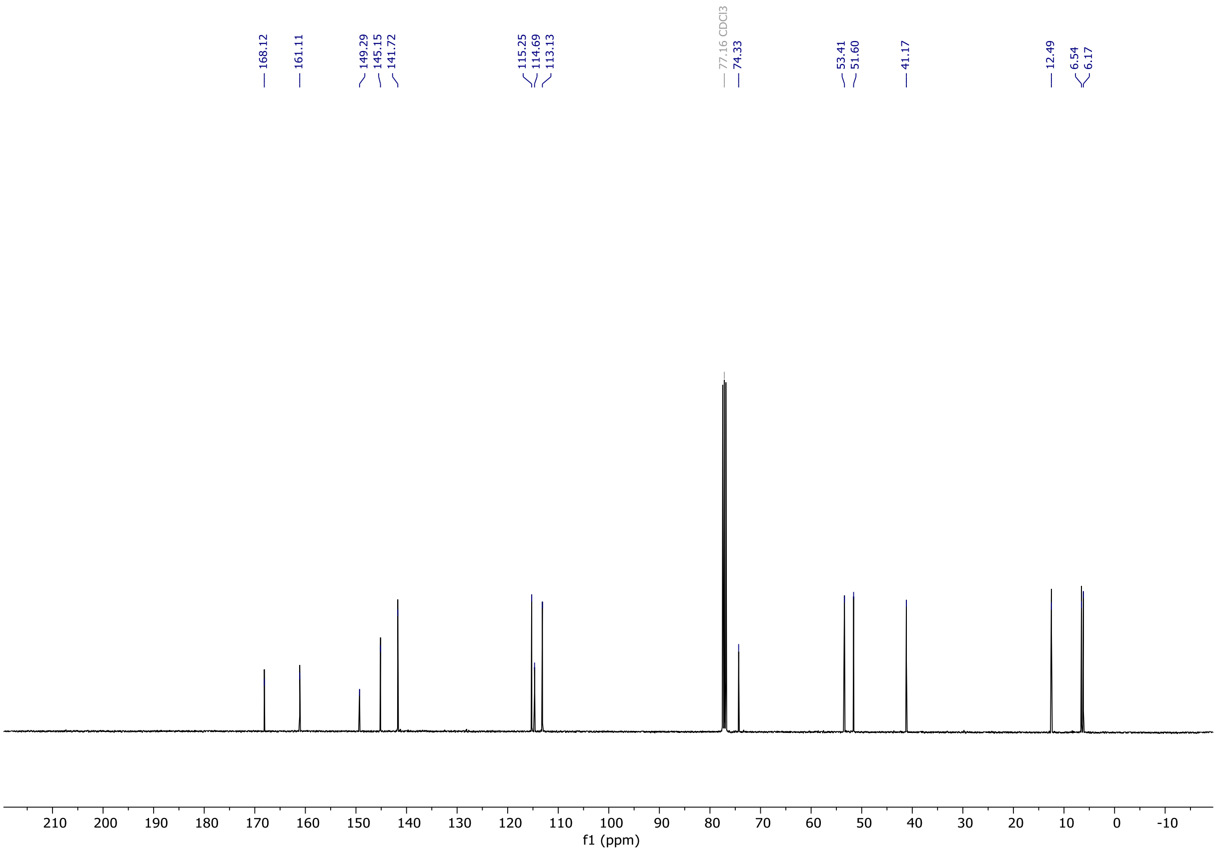

^{1}H-^{1}H COSY [400 MHz, $CDCl_3$]

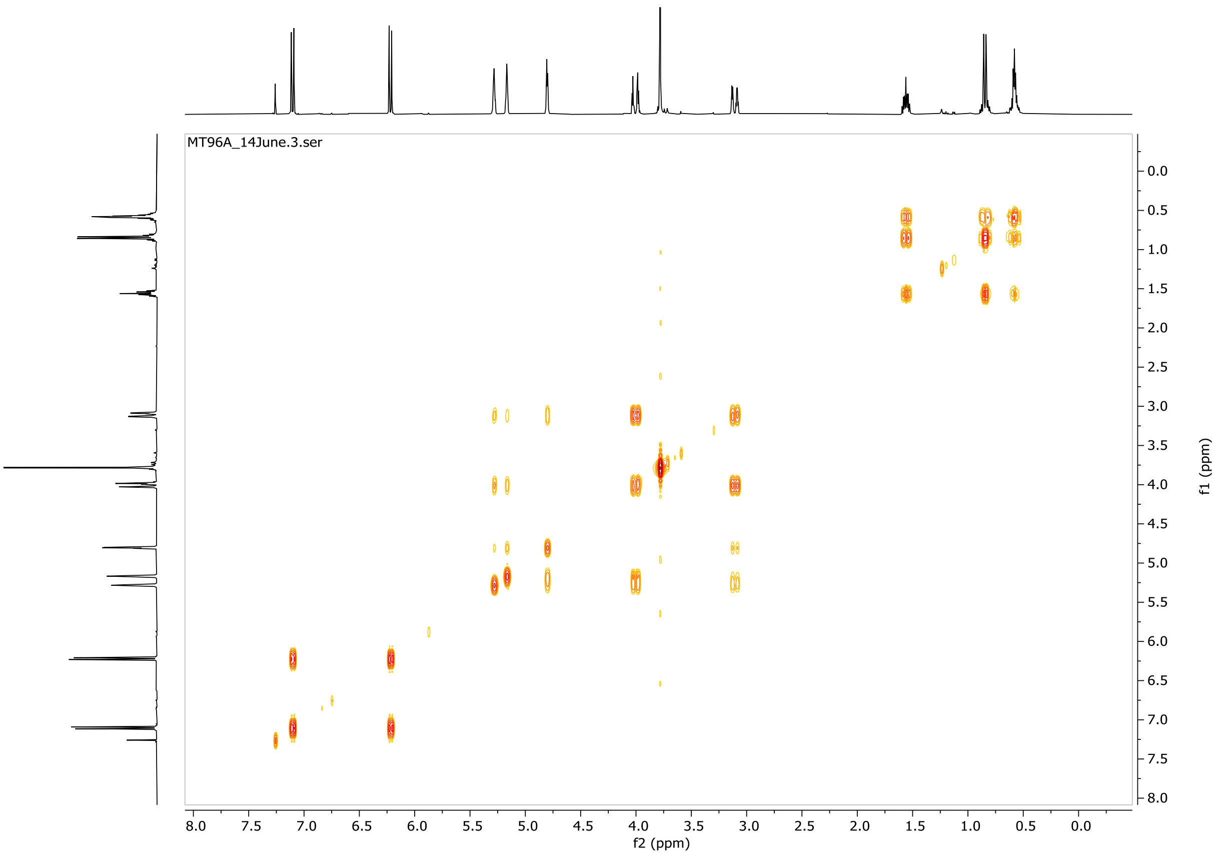

^{1}H-^{13}C HSQC [400 MHz, $CDCl_3$]

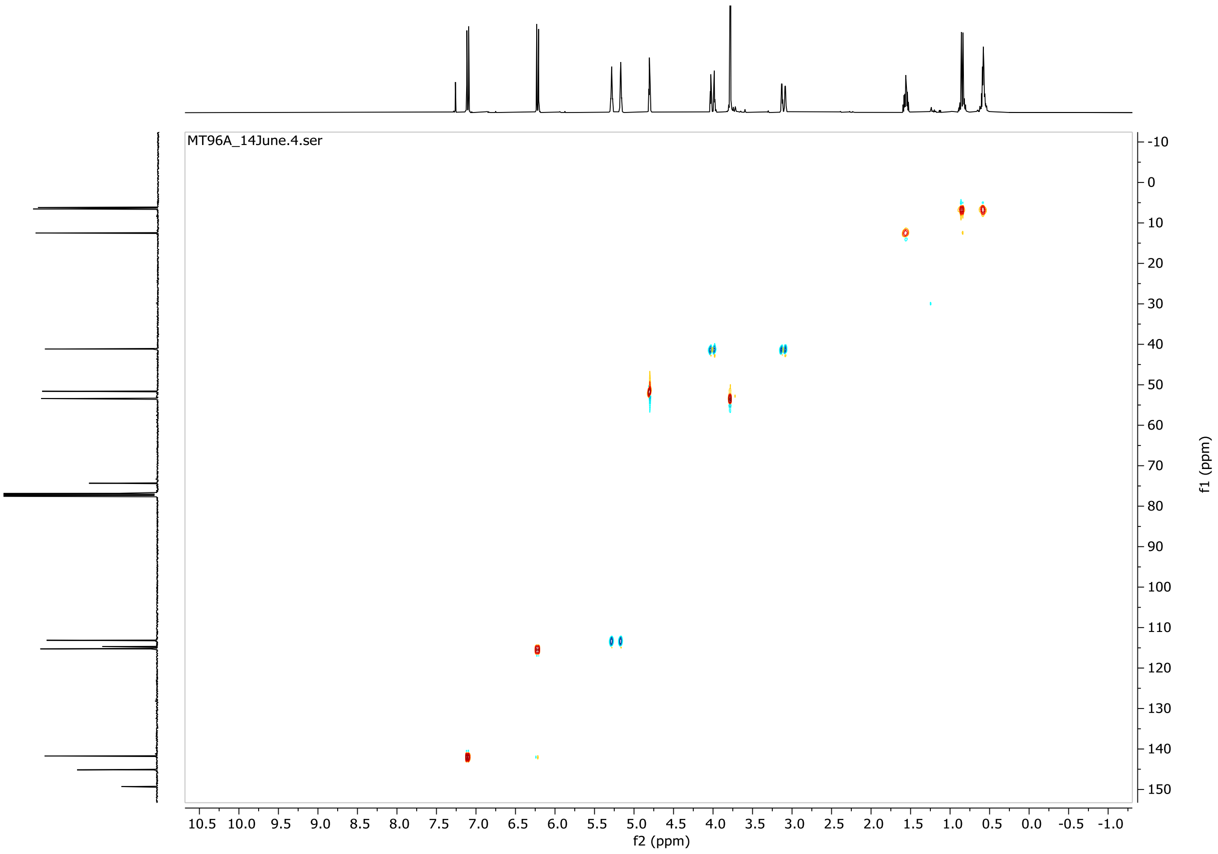

^{1}H-^{13}C HMBC [400 MHz, CDCl$_3$]

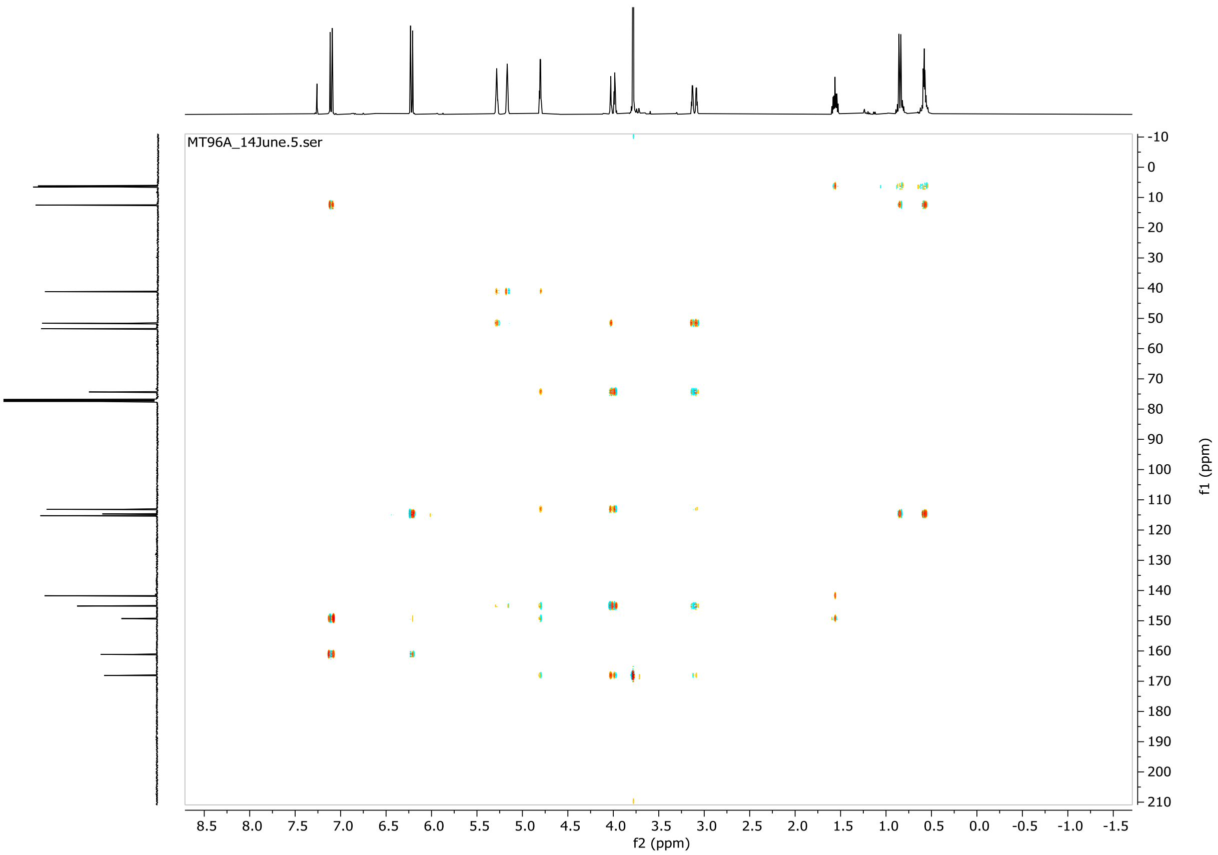

^{13}DEPT-135 [400 MHz, CDCl$_3$]

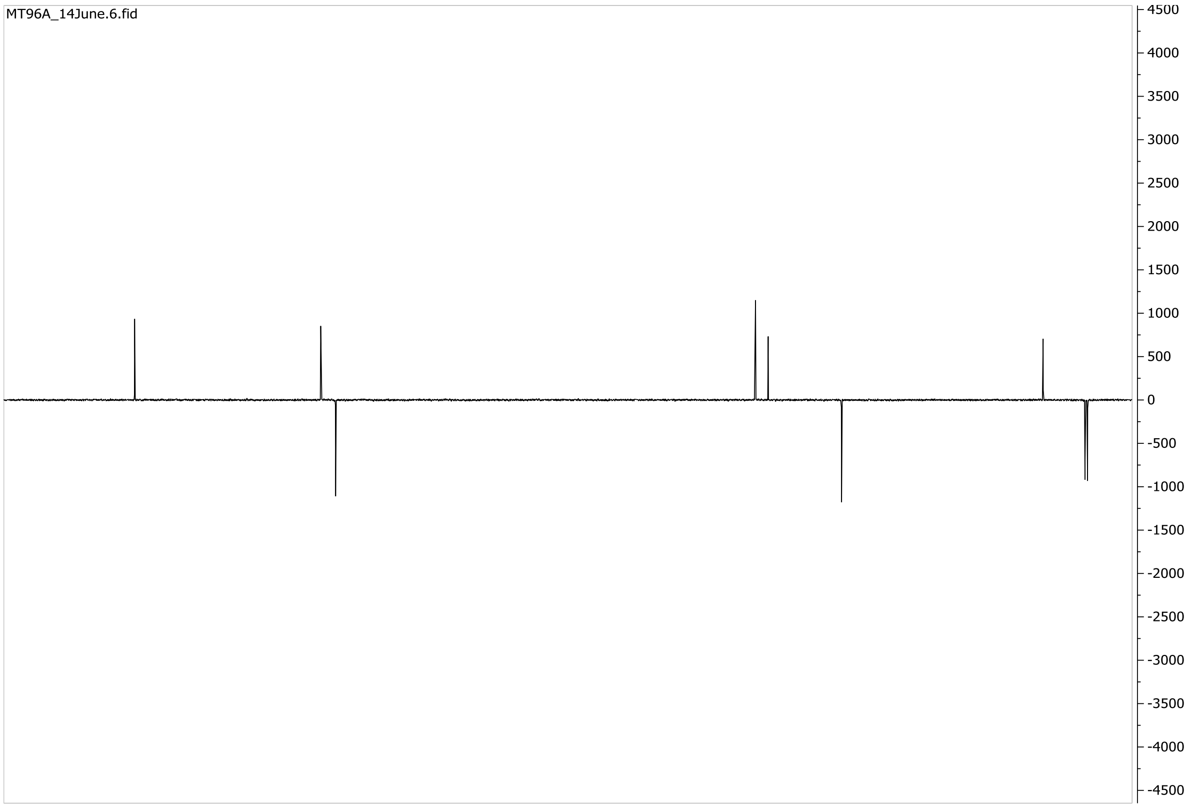

Compound **5b** ^{1}H NMR [400 MHz, $(CD_3)_2SO$] $^{13}C\{^1H\}$ NMR [100 MHz, $(CD_3)_2SO$]

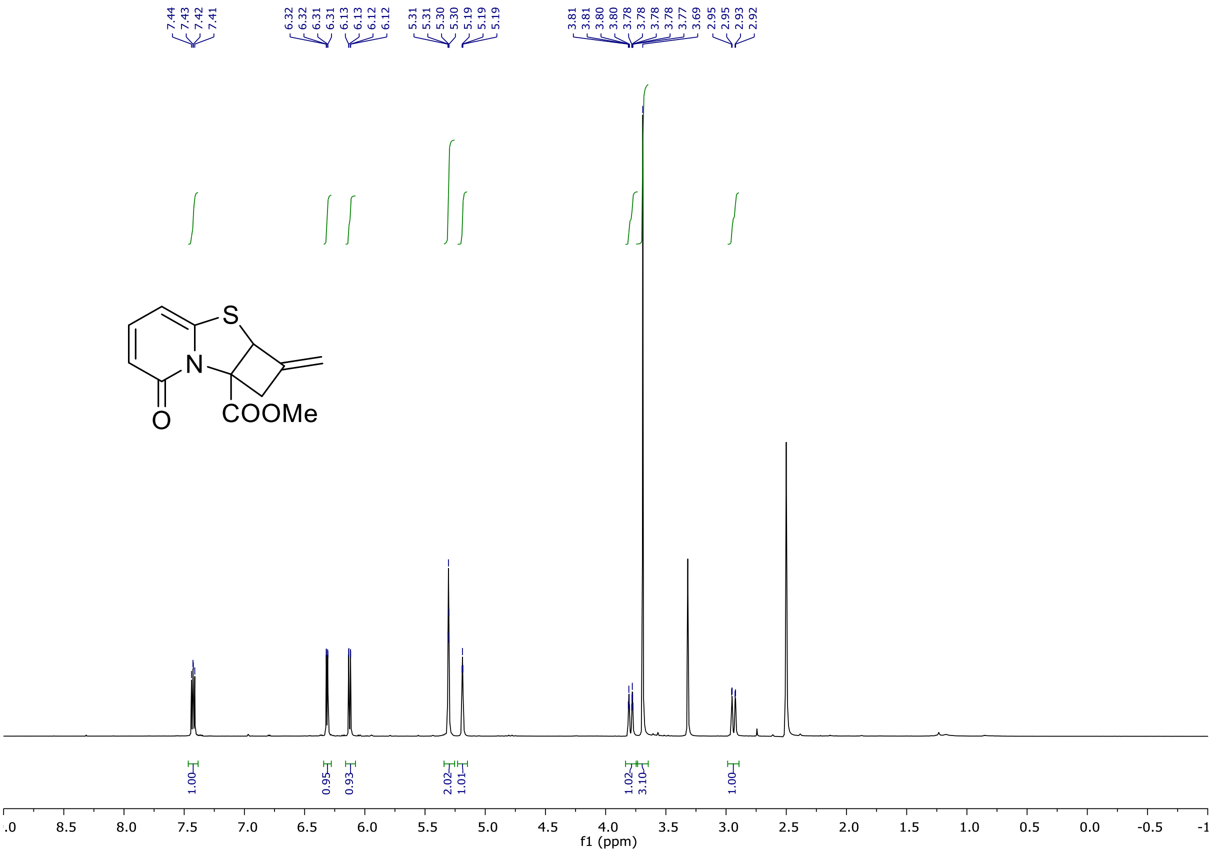

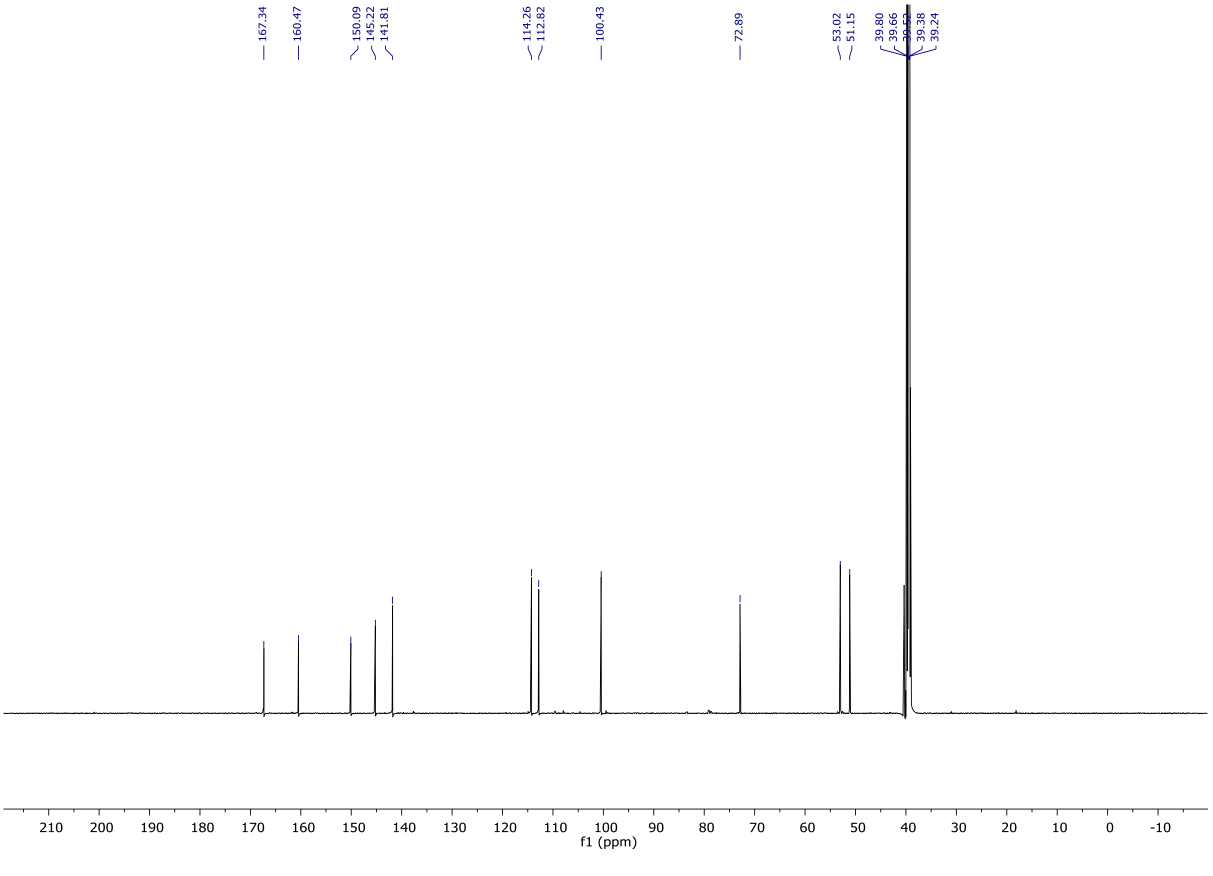

Compound **5c** ^{1}H NMR [400 MHz, $(CD_3)_2SO$] $^{13}C\{^{1}H\}$ NMR [100 MHz, $(CD_3)_2SO$]

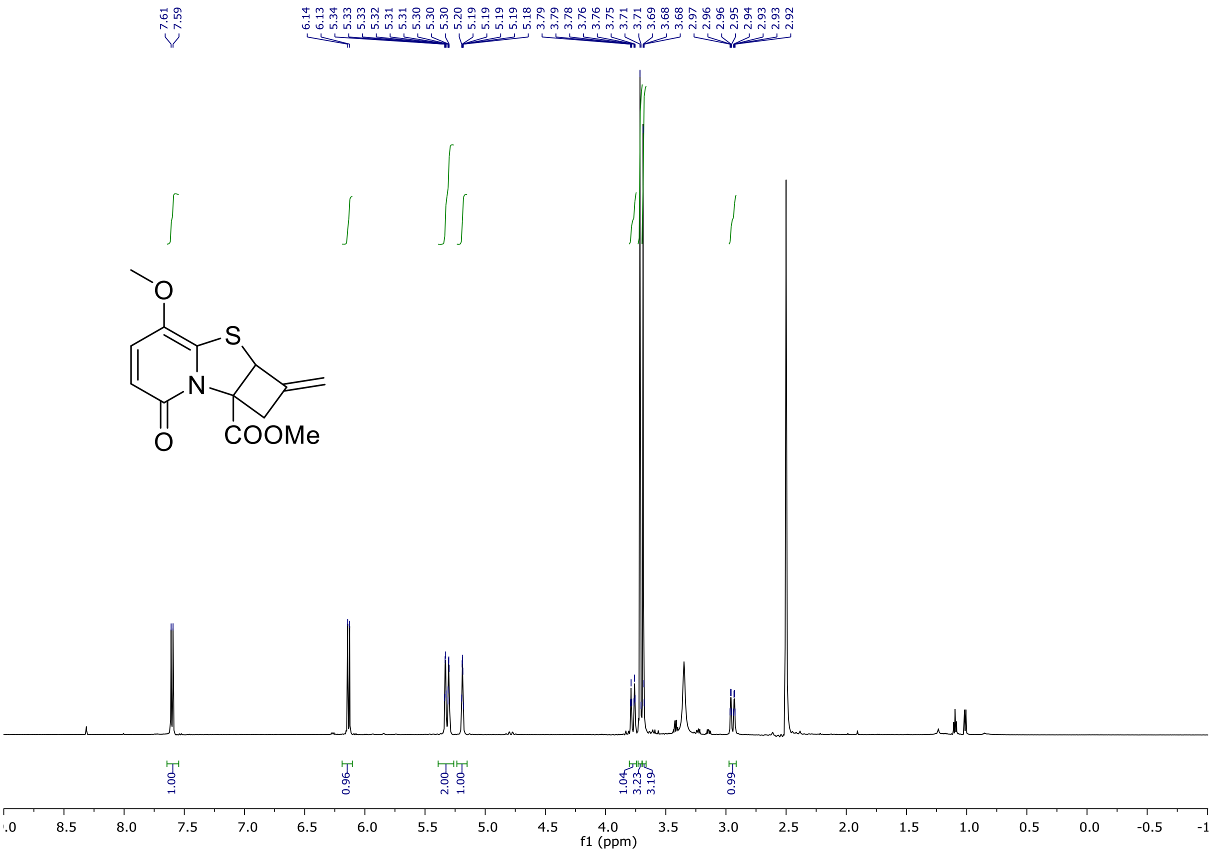

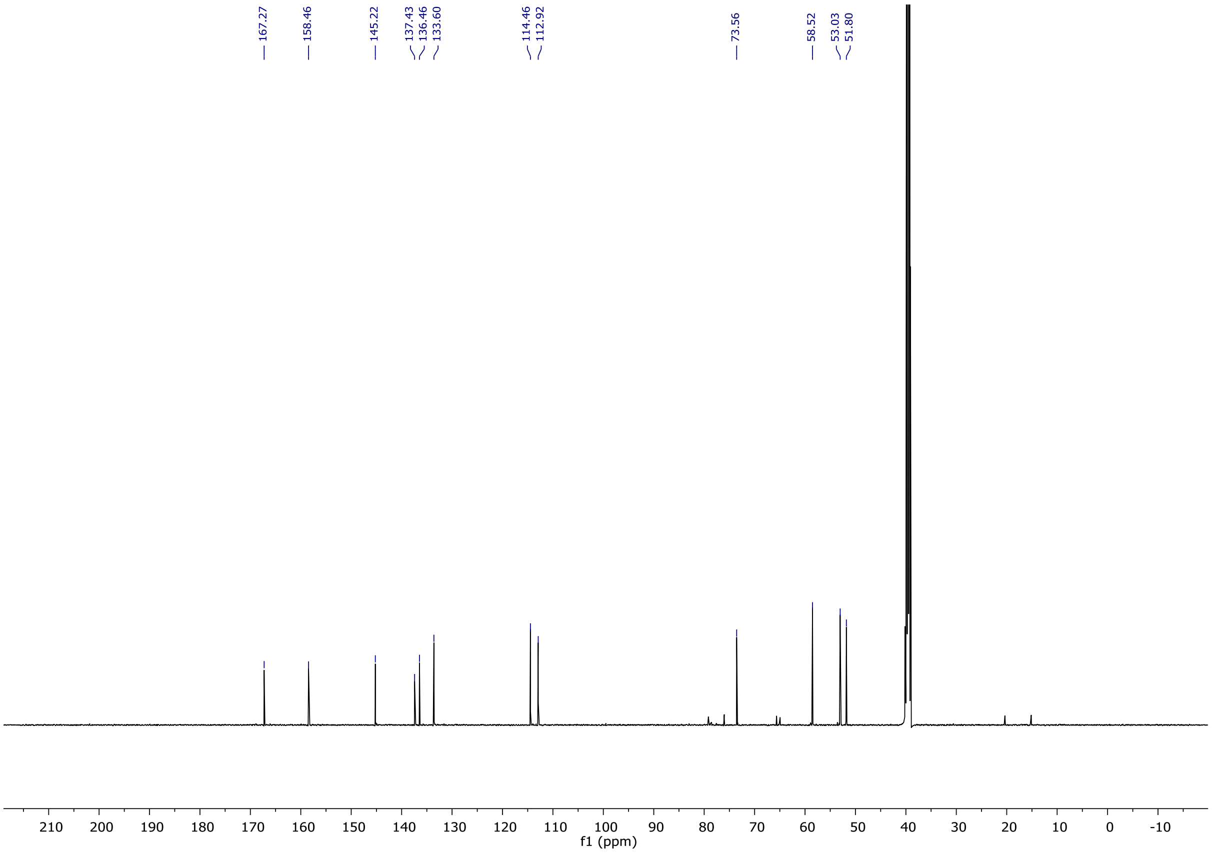

Compound **5d** ^{1}H NMR [400 MHz, $(CD_3)_2SO$] $^{13}C\{^{1}H\}$ NMR [100 MHz, $(CD_3)_2SO$]

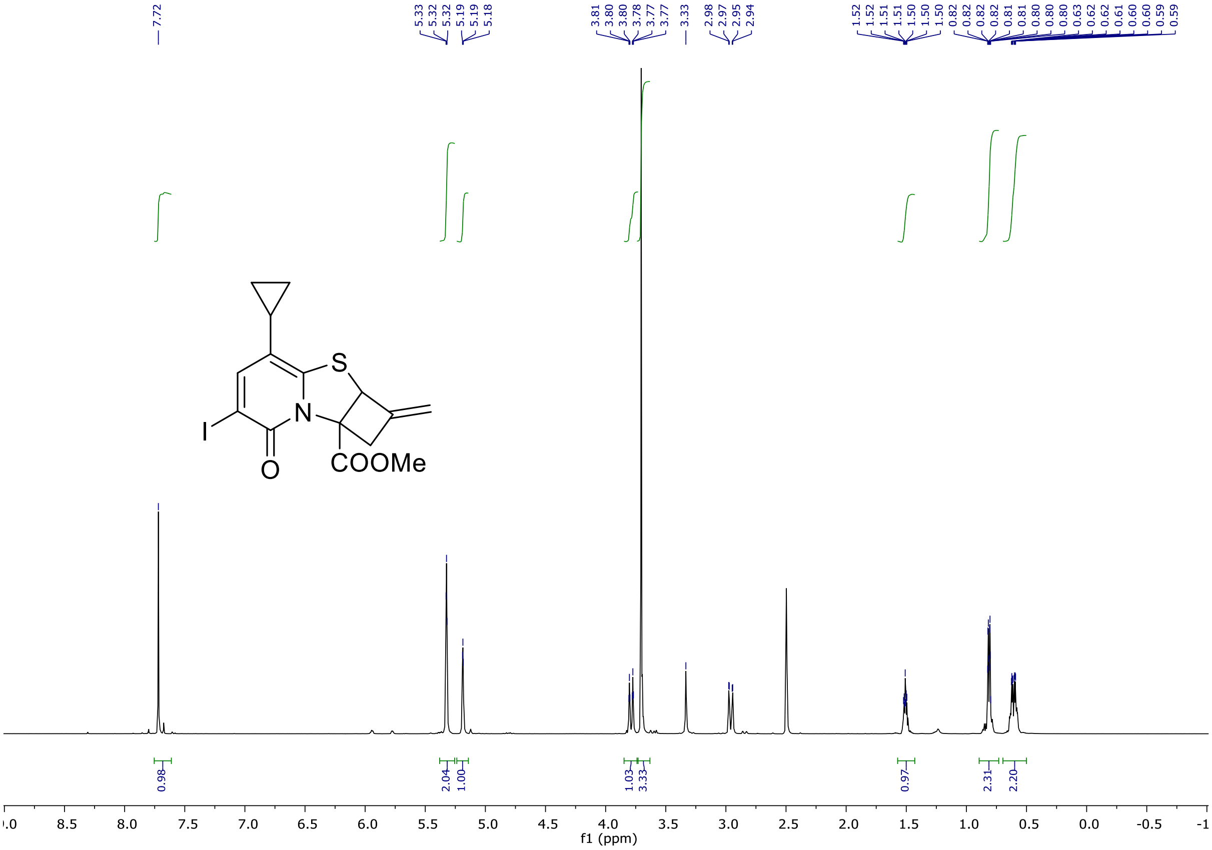

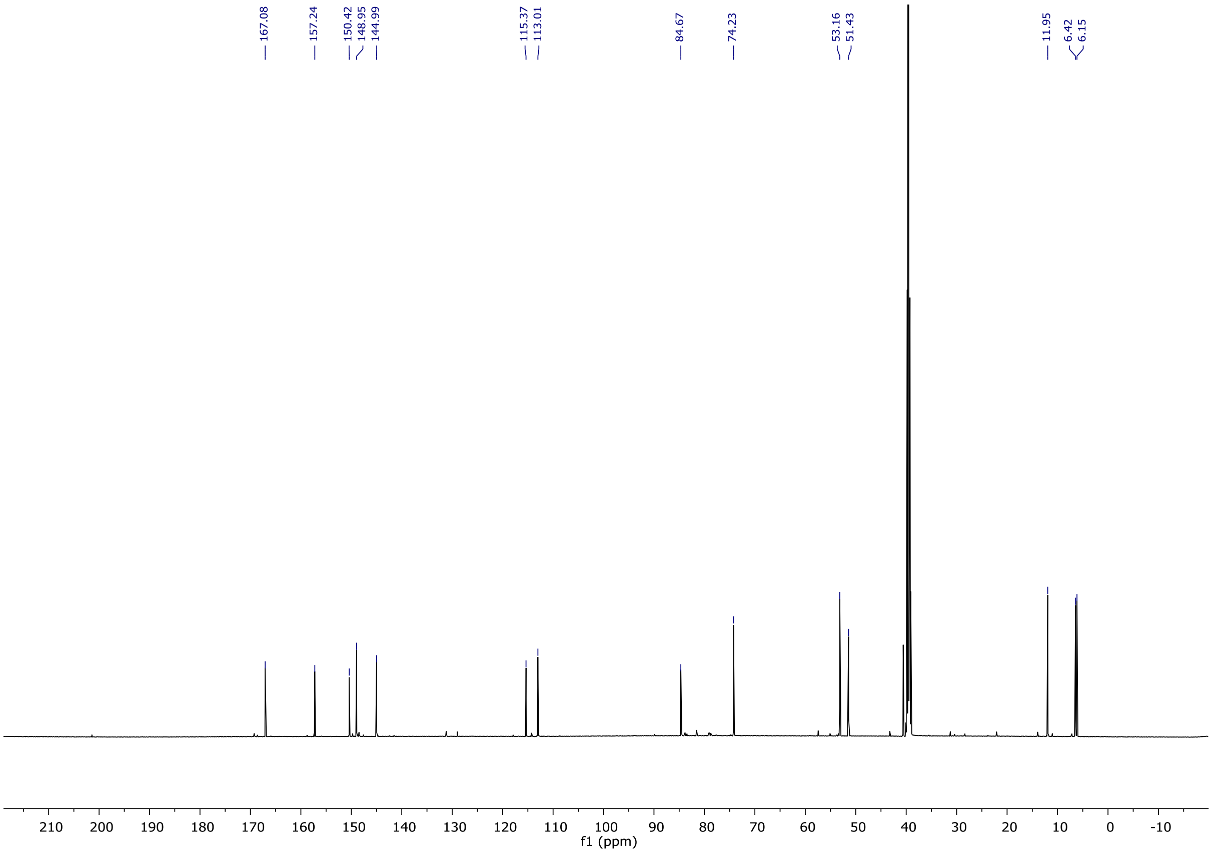

Compound **5e** ^{1}H NMR [400 MHz, $(CD_3)_2SO$] $^{13}C\{^{1}H\}$ NMR [100 MHz, $(CD_3)_2SO$]

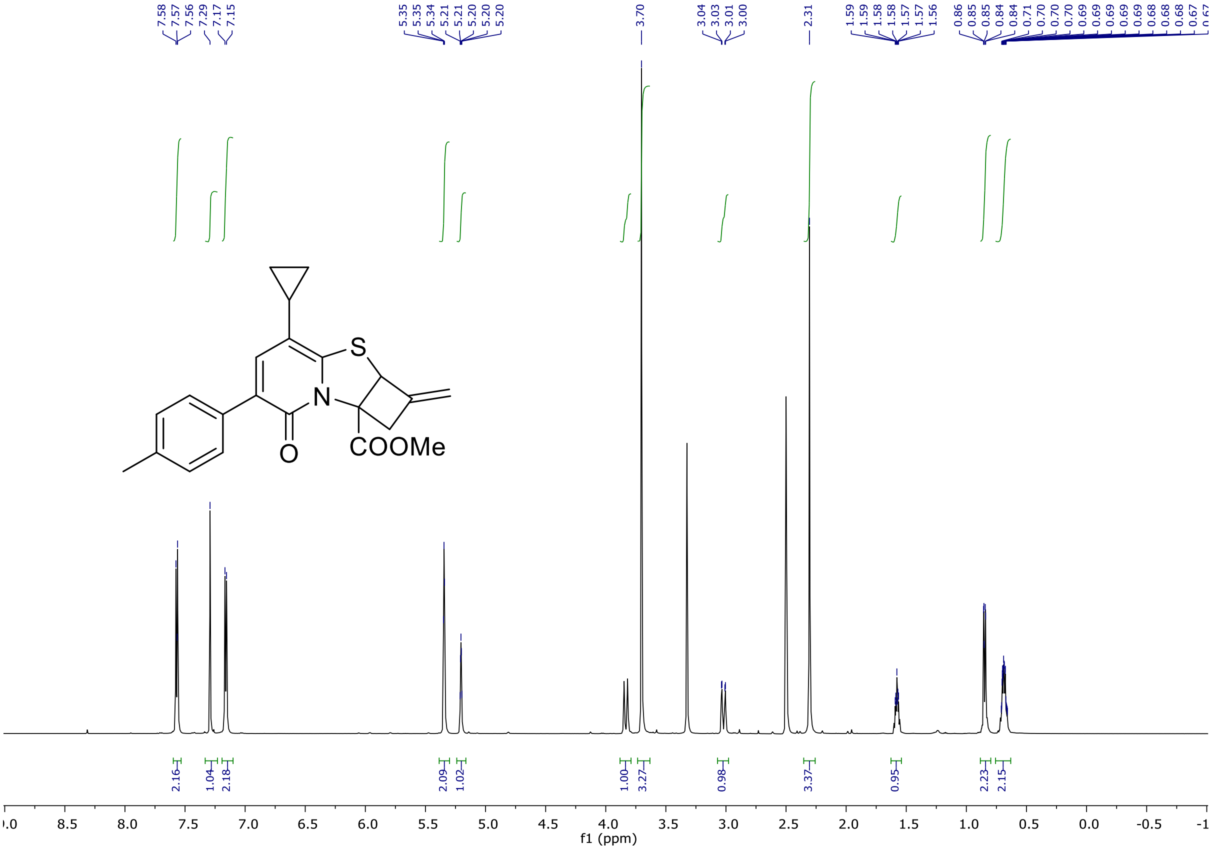

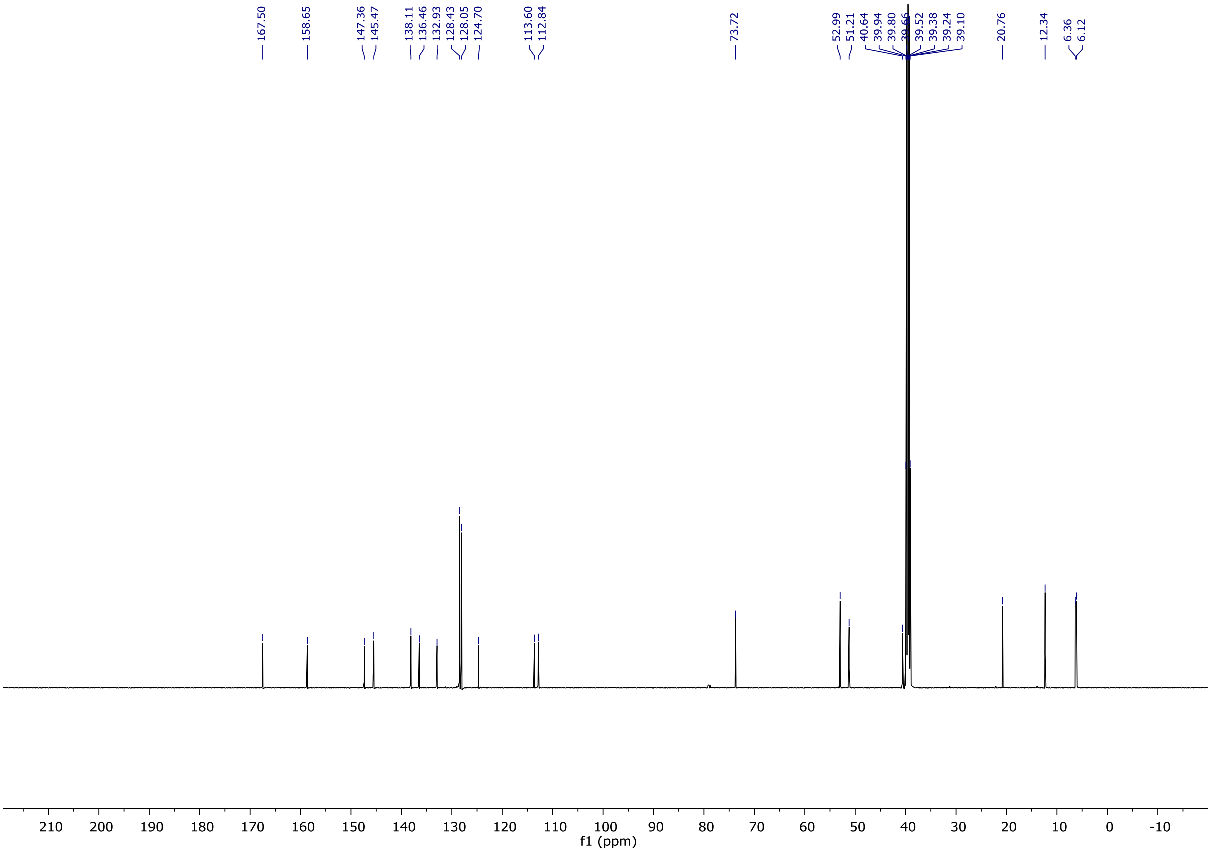

Compound **5f** ^{1}H NMR [400 MHz, $(CD_3)_2SO$] $^{13}C\{^{1}H\}$ NMR [100 MHz, $(CD_3)_2SO$]

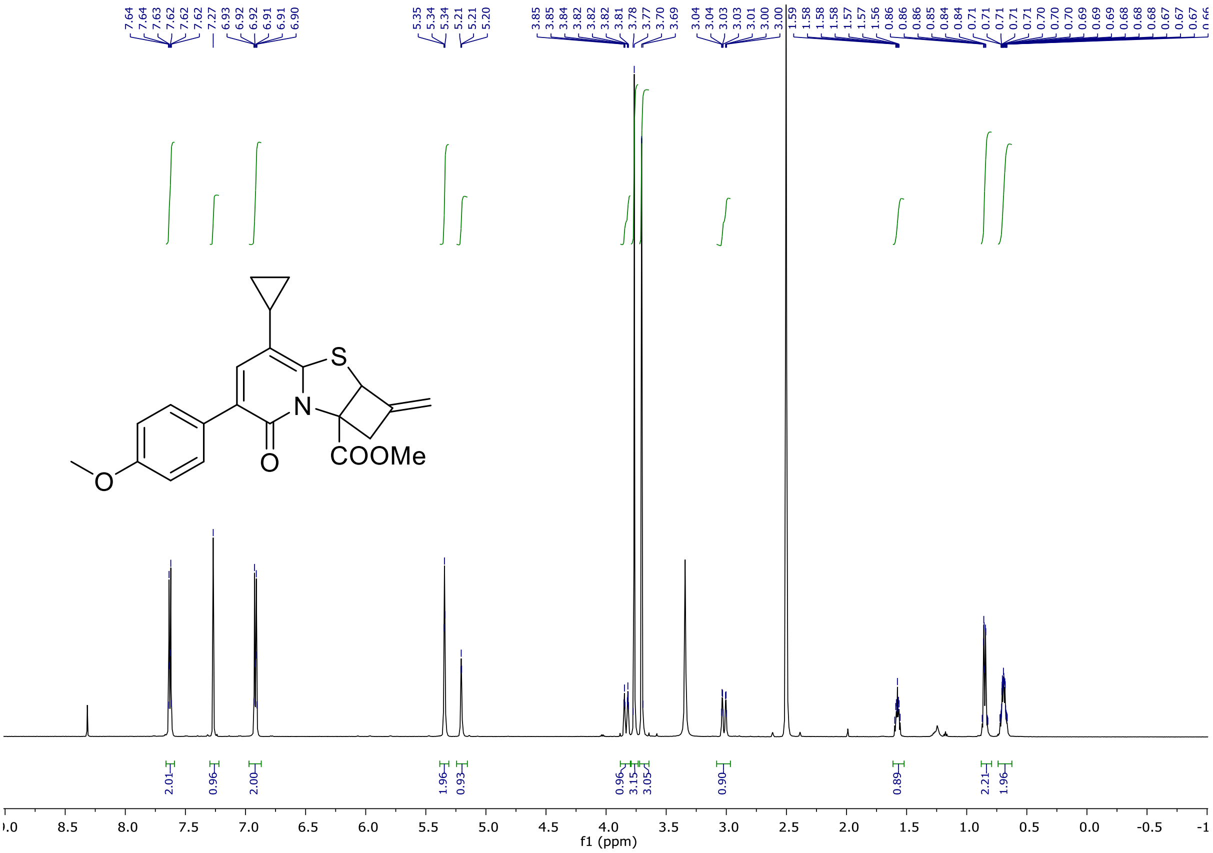

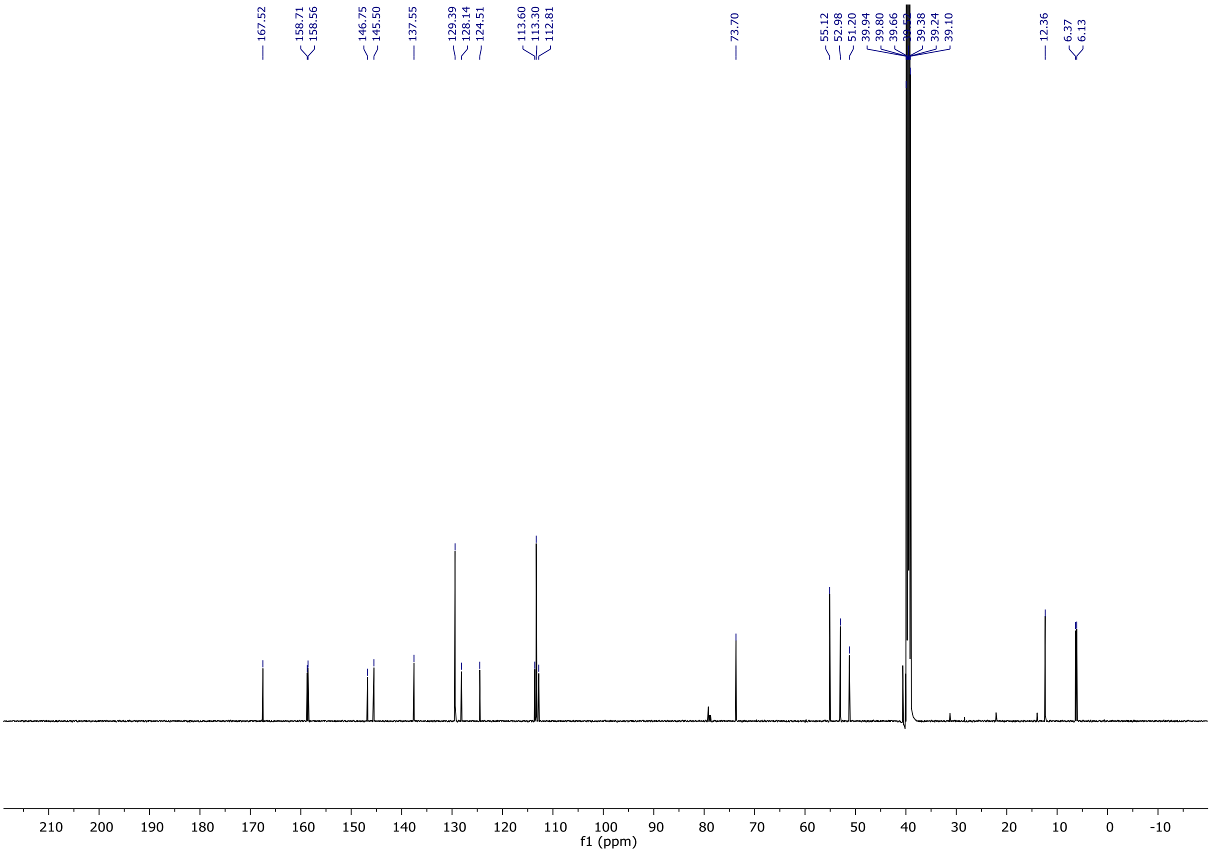

Compound **5g** ^{1}H NMR [400 MHz, $(CD_3)_2SO$] ^{13}C{^{1}H} NMR [100 MHz, $(CD_3)_2SO$]

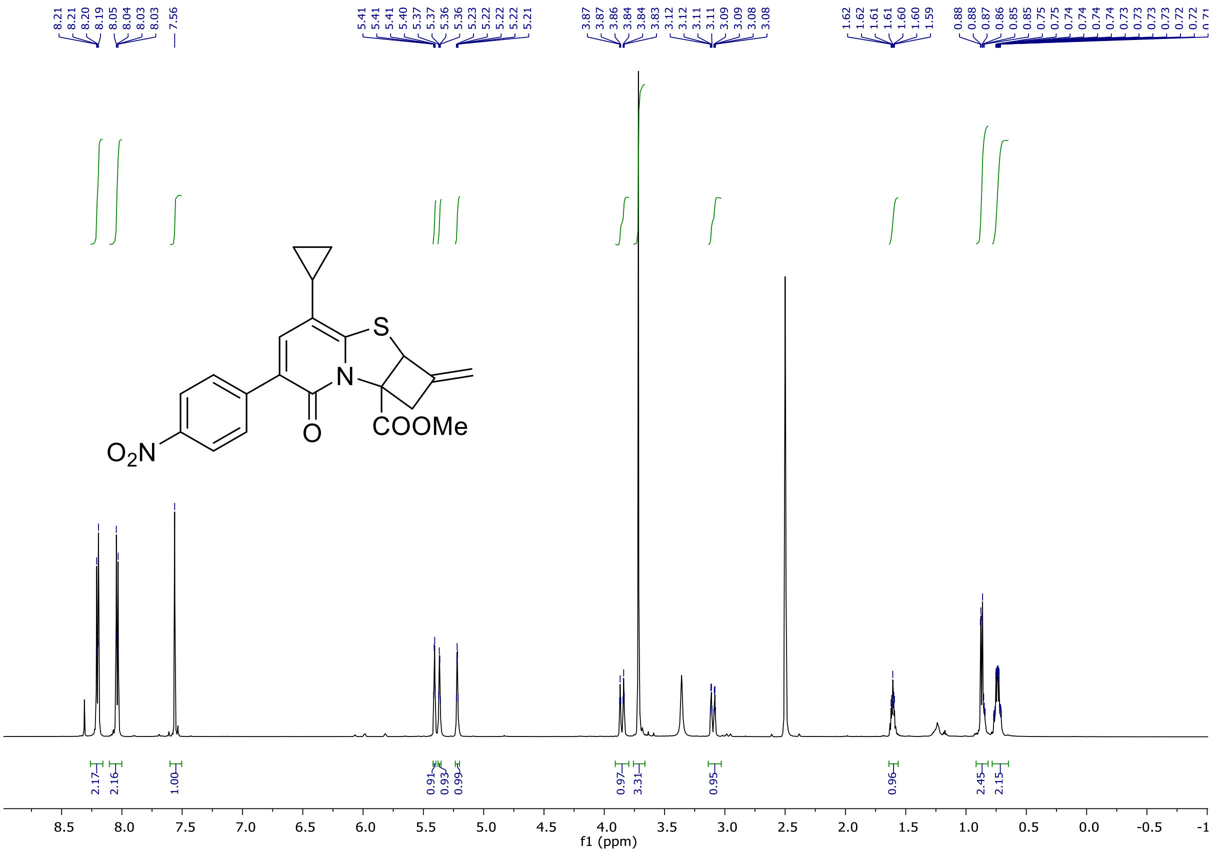

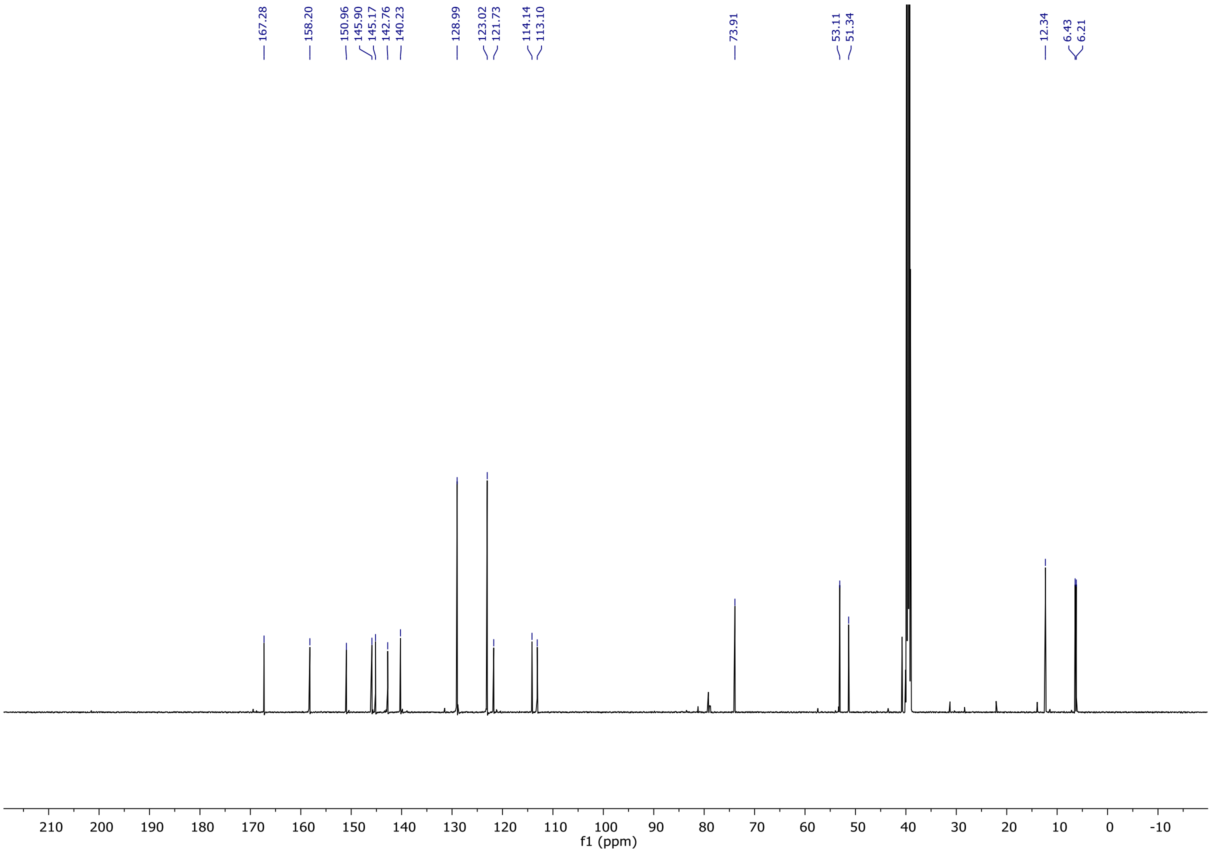

Compound **5h** ^{1}H NMR [400 MHz, $(CD_3)_2SO$] ^{13}C{^{1}H} NMR [100 MHz, $(CD_3)_2SO$]

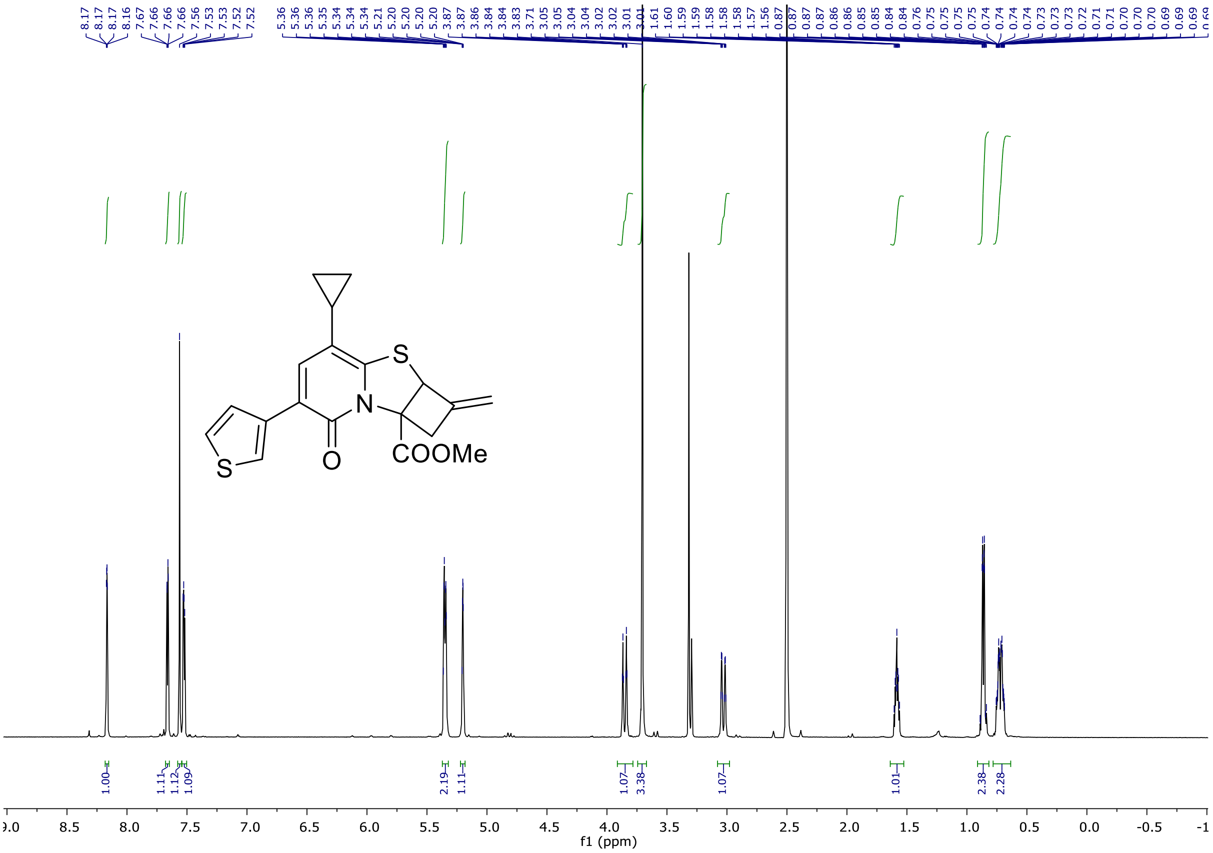

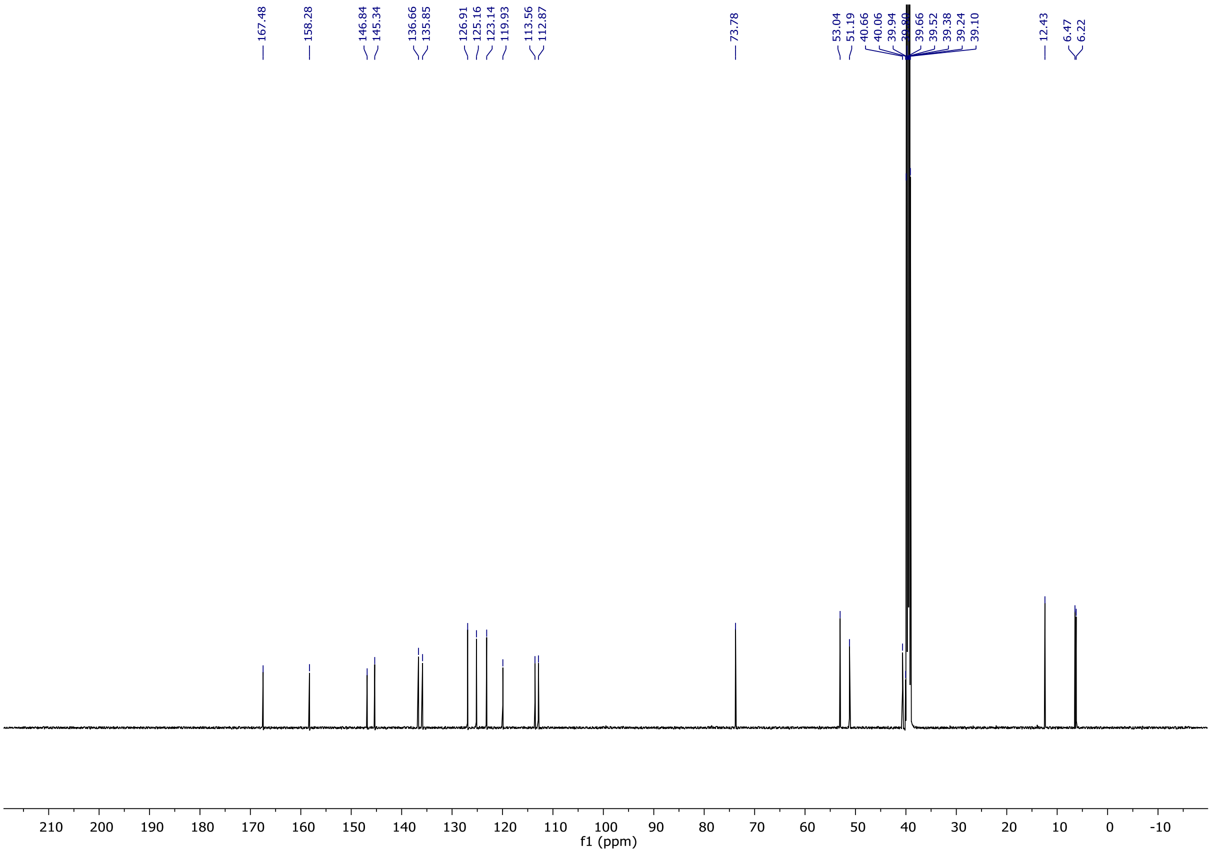

Compound **5i** ^{1}H NMR [400 MHz, $(CD_3)_2SO$] $^{13}C\{^{1}H\}$ NMR [100 MHz, $(CD_3)_2SO$]

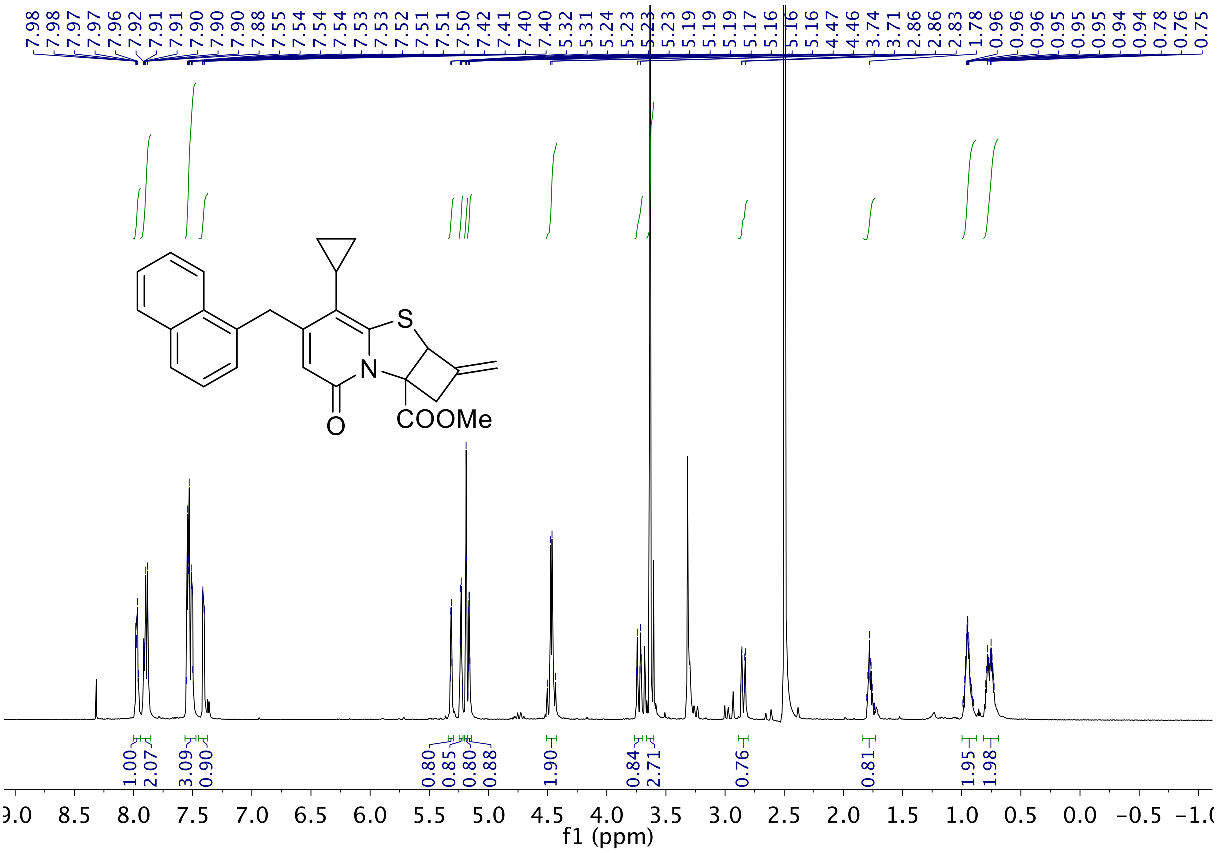

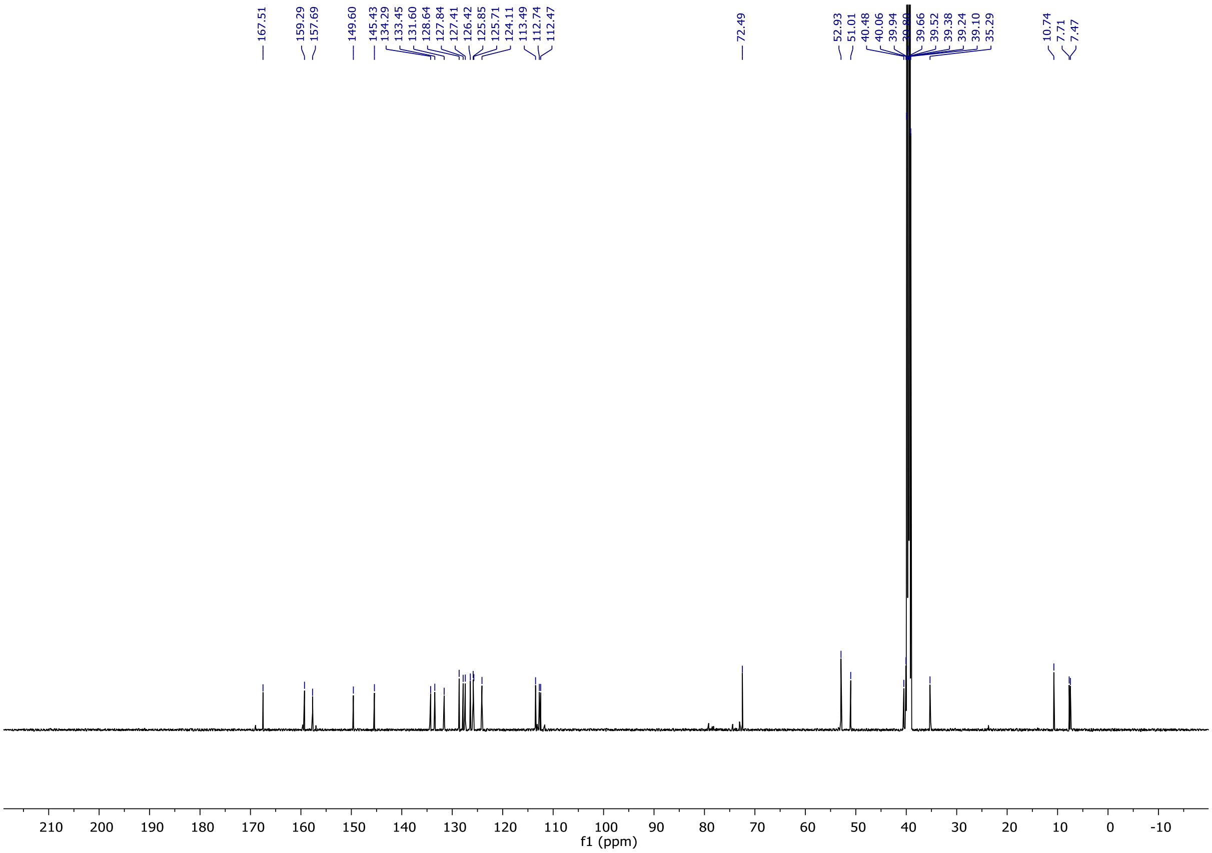

Compound **5j** ^{1}H NMR [400 MHz, $(CD_3)_2SO$] $^{13}C\{^{1}H\}$ NMR [100 MHz, $(CD_3)_2SO$] ^{19}F NMR [376 MHz, $(CD_3)_2SO$] 343 K

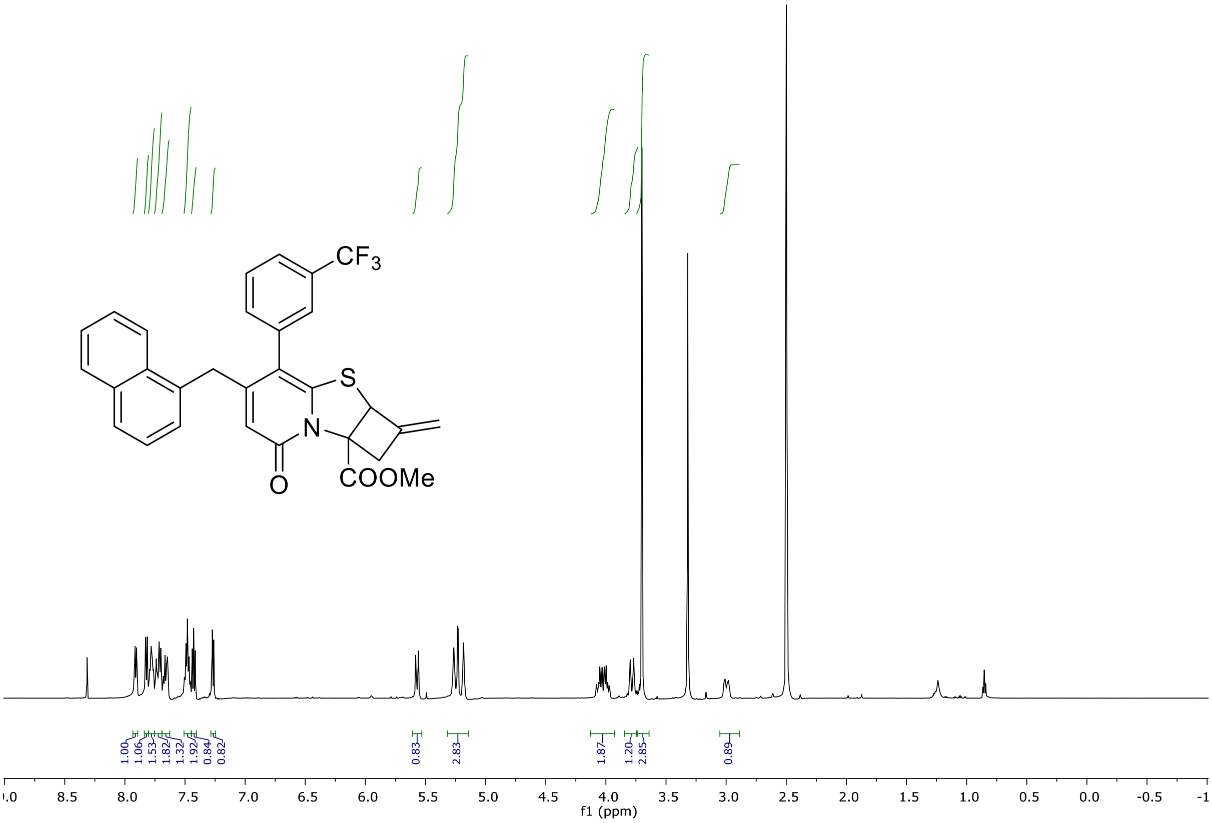

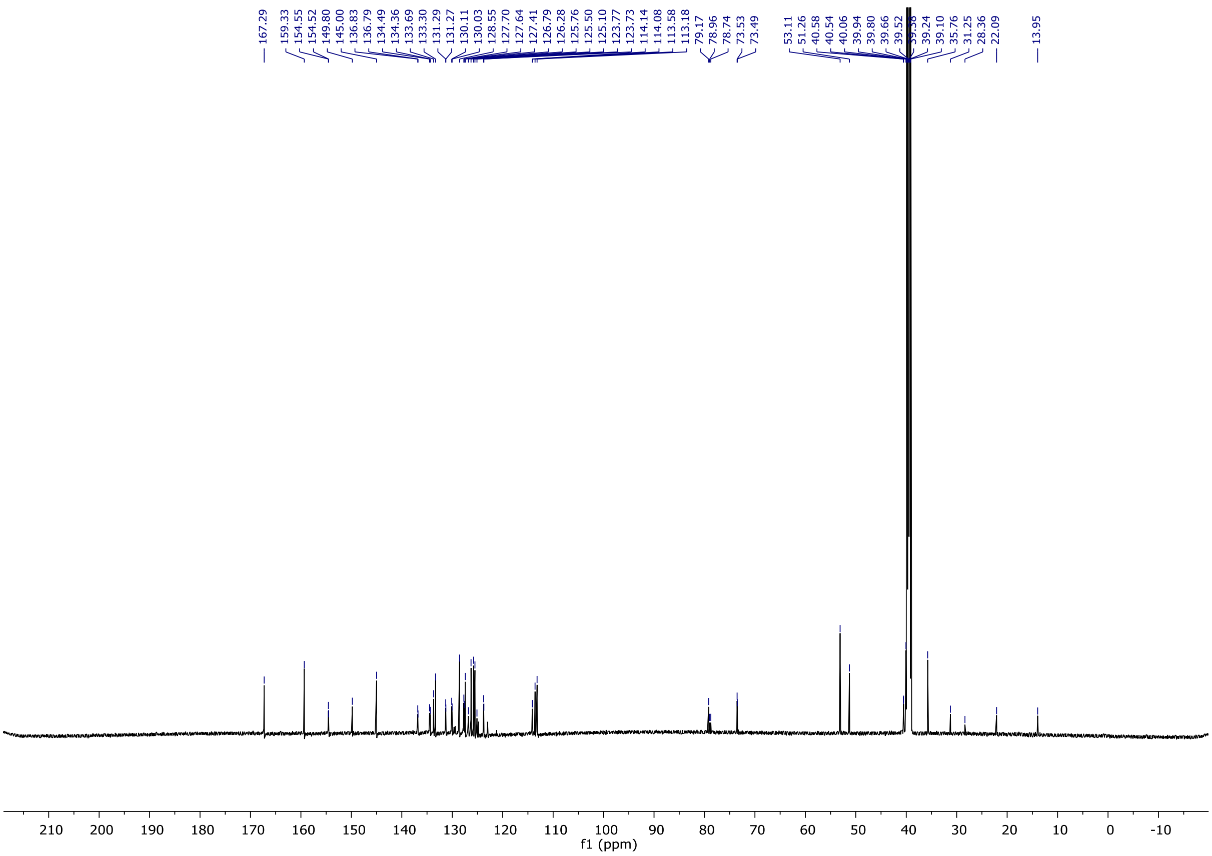

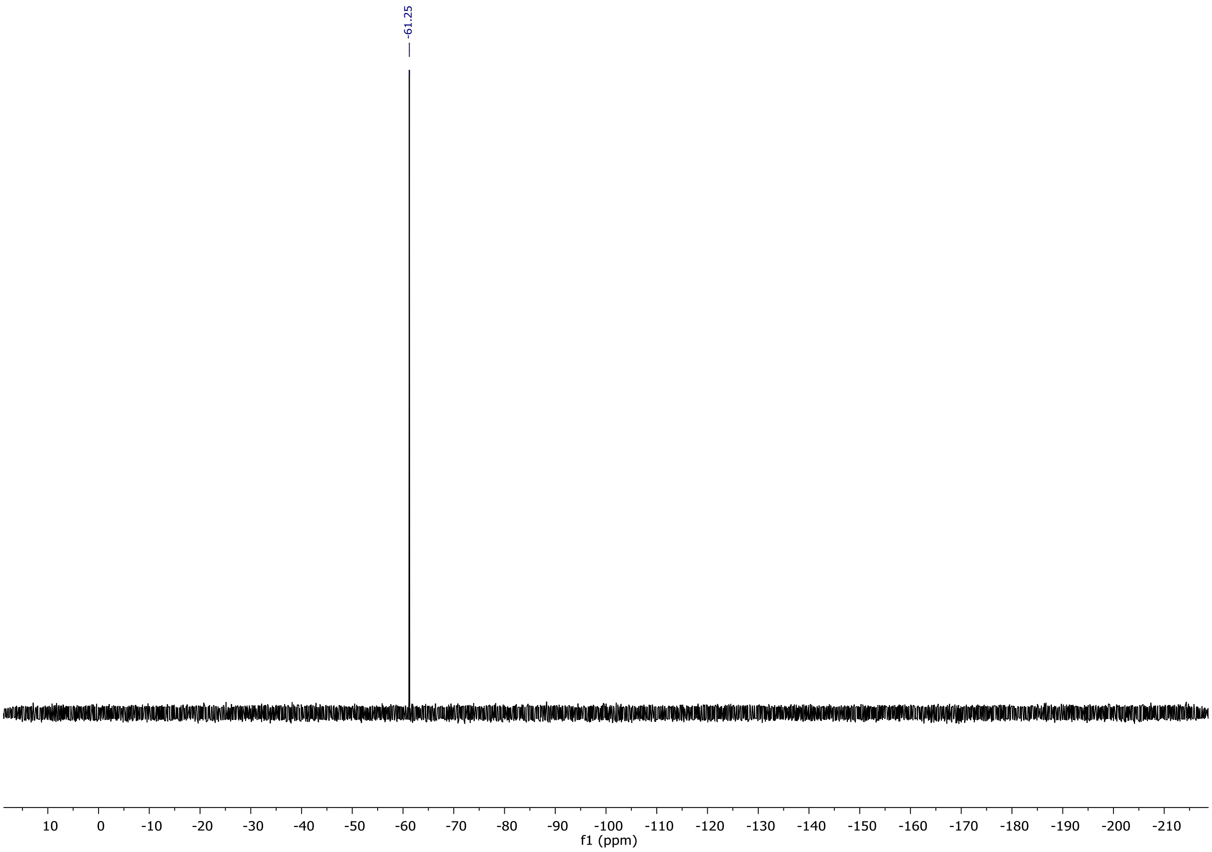
-61.25
10
0
-10
-20
-30
-40
-50
-60
-70
-80
-90
-100
-110
-120
-130
-140
-150
-160
-170
-180
-190
-200
-210
f1 (ppm)

Compound **5k** ^{1}H NMR [400 MHz, $(CD_3)_2SO$] $^{13}C\{^{1}H\}$ NMR [100 MHz, $(CD_3)_2SO$]

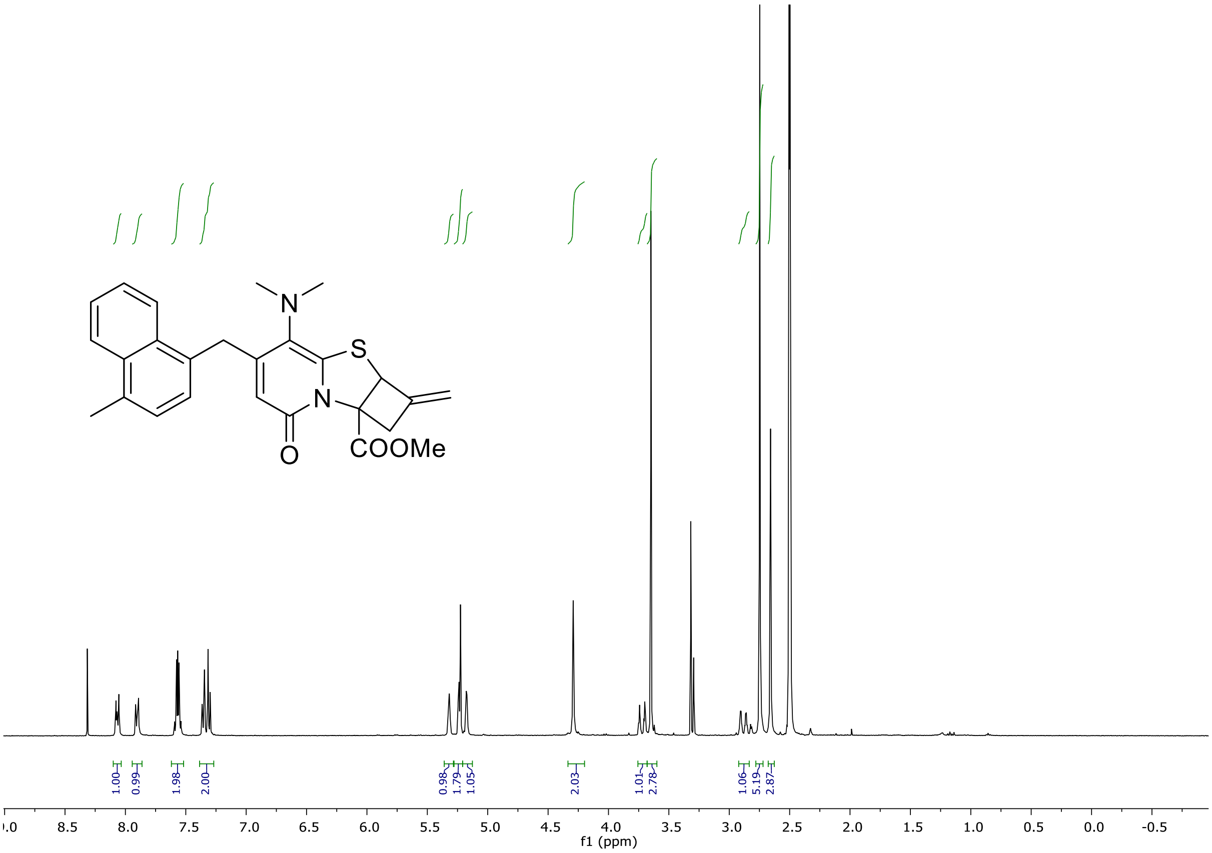

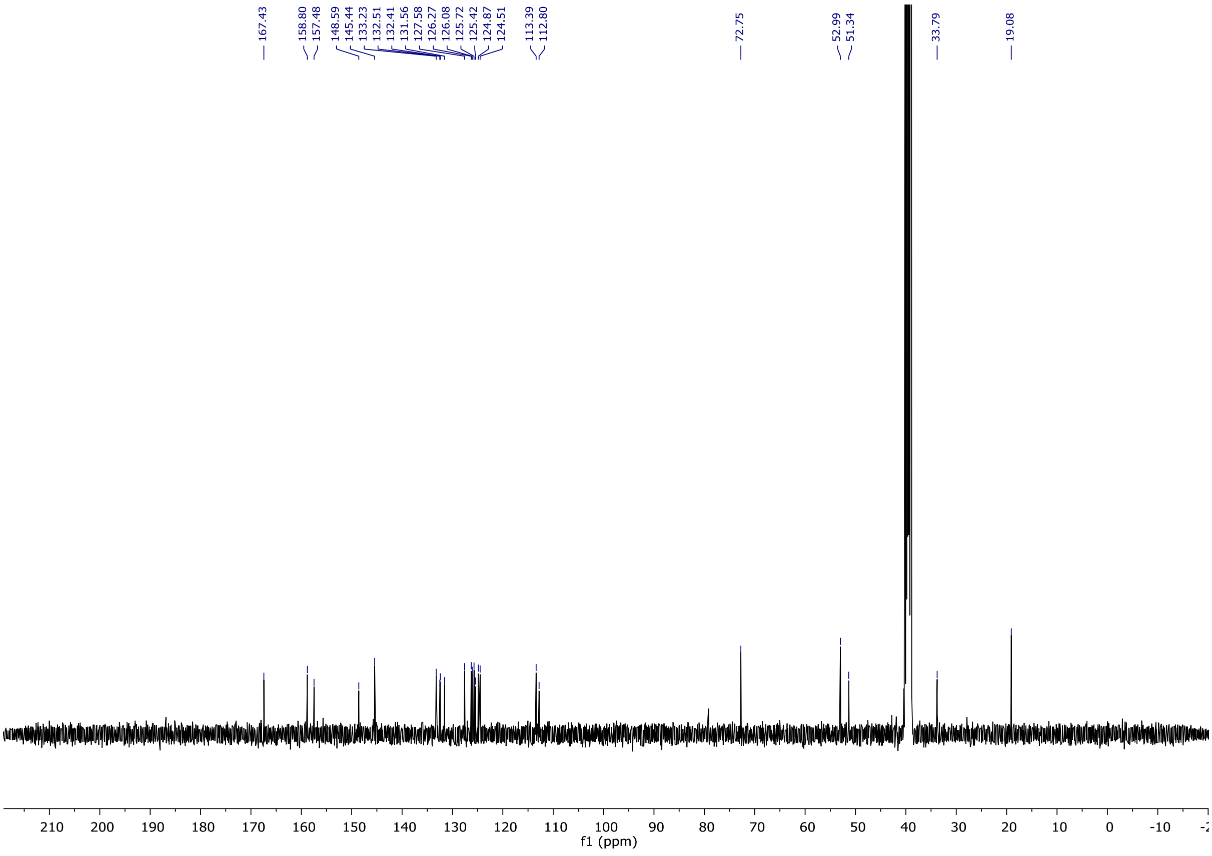

Compound **5l** ^{1}H NMR [400 MHz, $(CD_3)_2SO$] ^{13}C{^{1}H} NMR [100 MHz, $(CD_3)_2SO$]

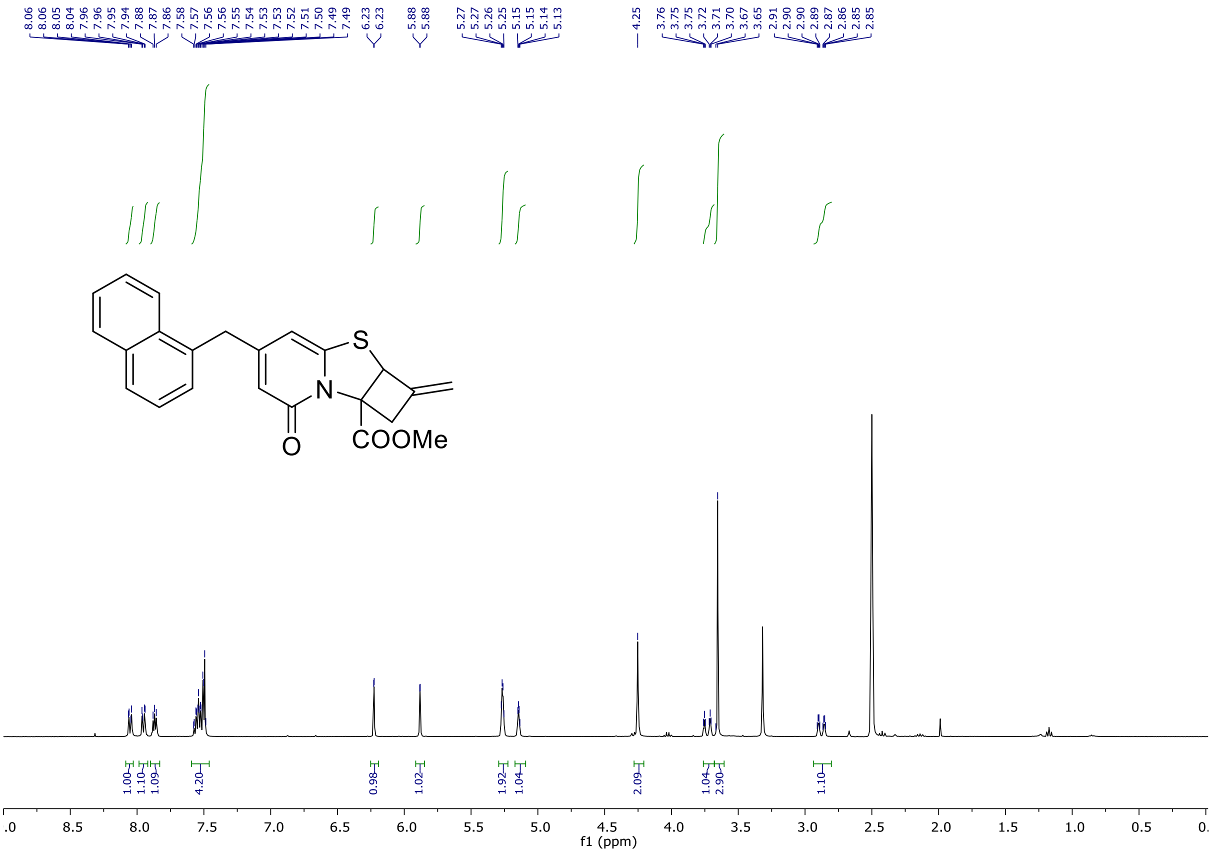

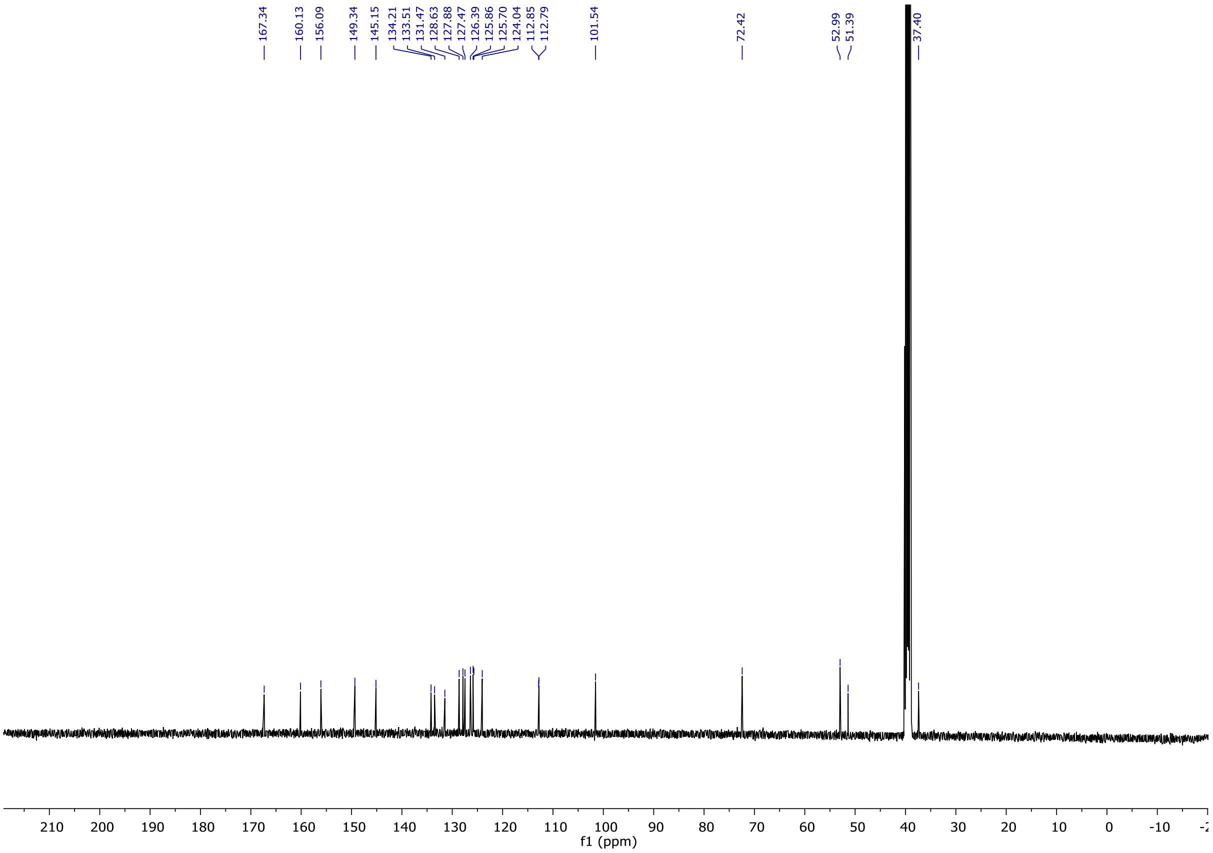

Compound **5m** (diastereomeric mixture) ^{1}H NMR [400 MHz, $CDCl_3$] $^{13}C\{^{1}H\}$ NMR [100 MHz, $CDCl_3$]

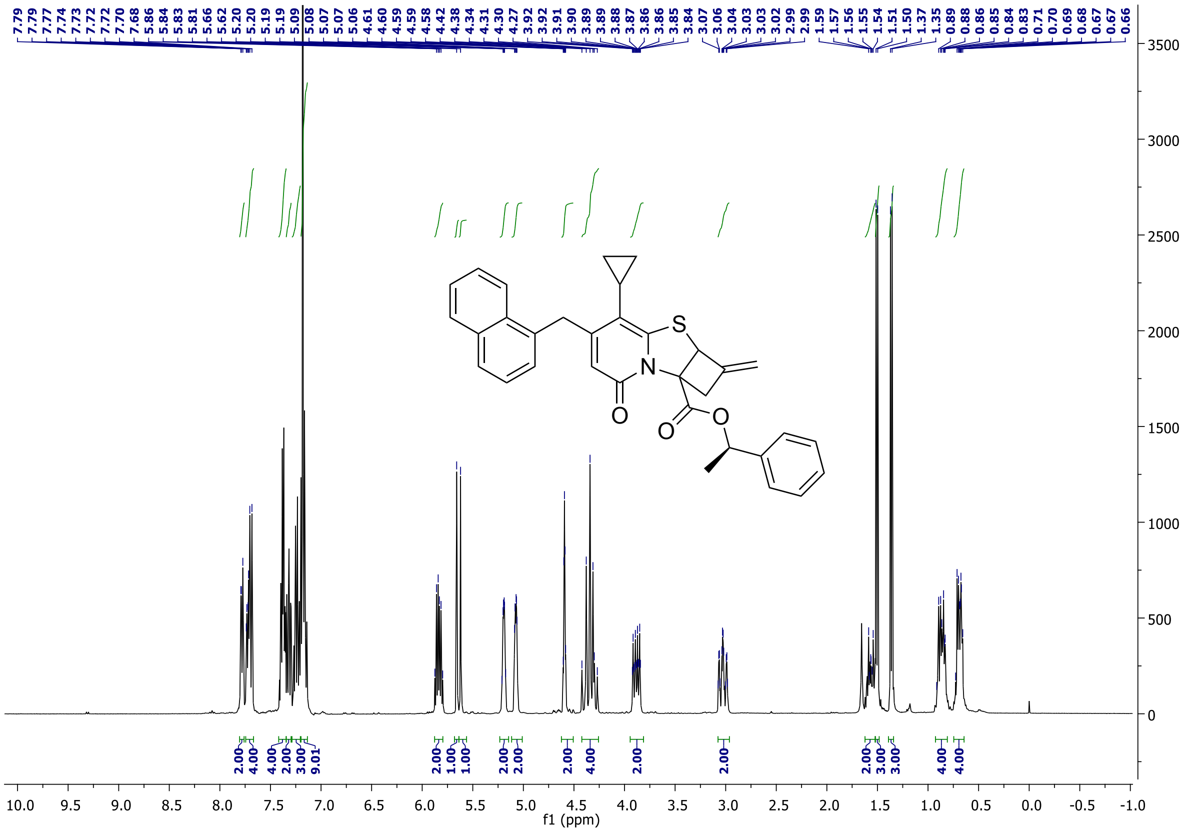

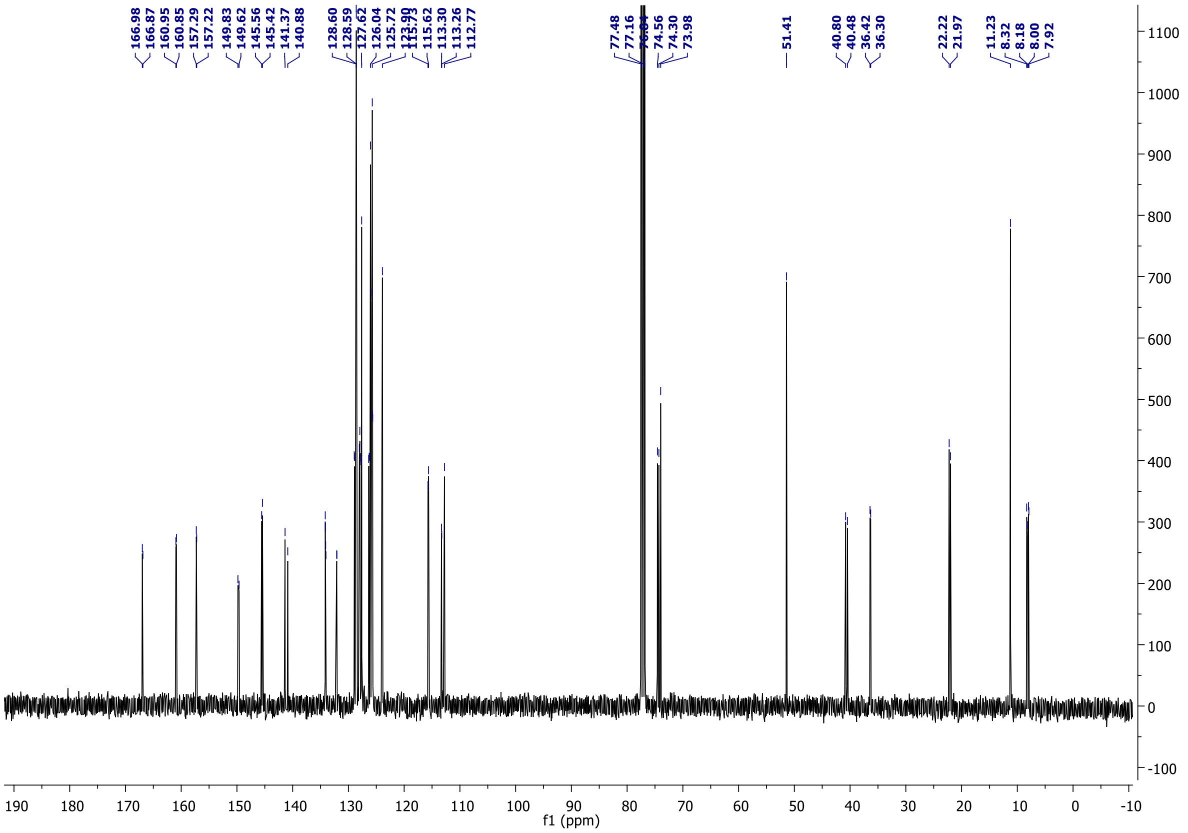

Compound **6a**. ^{1}H NMR [600 MHz, $(CD_3)_2SO$] $^{13}C\{^{1}H\}$ NMR [151 MHz, $(CD_3)_2SO$]

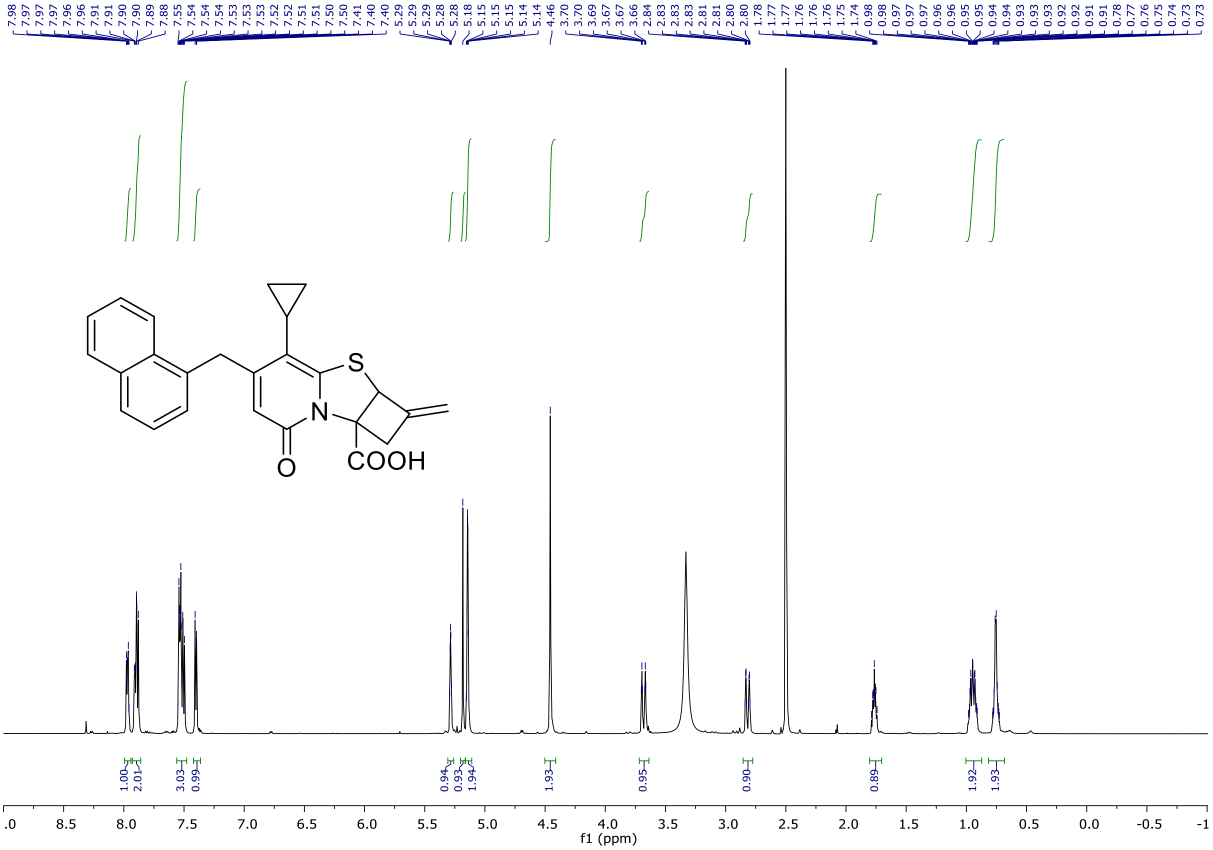

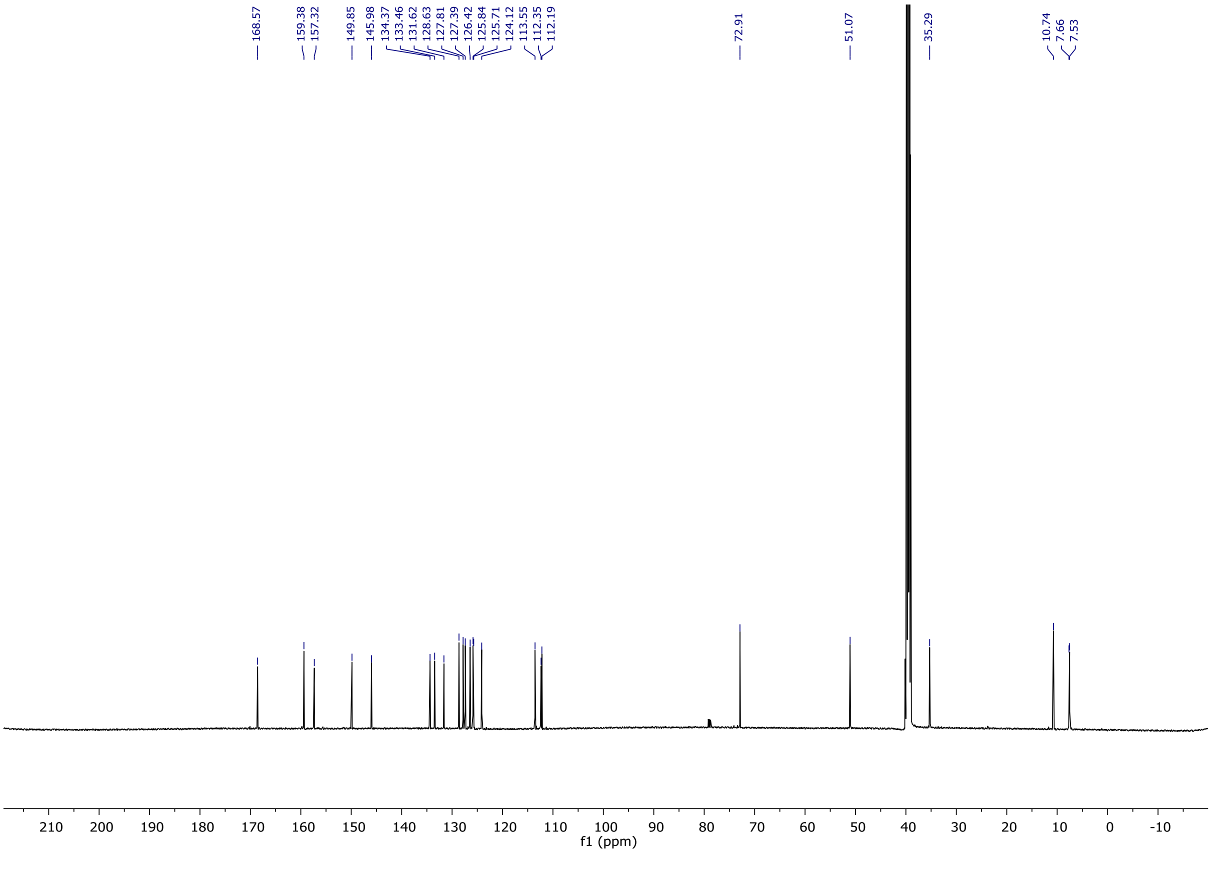

Compound **6b** ^{1}H NMR [600 MHz, $(CD_3)_2SO$] ^{13}C{^{1}H} NMR [151 MHz, $(CD_3)_2SO$] ^{19}F NMR [376 MHz, $(CD_3)_2SO$] 343 K

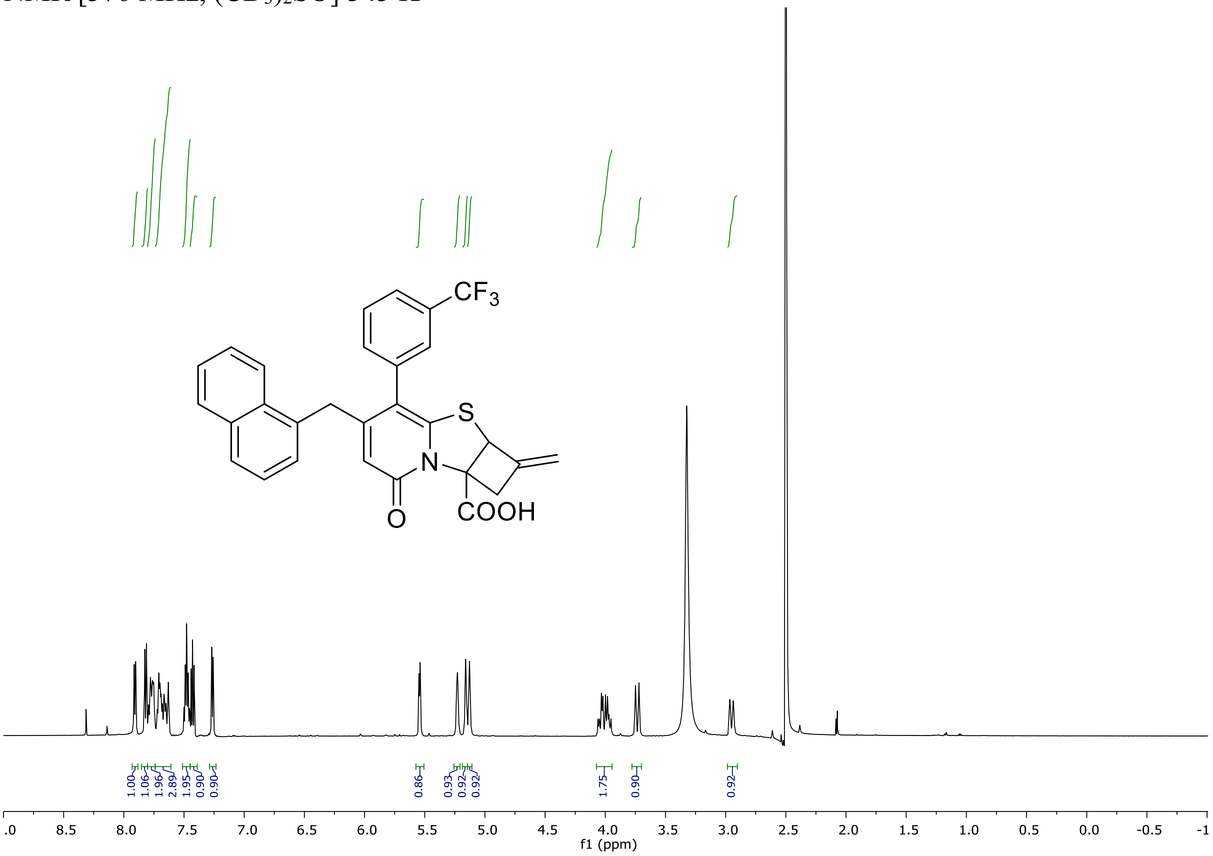

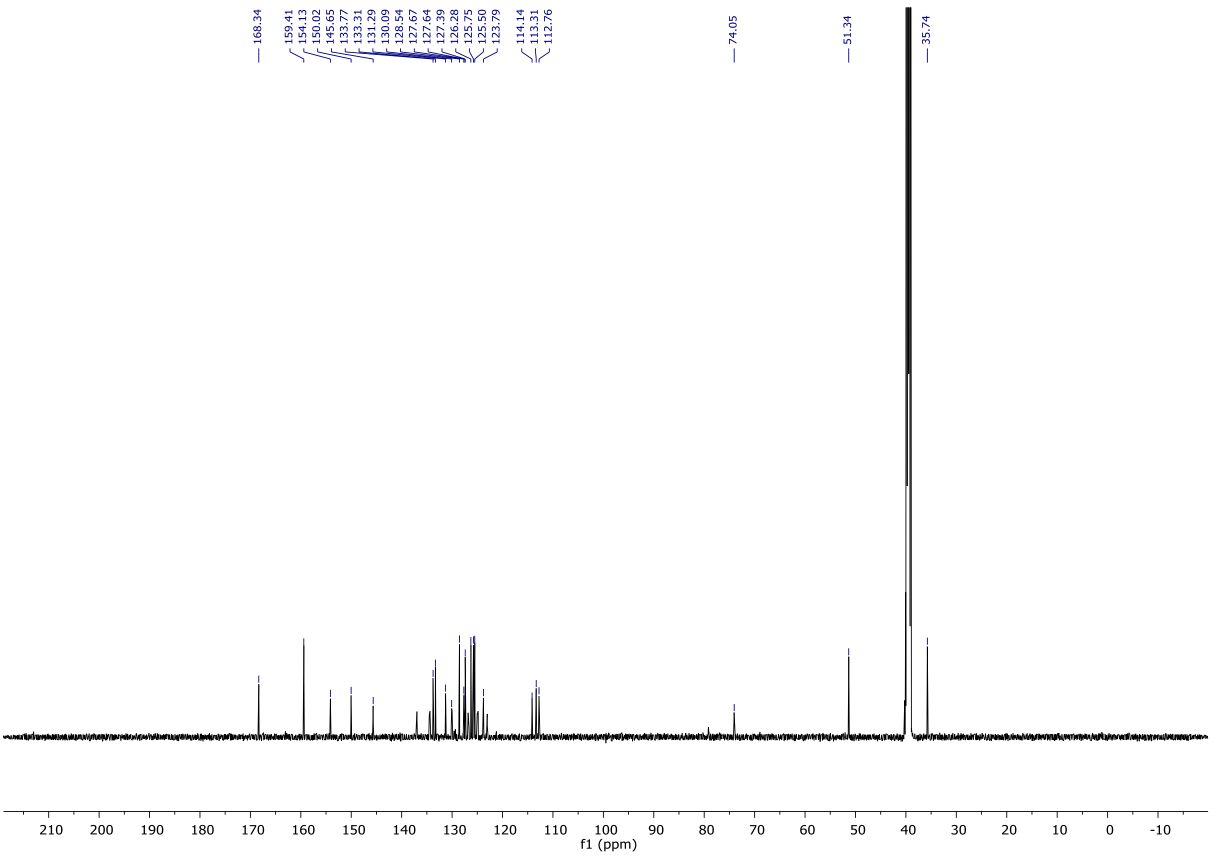

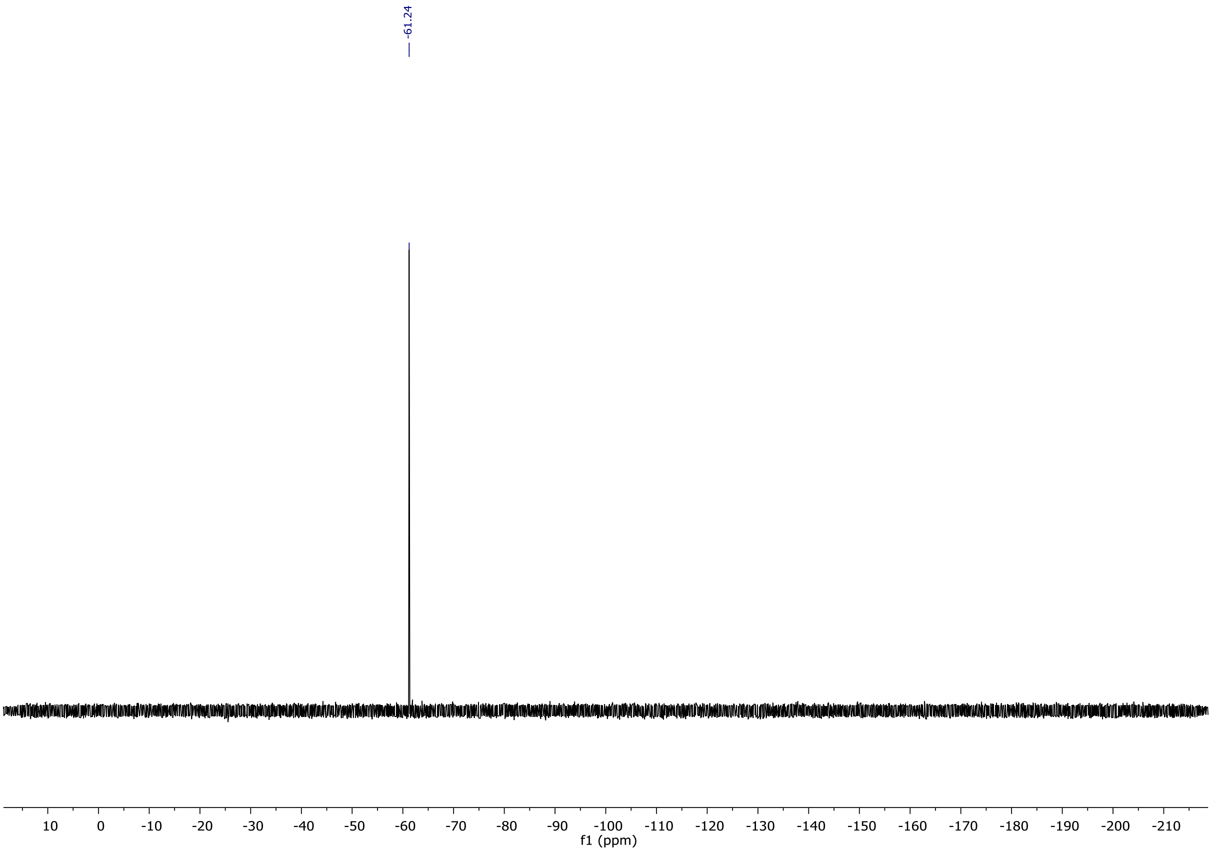
-61.24
10 0 -10 -20 -30 -40 -50 -60 -70 -80 -90 -100 -110 -120 -130 -140 -150 -160 -170 -180 -190 -200 -210
f1 (ppm)

Compound **6b (-)** ^{1}H NMR [600 MHz, $(CD_3)_2SO$] $^{13}C\{^{1}H\}$ NMR [151 MHz, $(CD_3)_2SO$]

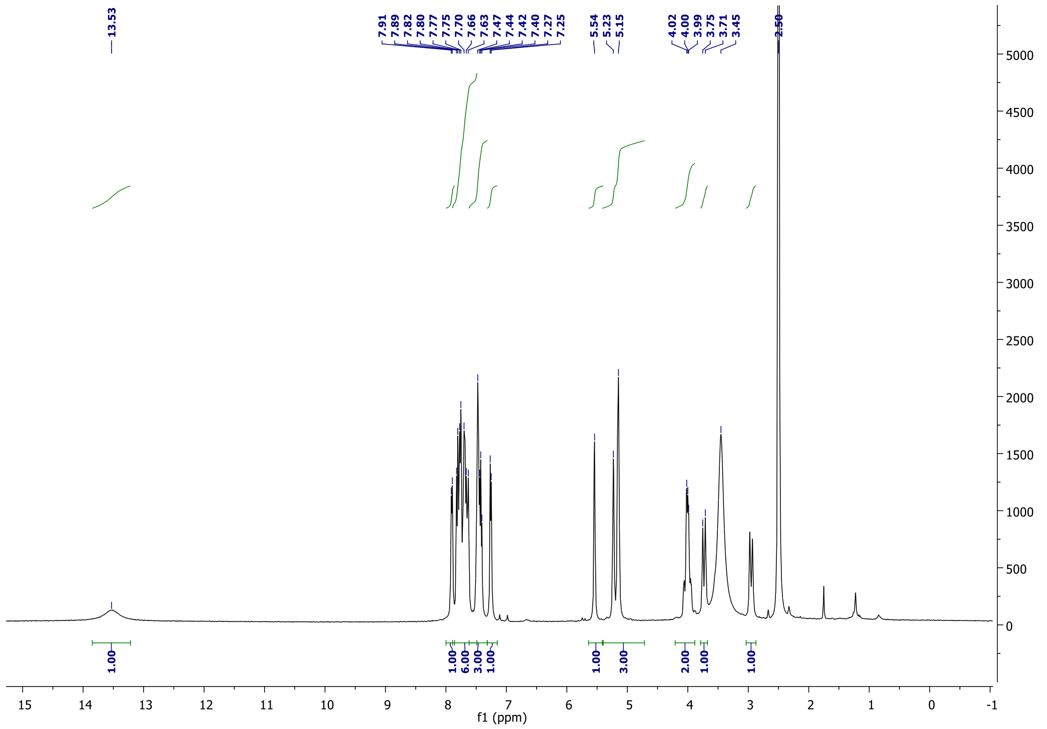

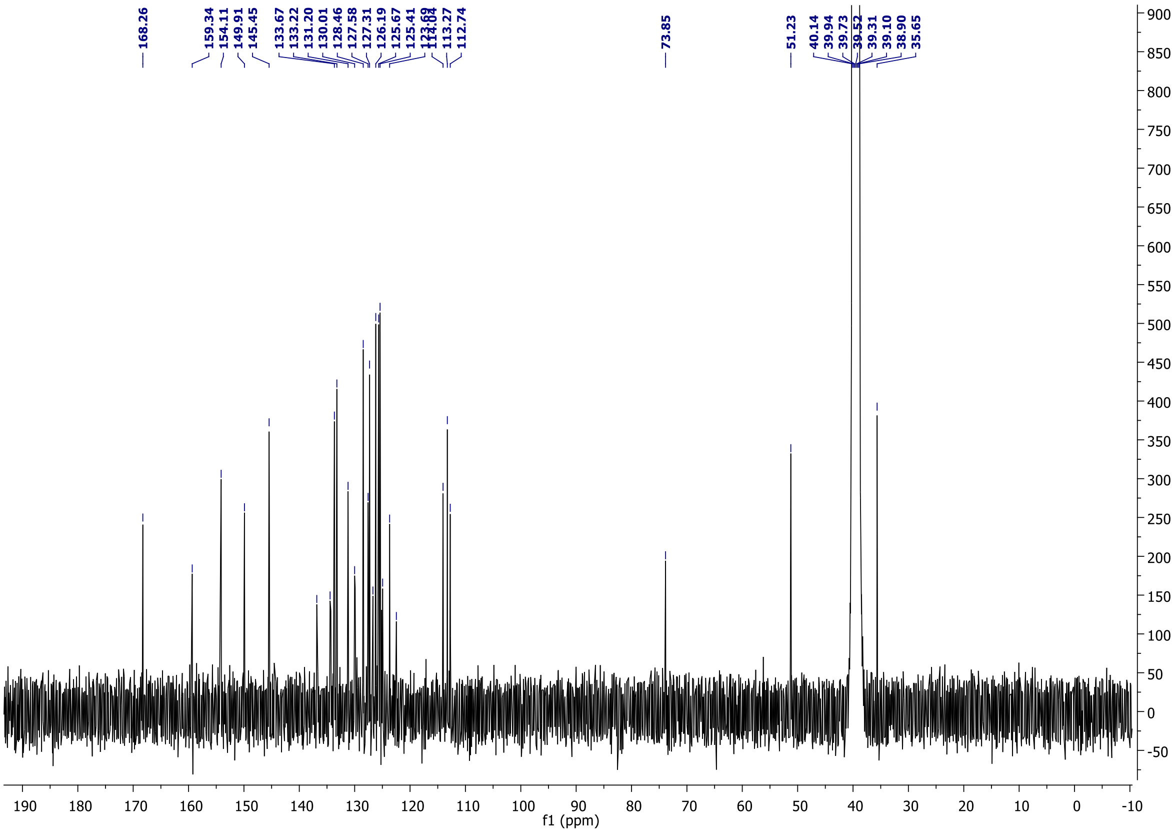

Compound **6b (+)** ^{1}H NMR [600 MHz, $(CD_3)_2SO$] $^{13}C\{^{1}H\}$ NMR [151 MHz, $(CD_3)_2SO$]

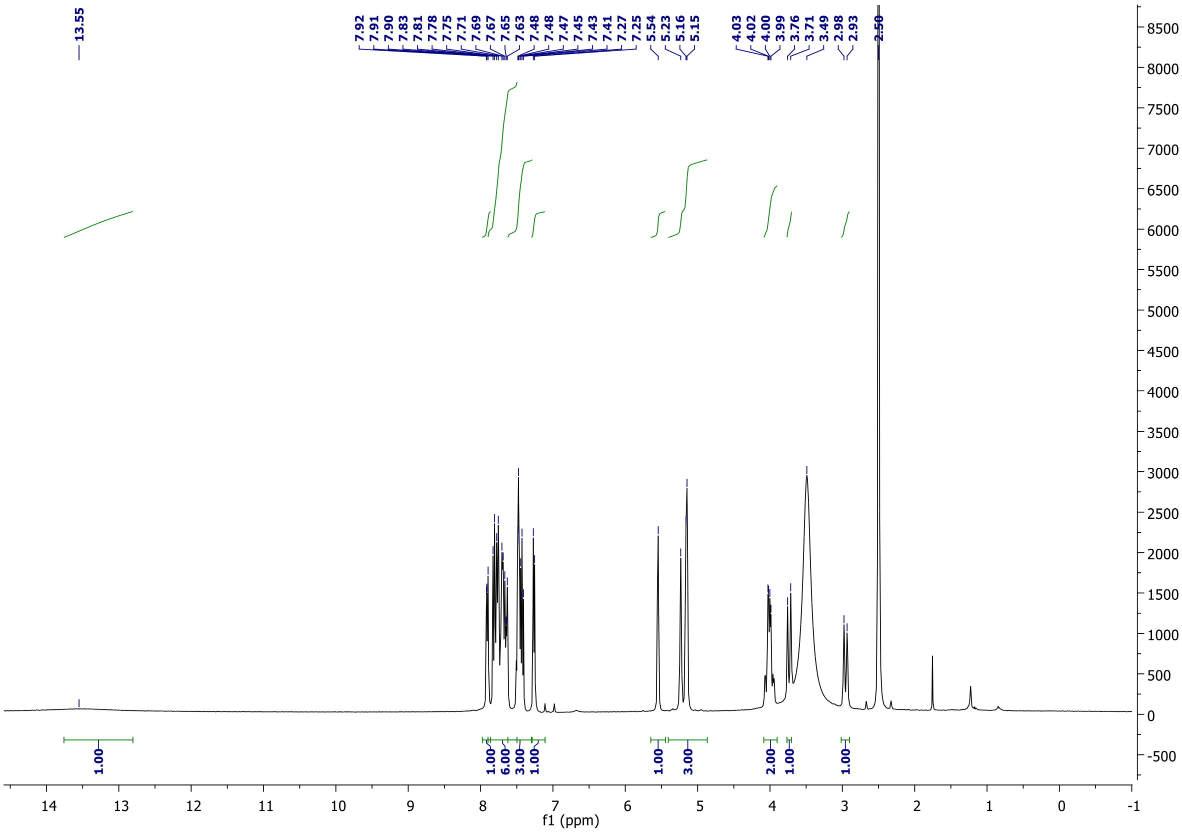

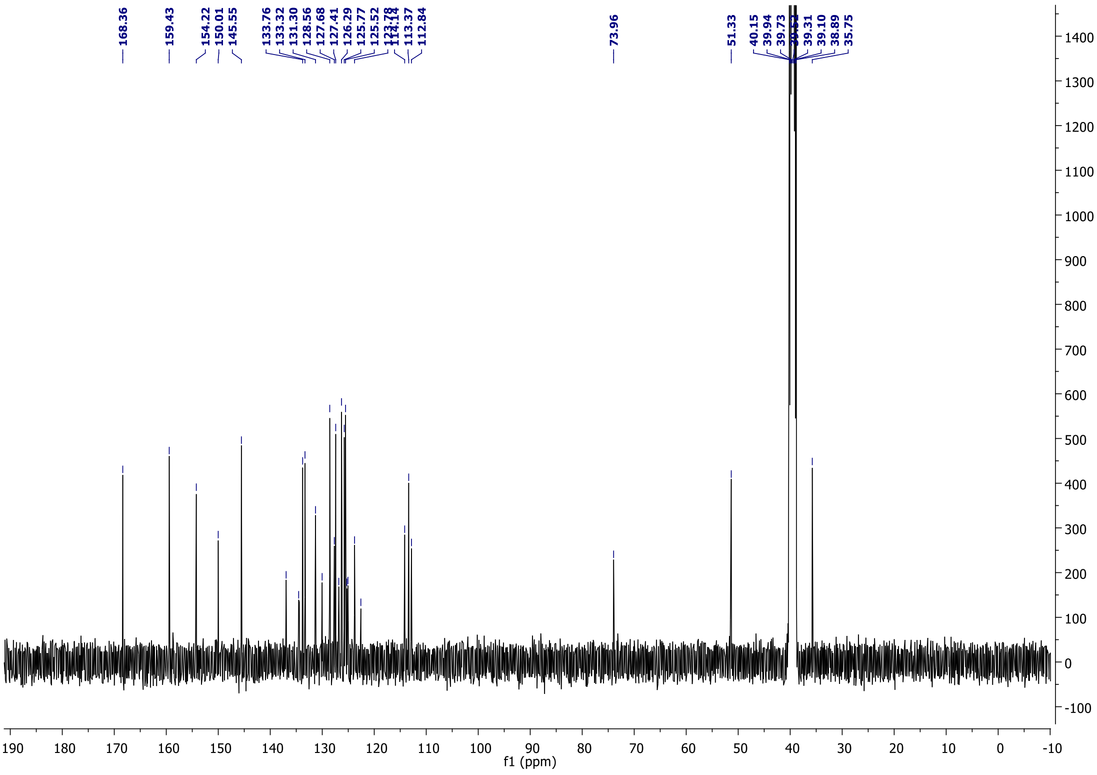

Compound **6c** ^{1}H NMR [600 MHz, $(CD_3)_2SO$] $^{13}C\{^{1}H\}$ NMR [151 MHz, $(CD_3)_2SO$]

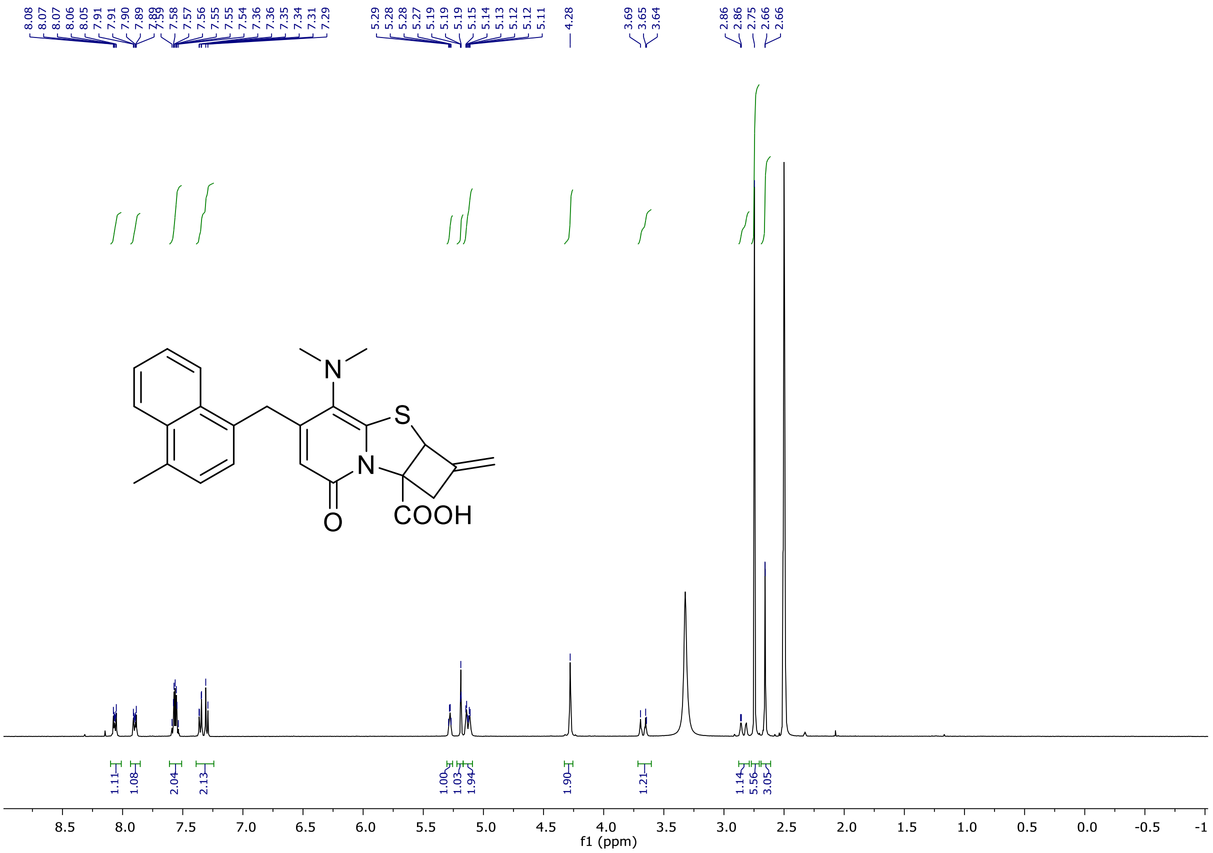

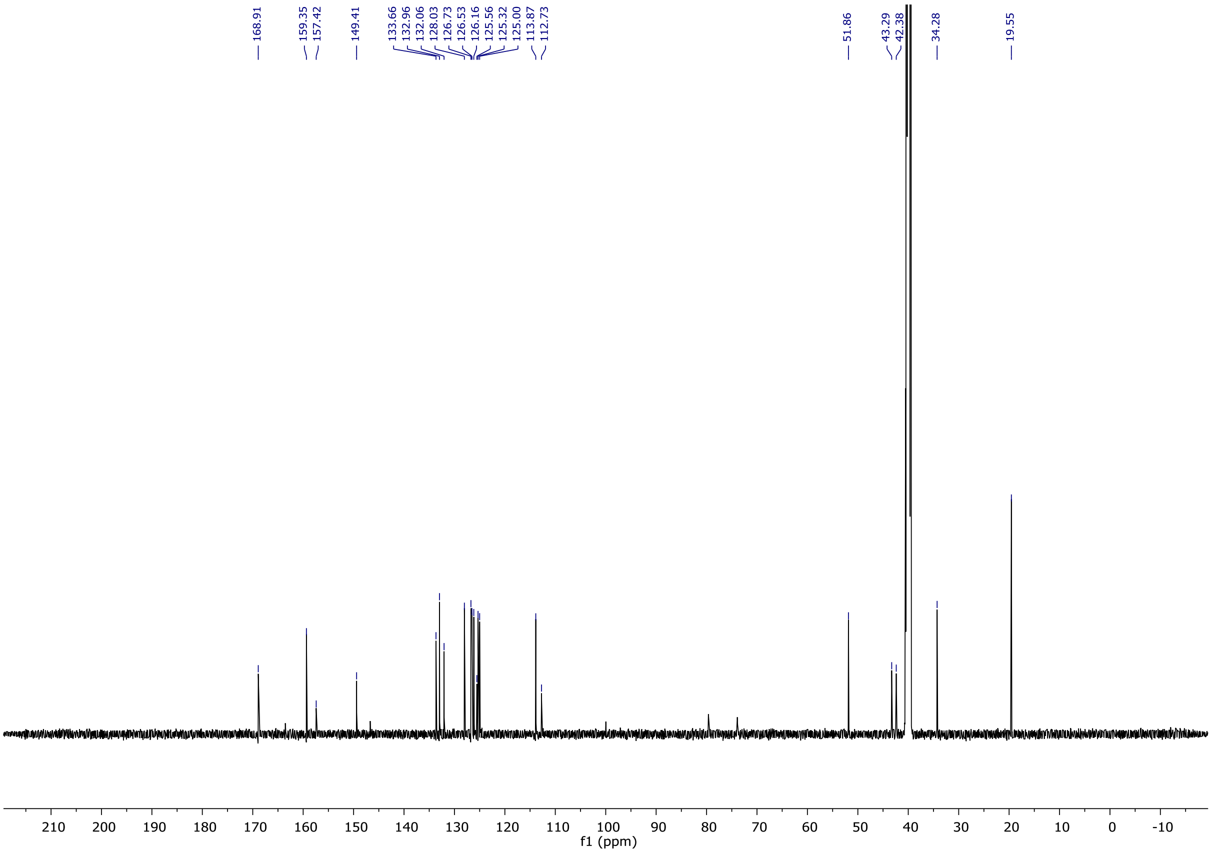

Compound **9a**. ^{1}H NMR [600 MHz, $(CD_3)_2SO$] ^{13}C{^{1}H} NMR [151 MHz, $(CD_3)_2SO$]

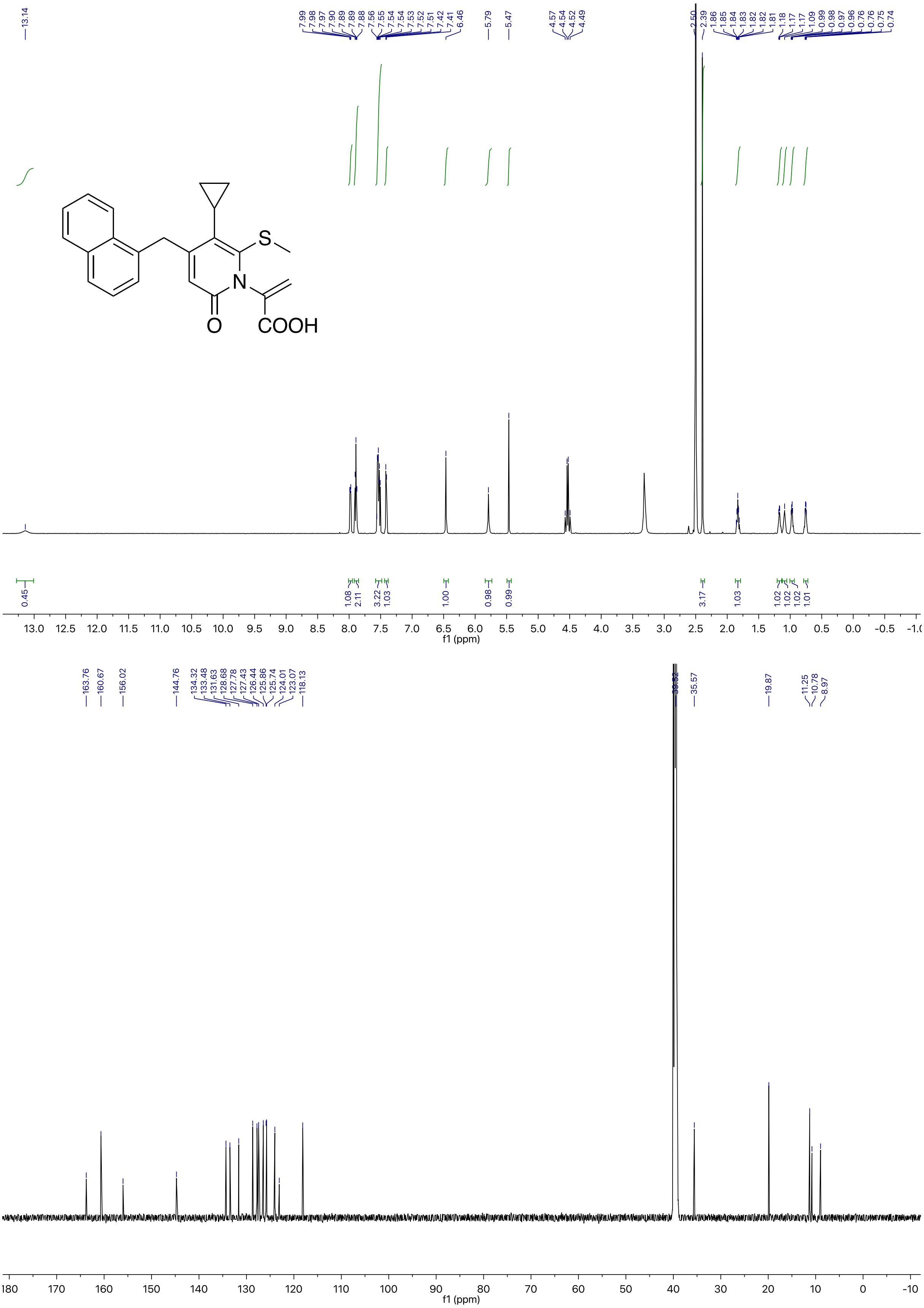

Compound **7**. ^{1}H NMR [600 MHz, $(CD_3)_2SO$] ^{13}C{^{1}H} NMR [151 MHz, $(CD_3)_2SO$]

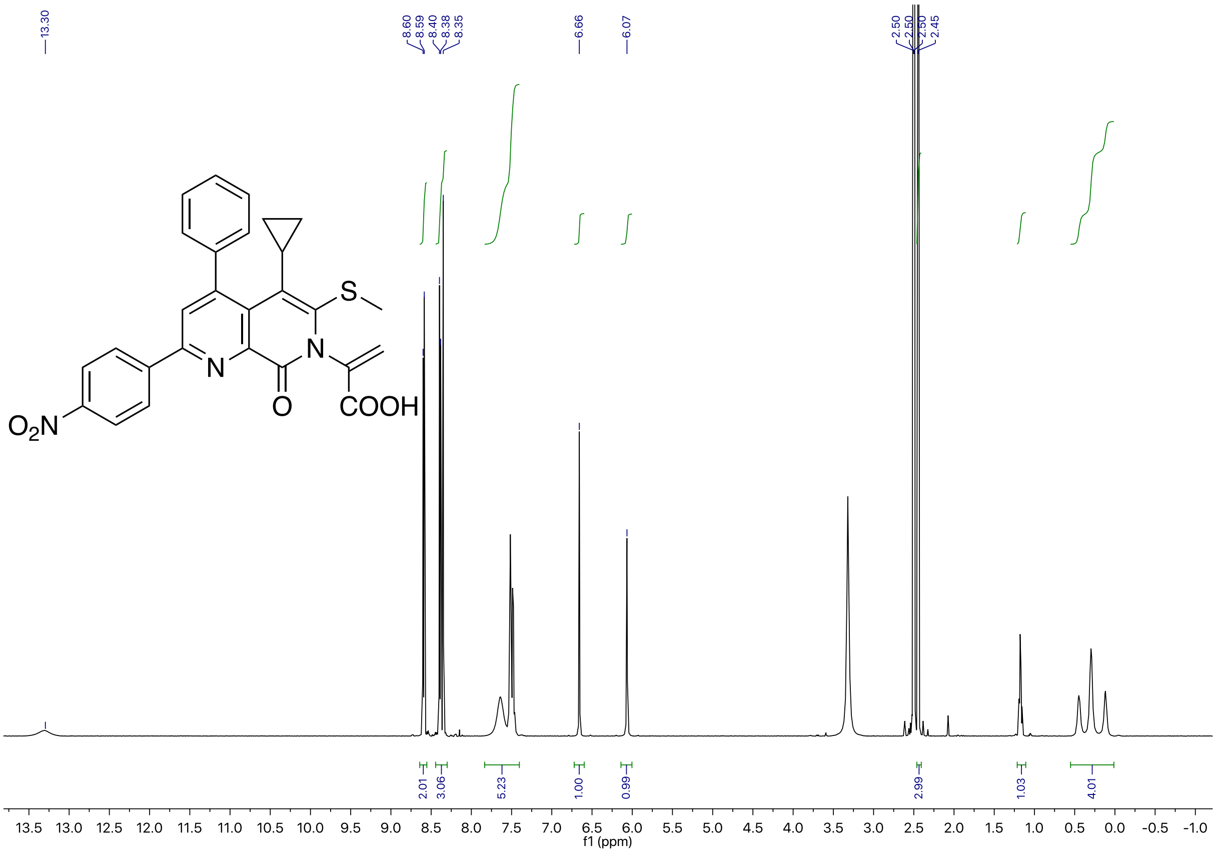

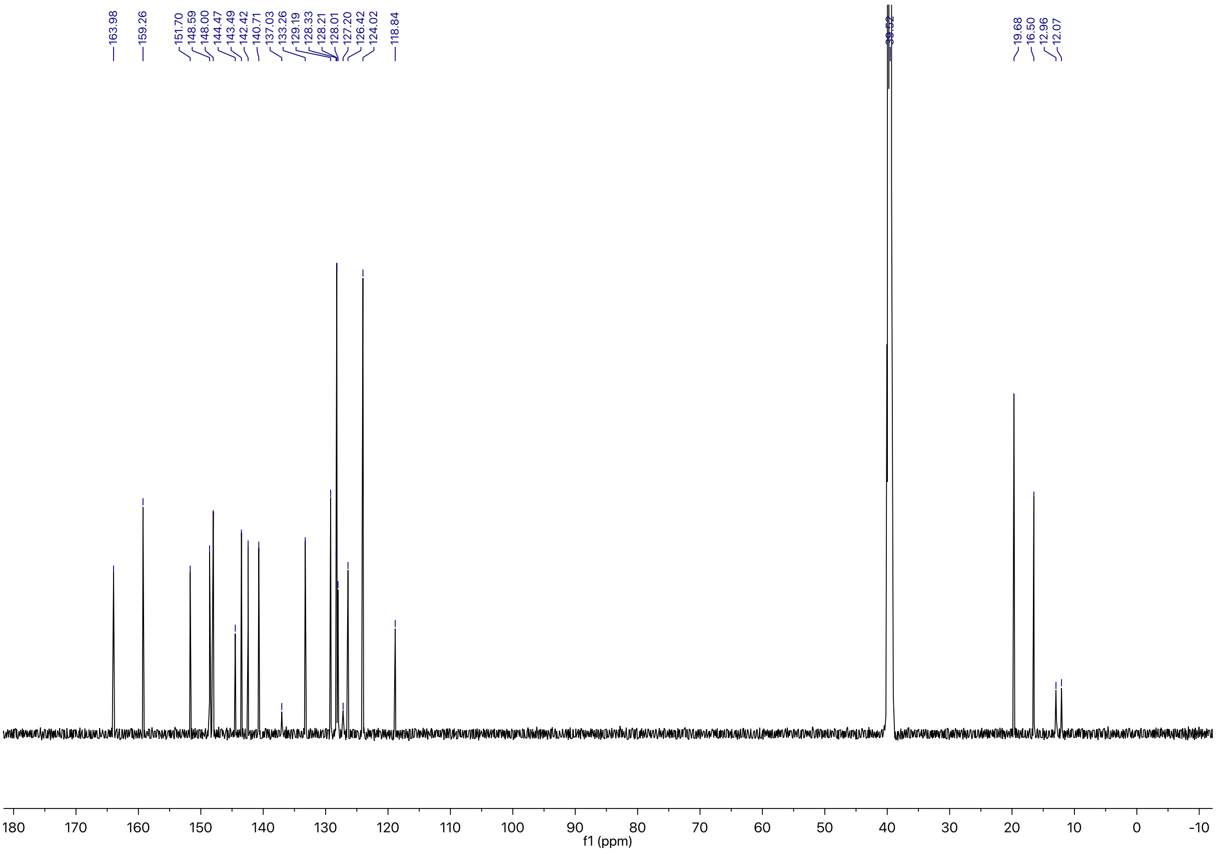

Compound **9b**. ^{1}H NMR [600 MHz, $(CD_3)_2SO$] $^{13}C\{^{1}H\}$ NMR [151 MHz, $(CD_3)_2SO$]

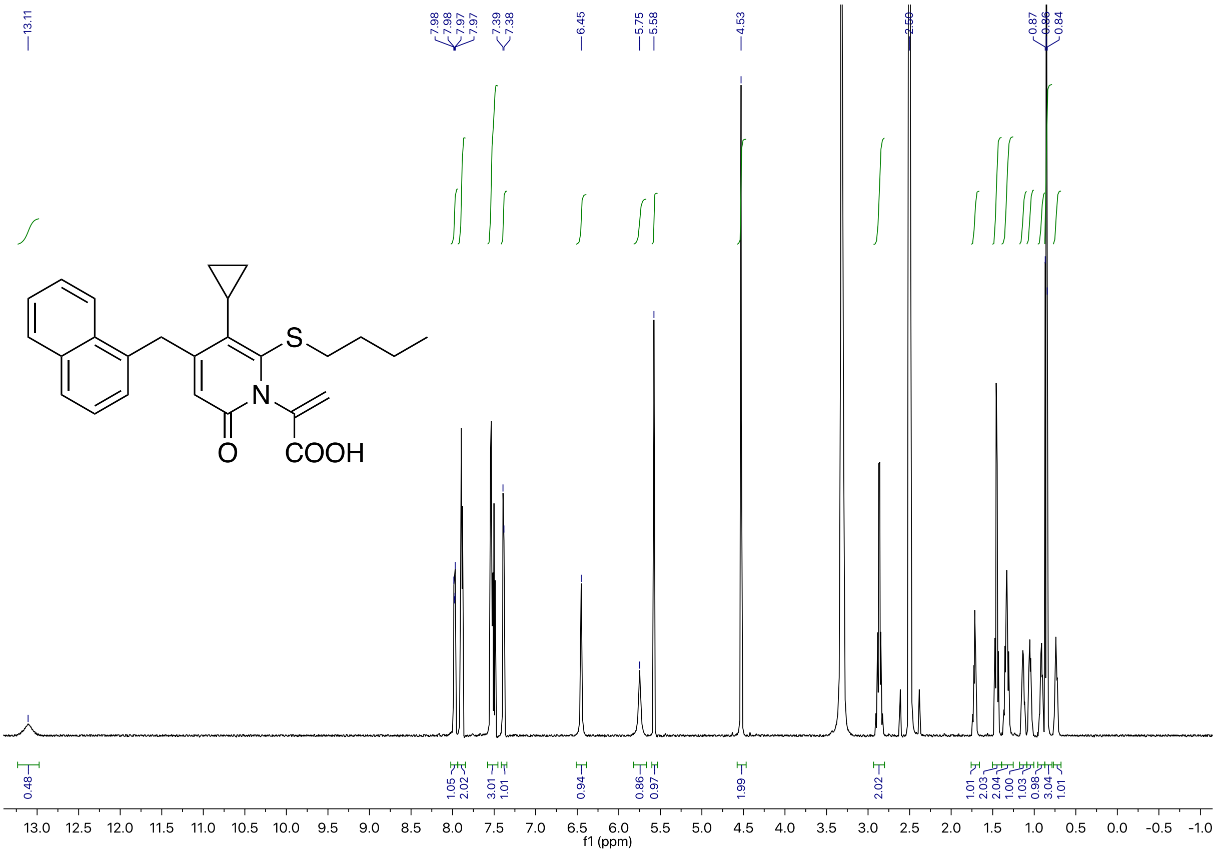

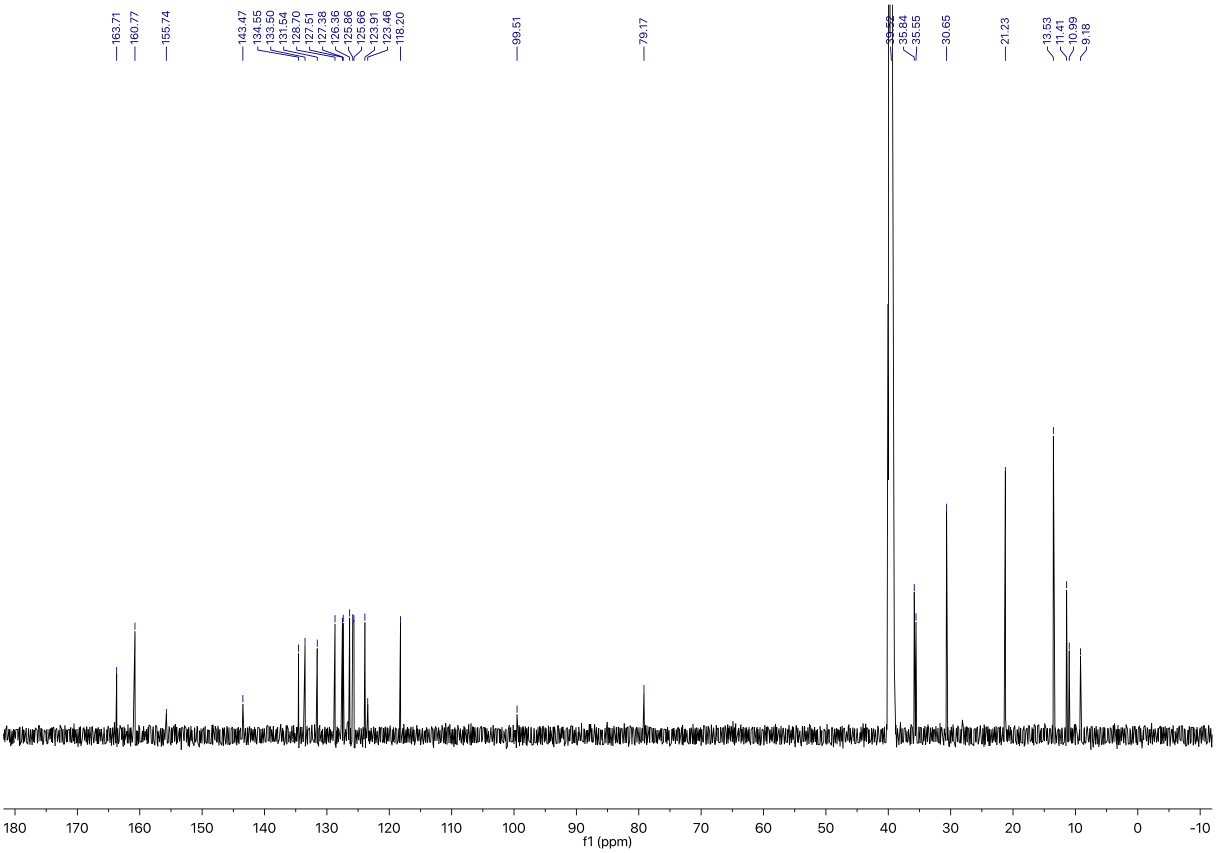

Compound **10a** ^{1}H NMR [400 MHz, $(CD_3)_2SO$] $^{13}C\{^1H\}$ NMR [100 MHz, $(CD_3)_2SO$]

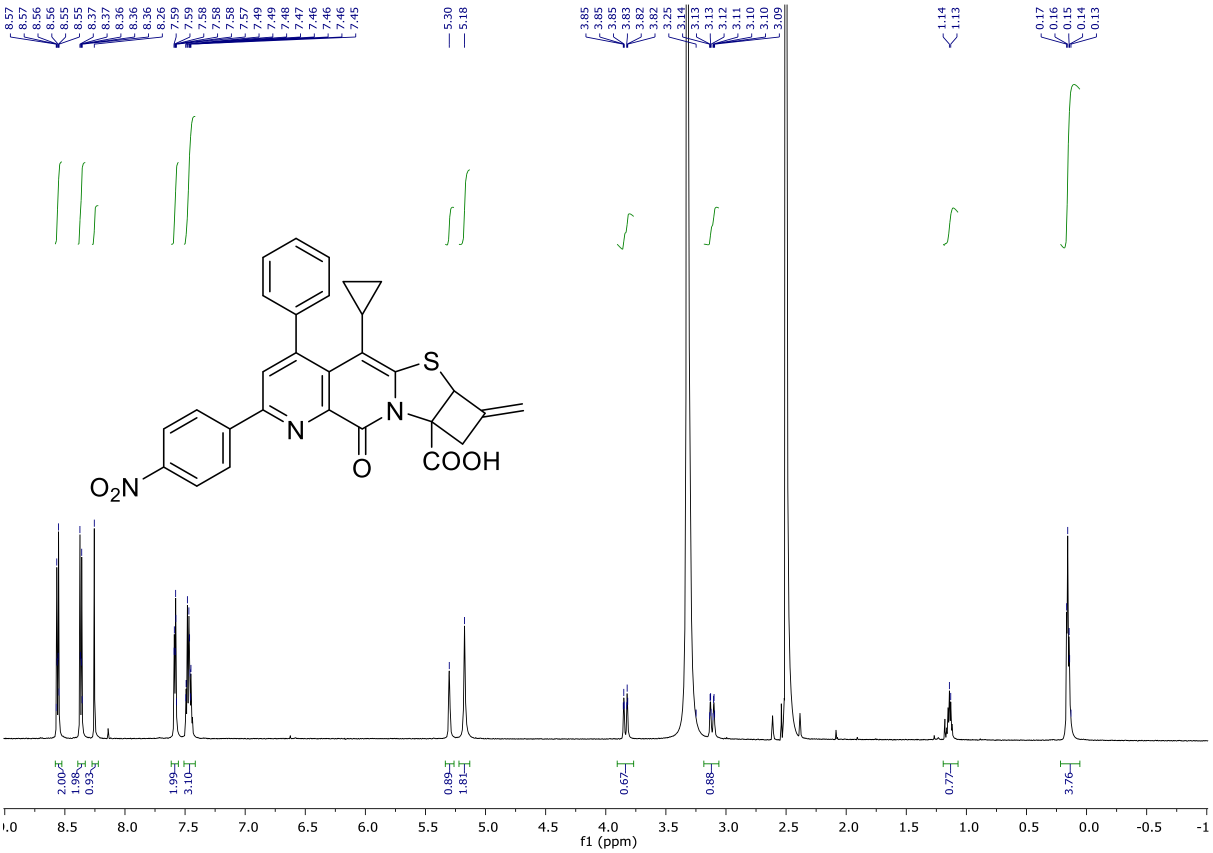

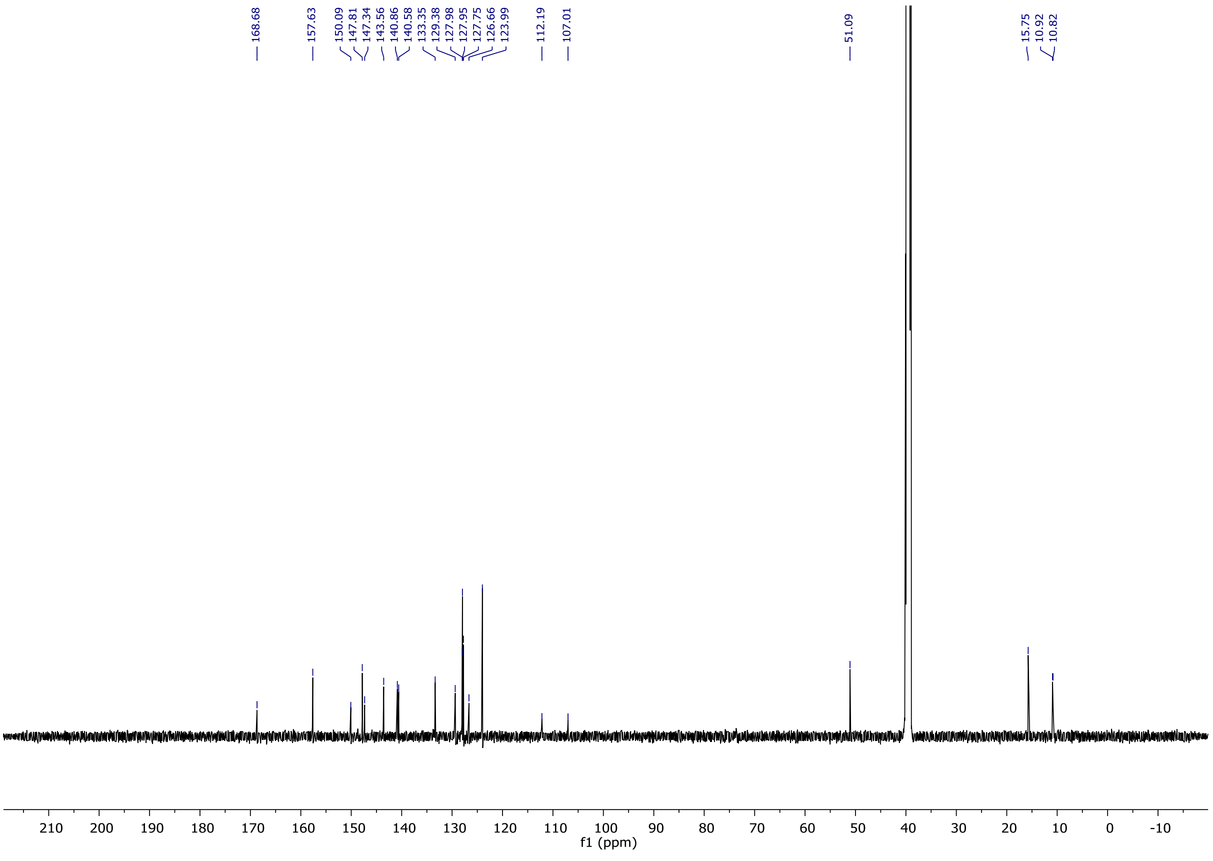

Compound **10b** ^{1}H NMR [400 MHz, $(CD_3)_2SO$] $^{13}C\{^{1}H\}$ NMR [100 MHz, $(CD_3)_2SO$]

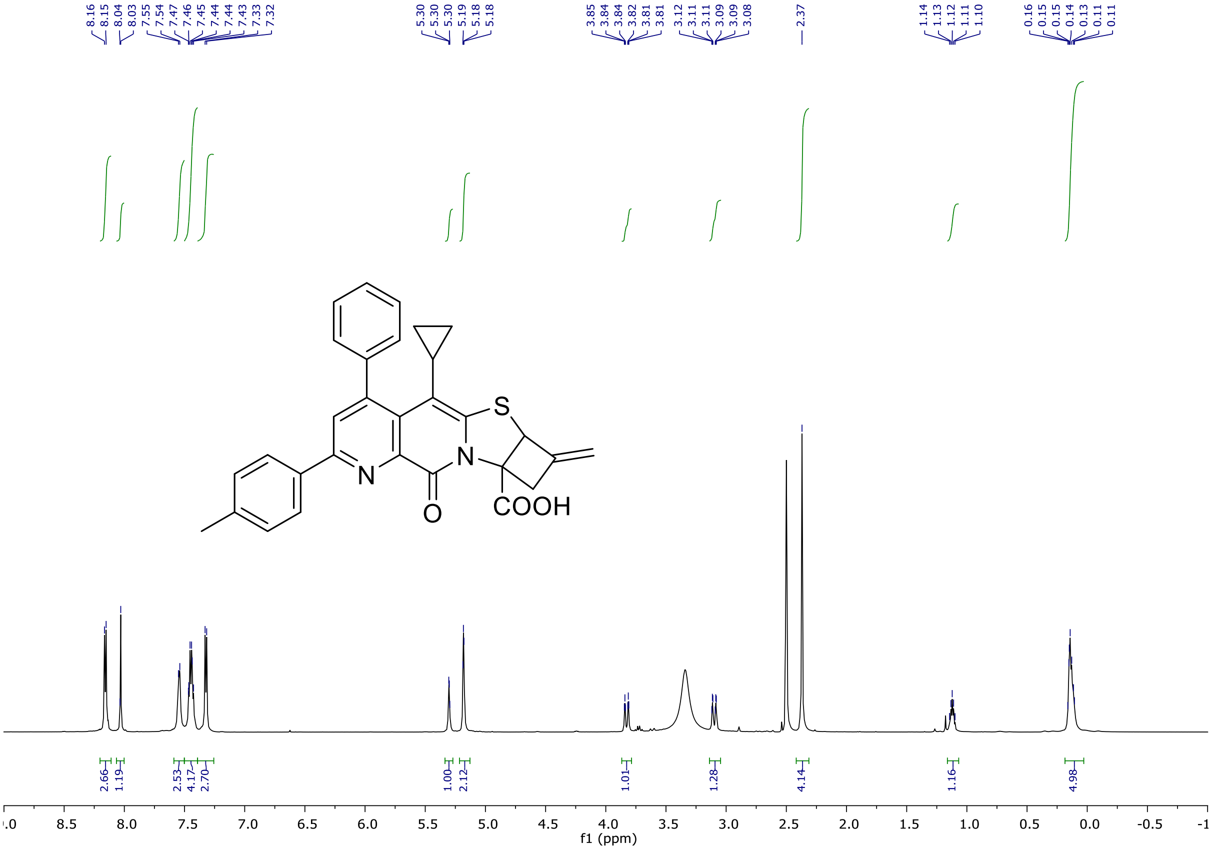

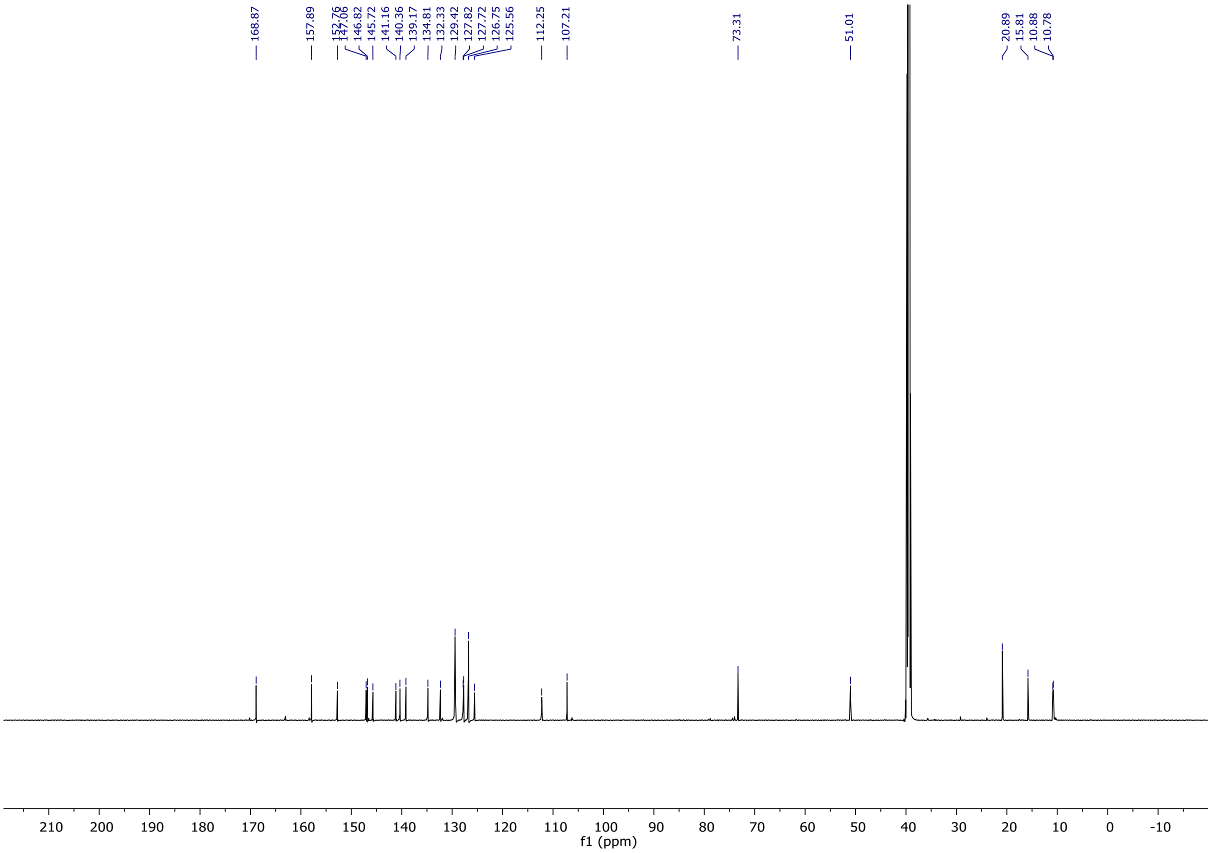

Compound **10b (-)** ^{1}H NMR [400 MHz, $(CD_3)_2SO$] ^{13}C{^{1}H} NMR [100 MHz, $(CD_3)_2SO$]

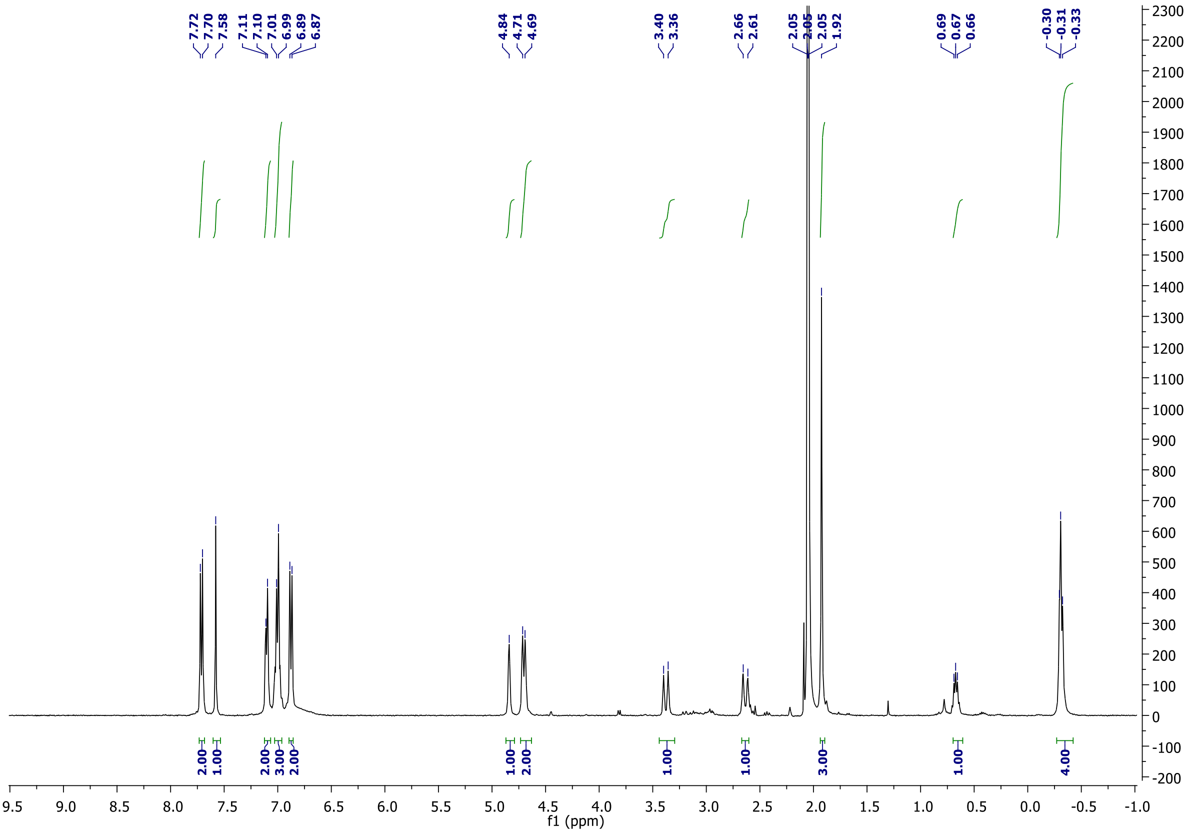

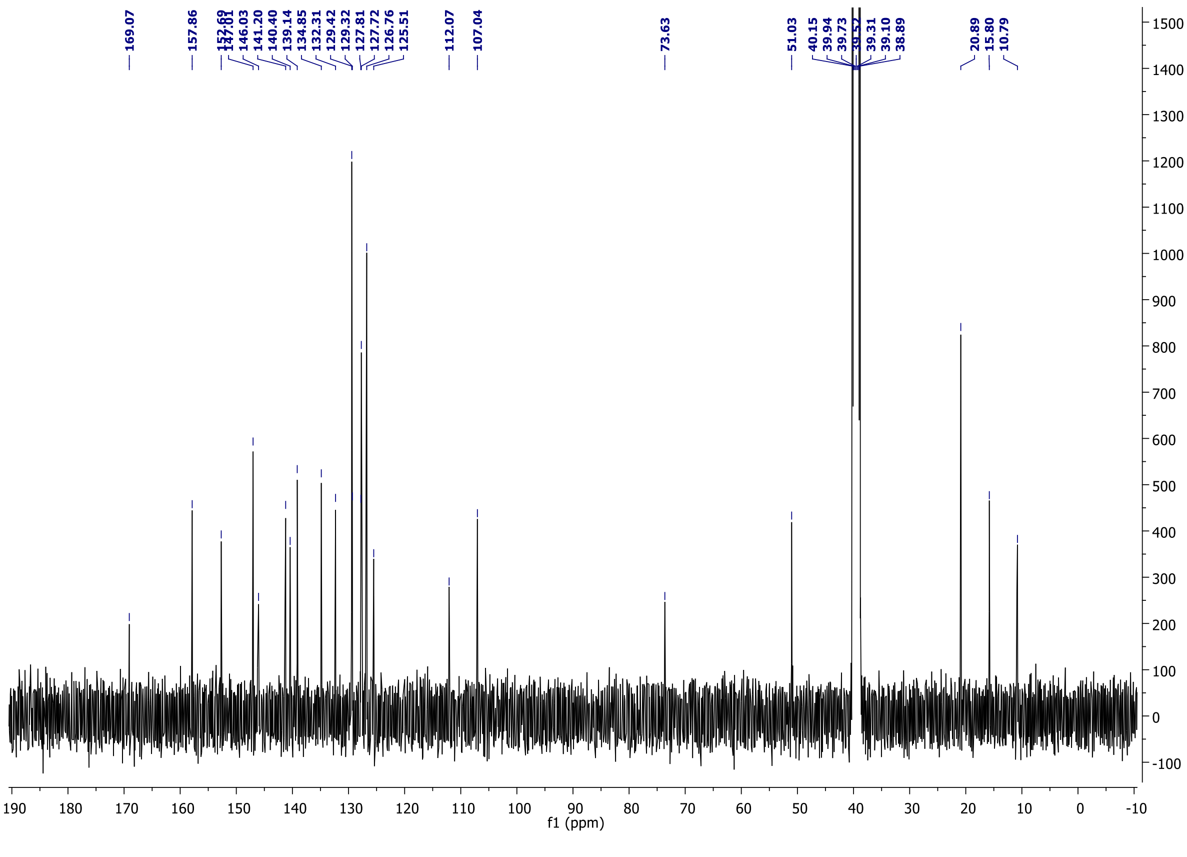

Compound **10b (+)** ^{1}H NMR [400 MHz, $(CD_3)_2SO$] $^{13}C\{^{1}H\}$ NMR [100 MHz, $(CD_3)_2SO$]

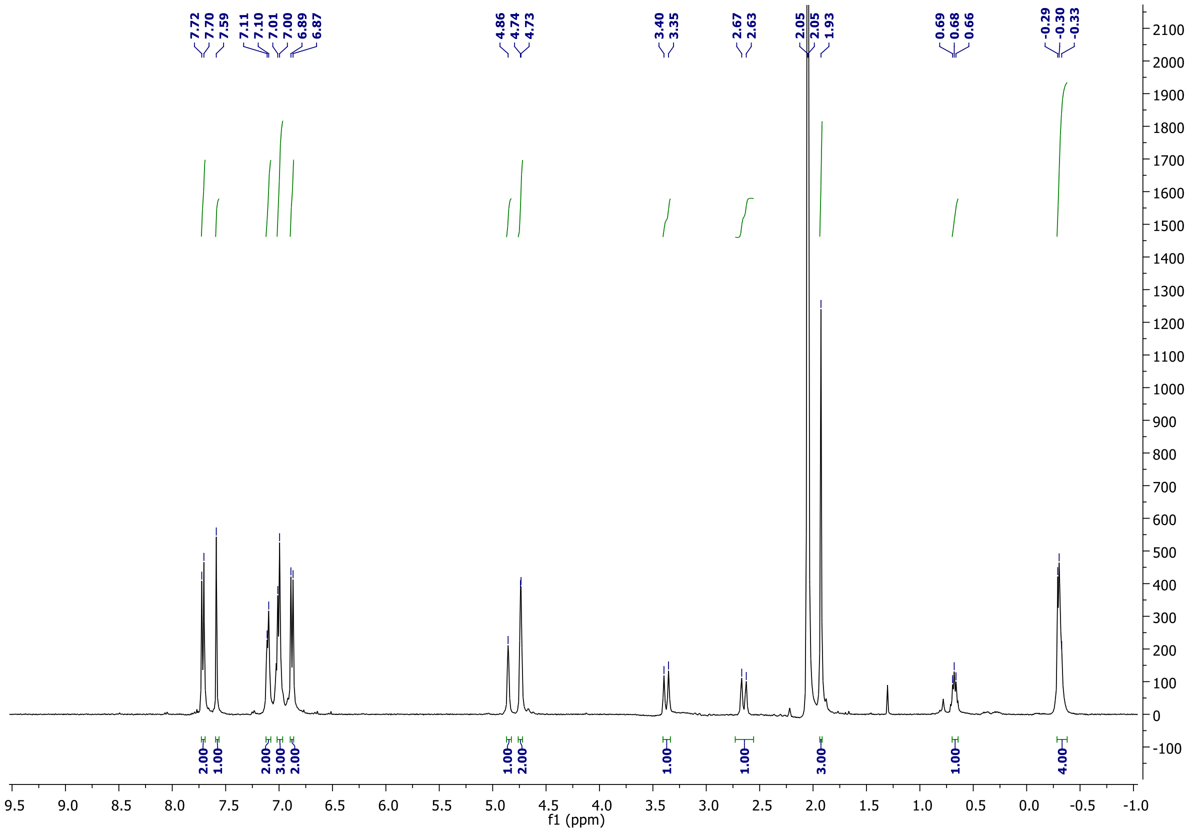

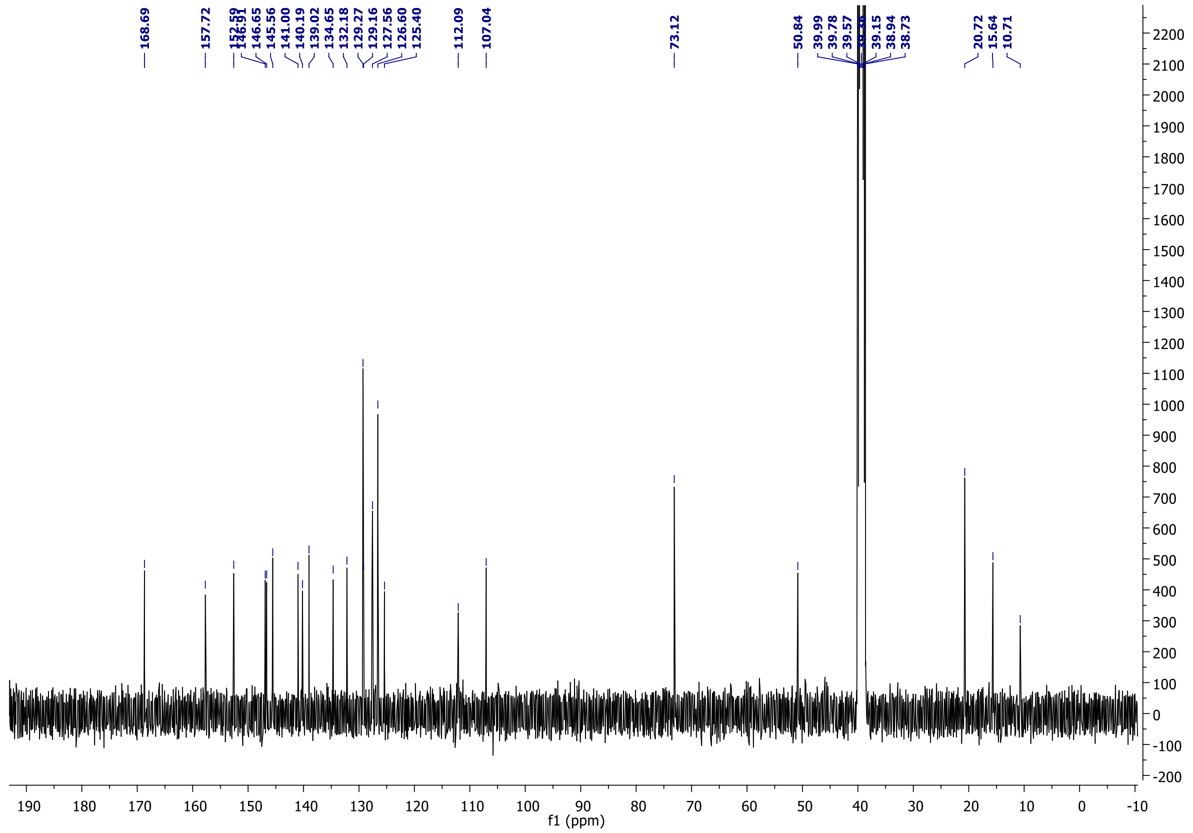

Compound **10c** ^{1}H NMR [400 MHz, $(CD_3)_2SO$] ^{13}C{^{1}H} NMR [100 MHz, $(CD_3)_2SO$] ^{19}F NMR [376 MHz, $(CD_3)_2SO$]

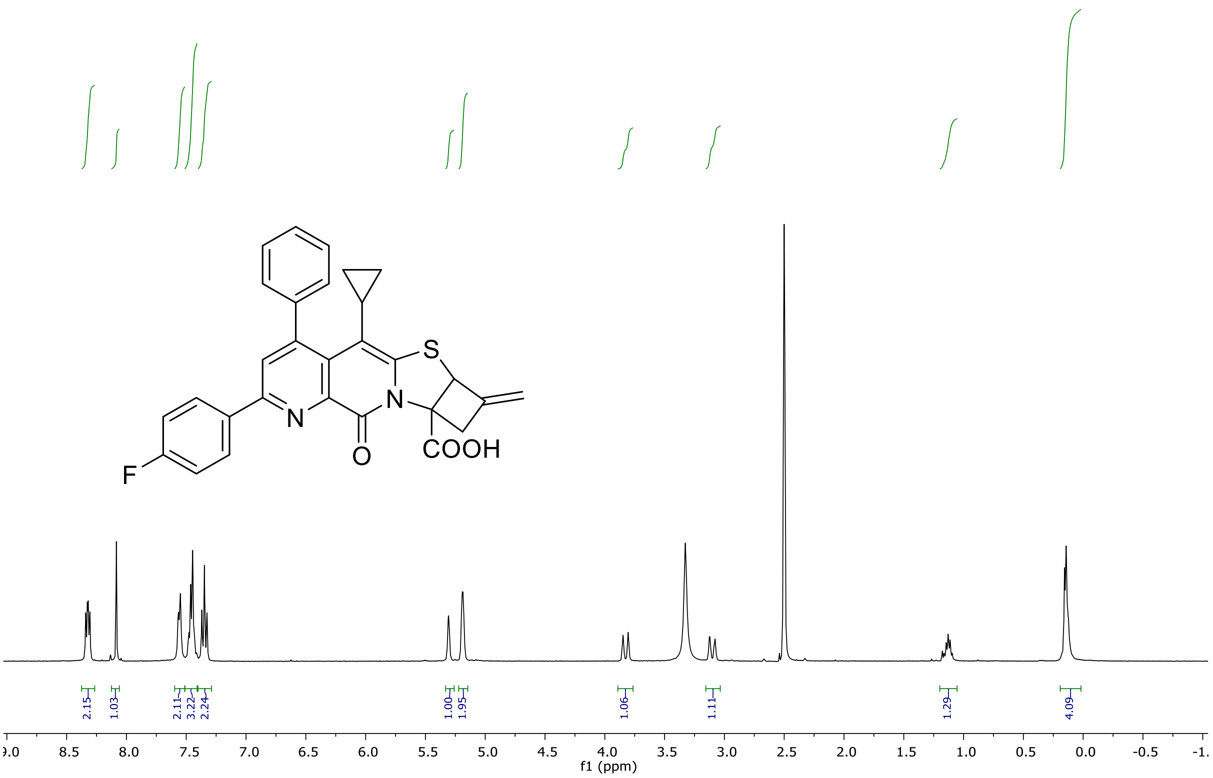

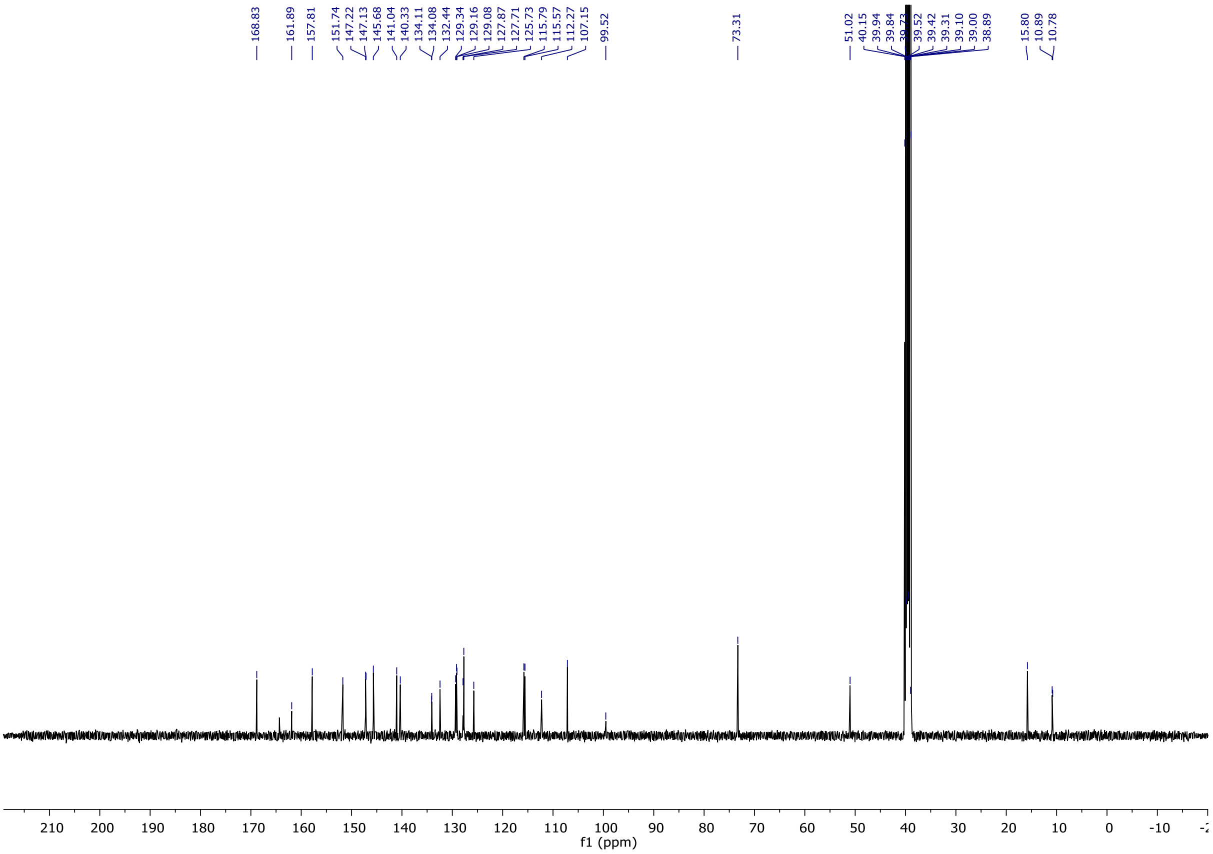

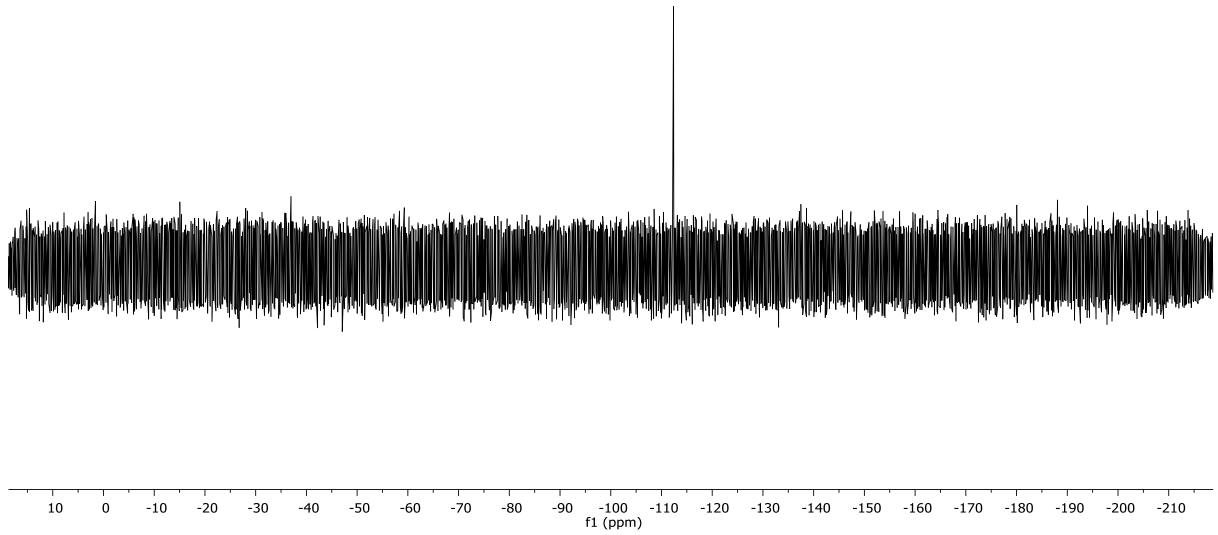
10 0 -10 -20 -30 -40 -50 -60 -70 -80 -90 -100 -110 -120 -130 -140 -150 -160 -170 -180 -190 -200 -210
f1 (ppm)

Compound **10d** ^{1}H NMR [400 MHz, $(CD_3)_2SO$] $^{13}C\{^{1}H\}$ NMR [100 MHz, $(CD_3)_2SO$]

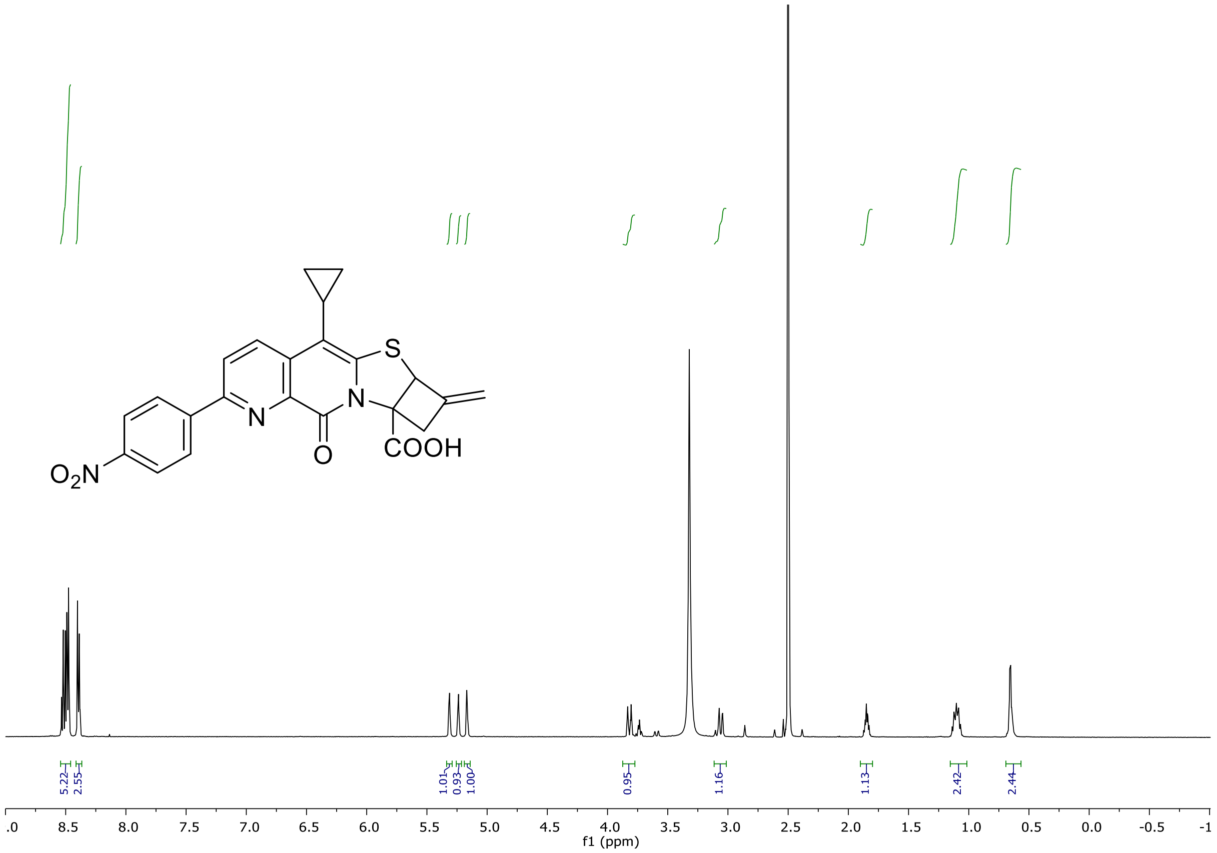

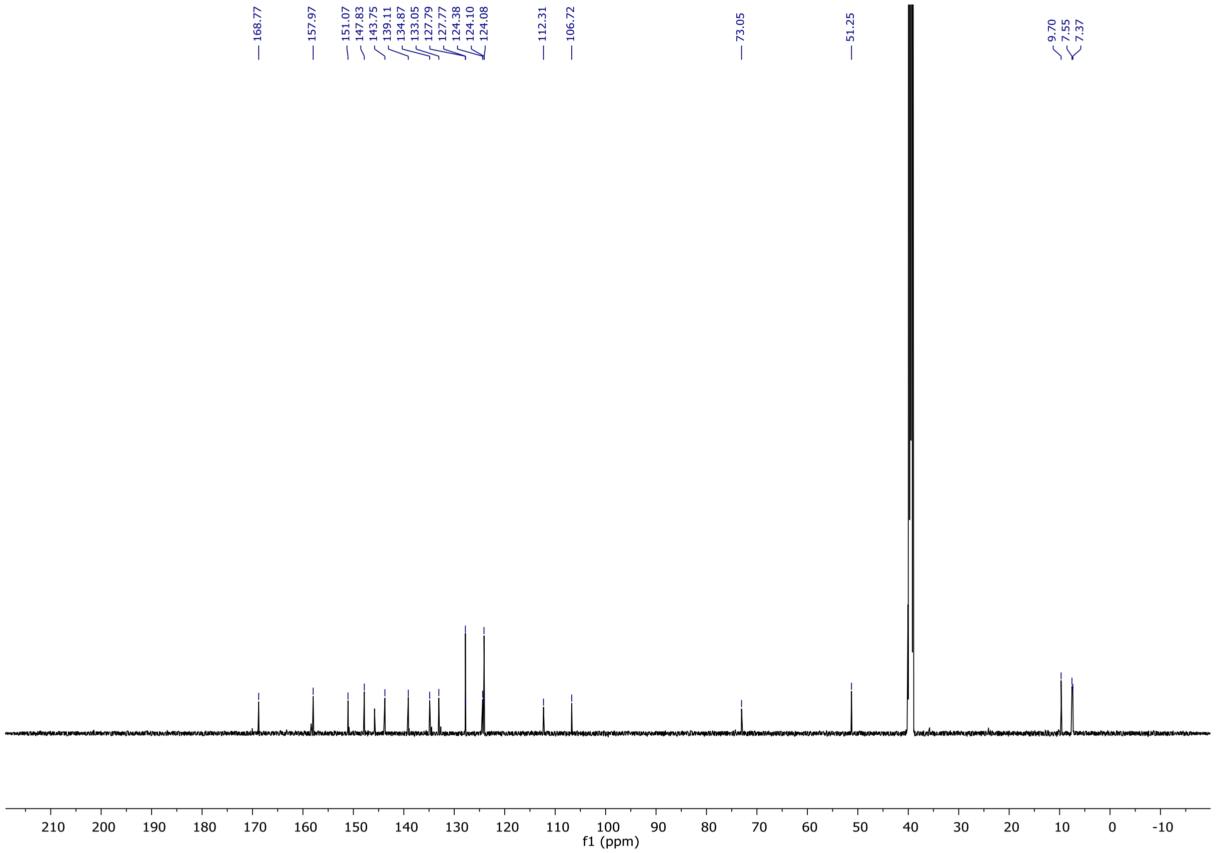

Compound **10e** ^{1}H NMR [400 MHz, $(CD_3)_2SO$] $^{13}C\{^{1}H\}$ NMR [100 MHz, $(CD_3)_2SO$]

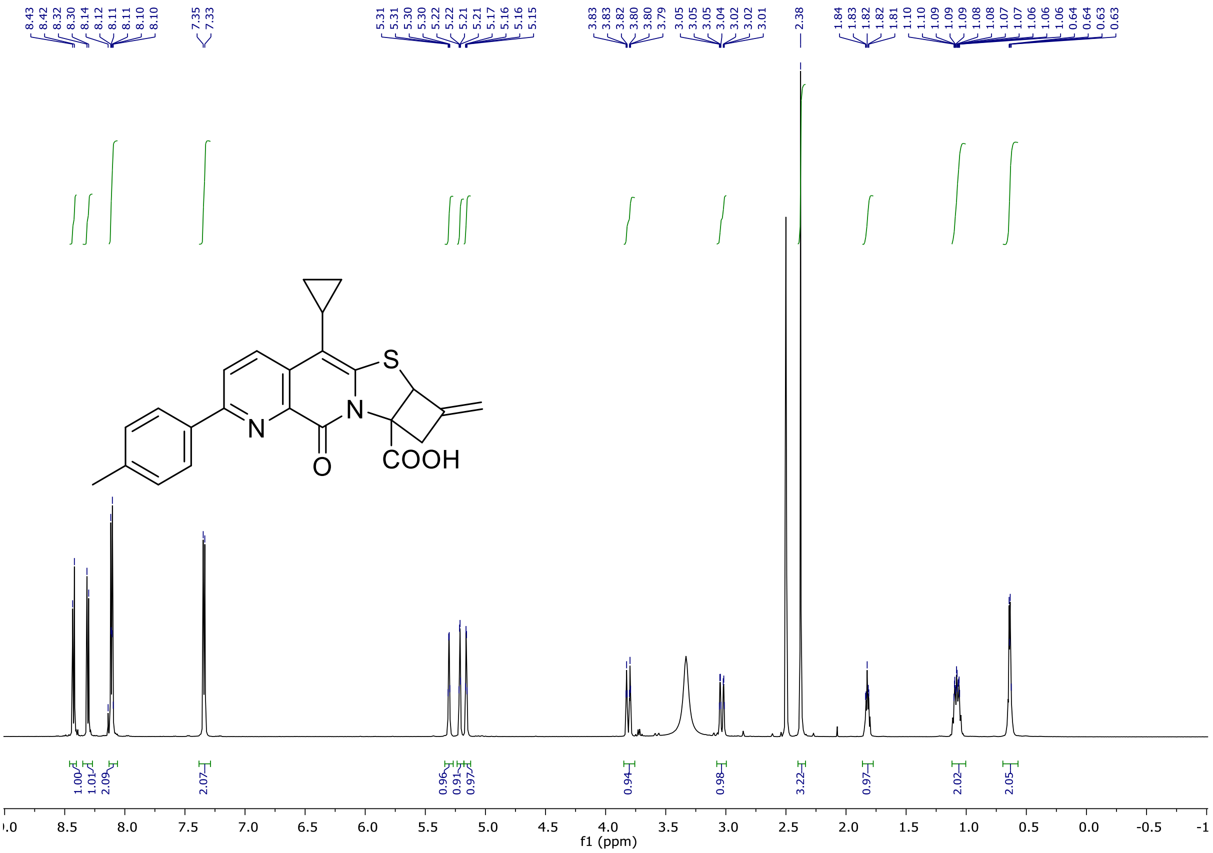

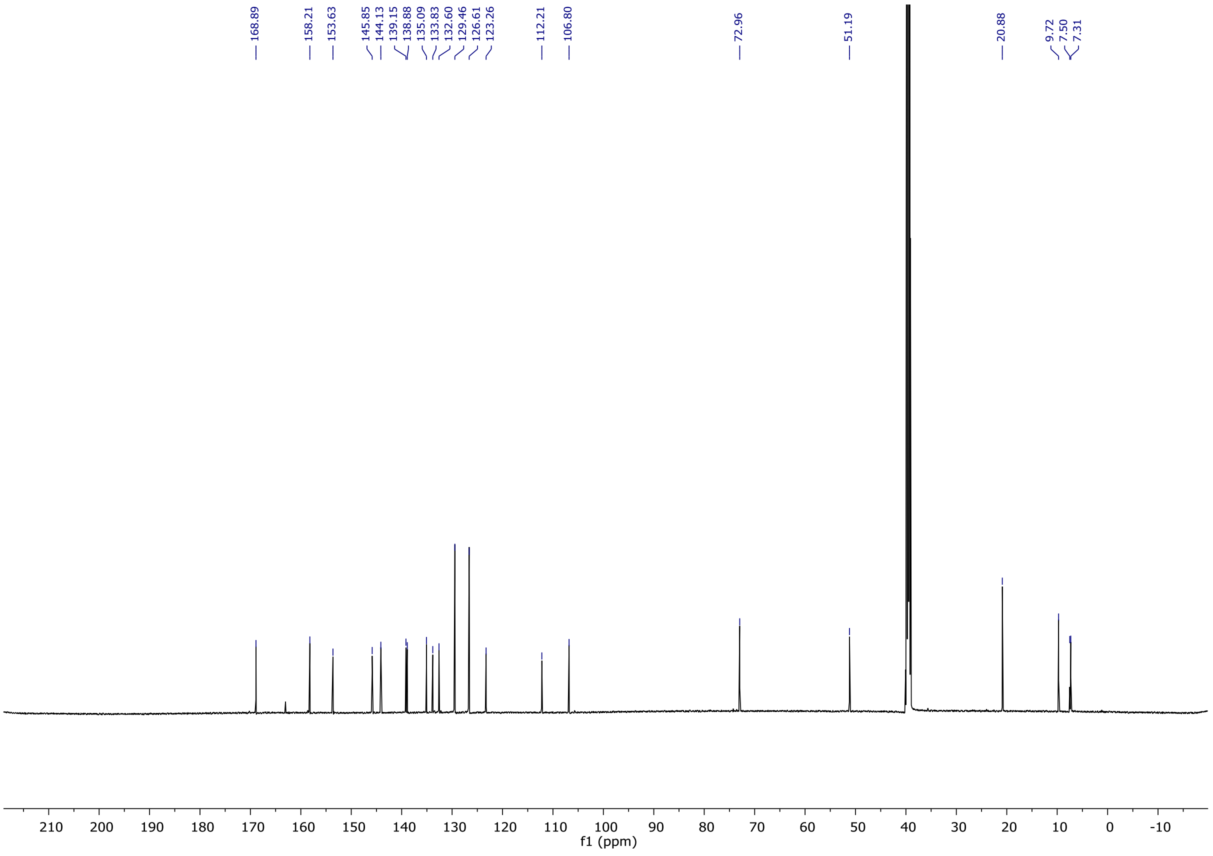

References.

1. (a) Singh, P.; Adolfsson, D. E.; Ådén, J.; Cairns, A. G.; Bartens, C.; Brännström, K.; Olofsson, A.; Almqvist, F., Pyridine-Fused 2-Pyridones via Povarov and A(3) Reactions: Rapid Generation of Highly Functionalized Tricyclic Heterocycles Capable of Amyloid Fibril Binding. *J. Org. Chem.,* **2019**, *84*, 3887–3903; (b) Singh, P.; Cairns, A. G.; Adolfsson, D. E.; Ådén, J.; Sauer, U. H.; Almqvist, F., Synthesis of Densely Functionalized N-Alkenyl 2-Pyridones via Benzyne-Induced Ring Opening of Thiazolino-Fused 2-Pyridones. *Org. Lett.,* **2019**, *21*, 6946–6950; (c) Singh, P.; Chorell, E.; Krishnan, K. S.; Kindahl, T.; Åden, J.; Wittung-Stafshede, P.; Almqvist, F., Synthesis of Multiring Fused 2-Pyridones via a Nitrene Insertion Reaction: Fluorescent Modulators of α-Synuclein Amyloid Formation. *Org. Lett.,* **2015**, *17*, 6194–6197. (d) M. Kulén, M. Lindgren, S. Hansen, A. G. Cairns, C. Grundström, A. Begum, I. van der Lingen, K. Brännström, M. Hall, U. H. Sauer, J. Johansson, A. E. Sauer-Eriksson, F. Almqvist, *J. Med. Chem.*, **2018**, *61*, 4165–4175 and the references cited therein.

2. Chorell, E.; Andersson, E.; Evans, M. L.; Jain, N.; Götheson, A.; Åden, J.; Chapman, M. R.; Almqvist, F.; Wittung-Stafshede, P. Bacterial chaperones CsgE and CsgC differentially modulate human a-synuclein amyloid formation *via* transient contacts. *PLoS ONE*., **2015**, 10(10).

3. Evans, M. L.; Chorell, E.; Taylor, J. D.; Åden, J.; Götheson, A.; Li, F.; Koch, M.; Sefer, L.; Matthews, S. J.; Wittung-Stafshede, P.; Almqvist F. and Chapman, M. R. The bacterial curli system possesses a potent and selective inhibitor of amyloid formation. *Mol. Cell,* **2015**, 57 (3), 445.

4. Brannstrom, K., Islam, T., Gharibyan, A. L., Iakovleva, I., Nilsson, L., Lee, C. C., Sandblad, L., Pamren, A., and Olofsson, A. The Properties of Amyloid-beta Fibrils Are Determined by their Path of Formation. *J. Mol. Bio.l* ***2018***. *430*, 1940-1949.

5 Nilsson, L., Pamrén, A., Islam, T., Brännström, K., Golchin, S.A., Pettersson, N., Iakovleva, I., Sandblad, L., Gharibyan, A.L. and Olofsson, A., Transthyretin interferes with Aβ amyloid formation by redirecting oligomeric nuclei into non-amyloid aggregates. *J. Mol. Biol.*, **2018**, *430* (17), 2722-2733.

6. Ono, K., Hasegawa, K., Naiki, H. and Yamada, M. Curcumin has potent anti-amyloidogenic effects for Alzheimer's β-amyloid fibrils in vitro. *J. Neurosci. Res.*, **2004**, *75* (6), 742-750.

7. Gaspar, R., Meisl, G., Buell, A. K., Young, L., Kaminski, C. F., Knowles, T. P. J., Sparr, E., and Linse, S. Secondary nucleation of monomers on fibril surface dominates alpha-synuclein aggregation and provides autocatalytic amyloid amplification. *Q Rev Biophys.* **2017**, ***50***, e6

8. Crysalis CCD; Oxford Diffraction Ltd.: Abingdon, O., UK, **2005**.

9. Crysalis RED; Oxford Diffraction Ltd.: Abingdon, O., UK, **2005**.

10. Sheldrick, G. M., Crystal structure refinement with SHELXL. *Acta Crystallographica. Section C, Structural Chemistry* **2015,** *71*, 3-8.

11. Ferrugia, L. J. J. *Appl. Cryst.* **2012**, *45*, 849-854.

12. CrystalMaker Software; Begbroke Science Park, S. L., Yarnton, Oxfordshire, OX5 1PF, United Kingdom, **2010**.
